# Supplementary material for: Na2CO3-responsive Photosynthetic and ROS Scavenging Mechanisms in Chloroplasts of Alkaligrass Revealed by Phosphoproteomics
Source: Genomics Proteomics Bioinformatics. 2020 Jul 16;18(3):271–88. doi: 10.1016/j.gpb.2018.10.011 (PMC7801222; doi:10.1016/j.gpb.2018.10.011)

Chloroplast phosphoproteome

1 Accession No. ERM94529

1.1 NPGS$_p$VNQDPIFK

#	b	b⁺⁺	Seq.	y	y⁺⁺	#
1	151.126	76.067	N			12
2	248.179	124.593	P	1317.663	659.335	11
3	305.200	153.104	G	1220.610	610.809	10
4	472.198	236.603	S	1163.589	582.298	9
5	571.267	286.137	V	996.591	498.799	8
6	685.310	343.159	N	897.522	449.265	7
7	813.368	407.188	Q	783.479	392.243	6
8	928.395	464.701	D	655.421	328.214	5
9	1025.448	513.228	P	540.394	270.700	4
10	1138.532	569.770	I	443.341	222.174	3
11	1285.601	643.304	F	330.257	165.632	2
12			K	183.188	92.098	1

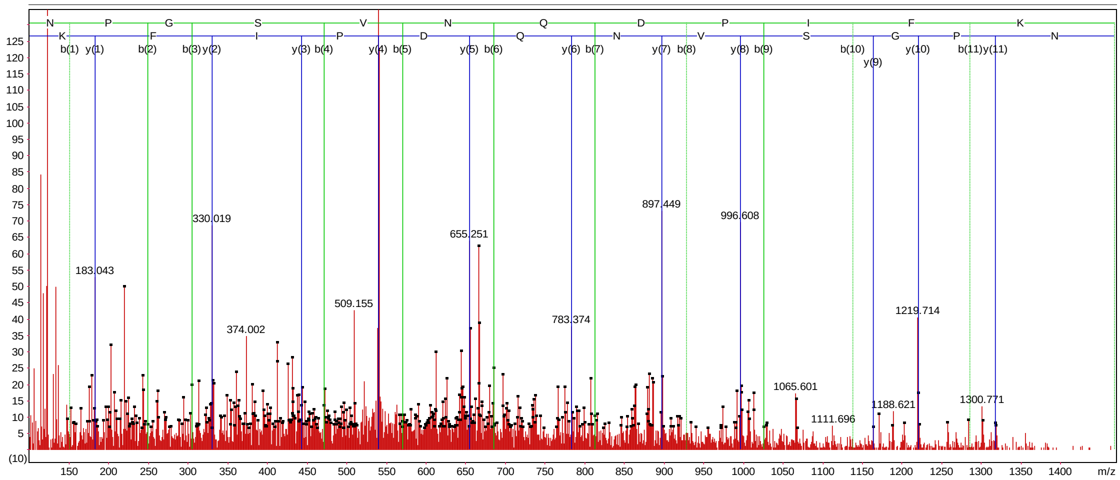

2 Accession No. BAK03973

2.1 VAAS$_p$SSPWYGSDR

#	b	b⁺⁺	Seq.	y	y⁺⁺	#
1	132.132	66.570	V			13
2	203.169	102.088	A	1363.531	682.269	12
3	274.206	137.607	A	1292.494	646.751	11
4	441.205	221.106	S	1221.457	611.232	10
5	528.237	264.622	S	1054.459	527.733	9
6	615.269	308.138	S	967.427	484.217	8
7	712.322	356.664	P	880.395	440.701	7
8	898.401	449.704	W	783.342	392.175	6
9	1061.464	531.236	Y	597.263	299.135	5
10	1118.486	559.746	G	434.199	217.603	4
11	1205.518	603.262	S	377.178	189.093	3
12	1320.545	660.776	D	290.146	145.577	2
13			R	175.119	88.063	1

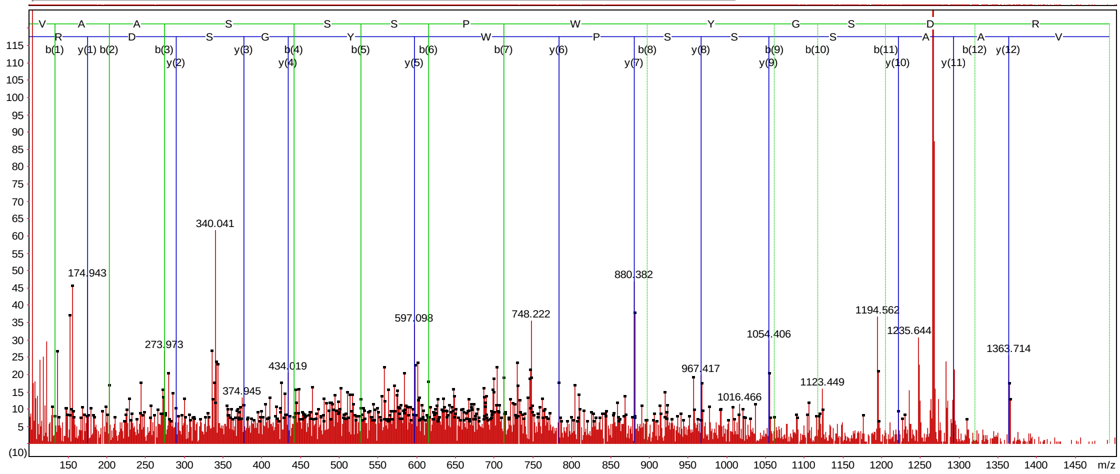

2.2 VAASSS$_p$PWYGSDR

#	b	b⁺⁺	Seq.	y	y⁺⁺	#
1	136.151	68.579	V			13
2	207.188	104.098	A	1363.531	682.269	12
3	278.226	139.616	A	1292.494	646.751	11
4	365.258	183.132	S	1221.457	611.232	10
5	452.290	226.648	S	1134.425	567.716	9
6	619.288	310.148	S	1047.393	524.200	8
7	716.341	358.674	P	880.395	440.701	7
8	902.420	451.714	W	783.342	392.175	6
9	1065.483	533.245	Y	597.263	299.135	5
10	1122.505	561.756	G	434.199	217.603	4
11	1209.537	605.272	S	377.178	189.093	3
12	1324.564	662.786	D	290.146	145.577	2
13			R	175.119	88.063	1

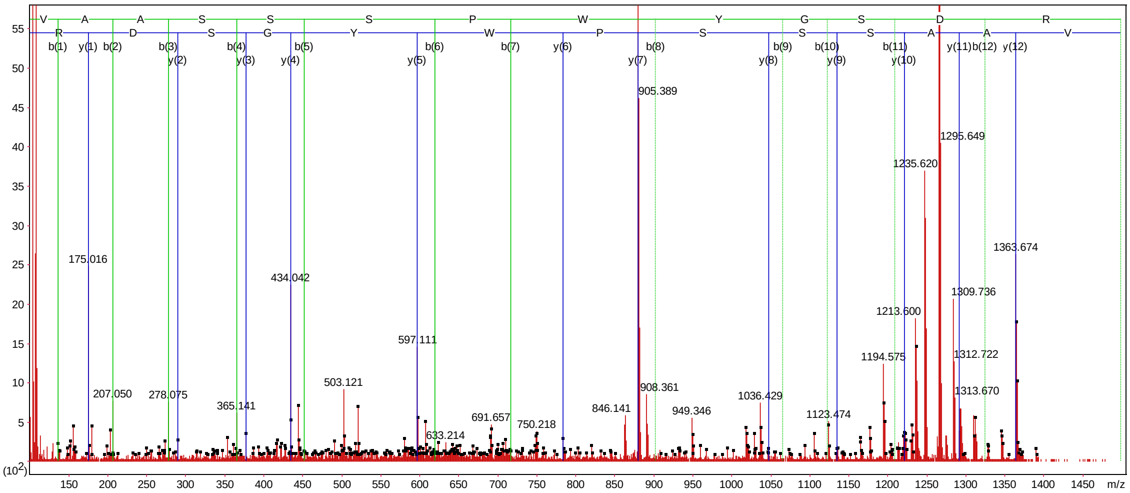

2.3 VAASSSPWYGS$_p$DR

#	b	b⁺⁺	Seq.	y	y⁺⁺	#
1	136.151	68.579	V			13
2	207.188	104.098	A	1363.531	682.269	12
3	278.226	139.616	A	1292.494	646.751	11
4	365.258	183.132	S	1221.457	611.232	10
5	452.290	226.648	S	1134.425	567.716	9
6	539.322	270.164	S	1047.393	524.200	8
7	636.374	318.691	P	960.361	480.684	7
8	822.454	411.731	W	863.308	432.158	6
9	985.517	493.262	Y	677.229	339.118	5
10	1042.539	521.773	G	514.166	257.586	4
11	1209.537	605.272	S	457.144	229.076	3
12	1324.564	662.786	D	290.146	145.577	2
13			R	175.119	88.063	1

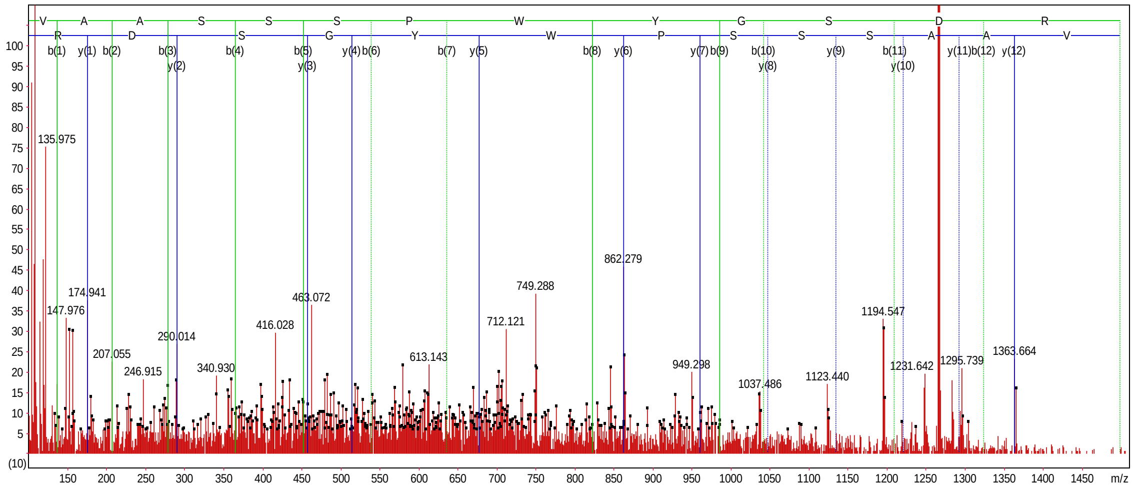

3 Accession No. ADL41158

3.1 AKPVS$_p$SGSPWYGSDR

#	b	b⁺⁺	Seq.	y	y⁺⁺	#
1	104.101	52.554	A			15
2	264.252	132.630	K	1634.751	817.879	14
3	361.305	181.156	P	1474.600	737.804	13
4	460.373	230.690	V	1377.547	689.277	12
5	627.372	314.189	S	1278.479	639.743	11
6	714.404	357.706	S	1111.480	556.244	10
7	771.425	386.216	G	1024.448	512.728	9
8	858.457	429.732	S	967.427	484.217	8
9	955.510	478.259	P	880.395	440.701	7
10	1141.589	571.298	W	783.342	392.175	6
11	1304.653	652.830	Y	597.263	299.135	5
12	1361.674	681.341	G	434.199	217.603	4
13	1448.706	724.857	S	377.178	189.093	3
14	1563.733	782.370	D	290.146	145.577	2
15			R	175.119	88.063	1

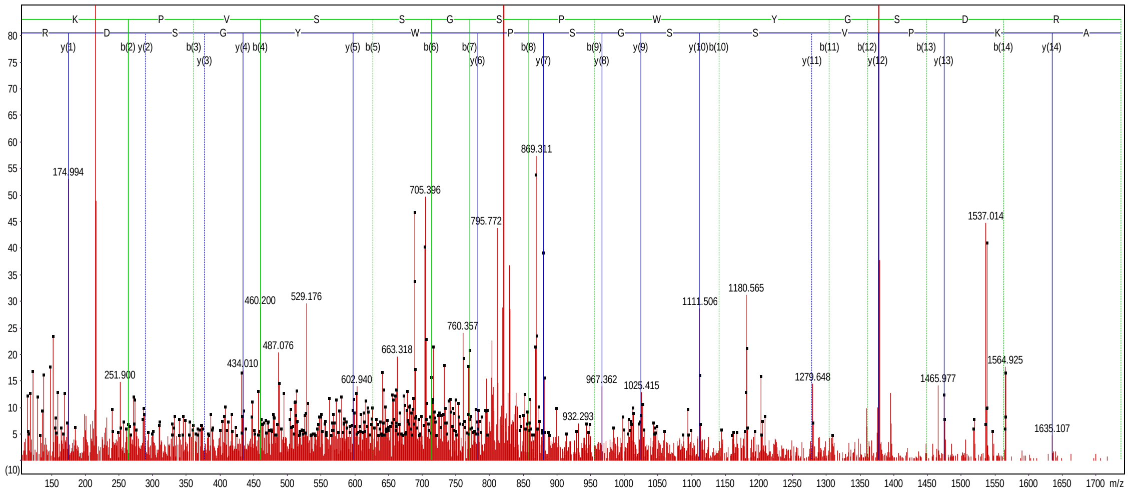

3.2 AKPVSSGS$_p$PWYGSDR

#	b	b⁺⁺	Seq.	y	y⁺⁺	#
1	108.120	54.564	A			15
2	272.291	136.649	K	1638.770	819.889	14
3	369.343	185.175	P	1474.600	737.804	13
4	468.412	234.710	V	1377.547	689.277	12
5	555.444	278.226	S	1278.479	639.743	11
6	642.476	321.742	S	1191.447	596.227	10
7	699.497	350.252	G	1104.415	552.711	9
8	866.496	433.752	S	1047.393	524.200	8
9	963.549	482.278	P	880.395	440.701	7
10	1149.628	575.318	W	783.342	392.175	6
11	1312.691	656.849	Y	597.263	299.135	5
12	1369.713	685.360	G	434.199	217.603	4
13	1456.745	728.876	S	377.178	189.093	3
14	1571.772	786.389	D	290.146	145.577	2
15			R	175.119	88.063	1

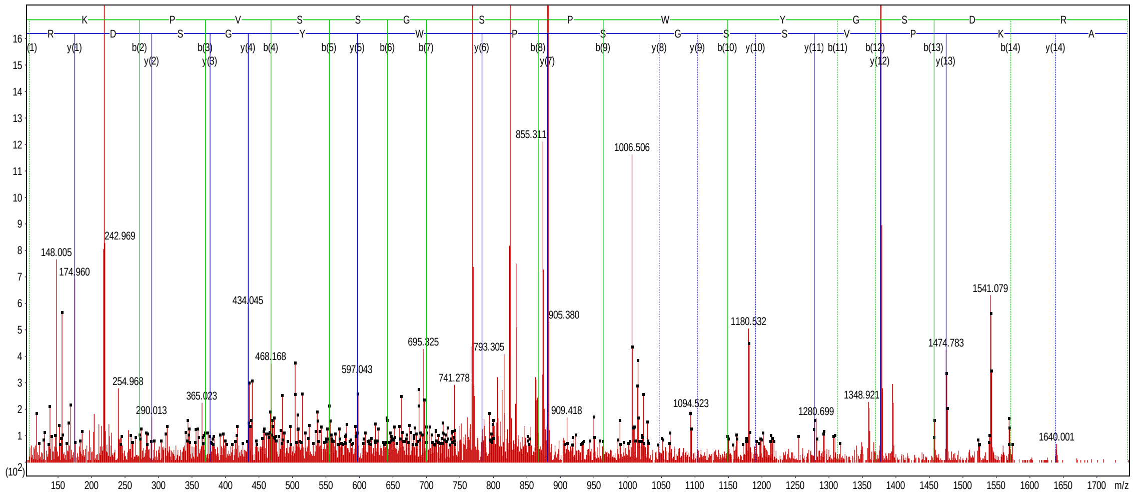

3.3 AKPVSSGSPWYGS$_p$DR

#	b	b⁺⁺	Seq.	y	y⁺⁺	#
1	108.120	54.564	A			15
2	272.291	136.649	K	1638.770	819.889	14
3	369.343	185.175	P	1474.600	737.804	13
4	468.412	234.710	V	1377.547	689.277	12
5	555.444	278.226	S	1278.479	639.743	11
6	642.476	321.742	S	1191.447	596.227	10
7	699.497	350.252	G	1104.415	552.711	9
8	786.529	393.768	S	1047.393	524.200	8
9	883.582	442.295	P	960.361	480.684	7
10	1069.661	535.334	W	863.308	432.158	6
11	1232.725	616.866	Y	677.229	339.118	5
12	1289.746	645.377	G	514.166	257.586	4
13	1456.745	728.876	S	457.144	229.076	3
14	1571.772	786.389	D	290.146	145.577	2
15			R	175.119	88.063	1

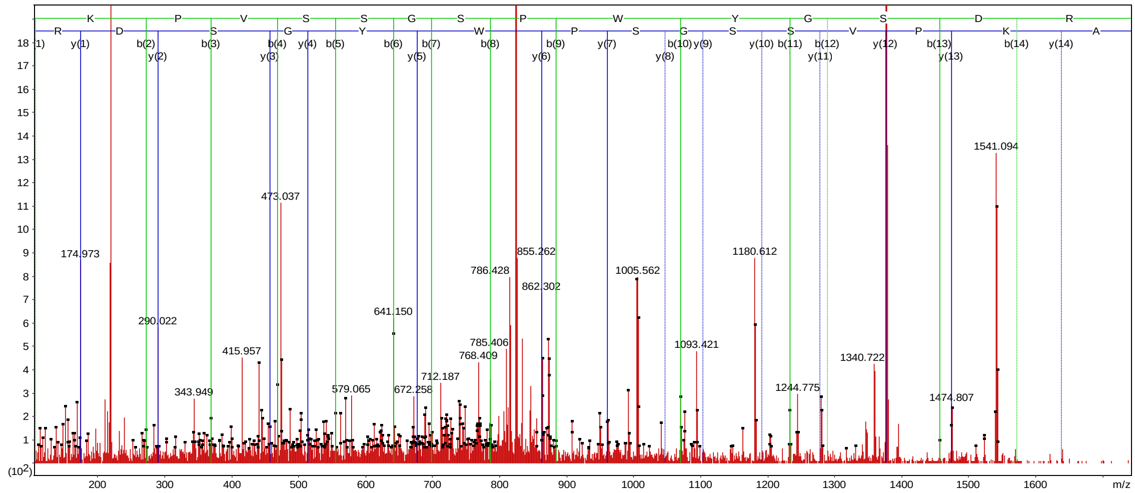

4 Accession No. BAF12500

4.1 TVKS$_p$APQSIWYGPDRPK

#	b	b⁺⁺	Seq.	y	y⁺⁺	#
1	130.086	65.547	T			17
2	229.155	115.081	V	1964.999	983.003	16
3	385.281	193.144	K	1865.931	933.469	15
4	552.279	276.643	S	1709.805	855.406	14
5	623.316	312.162	A	1542.806	771.907	13
6	720.369	360.688	P	1471.769	736.388	12
7	848.428	424.718	Q	1374.716	687.862	11
8	935.460	468.234	S	1246.658	623.833	10
9	1048.544	524.776	I	1159.626	580.317	9
10	1234.623	617.815	W	1046.542	523.775	8
11	1397.686	699.347	Y	860.462	430.735	7
12	1454.708	727.858	G	697.399	349.203	6
13	1551.761	776.384	P	640.378	320.692	5
14	1666.788	833.897	D	543.325	272.166	4
15	1822.889	911.948	R	428.298	214.653	3
16	1919.942	960.474	P	272.197	136.602	2
17			K	175.144	88.076	1

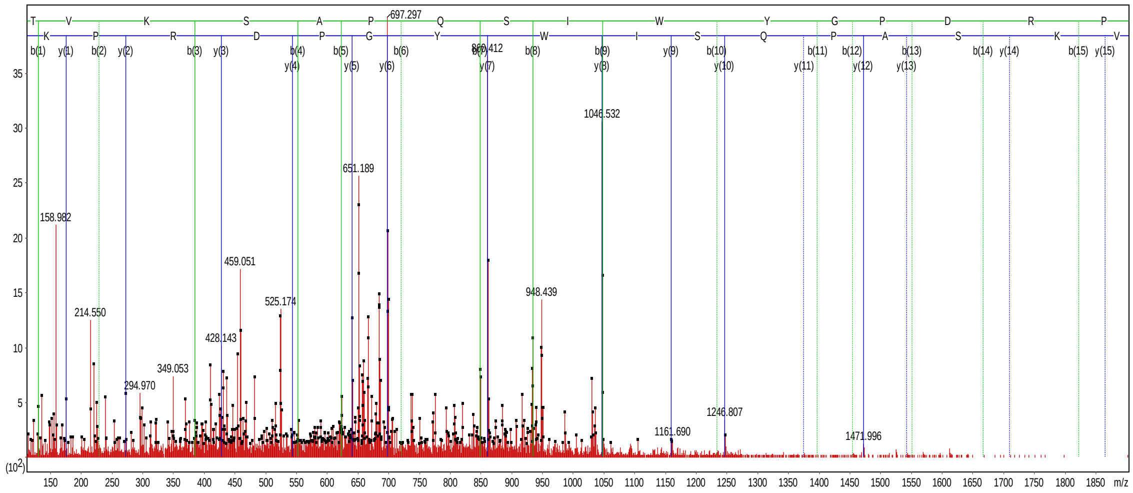

5 Accession No. BAJ85110

5.1 QVSS$_p$GSPWYGADR

#	b	b++	Seq.	y	y++	#
1	165.142	83.074	Q			13
2	264.210	132.609	V	1361.552	681.280	12
3	351.242	176.125	S	1262.484	631.746	11
4	518.240	259.624	S	1175.452	588.230	10
5	575.262	288.135	G	1008.453	504.730	9
6	662.294	331.651	S	951.432	476.220	8
7	759.347	380.177	P	864.400	432.704	7
8	945.426	473.217	W	767.347	384.177	6
9	1108.489	554.748	Y	581.268	291.138	5
10	1165.511	583.259	G	418.204	209.606	4
11	1236.548	618.778	A	361.183	181.095	3
12	1351.575	676.291	D	290.146	145.577	2
13			R	175.119	88.063	1

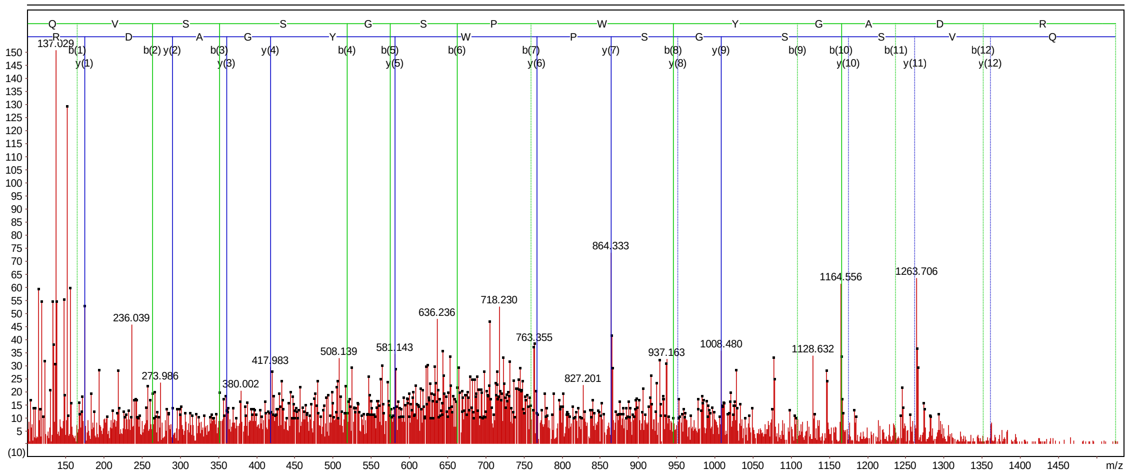

6 Accession No. P27523

6.1 IT$_p$MGNDLWYGPDR

#1	b+	b2+	Seq.	y+	y2+	#2
1	150.16702	75.58715	I-Dimethyl:...			13
2	331.18103	166.09415	T-Phospho	1504.59272	752.80000	12
3	462.22153	231.61440	M	1323.57871	662.29299	11
4	519.24300	260.12514	G	1192.53821	596.77274	10
5	633.28593	317.14660	N	1135.51674	568.26201	9
6	748.31288	374.66008	D	1021.47381	511.24054	8
7	861.39695	431.20211	L	906.44686	453.72707	7
8	1047.47627	524.24177	W	793.36279	397.18503	6
9	1210.53959	605.77343	Y	607.28347	304.14537	5
10	1267.56106	634.28417	G	444.22015	222.61371	4
11	1364.61383	682.81055	P	387.19868	194.10298	3
12	1479.64078	740.32403	D	290.14591	145.57659	2
13			R	175.11896	88.06312	1

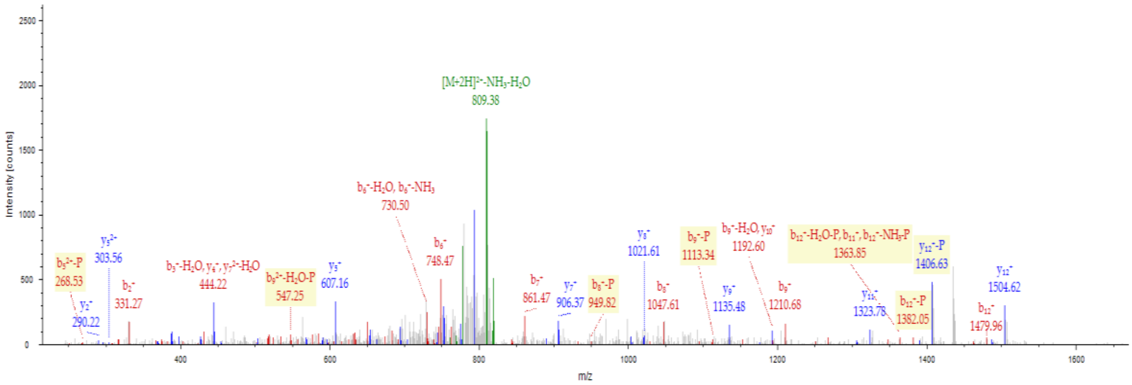

7 Accession No. XP_003579885

7.1 TADNFANS$_p$TGDQGYPGGK

#	b	b⁺⁺	Seq.	y	y⁺⁺	#
1	138.131	69.569	T			18
2	209.168	105.088	A	1814.777	907.892	17
3	324.195	162.601	D	1743.740	872.374	16
4	438.238	219.622	N	1628.713	814.860	15
5	585.306	293.157	F	1514.670	757.839	14
6	656.343	328.675	A	1367.602	684.305	13
7	770.386	385.697	N	1296.565	648.786	12
8	937.384	469.196	S	1182.522	591.765	11
9	1038.432	519.720	T	1015.524	508.265	10
10	1095.454	548.230	G	914.476	457.742	9
11	1210.481	605.744	D	857.454	429.231	8
12	1338.539	669.773	Q	742.428	371.717	7
13	1395.561	698.284	G	614.369	307.688	6
14	1558.624	779.816	Y	557.347	279.177	5
15	1655.677	828.342	P	394.284	197.646	4
16	1712.698	856.853	G	297.231	149.119	3
17	1769.720	885.363	G	240.210	120.609	2
18			K	183.188	92.098	1

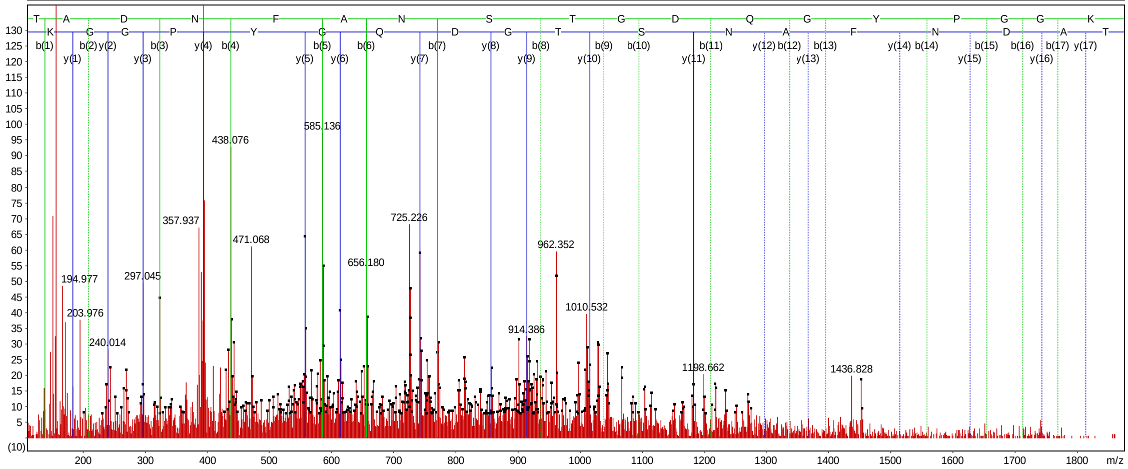

8 Accession No. A1EA37

8.1 AT$_p$QTVEDSSKPR

#	b	b⁺⁺	Seq.	y	y⁺⁺	#
1	100.076	50.541	A			12
2	281.090	141.048	T	1355.620	678.314	11
3	409.148	205.078	Q	1174.606	587.807	10
4	510.196	255.602	T	1046.548	523.777	9
5	609.264	305.136	V	945.500	473.254	8
6	738.307	369.657	E	846.432	423.719	7
7	853.334	427.171	D	717.389	359.198	6
8	940.366	470.687	S	602.362	301.685	5
9	1027.398	514.203	S	515.330	258.169	4
10	1183.524	592.266	K	428.298	214.653	3
11	1280.577	640.792	P	272.172	136.589	2
12			R	175.119	88.063	1

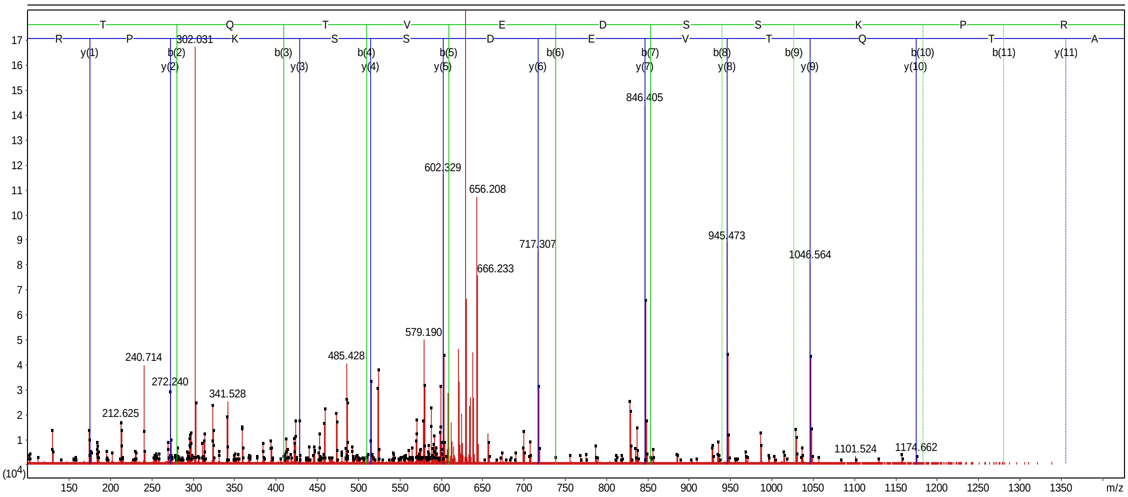

8.2 AT$_p$QT$_p$VEDSSKPRPK

#	b	b⁺⁺	Seq.	y	y⁺⁺	#
1	100.076	50.541	A			12
2	281.090	141.048	T	1435.587	718.297	11
3	409.148	205.078	Q	1254.573	627.790	10
4	590.162	295.585	T	1126.514	563.761	9
5	689.231	345.119	V	945.500	473.254	8
6	818.273	409.640	E	846.432	423.719	7
7	933.300	467.154	D	717.389	359.198	6
8	1020.332	510.670	S	602.362	301.685	5
9	1107.364	554.186	S	515.330	258.169	4
10	1263.491	632.249	K	428.298	214.653	3
11	1360.543	680.775	P	272.172	136.589	2
12			R	175.119	88.063	1

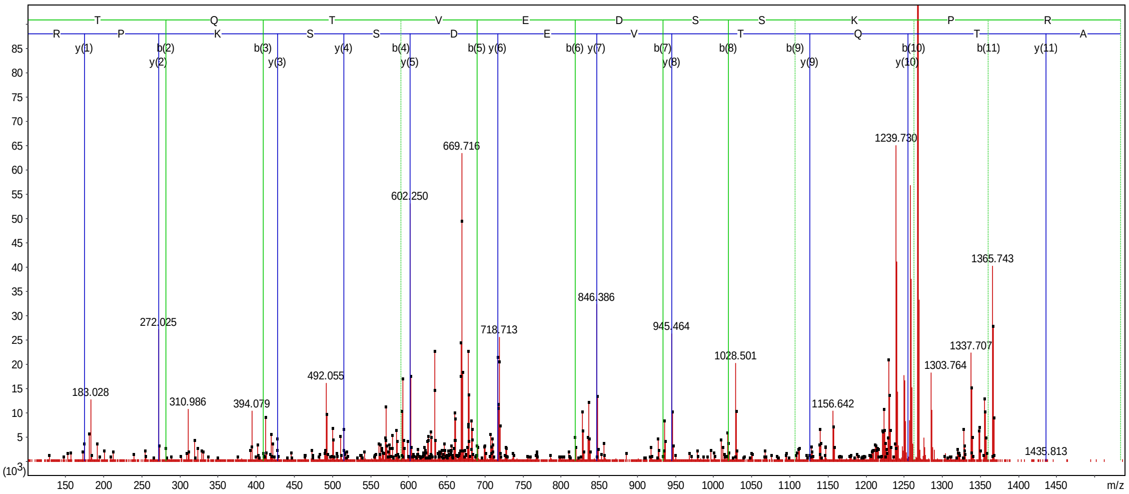

9 Accession No. ACF08666

9.1 AT$_p$QT$_p$VEDSSKPKPR

#	b	b⁺⁺	Seq.	y	y⁺⁺	#
1	100.076	50.541	A			14
2	281.090	141.048	T	1688.766	844.886	13
3	409.148	205.078	Q	1507.752	754.379	12
4	590.162	295.585	T	1379.693	690.350	11
5	689.231	345.119	V	1198.679	599.843	10
6	818.273	409.640	E	1099.611	550.309	9
7	933.300	467.154	D	970.568	485.788	8
8	1020.332	510.670	S	855.541	428.274	7
9	1107.364	554.186	S	768.509	384.758	6
10	1263.491	632.249	K	681.477	341.242	5
11	1360.543	680.775	P	525.351	263.179	4
12	1516.670	758.838	K	428.298	214.653	3
13	1613.722	807.365	P	272.172	136.589	2
14			R	175.119	88.063	1

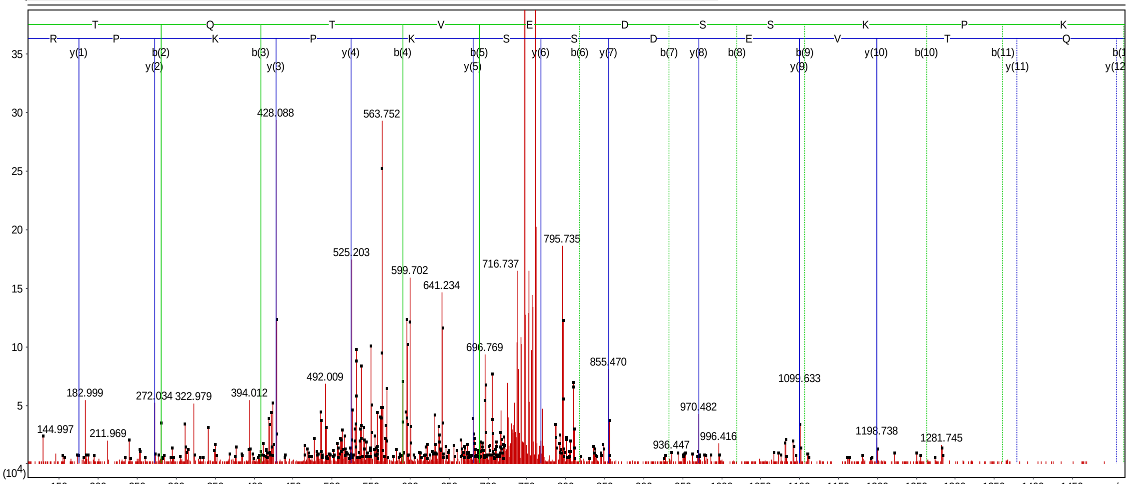

10 Accession No. EMT33794

10.1 QLVATGKPES$_p$FSGPFLVPSYR

#	b	b++	Seq.	y	y++	#
1	157.097	79.052	Q			21
2	270.181	135.594	L	2260.141	1130.574	20
3	369.250	185.128	V	2147.057	1074.032	19
4	440.287	220.647	A	2047.989	1024.498	18
5	541.334	271.171	T	1976.952	988.980	17
6	598.356	299.682	G	1875.904	938.456	16
7	754.482	377.745	K	1818.883	909.945	15
8	851.535	426.271	P	1662.756	831.882	14
9	980.578	490.792	E	1565.704	783.355	13
10	1147.576	574.292	S	1436.661	718.834	12
11	1294.644	647.826	F	1269.663	635.335	11
12	1381.676	691.342	S	1122.594	561.801	10
13	1438.698	719.853	G	1035.562	518.285	9
14	1535.751	768.379	P	978.541	489.774	8
15	1682.819	841.913	F	881.488	441.248	7
16	1795.903	898.455	L	734.420	367.713	6
17	1894.971	947.989	V	621.335	311.171	5
18	1992.024	996.516	P	522.267	261.637	4
19	2079.056	1040.032	S	425.214	213.111	3
20	2242.120	1121.563	Y	338.182	169.595	2
21			R	175.119	88.063	1

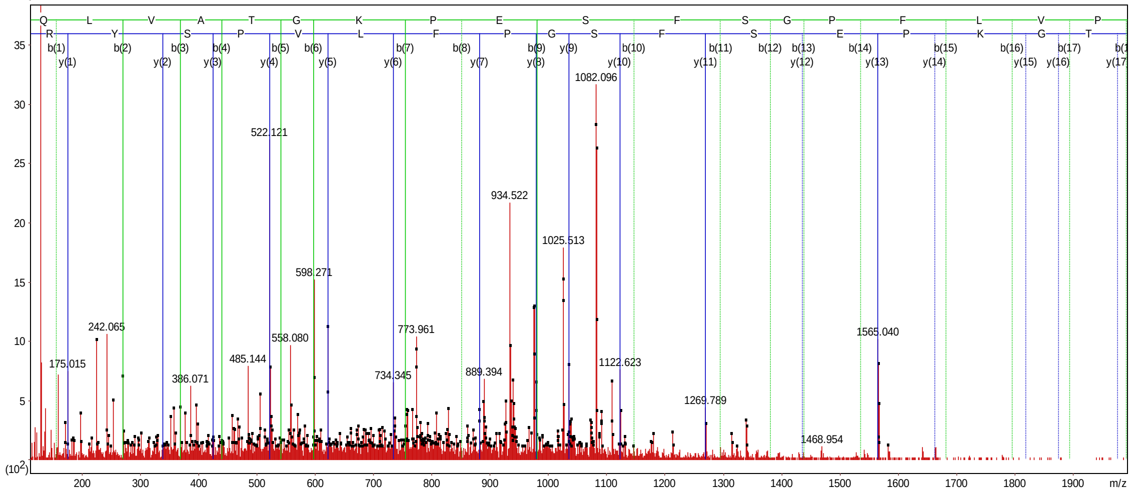

11 Accession No. EMS62589

11.1 SYAS$_p$NNELAVMPK

#	b	b++	Seq.	y	y++	#
1	116.071	58.539	S			13
2	279.134	140.071	Y	1444.654	722.831	12
3	350.171	175.589	A	1281.591	641.299	11
4	517.169	259.088	S	1210.554	605.781	10
5	631.212	316.110	N	1043.555	522.281	9
6	745.255	373.131	N	929.512	465.260	8
7	874.298	437.653	E	815.470	408.238	7
8	987.382	494.195	L	686.427	343.717	6
9	1058.419	529.713	A	573.343	287.175	5
10	1157.487	579.247	V	502.306	251.657	4
11	1288.528	644.768	M	403.237	202.122	3
12	1385.581	693.294	P	272.197	136.602	2
13			K	175.144	88.076	1

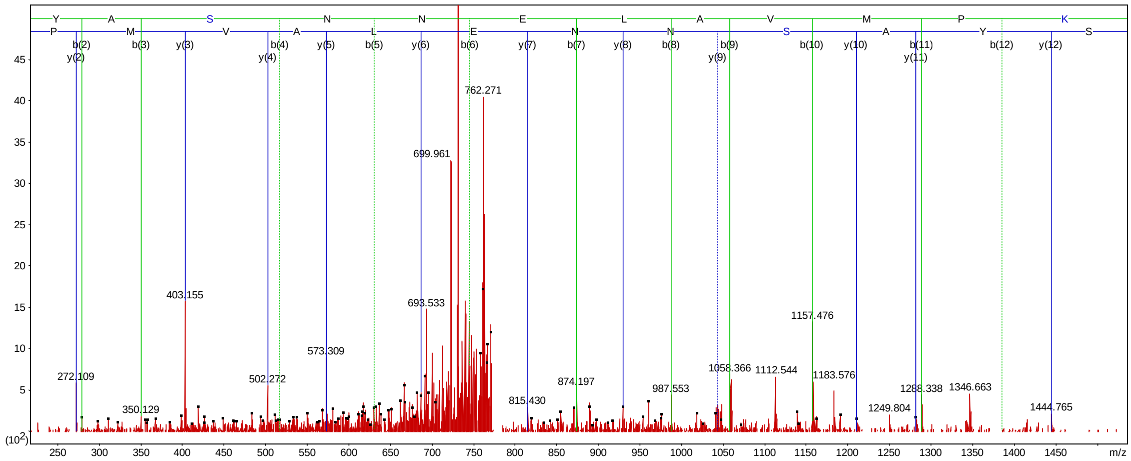

12 Accession No. AAN32350

12.1 T$_p$ASNPNEQNVELNR

#	b	b++	Seq.	y	y++	#
1	218.097	109.552	T			14
2	289.134	145.071	A	1484.709	742.858	13
3	376.166	188.587	S	1413.672	707.339	12
4	490.209	245.608	N	1326.640	663.823	11
5	587.262	294.135	P	1212.597	606.802	10
6	701.305	351.156	N	1115.544	558.276	9
7	830.347	415.677	E	1001.501	501.254	8
8	958.406	479.707	Q	872.458	436.733	7
9	1072.449	536.728	N	744.400	372.704	6
10	1171.517	586.262	V	630.357	315.682	5
11	1300.560	650.784	E	531.289	266.148	4
12	1413.644	707.326	L	402.246	201.627	3
13	1527.687	764.347	N	289.162	145.085	2
14			R	175.119	88.063	1

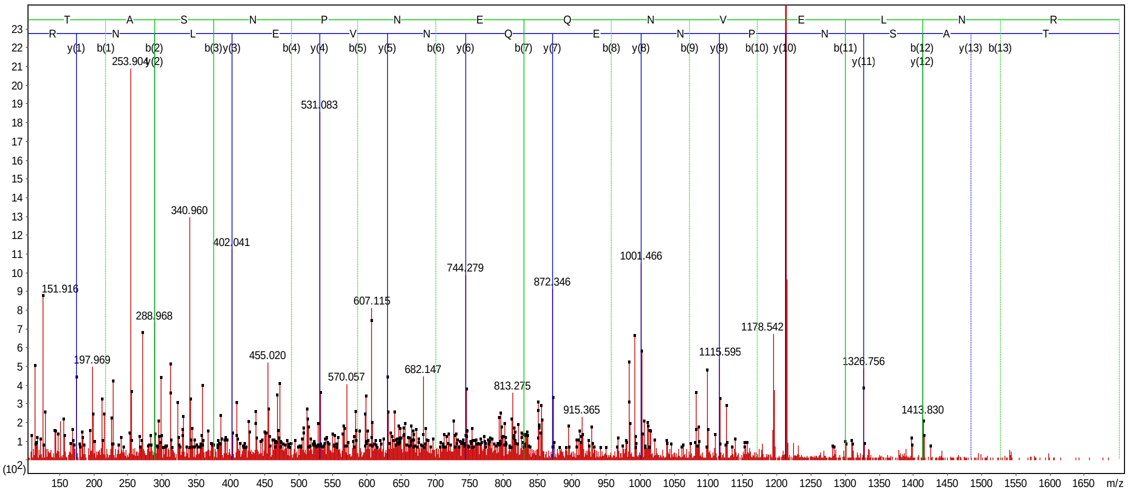

13 Accession No. BAJ87776

13.1 TLYSAYGSS$_p$GQWGFFDK

#	b	b++	Seq.	y	y++	#
1	134.111	67.559	T			17
2	247.195	124.101	L	1924.846	962.926	16
3	410.259	205.633	Y	1811.761	906.384	15
4	497.291	249.149	S	1648.698	824.853	14
5	568.328	284.668	A	1561.666	781.337	13
6	731.391	366.199	Y	1490.629	745.818	12
7	788.413	394.710	G	1327.566	664.286	11
8	875.445	438.226	S	1270.544	635.776	10
9	1042.443	521.725	S	1183.512	592.260	9
10	1099.465	550.236	G	1016.514	508.761	8
11	1227.523	614.265	Q	959.492	480.250	7
12	1413.602	707.305	W	831.434	416.221	6
13	1470.624	735.816	G	645.354	323.181	5
14	1617.692	809.350	F	588.333	294.670	4
15	1764.761	882.884	F	441.265	221.136	3
16	1879.788	940.397	D	294.196	147.602	2
17			K	179.169	90.088	1

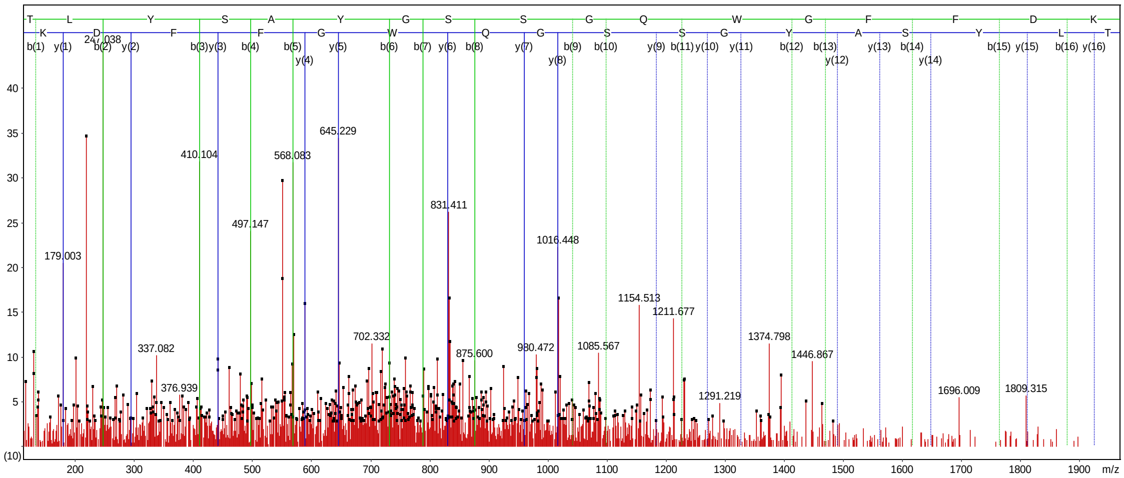

14 Accession No. P0C359

14.1 VYLGPET$_p$TR

#	b	b++	Seq.	y	y++	#
1	132.132	66.570	V			9
2	295.195	148.101	Y	1016.445	508.726	8
3	408.279	204.643	L	853.382	427.194	7
4	465.301	233.154	G	740.297	370.652	6
5	562.354	281.680	P	683.276	342.142	5
6	691.396	346.202	E	586.223	293.615	4
7	872.410	436.709	T	457.181	229.094	3
8	973.458	487.233	T	276.167	138.587	2
9			R	175.119	88.063	1

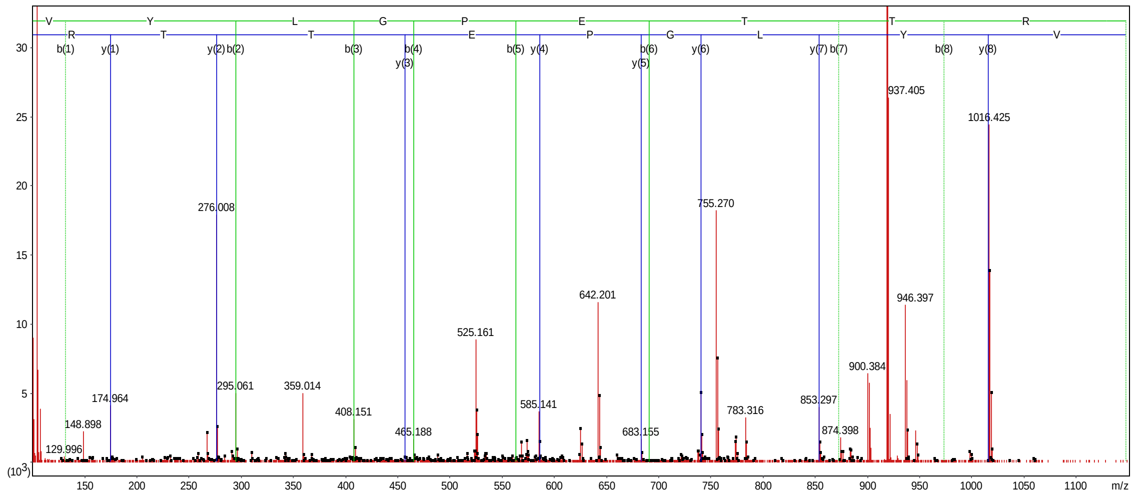

15 Accession No. XP_003564108

15.1 VFPS$_p$GEVQYLHPK

#	b	b++	Seq.	y	y++	#
1	132.132	66.570	V			13
2	279.201	140.104	F	1513.739	757.373	12
3	376.253	188.630	P	1366.670	683.839	11
4	543.252	272.129	S	1269.618	635.312	10
5	600.273	300.640	G	1102.619	551.813	9
6	729.316	365.161	E	1045.598	523.303	8
7	828.384	414.696	V	916.555	458.781	7
8	956.443	478.725	Q	817.487	409.247	6
9	1119.506	560.257	Y	689.428	345.218	5
10	1232.590	616.799	L	526.365	263.686	4
11	1369.649	685.328	H	413.281	207.144	3
12	1466.702	733.855	P	276.222	138.615	2
13			K	179.169	90.088	1

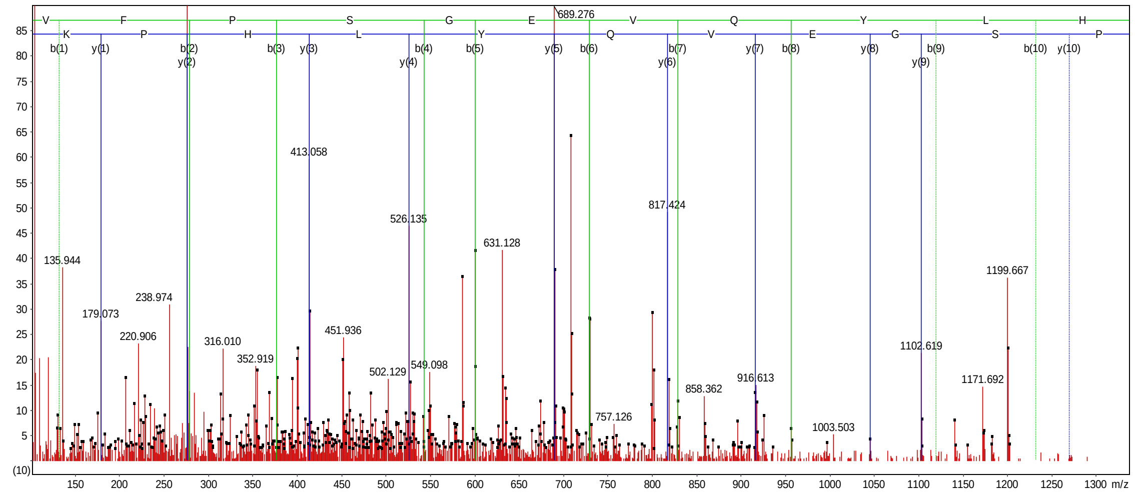

16 Accession No. XP_003563195

16.1 ESYWYNGTGS$_{p}$VVTVDQDPNTR

#	b	b++	Seq.	y	y++	#
1	166.126	83.566	E			21
2	253.158	127.082	S	2338.998	1170.002	20
3	416.221	208.614	Y	2251.966	1126.486	19
4	602.300	301.654	W	2088.902	1044.955	18
5	765.364	383.185	Y	1902.823	951.915	17
6	879.406	440.207	N	1739.760	870.383	16
7	936.428	468.718	G	1625.717	813.362	15
8	1037.476	519.241	T	1568.695	784.851	14
9	1094.497	547.752	G	1467.648	734.327	13
10	1261.495	631.251	S	1410.626	705.817	12
11	1360.564	680.786	V	1243.628	622.317	11
12	1459.632	730.320	V	1144.559	572.783	10
13	1560.680	780.844	T	1045.491	523.249	9
14	1659.748	830.378	V	944.443	472.725	8
15	1774.775	887.891	D	845.375	423.191	7
16	1902.834	951.921	Q	730.348	365.678	6
17	2017.861	1009.434	D	602.289	301.648	5
18	2114.914	1057.960	P	487.262	244.135	4
19	2228.957	1114.982	N	390.210	195.608	3
20	2330.004	1165.506	T	276.167	138.587	2
21			R	175.119	88.063	1

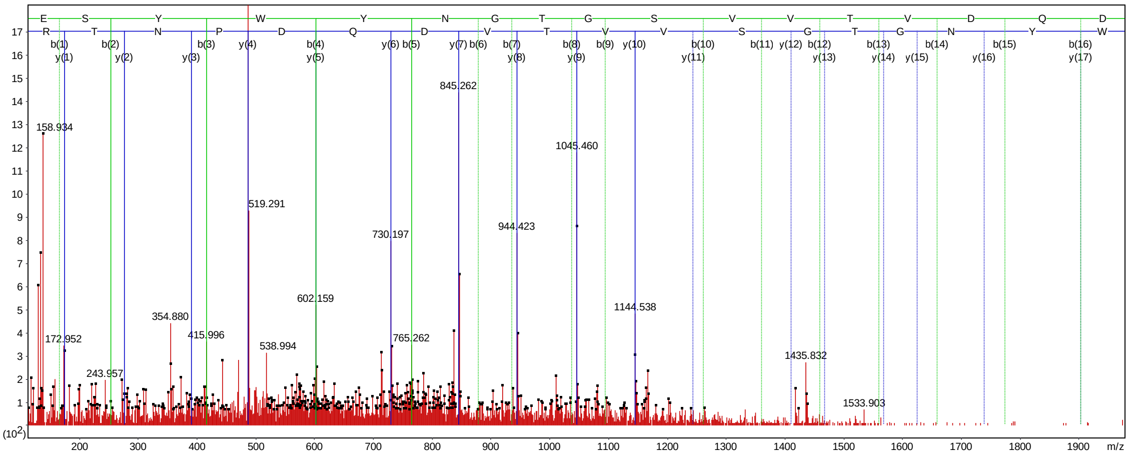

17 Accession No. ACG30530

17.1 VNYAGVS$_{p}$TNNYALDEVLEVK

#	b	b++	Seq.	y	y++	#
1	136.151	68.579	V			20
2	250.194	125.601	N	2215.071	1108.039	19
3	413.258	207.132	Y	2101.028	1051.018	18
4	484.295	242.651	A	1937.965	969.486	17
5	541.316	271.162	G	1866.928	933.968	16
6	640.385	320.696	V	1809.906	905.457	15
7	807.383	404.195	S	1710.838	855.923	14
8	908.431	454.719	T	1543.840	772.423	13
9	1022.474	511.740	N	1442.792	721.900	12
10	1136.517	568.762	N	1328.749	664.878	11
11	1299.580	650.294	Y	1214.706	607.857	10
12	1370.617	685.812	A	1051.643	526.325	9
13	1483.701	742.354	L	980.606	490.806	8
14	1598.728	799.868	D	867.521	434.264	7
15	1727.771	864.389	E	752.495	376.751	6
16	1826.839	913.923	V	623.452	312.230	5
17	1939.923	970.465	L	524.384	262.695	4
18	2068.966	1034.986	E	411.299	206.153	3
19	2168.034	1084.521	V	282.257	141.632	2
20			K	183.188	92.098	1

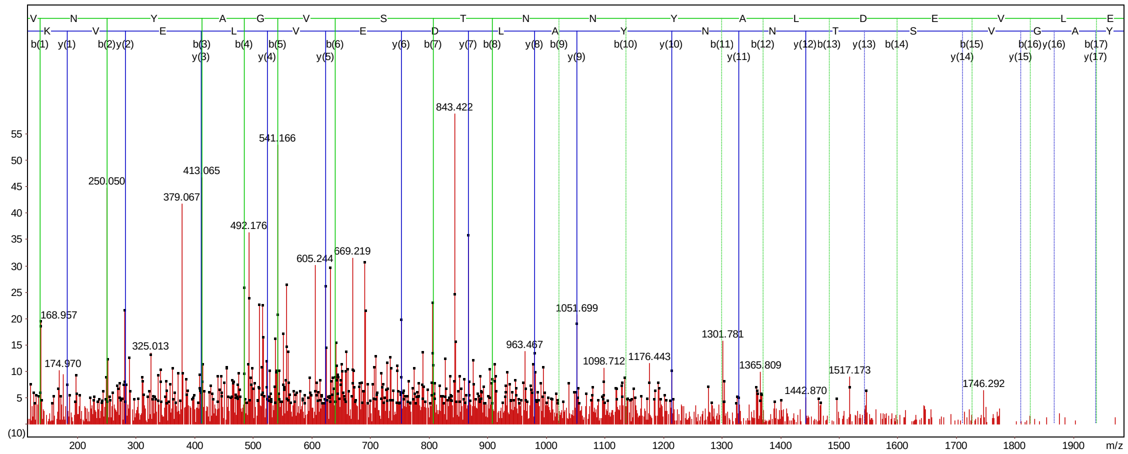

18 Accession No. EMT19581

18.1 GPQLPPT$_p$PGPR

#	b	b^{++}	Seq.	y	y^{++}	#
1	90.085	45.546	G			11
2	187.138	94.073	P	1139.561	570.284	10
3	315.196	158.102	Q	1042.508	521.758	9
4	428.281	214.644	L	914.450	457.728	8
5	525.333	263.170	P	801.365	401.186	7
6	622.386	311.697	P	704.313	352.660	6
7	803.400	402.204	T	607.260	304.134	5
8	900.453	450.730	P	426.246	213.627	4
9	957.474	479.241	G	329.193	165.100	3
10	1054.527	527.767	P	272.172	136.589	2
11			R	175.119	88.063	1

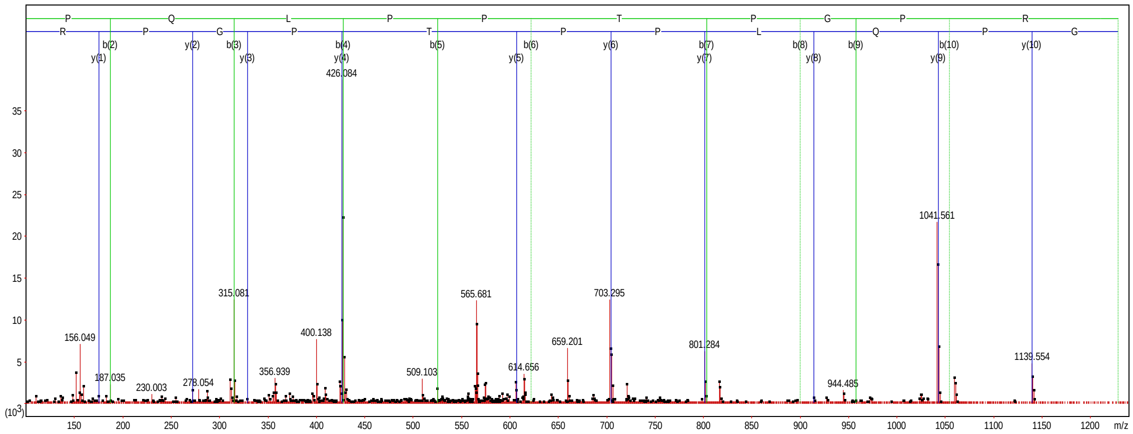

19 Accession No. A1EA21

19.1 VQLY$_p$EMNF

#	b	b^{++}	Seq.	y	y^{++}	#
1	132.132	66.570	V			8
2	260.191	130.599	Q	1024.385	512.696	7
3	373.275	187.141	L	896.326	448.667	6
4	616.304	308.656	Y	783.242	392.125	5
5	745.347	373.177	E	540.212	270.610	4
6	876.387	438.697	M	411.170	206.088	3
7	990.430	495.719	N	280.129	140.568	2
8			F	166.086	83.547	1

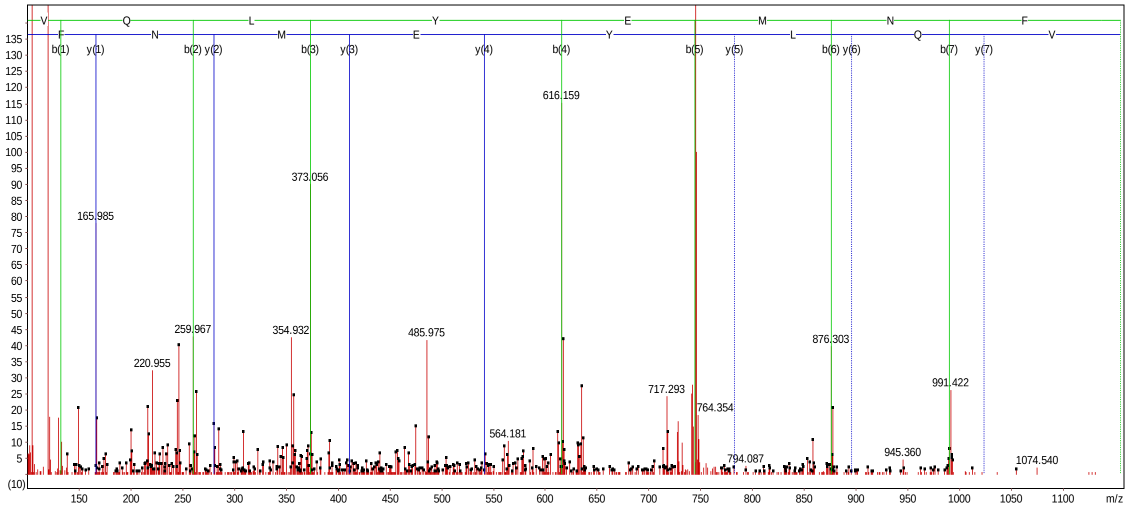

20 Accession No. BAK01659

20.1 AQPGSTAS$_p$DVNIEEVR

#	b	b++	Seq.	y	y++	#
1	108.120	54.564	A			16
2	236.179	118.593	Q	1681.743	841.375	15
3	333.231	167.119	P	1553.684	777.346	14
4	390.253	195.630	G	1456.632	728.819	13
5	477.285	239.146	S	1399.610	700.309	12
6	578.333	289.670	T	1312.578	656.793	11
7	649.370	325.188	A	1211.530	606.269	10
8	816.368	408.688	S	1140.493	570.750	9
9	931.395	466.201	D	973.495	487.251	8
10	1030.463	515.735	V	858.468	429.738	7
11	1144.506	572.757	N	759.400	380.203	6
12	1257.590	629.299	I	645.357	323.182	5
13	1386.633	693.820	E	532.273	266.640	4
14	1515.676	758.341	E	403.230	202.119	3
15	1614.744	807.876	V	274.187	137.597	2
16			R	175.119	88.063	1

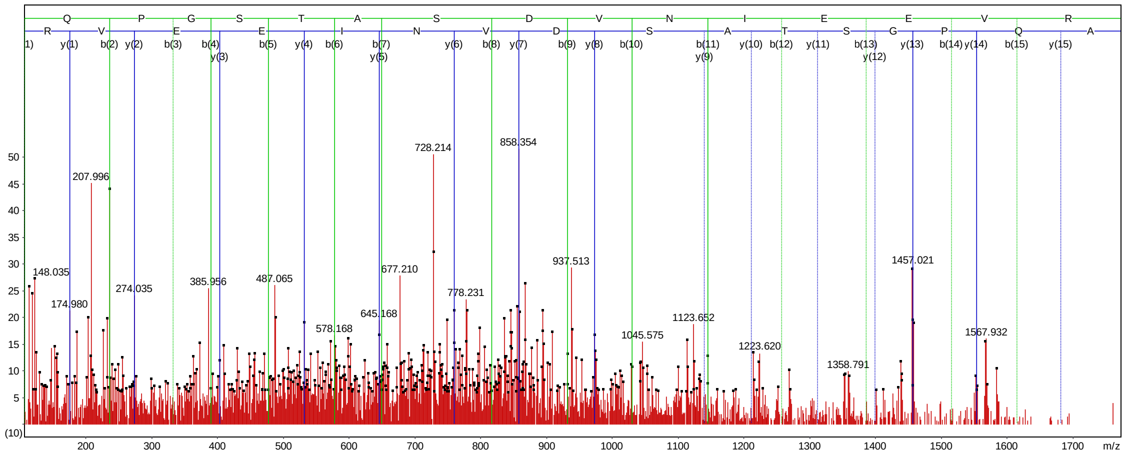

21 Accession No. KEH27588

21.1 LVYT$_p$NDAGEVVK

#1	b+	b2+	Seq.	y+	y2+	#2
1	150.16702	75.58715	L-Dimethyl...			12
2	249.23544	125.12136	V	1310.64214	655.82471	11
3	412.29876	206.65302	Y	1211.57372	606.29050	10
4	593.31277	297.16002	T-Phospho	1048.51040	524.75884	9
5	707.35570	354.18149	N	867.49639	434.25183	8
6	822.38265	411.69496	D	753.45346	377.23037	7
7	893.41977	447.21352	A	638.42651	319.71689	6
8	950.44124	475.72426	G	567.38939	284.19833	5
9	1079.48384	540.24556	E	510.36792	255.68760	4
10	1178.55226	589.77977	V	381.32532	191.16630	3
11	1277.62068	639.31398	V	282.25690	141.63209	2
12			K-Dimethyl...	183.18848	92.09788	1

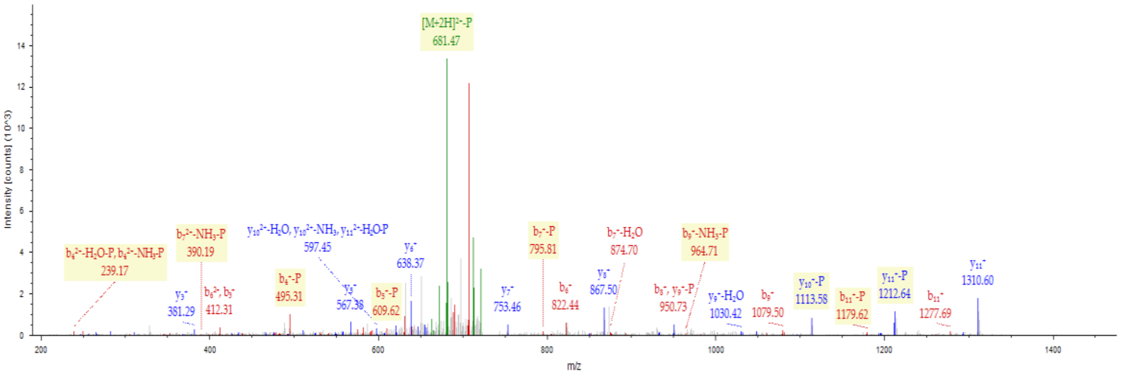

22 Accession No. EEH57944

22.1 LGIAPIIMS$_p$AGELESGNAGEPAK

#	b	b⁺⁺	Seq.	y	y⁺⁺	#
1	146.148	73.578	L			23
2	203.169	102.088	G	2240.082	1120.545	22
3	316.253	158.630	I	2183.060	1092.034	21
4	387.290	194.149	A	2069.976	1035.492	20
5	484.343	242.675	P	1998.939	999.973	19
6	597.427	299.217	I	1901.886	951.447	18
7	710.511	355.759	I	1788.802	894.905	17
8	857.547	429.277	M	1675.718	838.363	16
9	1024.545	512.776	S	1528.683	764.845	15
10	1095.582	548.295	A	1361.685	681.346	14
11	1152.604	576.805	G	1290.647	645.827	13
12	1281.646	641.327	E	1233.626	617.317	12
13	1394.730	697.869	L	1104.583	552.795	11
14	1523.773	762.390	E	991.499	496.253	10
15	1610.805	805.906	S	862.457	431.732	9
16	1667.826	834.417	G	775.425	388.216	8
17	1781.869	891.438	N	718.403	359.705	7
18	1852.906	926.957	A	604.360	302.684	6
19	1909.928	955.468	G	533.323	267.165	5
20	2038.970	1019.989	E	476.302	238.654	4
21	2136.023	1068.515	P	347.259	174.133	3
22	2207.060	1104.034	A	250.206	125.607	2
23			K	179.169	90.088	1

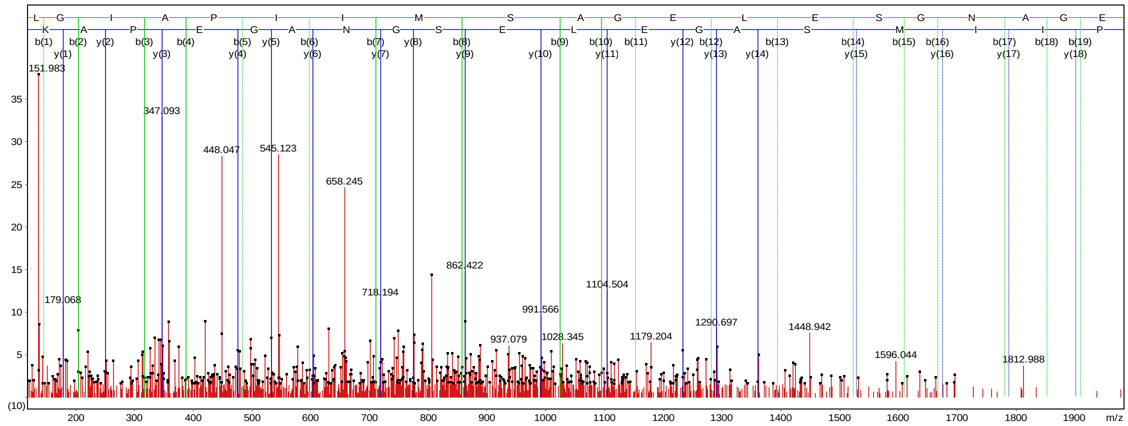

23 Accession No. AAB70542

23.1 GLVPSAGS$_p$NNESWCQGLDGLASR

#	b	b⁺⁺	Seq.	y	y⁺⁺	#
1	94.104	47.556	G			23
2	207.188	104.098	L	2398.049	1199.528	22
3	306.257	153.632	V	2284.965	1142.986	21
4	403.310	202.158	P	2185.897	1093.452	20
5	490.342	245.674	S	2088.844	1044.926	19
6	561.379	281.193	A	2001.812	1001.410	18
7	618.400	309.704	G	1930.775	965.891	17
8	785.399	393.203	S	1873.753	937.380	16
9	899.442	450.224	N	1706.755	853.881	15
10	1013.484	507.246	N	1592.712	796.860	14
11	1142.527	571.767	E	1478.669	739.838	13
12	1229.559	615.283	S	1349.627	675.317	12
13	1415.638	708.323	W	1262.595	631.801	11
14	1575.669	788.338	C	1076.515	538.761	10
15	1703.728	852.367	Q	916.485	458.746	9
16	1760.749	880.878	G	788.426	394.717	8
17	1873.833	937.420	L	731.405	366.206	7
18	1988.860	994.934	D	618.321	309.664	6
19	2045.882	1023.444	G	503.294	252.150	5
20	2158.966	1079.986	L	446.272	223.640	4
21	2230.003	1115.505	A	333.188	167.098	3
22	2317.035	1159.021	S	262.151	131.579	2
23			R	175.119	88.063	1

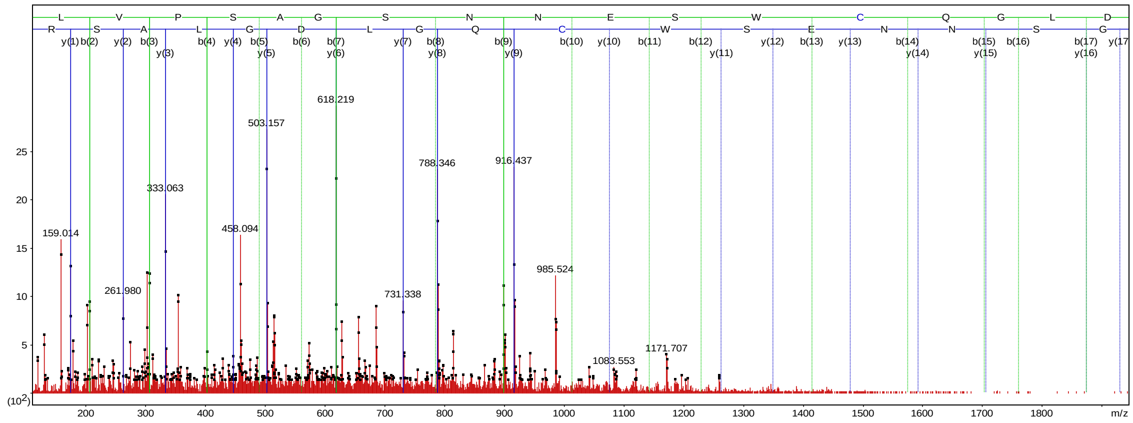

24 Accession No. KEH17711

24.1 GEIIAS$_p$ESR

#1	b+	b2+	Seq.	y+	y2+	#2
1	94.10442	47.55585	G-Dimethyl…			9
2	223.14702	112.07715	E	984.43981	492.72354	8
3	336.23109	168.61918	I	855.39721	428.20224	7
4	449.31516	225.16122	I	742.31314	371.66021	6
5	520.35228	260.67978	A	629.22907	315.11817	5
6	687.35064	344.17896	S-Phospho	558.19195	279.59961	4
7	816.39324	408.70026	E	391.19359	196.10043	3
8	903.42527	452.21627	S	262.15099	131.57913	2
9			R	175.11896	88.06312	1

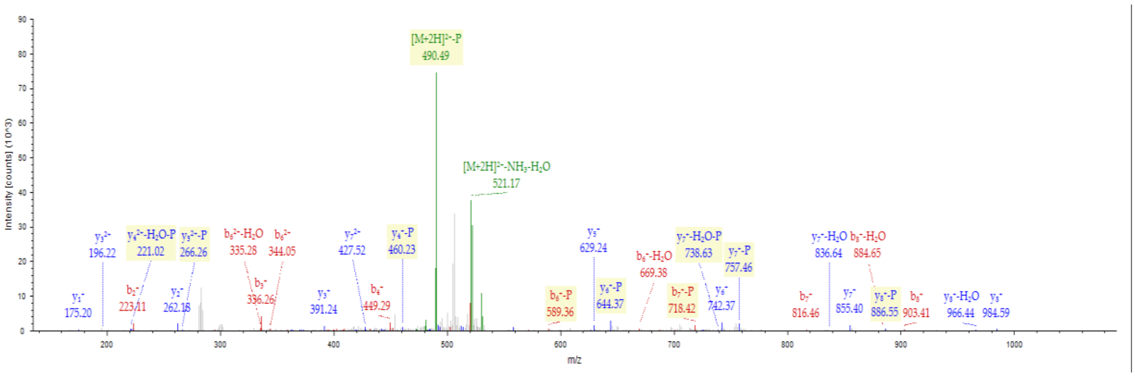

25 Accession No. ABH02573

25.1 AITLEEENKS$_p$KK

#	b	b++	Seq.	y	y++	#
1	100.076	50.541	A			12
2	213.160	107.084	I	1482.782	741.894	11
3	314.207	157.607	T	1369.697	685.352	10
4	427.291	214.149	L	1268.650	634.829	9
5	556.334	278.671	E	1155.566	578.286	8
6	685.377	343.192	E	1026.523	513.765	7
7	814.419	407.713	E	897.481	449.244	6
8	928.462	464.735	N	768.438	384.723	5
9	1084.588	542.798	K	654.395	327.701	4
10	1251.587	626.297	S	498.269	249.638	3
11	1407.713	704.360	K	331.270	166.139	2
12			K	175.144	88.076	1

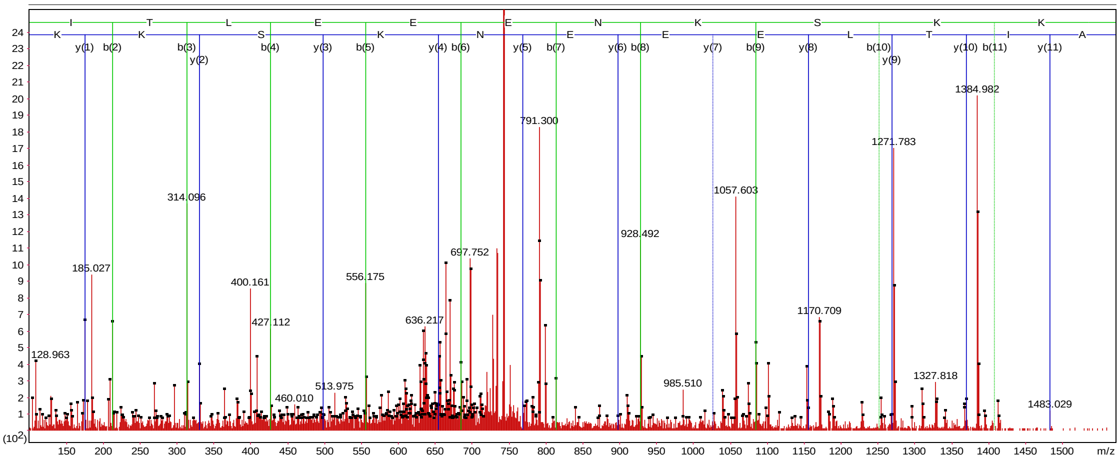

26 Accession No. CAB89989

26.1 GRNTGGQPINAT$_{p}$CEVQQLLGNNR

#	b	b⁺⁺	Seq.	y	y⁺⁺	#
4	461.277	231.142	T	2250.033	1125.520	20
5	518.298	259.653	G	2148.986	1074.996	19
6	575.320	288.164	G	2091.964	1046.486	18
7	703.378	352.193	Q	2034.943	1017.975	17
8	800.431	400.719	P	1906.884	953.946	16
9	913.515	457.261	I	1809.831	905.419	15
10	1027.558	514.283	N	1696.747	848.877	14
11	1098.595	549.801	A	1582.704	791.856	13
12	1279.609	640.308	T	1511.667	756.337	12
13	1439.640	720.324	C	1330.653	665.830	11
14	1568.682	784.845	E	1170.623	585.815	10
15	1667.751	834.379	V	1041.580	521.294	9
16	1795.809	898.408	Q	942.512	471.759	8
17	1923.868	962.438	Q	814.453	407.730	7
18	2036.952	1018.980	L	686.394	343.701	6
19	2150.036	1075.522	L	573.310	287.159	5
20	2207.058	1104.032	G	460.226	230.617	4
21	2321.101	1161.054	N	403.205	202.106	3
22	2435.144	1218.075	N	289.162	145.085	2
23			R	175.119	88.063	1

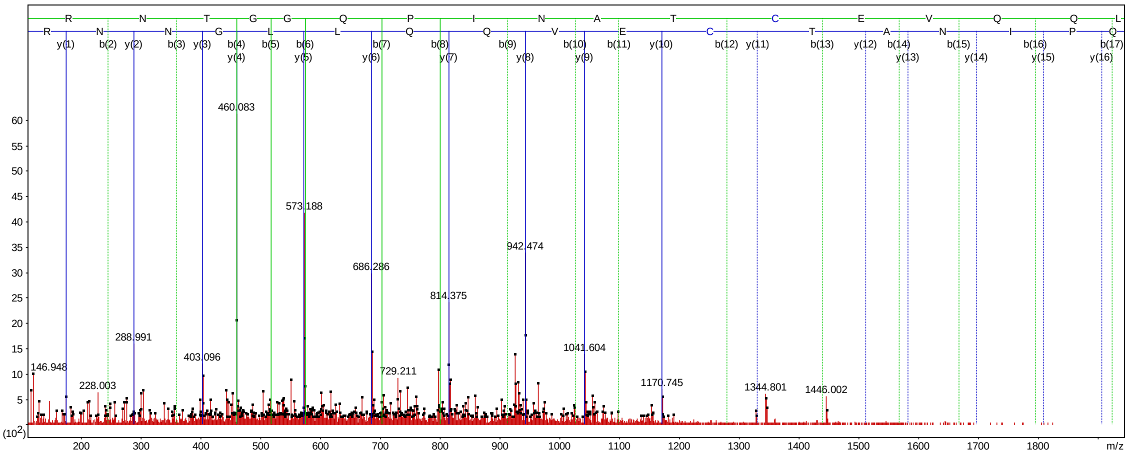

27 Accession No. AAK72724

27.1 DT$_{p}$VRQQINVTCEVQQLLGNNR

#	b	b⁺⁺	Seq.	y	y⁺⁺	#
1	152.110	76.559	D			21
2	333.124	167.066	T	2391.149	1196.078	20
3	432.192	216.600	V	2210.135	1105.571	19
4	529.245	265.126	P	2111.066	1056.037	18
5	657.304	329.155	Q	2014.013	1007.510	17
6	785.362	393.185	Q	1885.955	943.481	16
7	898.446	449.727	I	1757.896	879.452	15
8	1012.489	506.748	N	1644.812	822.910	14
9	1111.558	556.282	V	1530.769	765.888	13
10	1212.605	606.806	T	1431.701	716.354	12
11	1372.636	686.822	C	1330.653	665.830	11
12	1501.679	751.343	E	1170.623	585.815	10
13	1600.747	800.877	V	1041.580	521.294	9
14	1728.806	864.906	Q	942.512	471.759	8
15	1856.864	928.936	Q	814.453	407.730	7
16	1969.948	985.478	L	686.394	343.701	6
17	2083.032	1042.020	L	573.310	287.159	5
18	2140.054	1070.530	G	460.226	230.617	4
19	2254.097	1127.552	N	403.205	202.106	3
20	2368.140	1184.573	N	289.162	145.085	2
21			R	175.119	88.063	1

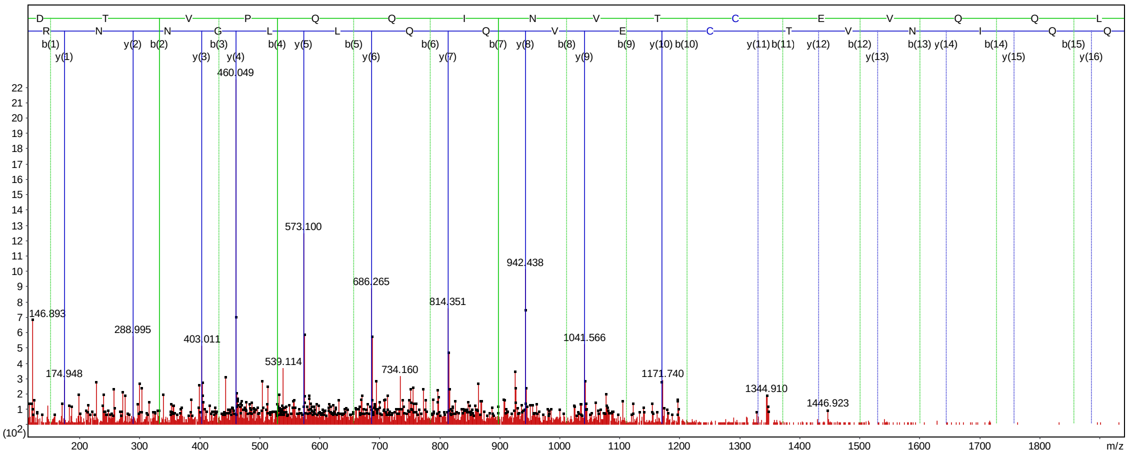

28 Accession No. ACF08646

28.1 AEGT$_p$KELVEAK

#	b	b++	Seq.	y	y++	#
1	108.120	54.564	A			11
2	237.163	119.085	E	1255.712	628.360	10
3	294.184	147.596	G	1126.669	563.838	9
4	475.198	238.103	T	1069.648	535.328	8
5	639.369	320.188	K	888.634	444.821	7
6	768.411	384.709	E	724.463	362.735	6
7	881.495	441.251	L	595.421	298.214	5
8	980.564	490.786	V	482.337	241.672	4
9	1109.606	555.307	E	383.268	192.138	3
10	1180.644	590.825	A	254.226	127.616	2
11			K	183.188	92.098	1

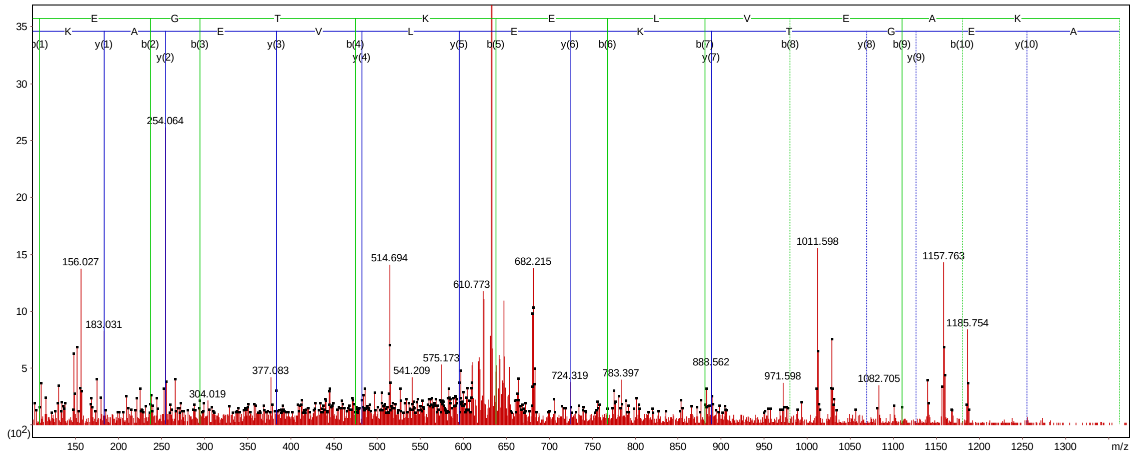

29 Accession No. EAY89475

29.1 AELGGVKDAS$_p$EEVR

#	b	b++	Seq.	y	y++	#
1	108.120	54.564	A			14
2	237.163	119.085	E	1504.744	752.875	13
3	350.247	175.627	L	1375.701	688.354	12
4	407.268	204.138	G	1262.617	631.812	11
5	464.290	232.648	G	1205.595	603.301	10
6	563.358	282.183	V	1148.574	574.791	9
7	727.529	364.268	K	1049.506	525.256	8
8	842.556	421.781	D	885.335	443.171	7
9	913.593	457.300	A	770.308	385.658	6
10	1080.591	540.799	S	699.271	350.139	5
11	1209.634	605.320	E	532.273	266.640	4
12	1338.676	669.842	E	403.230	202.119	3
13	1437.745	719.376	V	274.187	137.597	2
14			R	175.119	88.063	1

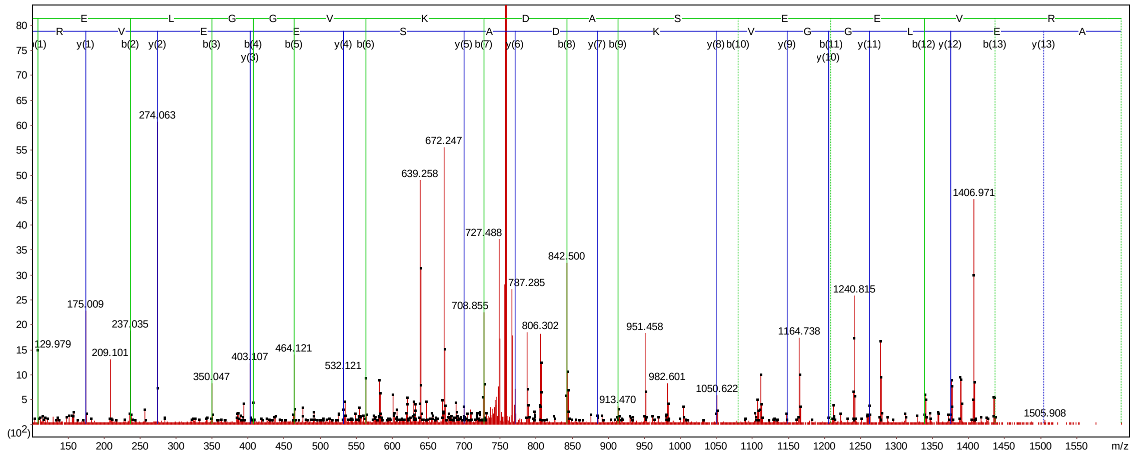

30 Accession No. AAQ14552

30.1 LVS$_p$DDEDEQSK

#	b	b++	Seq.	y	y++	#
1	142.123	71.565	L			11
2	241.191	121.099	V	1259.467	630.237	10
3	408.189	204.598	S	1160.399	580.703	9
4	523.216	262.112	D	993.401	497.204	8
5	638.243	319.625	D	878.374	439.691	7
6	767.286	384.147	E	763.347	382.177	6
7	882.313	441.660	D	634.304	317.656	5
8	1011.355	506.181	E	519.277	260.142	4
9	1139.414	570.211	Q	390.235	195.621	3
10	1226.446	613.727	S	262.176	131.592	2
11			K	175.144	88.076	1

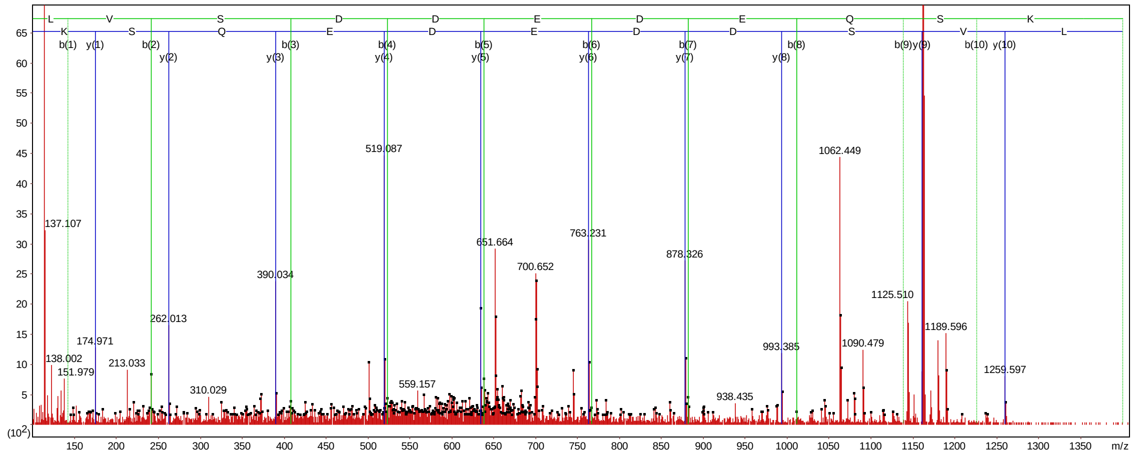

31 Accession No. Q8H1Y0

31.1 YHGHS$_p$MSDPGSTYR

#	b	b++	Seq.	y	y++	#
1	200.146	100.577	Y			14
2	337.205	169.106	H	1511.573	756.290	13
3	394.227	197.617	G	1374.514	687.761	12
4	531.286	266.146	H	1317.493	659.250	11
5	698.284	349.646	S	1180.434	590.721	10
6	829.324	415.166	M	1013.436	507.221	9
7	916.356	458.682	S	882.395	441.701	8
8	1031.383	516.195	D	795.363	398.185	7
9	1128.436	564.722	P	680.336	340.672	6
10	1185.458	593.232	G	583.283	292.145	5
11	1272.490	636.748	S	526.262	263.635	4
12	1373.537	687.272	T	439.230	220.119	3
13	1536.601	768.804	Y	338.182	169.595	2
14			R	175.119	88.063	1

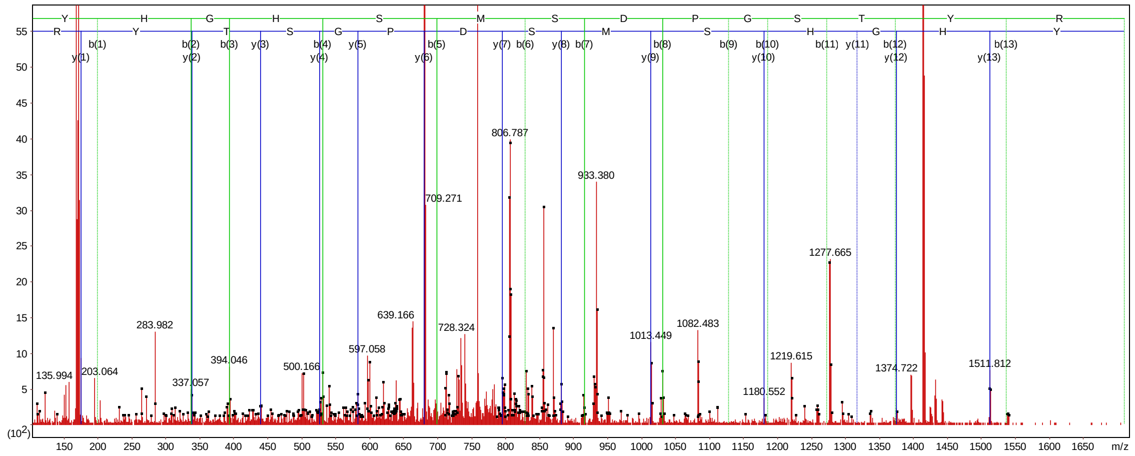

32 Accession No. BAK01943

32.1 QVAHAPQELNS$_p$PR

#	b	b^{++}	Seq.	y	y^{++}	#
1	161.122	81.065	Q			13
2	260.191	130.599	V	1398.653	699.830	12
3	331.228	166.118	A	1299.584	650.296	11
4	468.287	234.647	H	1228.547	614.777	10
5	539.324	270.166	A	1091.488	546.248	9
6	636.377	318.692	P	1020.451	510.729	8
7	764.435	382.721	Q	923.398	462.203	7
8	893.478	447.243	E	795.340	398.173	6
9	1006.562	503.785	L	666.297	333.652	5
10	1120.605	560.806	N	553.213	277.110	4
11	1287.603	644.305	S	439.170	220.089	3
12	1384.656	692.832	P	272.172	136.589	2
13			R	175.119	88.063	1

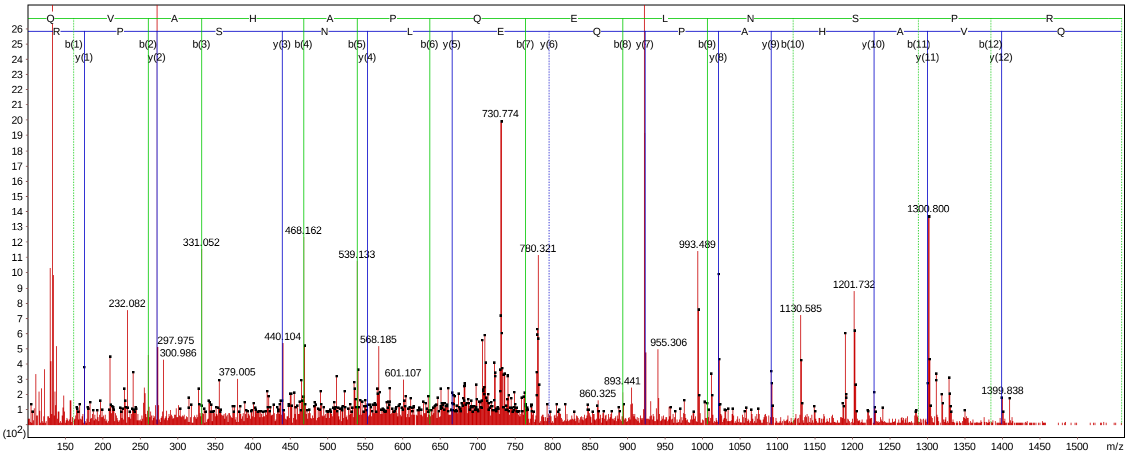

33 Accession No. BAJ06107

33.1 GFVPFVPGS$_p$PVER

#	b	b^{++}	Seq.	y	y^{++}	#
1	90.085	45.546	G			13
2	237.154	119.080	F	1410.682	705.845	12
3	336.222	168.615	V	1263.613	632.310	11
4	433.275	217.141	P	1164.545	582.776	10
5	580.343	290.675	F	1067.492	534.250	9
6	679.412	340.209	V	920.424	460.715	8
7	776.464	388.736	P	821.355	411.181	7
8	833.486	417.247	G	724.303	362.655	6
9	1000.484	500.746	S	667.281	334.144	5
10	1097.537	549.272	P	500.283	250.645	4
11	1196.605	598.806	V	403.230	202.119	3
12	1325.648	663.328	E	304.162	152.584	2
13			R	175.119	88.063	1

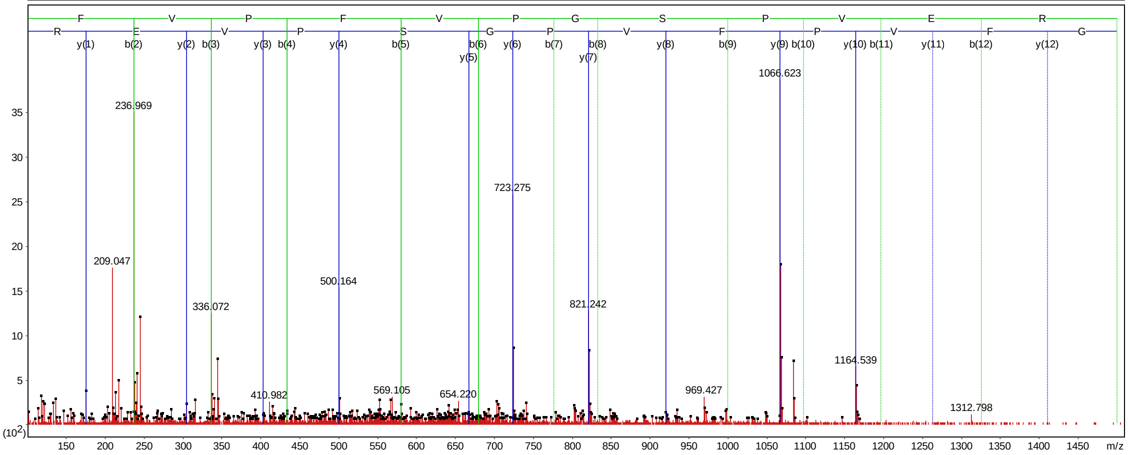

34 Accession No. EMT05628

34.1 AAAVAALSSVLTAEQSGS$_p$SDNLR

#	b	b⁺⁺	Seq.	y	y⁺⁺	#
1	104.101	52.554	A			23
2	175.138	88.073	A	2227.060	1114.034	22
3	246.175	123.591	A	2156.023	1078.515	21
4	345.243	173.125	V	2084.986	1042.997	20
5	416.281	208.644	A	1985.918	993.462	19
6	487.318	244.162	A	1914.880	957.944	18
7	600.402	300.705	L	1843.843	922.425	17
8	687.434	344.221	S	1730.759	865.883	16
9	774.466	387.737	S	1643.727	822.367	15
10	873.534	437.271	V	1556.695	778.851	14
11	986.618	493.813	L	1457.627	729.317	13
12	1087.666	544.337	T	1344.543	672.775	12
13	1158.703	579.855	A	1243.495	622.251	11
14	1287.746	644.376	E	1172.458	586.733	10
15	1415.804	708.406	Q	1043.415	522.211	9
16	1502.836	751.922	S	915.357	458.182	8
17	1559.858	780.432	G	828.325	414.666	7
18	1726.856	863.932	S	771.303	386.155	6
19	1813.888	907.448	S	604.305	302.656	5
20	1928.915	964.961	D	517.273	259.140	4
21	2042.958	1021.983	N	402.246	201.627	3
22	2156.042	1078.525	L	288.203	144.605	2
23			R	175.119	88.063	1

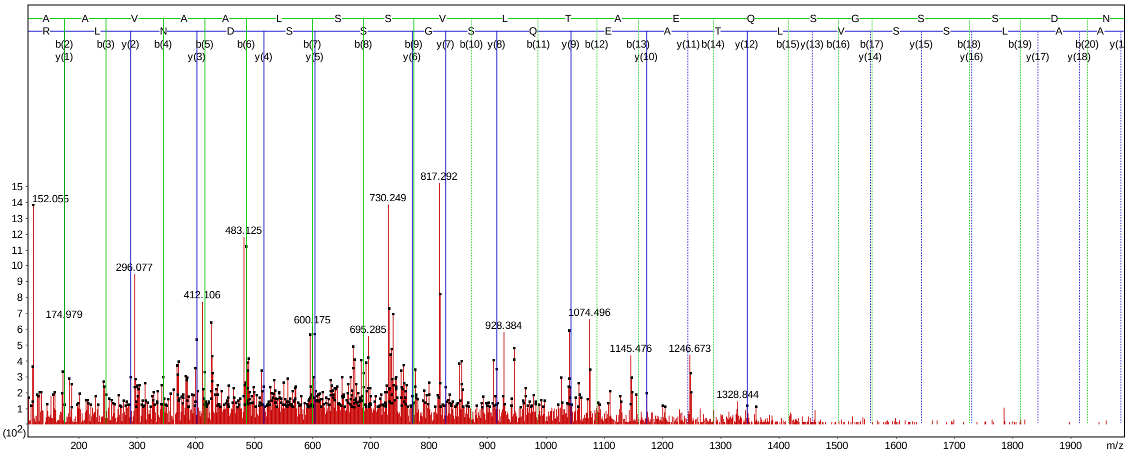

35 Accession No. BAK02001

35.1 ASSDDTSTSAAS$_p$GDELVDDLK

#	b	b⁺⁺	Seq.	y	y⁺⁺	#
1	100.076	50.541	A			21
2	187.108	94.057	S	2120.875	1060.941	20
3	274.140	137.574	S	2033.843	1017.425	19
4	389.167	195.087	D	1946.811	973.909	18
5	504.194	252.600	D	1831.784	916.396	17
6	605.241	303.124	T	1716.758	858.882	16
7	692.273	346.640	S	1615.710	808.359	15
8	793.321	397.164	T	1528.678	764.843	14
9	880.353	440.680	S	1427.630	714.319	13
10	951.390	476.199	A	1340.598	670.803	12
11	1022.427	511.717	A	1269.561	635.284	11
12	1189.426	595.216	S	1198.524	599.766	10
13	1246.447	623.727	G	1031.526	516.266	9
14	1361.474	681.241	D	974.504	487.756	8
15	1490.517	745.762	E	859.477	430.242	7
16	1603.601	802.304	L	730.435	365.721	6
17	1702.669	851.838	V	617.350	309.179	5
18	1817.696	909.352	D	518.282	259.645	4
19	1932.723	966.865	D	403.255	202.131	3
20	2045.807	1023.407	L	288.228	144.618	2
21			K	175.144	88.076	1

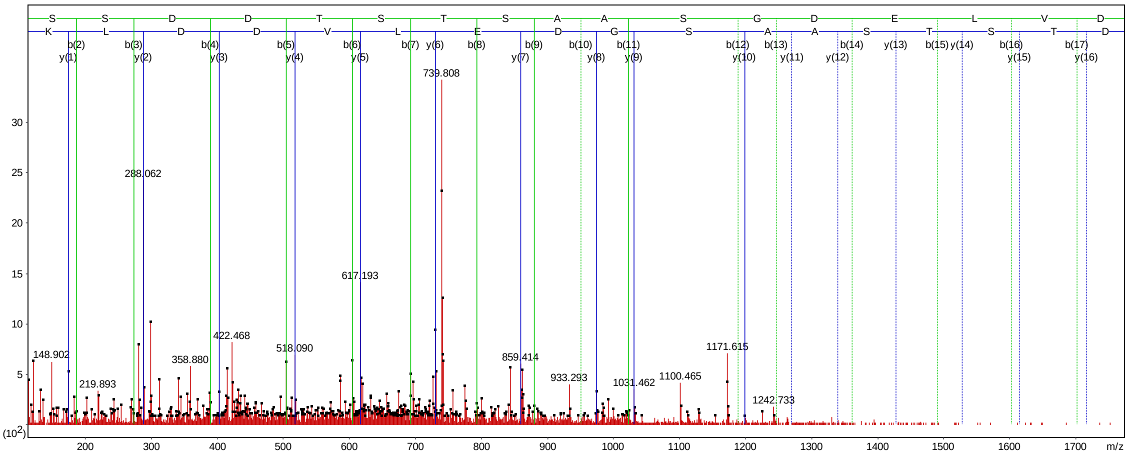

36 Accession No. XP_004965129

36.1 QSHS$_p$DGSLDTMAR

#	b	b⁺⁺	Seq.	y	y⁺⁺	#
1	165.142	83.074	Q			13
2	252.174	126.590	S	1356.525	678.766	12
3	389.232	195.120	H	1269.493	635.250	11
4	556.231	278.619	S	1132.434	566.721	10
5	671.258	336.133	D	965.436	483.221	9
6	728.279	364.643	G	850.409	425.708	8
7	815.311	408.159	S	793.387	397.197	7
8	928.395	464.701	L	706.355	353.681	6
9	1043.422	522.215	D	593.271	297.139	5
10	1144.470	572.739	T	478.244	239.626	4
11	1275.510	638.259	M	377.197	189.102	3
12	1346.548	673.777	A	246.156	123.582	2
13			R	175.119	88.063	1

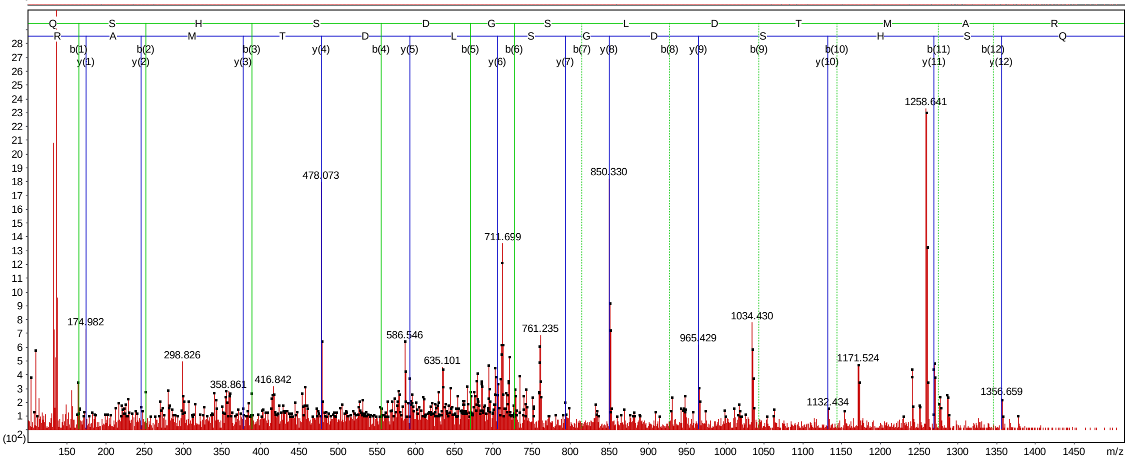

37 Accession No. EMS47290

37.1 VAEQLS$_p$DDEGEDQSK

#	b	b⁺⁺	Seq.	y	y⁺⁺	#
1	128.107	64.557	V			15
2	199.144	100.076	A	1560.666	780.837	14
3	328.187	164.597	E	1489.629	745.318	13
4	456.245	228.626	Q	1360.586	680.797	12
5	569.329	285.168	L	1232.528	616.767	11
6	638.351	319.679	S	1119.444	560.225	10
7	753.378	377.193	D	1050.422	525.715	9
8	868.405	434.706	D	935.395	468.201	8
9	997.447	499.227	E	820.368	410.688	7
10	1054.469	527.738	G	691.326	346.166	6
11	1183.511	592.259	E	634.304	317.656	5
12	1298.538	649.773	D	505.262	253.134	4
13	1426.597	713.802	Q	390.235	195.621	3
14	1513.629	757.318	S	262.176	131.592	2
15			K	175.144	88.076	1

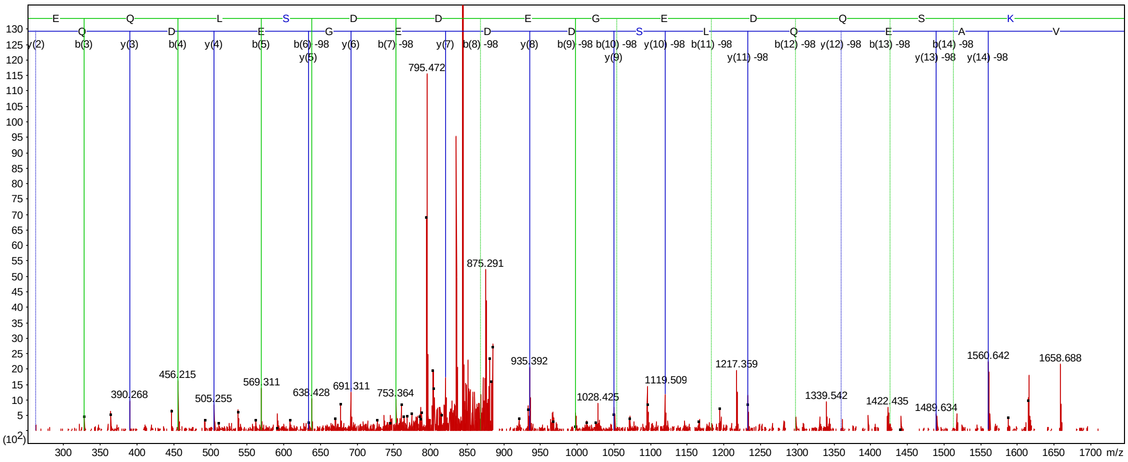

38 Accession No. EMT08603

38.1 SIS$_{p}$ADGLNSLR

#	b	b^{++}	Seq.	y	y^{++}	#
1	116.071	58.539	S			11
2	229.155	115.081	I	1125.530	563.269	10
3	396.153	198.580	S	1012.446	506.727	9
4	467.190	234.099	A	845.448	423.227	8
5	582.217	291.612	D	774.410	387.709	7
6	639.239	320.123	G	659.383	330.195	6
7	752.323	376.665	L	602.362	301.685	5
8	866.366	433.686	N	489.278	245.143	4
9	953.398	477.202	S	375.235	188.121	3
10	1066.482	533.744	L	288.203	144.605	2
11			R	175.119	88.063	1

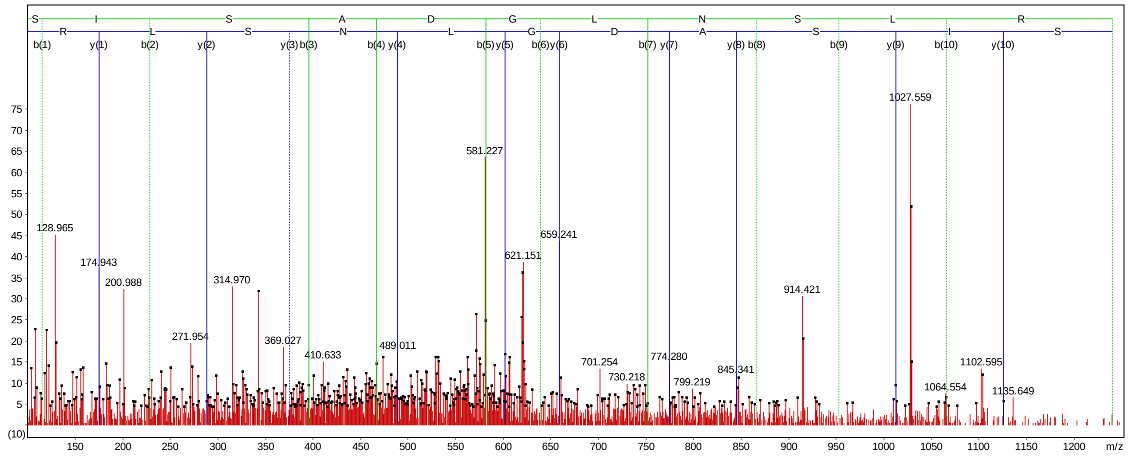

39 Accession No. AAS00828

39.1 KLLPGS$_{p}$VDG

#	b	b^{++}	Seq.	y	y^{++}	#
1	193.215	97.111	K			9
2	306.299	153.653	L	837.375	419.191	8
3	419.383	210.195	L	724.291	362.649	7
4	516.436	258.722	P	611.207	306.107	6
5	573.457	287.232	G	514.154	257.581	5
6	740.456	370.732	S	457.133	229.070	4
7	839.524	420.266	V	290.135	145.571	3
8	954.551	477.779	D	191.066	96.037	2
9			G	76.039	38.523	1

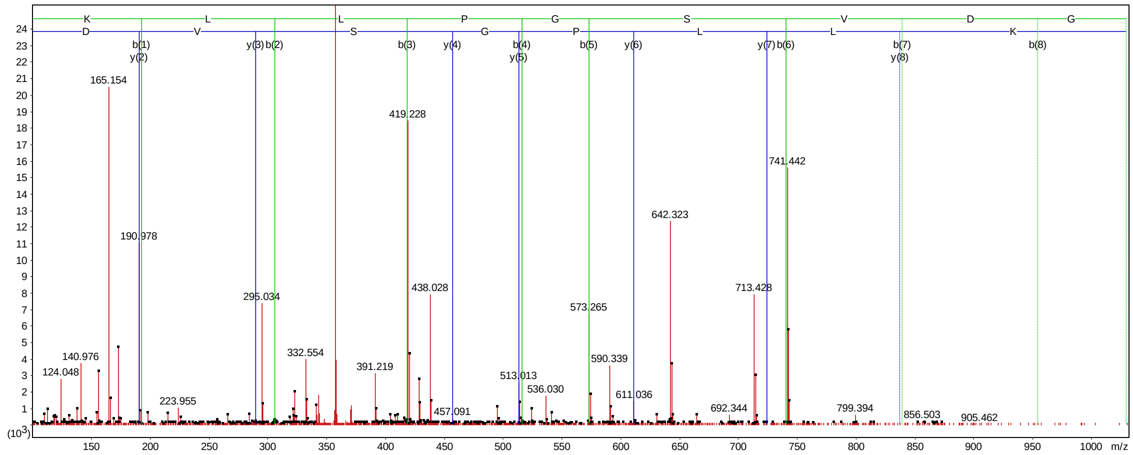

40 Accession No. EMT29522

40.1 GQDPQGMS$_p$PGPGGR

#	b	b⁺⁺	Seq.	y	y⁺⁺	#
1	90.085	45.546	G			14
2	218.144	109.576	Q	1363.546	682.277	13
3	333.171	167.089	D	1235.487	618.247	12
4	430.223	215.615	P	1120.461	560.734	11
5	558.282	279.645	Q	1023.408	512.208	10
6	615.303	308.155	G	895.349	448.178	9
7	746.344	373.676	M	838.328	419.667	8
8	913.342	457.175	S	707.287	354.147	7
9	1010.395	505.701	P	540.289	270.648	6
10	1067.417	534.212	G	443.236	222.122	5
11	1164.469	582.738	P	386.215	193.611	4
12	1221.491	611.249	G	289.162	145.085	3
13	1278.512	639.760	G	232.140	116.574	2
14			R	175.119	88.063	1

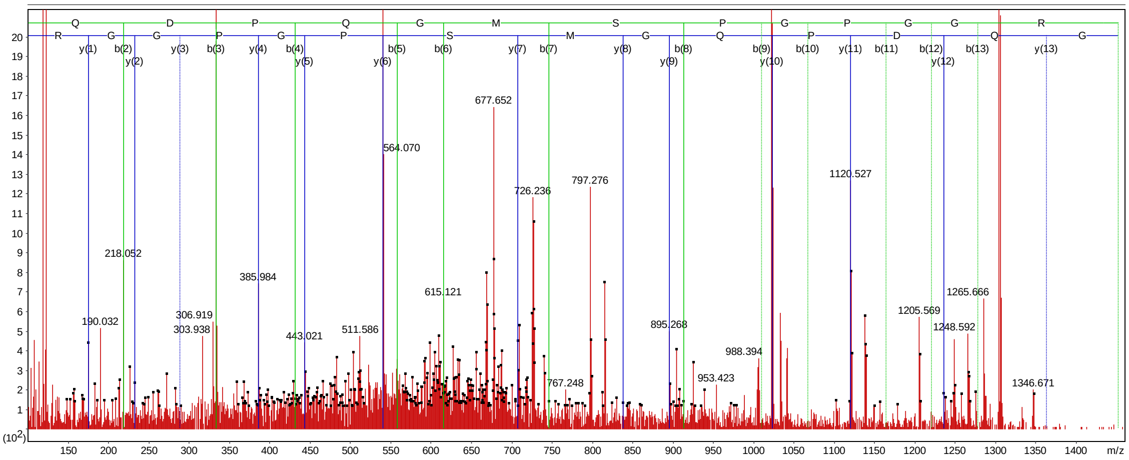

41 Accession No. EEC75374

41.1 SDSTASGDS$_p$PKER

#	b	b⁺⁺	Seq.	y	y⁺⁺	#
1	120.096	60.551	S			13
2	235.123	118.065	D	1361.588	681.298	12
3	322.155	161.581	S	1246.561	623.784	11
4	423.202	212.105	T	1159.529	580.268	10
5	494.239	247.623	A	1058.482	529.744	9
6	581.272	291.139	S	987.444	494.226	8
7	638.293	319.650	G	900.412	450.710	7
8	753.320	377.164	D	843.391	422.199	6
9	920.318	460.663	S	728.364	364.686	5
10	1017.371	509.189	P	561.366	281.186	4
11	1177.522	589.265	K	464.313	232.660	3
12	1306.565	653.786	E	304.162	152.584	2
13			R	175.119	88.063	1

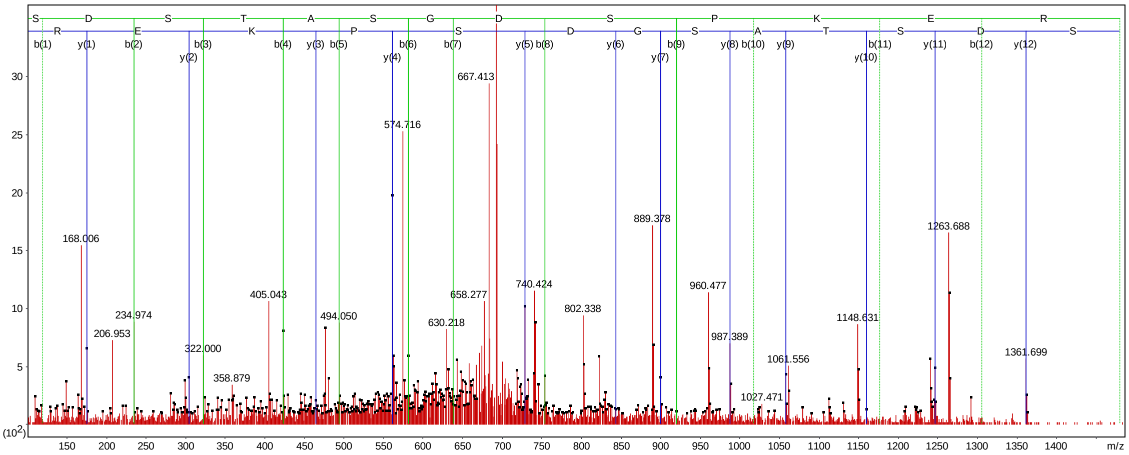

42 Accession No. CAN61094

42.1 PIDT$_p$FIDVNIK

#	b	b⁺⁺	Seq.	y	y⁺⁺	#
1	134.136	67.571	P			11
2	247.220	124.114	I	1293.688	647.348	10
3	362.247	181.627	D	1180.604	590.806	9
4	543.261	272.134	T	1065.577	533.292	8
5	690.329	345.668	F	884.563	442.785	7
6	803.413	402.210	I	737.495	369.251	6
7	918.440	459.724	D	624.411	312.709	5
8	1017.509	509.258	V	509.384	255.196	4
9	1131.551	566.279	N	410.315	205.661	3
10	1244.636	622.821	I	296.273	148.640	2
11			K	183.188	92.098	1

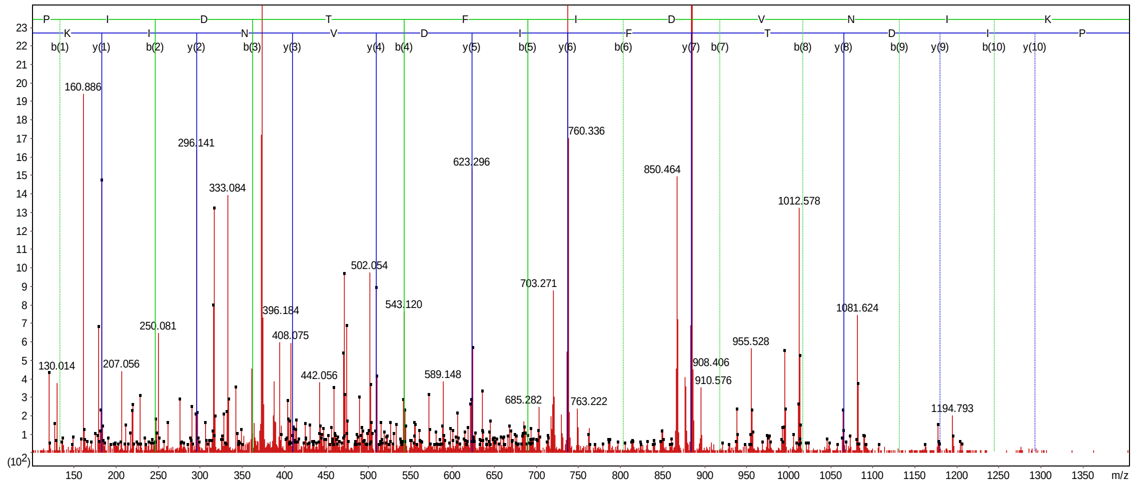

43 Accession No. BAJ99149

43.1 LQS$_p$PGAQQYYGTSR

#	b	b⁺⁺	Seq.	y	y⁺⁺	#
1	150.167	75.587	L			14
2	278.226	139.616	Q	1522.632	761.820	13
3	445.224	223.116	S	1394.574	697.790	12
4	542.277	271.642	P	1227.575	614.291	11
5	599.298	300.153	G	1130.523	565.765	10
6	670.335	335.671	A	1073.501	537.254	9
7	798.394	399.701	Q	1002.464	501.736	8
8	926.452	463.730	Q	874.405	437.706	7
9	1089.516	545.262	Y	746.347	373.677	6
10	1252.579	626.793	Y	583.283	292.145	5
11	1309.601	655.304	G	420.220	210.614	4
12	1410.648	705.828	T	363.199	182.103	3
13	1497.680	749.344	S	262.151	131.579	2
14			R	175.119	88.063	1

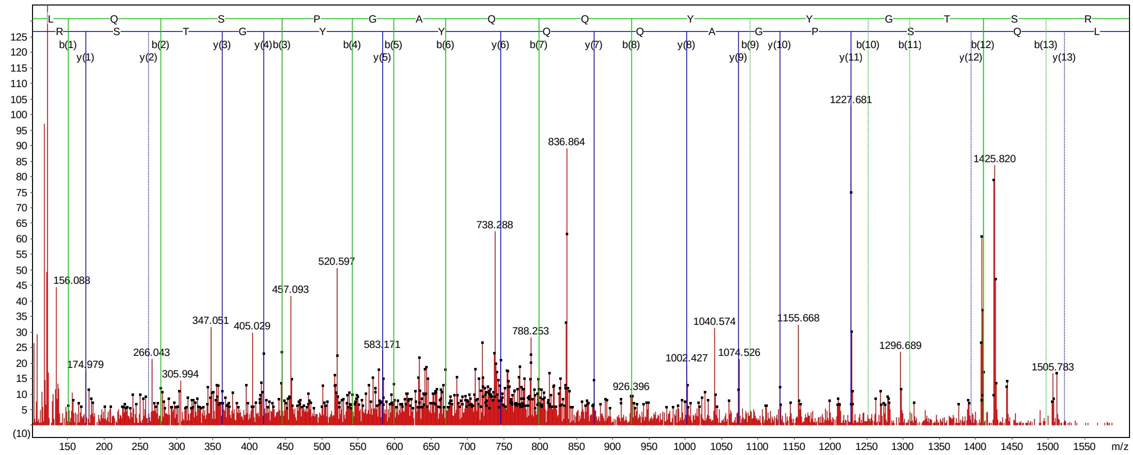

44 Accession No. XP_004980077

44.1 RGY$_p$GGGGGGGGGGGGGGGGGGGGGGR

#	b	b++	Seq.	y	y++	#
1	189.165	95.086	R			26
2	246.186	123.597	G	1729.642	865.325	25
3	489.216	245.112	Y	1672.621	836.814	24
4	546.237	273.622	G	1429.591	715.299	23
5	603.259	302.133	G	1372.570	686.788	22
6	660.280	330.644	G	1315.548	658.278	21
7	717.302	359.155	G	1258.527	629.767	20
8	774.323	387.665	G	1201.505	601.256	19
9	831.345	416.176	G	1144.484	572.746	18
10	888.366	444.687	G	1087.462	544.235	17
11	945.388	473.197	G	1030.441	515.724	16
12	1002.409	501.708	G	973.419	487.213	15
13	1059.431	530.219	G	916.398	458.703	14
14	1116.452	558.730	G	859.377	430.192	13
15	1173.473	587.240	G	802.355	401.681	12
16	1230.495	615.751	G	745.334	373.170	11
17	1287.516	644.262	G	688.312	344.660	10
18	1344.538	672.773	G	631.291	316.149	9
19	1401.559	701.283	G	574.269	287.638	8
20	1458.581	729.794	G	517.248	259.128	7
21	1515.602	758.305	G	460.226	230.617	6
22	1572.624	786.816	G	403.205	202.106	5
23	1629.645	815.326	G	346.183	173.595	4
24	1686.667	843.837	G	289.162	145.085	3
25	1743.688	872.348	G	232.140	116.574	2
26			R	175.119	88.063	1

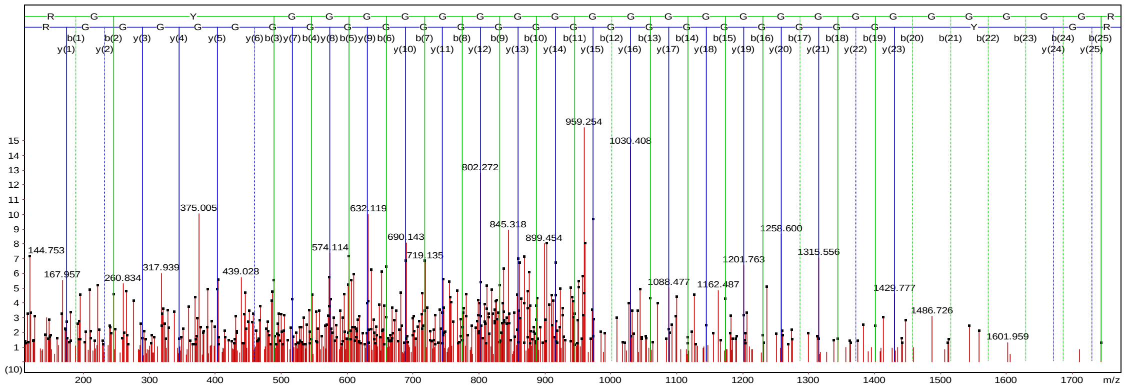

45 Accession No. ACO61321

45.1 MAS$_p$AASSSTETAAPK

#	b	b++	Seq.	y	y++	#
1	210.134	105.571	M			15
2	281.171	141.089	A	1394.659	697.833	14
3	448.169	224.588	S	1323.622	662.315	13
4	519.207	260.107	A	1156.624	578.816	12
5	590.244	295.625	A	1085.587	543.297	11
6	677.276	339.142	S	1014.550	507.778	10
7	764.308	382.658	S	927.517	464.262	9
8	851.340	426.174	S	840.485	420.746	8
9	952.387	476.697	T	753.453	377.230	7
10	1081.430	541.219	E	652.406	326.707	6
11	1182.478	591.743	T	523.363	262.185	5
12	1253.515	627.261	A	422.315	211.661	4
13	1324.552	662.780	A	351.278	176.143	3
14	1421.605	711.306	P	280.241	140.624	2
15			K	183.188	92.098	1

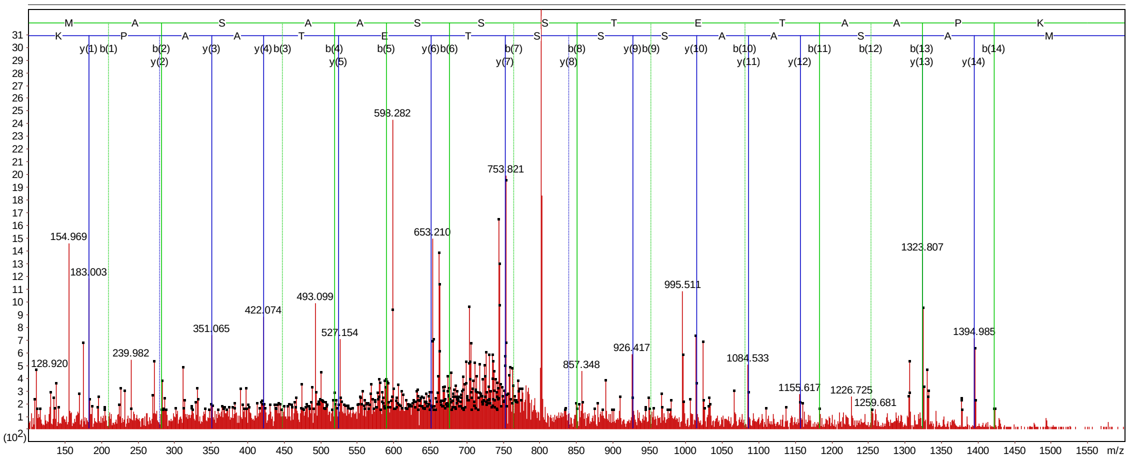

46 Accession No. EMT25919

46.1 QVS$_p$VDVPDVR

#	b	b⁺⁺	Seq.	y	y⁺⁺	#
1	165.142	83.074	Q			10
2	264.210	132.609	V	1065.498	533.252	9
3	431.208	216.108	S	966.429	483.718	8
4	530.277	265.642	V	799.431	400.219	7
5	645.304	323.155	D	700.362	350.685	6
6	744.372	372.690	V	585.335	293.171	5
7	841.425	421.216	P	486.267	243.637	4
8	956.452	478.730	D	389.214	195.111	3
9	1055.520	528.264	V	274.187	137.597	2
10			R	175.119	88.063	1

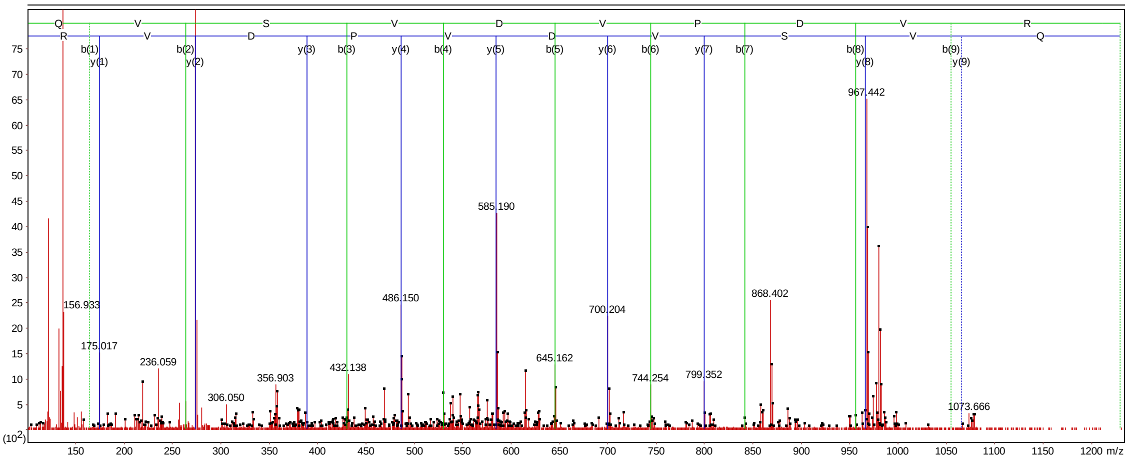

47 Accession No. EMS60685

47.1 TLDLTGVQPPS$_p$PKPK

#	b	b⁺⁺	Seq.	y	y⁺⁺	#
1	130.086	65.547	T			15
2	243.170	122.089	L	1612.871	806.939	14
3	358.197	179.602	D	1499.787	750.397	13
4	471.281	236.144	L	1384.760	692.884	12
5	572.329	286.668	T	1271.676	636.342	11
6	629.350	315.179	G	1170.628	585.818	10
7	728.419	364.713	V	1113.607	557.307	9
8	856.477	428.742	Q	1014.538	507.773	8
9	953.530	477.269	P	886.480	443.744	7
10	1050.583	525.795	P	789.427	395.217	6
11	1217.581	609.294	S	692.374	346.691	5
12	1314.634	657.821	P	525.376	263.192	4
13	1470.760	735.884	K	428.323	214.665	3
14	1567.813	784.410	P	272.197	136.602	2
15			K	175.144	88.076	1

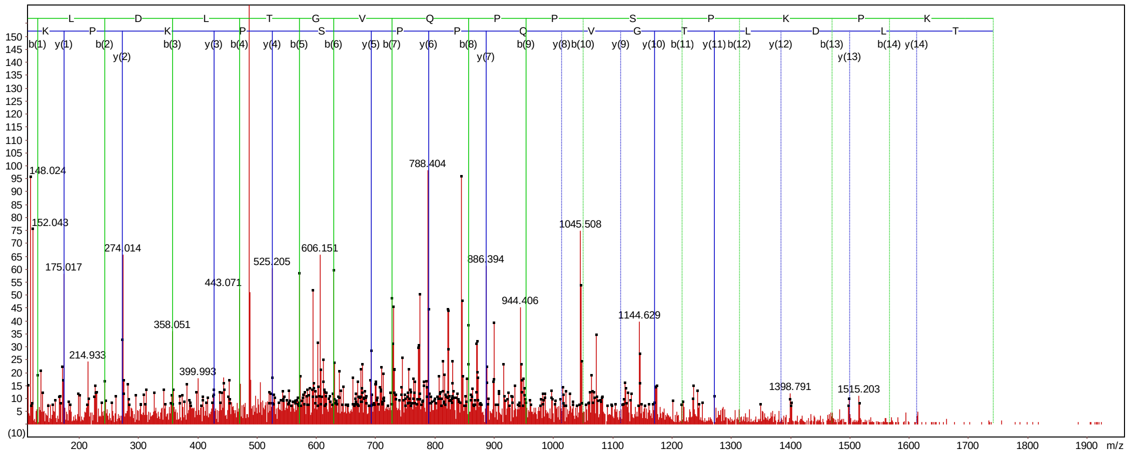

48 Accession No. CBF59389

48.1 RESLY$_p$GSLSS$_p$LEDDIVR

#	b	b^{++}	Seq.	y	y^{++}	#
1	185.140	93.073	R			17
2	314.182	157.595	E	1942.808	971.908	16
3	401.214	201.111	S	1813.766	907.386	15
4	514.298	257.653	L	1726.734	863.870	14
5	757.328	379.168	Y	1613.650	807.328	13
6	814.349	407.678	G	1370.620	685.814	12
7	901.382	451.194	S	1313.598	657.303	11
8	1014.466	507.736	L	1226.566	613.787	10
9	1101.498	551.252	S	1113.482	557.245	9
10	1268.496	634.752	S	1026.450	513.729	8
11	1381.580	691.294	L	859.452	430.230	7
12	1510.623	755.815	E	746.368	373.688	6
13	1625.650	813.328	D	617.325	309.166	5
14	1740.677	870.842	D	502.298	251.653	4
15	1853.761	927.384	I	387.271	194.139	3
16	1952.829	976.918	V	274.187	137.597	2
17			R	175.119	88.063	1

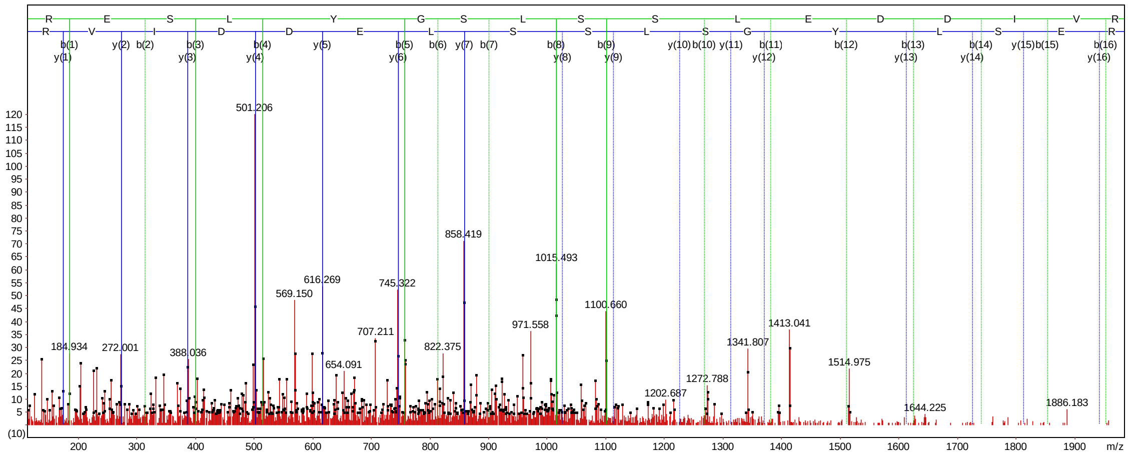

49 Accession No. DAA41082

49.1 NLFFFS$_p$RLAGR

#	b	b^{++}	Seq.	y	y^{++}	#
1	143.082	72.044	N			11
2	256.166	128.586	L	1293.650	647.329	10
3	403.234	202.121	F	1180.566	590.787	9
4	550.302	275.655	F	1033.498	517.253	8
5	697.371	349.189	F	886.429	443.718	7
6	864.369	432.688	S	739.361	370.184	6
7	1020.470	510.739	R	572.363	286.685	5
8	1133.554	567.281	L	416.262	208.634	4
9	1204.591	602.799	A	303.178	152.092	3
10	1261.613	631.310	G	232.140	116.574	2
11			R	175.119	88.063	1

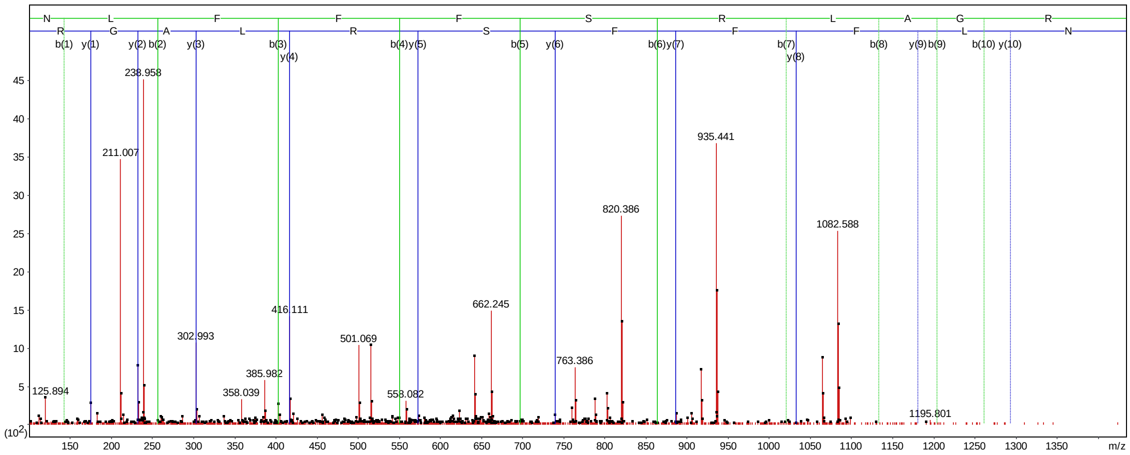

50 Accession No. XP_010104076

50.1 EEEPEQYWQT$_p$AGER

#	b	b^{++}	Seq.	y	y^{++}	#
1	166.126	83.566	E			14
2	295.168	148.088	E	1702.674	851.841	13
3	424.211	212.609	E	1573.632	787.320	12
4	521.263	261.135	P	1444.589	722.798	11
5	650.306	325.657	E	1347.537	674.272	10
6	778.365	389.686	Q	1218.494	609.751	9
7	941.428	471.218	Y	1090.435	545.721	8
8	1127.507	564.257	W	927.372	464.190	7
9	1255.566	628.287	Q	741.293	371.150	6
10	1436.580	718.794	T	613.234	307.121	5
11	1507.617	754.312	A	432.220	216.614	4
12	1564.638	782.823	G	361.183	181.095	3
13	1693.681	847.344	E	304.162	152.584	2
14			R	175.119	88.063	1

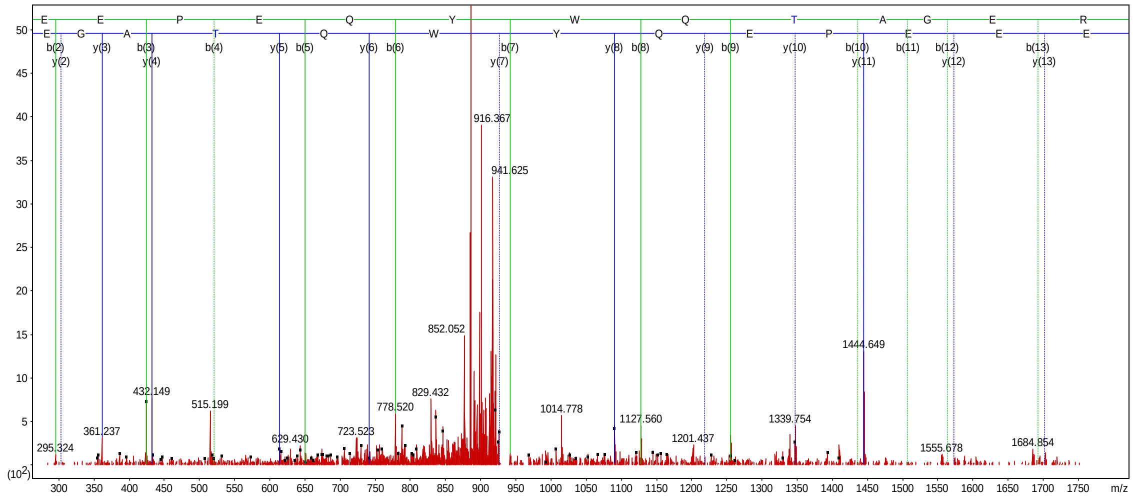

Supplement: Supplementary Figure S8 — Schematic presentation of Na2CO3 responsive mechanisms in leaves from alkaligrass The Na2CO3 responsive proteins from both leaf and chloroplast proteomes were integrated into subcellular pathways. A. Thermal dissipation. B. State transition. C. Photosynthetic electron transfer. D. Photorespiration and Calvin cycle. E. PSII repair cycle. F. ROS scavenging. G. Chlorophyll biosynthesis. H. Chloroplast movement and thylakoid membrane stability. I. Ionic and osmotic homeostasis. J. Gene expression, protein turnover, and transport. The scale bar indicates log2 transformed protein abundance, enzyme activity, and substrate content ratios (compared with 0 mM Na2CO3 treatment). The ratio was ranging from -3.0 to 3.0 with 7 different colors from green to red. P in a red (increased in phosphorylation level), green (decreased in phosphorylation level), and blue (can be phosphorylated by other protein, but did not identified in our phosphoproteomic study) circle indicates phosphorylated protein. The solid line indicates single-step reaction, dashed line indicates multistep reaction, and the dotted line indicates movement of proteins or other substances. Abbreviations: 1,3-BPG, 1,3-bisphosphoglyceric acid; 131-Hydroxy-Mg-Proto ME, 131-hydroxy-magnesium-protoporphyrin IX 13-monomethyl ester; 3-PGA, 3-phosphoglyceric acid; 30S/50S, 30S/50S ribosomal protein; 40S/60S, eukaryotic small/large ribosomal subunit; ABA, abscisic acid; ABC1K, activator of bc1 complex kinases; ALA, 5-aminolaevulinic acid; AlaRS, alanine-tRNA ligase; APX, ascorbate peroxidase; AsA, ascorbic acid; CA, carbonic anhydrase; CAT, catalase; Coprogen III, corproporphyrinogen III; CP, chlorophyll a/b binding protein; CPOX, coproporphyrinogen-III oxidase; CSase, cysteine synthase; CURT1A, curvature thylakoid 1A protein; Cyt f, cytochrome f; D1/D2, photosystem II D1/D2 protein; DHA, dehydroascorbic acid; DHAP, dihydroxyacetone phosphate; DHAR, dehydroascorbate reductase; DLD, dihydrolipoyl dehydrogenase; DLP, dynamin-2 [file mmc1.pdf]
