# Supplementary material for: Na2CO3-responsive Photosynthetic and ROS Scavenging Mechanisms in Chloroplasts of Alkaligrass Revealed by Phosphoproteomics
Source: Genomics Proteomics Bioinformatics. 2020 Jul 16;18(3):271–88. doi: 10.1016/j.gpb.2018.10.011 (PMC7801222; doi:10.1016/j.gpb.2018.10.011)

Leaf phosphoproteome

1 Accession No. CAA59049/ gi|666054

1.1 APERPIWFPGS[Pho]TPPPWLDGS[Pho]LPGDFGFDPWGLGSDPESLR

Residue	b	b+2	y	y+2
A	376.2497	188.6285	4814.2311	2407.6192
P	473.3025	237.1549	4438.9886	2219.9980
E	602.3451	301.6762	4341.9359	2171.4716
R	758.4462	379.7267	4212.8933	2106.9503
P	855.4990	428.2531	4056.7922	2028.8997
I	968.5830	484.7952	3959.7394	1980.3733
W	1154.6624	577.8348	3846.6553	1923.8313
F	1301.7308	651.3690	3660.5760	1830.7916
P	1398.7835	699.8954	3513.5076	1757.2574
G	1455.8050	728.4061	3416.4548	1708.7311
S[Pho]	1622.8034	811.9053	3359.4334	1680.2203
T	1723.8510	862.4292	3192.4350	1596.7211
P	1820.9038	910.9555	3091.3873	1546.1973
P	1917.9566	959.4819	2994.3346	1497.6709
P	2015.0093	1008.0083	2897.2818	1449.1445
W	2201.0886	1101.0480	2800.2291	1400.6182
L	2314.1727	1157.5900	2614.1497	1307.5785
D	2429.1996	1215.1035	2501.0657	1251.0365
G	2486.2211	1243.6142	2386.0387	1193.5230
S[Pho]	2653.2195	1327.1134	2329.0173	1165.0123
L	2766.3035	1383.6554	2162.0189	1081.5131
P	2863.3563	1432.1818	2048.9348	1024.9711
G	2920.3778	1460.6925	1951.8821	976.4447

Residue	b	b+2	y	y+2
S[Pho]	2653.2195	1327.1134	2329.0173	1165.0123
L	2766.3035	1383.6554	2162.0189	1081.5131
P	2863.3563	1432.1818	2048.9348	1024.9711
G	2920.3778	1460.6925	1951.8821	976.4447
D	3035.4047	1518.2060	1894.8606	947.9339
F	3182.4731	1591.7402	1779.8337	890.4205
G	3239.4946	1620.2509	1632.7653	816.8863
F	3386.5630	1693.7851	1575.7438	788.3755
D	3501.5899	1751.2986	1428.6754	714.8413
P	3598.6427	1799.8250	1313.6484	657.3279
W	3784.7220	1892.8646	1216.5957	608.8015
G	3841.7435	1921.3754	1030.5164	515.7618
L	3954.8275	1977.9174	973.4949	487.2511
G	4011.8490	2006.4281	860.4108	430.7091
S	4098.8810	2049.9442	803.3894	402.1983
D	4213.9080	2107.4576	716.3573	358.6823
P	4310.9607	2155.9840	601.3304	301.1688
E	4440.0033	2220.5053	504.2776	252.6425
S	4527.0354	2264.0213	375.2350	188.1212
L	4640.1194	2320.5634	288.2030	144.6051
R	4796.2205	2398.6139	175.1190	88.0631

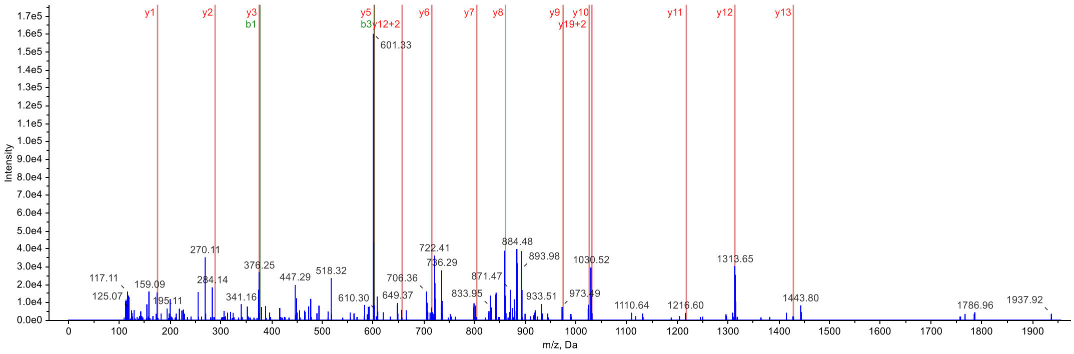

2 Accession No. XP_003618083/ gi|357495589

2.1 QSLS[Pho]YLDGSLPGDFGFDPLGLSDPEGTGGFIEPR

Residue	b	b+2	y	y+2
Q	433.2712	217.1392	3906.8417	1953.9245
S	520.3032	260.6553	3474.5777	1737.7925
L	633.3873	317.1973	3387.5457	1694.2765
S[Pho]	800.3857	400.6965	3274.4616	1637.7345
Y	963.4490	482.2281	3107.4633	1554.2353
L	1076.5331	538.7702	2944.3999	1472.7036
D	1191.5600	596.2836	2831.3159	1416.1616
G	1248.5815	624.7944	2716.2889	1358.6481
S[Dhy]	1317.6029	659.3051	2659.2675	1330.1374
L	1430.6870	715.8471	2590.2460	1295.6266
P	1527.7398	764.3735	2477.1619	1239.0846
G	1584.7612	792.8842	2380.1092	1190.5582
D	1699.7882	850.3977	2323.0877	1162.0475
F	1846.8566	923.9319	2208.0608	1104.5340
G	1903.8780	952.4427	2060.9924	1030.9998
F	2050.9465	1025.9769	2003.9709	1002.4891
D	2165.9734	1083.4903	1856.9025	928.9549
P	2263.0262	1132.0167	1741.8755	871.4414
L	2376.1102	1188.5588	1644.8228	822.9150

Residue	b	b+2	y	y+2
G	2433.1317	1217.0695	1531.7387	766.3730
L	2546.2158	1273.6115	1474.7172	737.8623
S	2633.2478	1317.1275	1361.6332	681.3202
D	2748.2747	1374.6410	1274.6012	637.8042
P	2845.3275	1423.1674	1159.5742	580.2907
E	2974.3701	1487.6887	1062.5214	531.7644
G	3031.3915	1516.1994	933.4789	467.2431
T	3132.4392	1566.7232	876.4574	438.7323
G	3189.4607	1595.2340	775.4097	388.2085
G	3246.4822	1623.7447	718.3883	359.6978
F	3393.5506	1697.2789	661.3668	331.1870
I	3506.6346	1753.8210	514.2984	257.6528
E	3635.6772	1818.3422	401.2143	201.1108
P	3732.7300	1866.8686	272.1717	136.5895
R	3888.8311	1944.9192	175.1190	88.0631

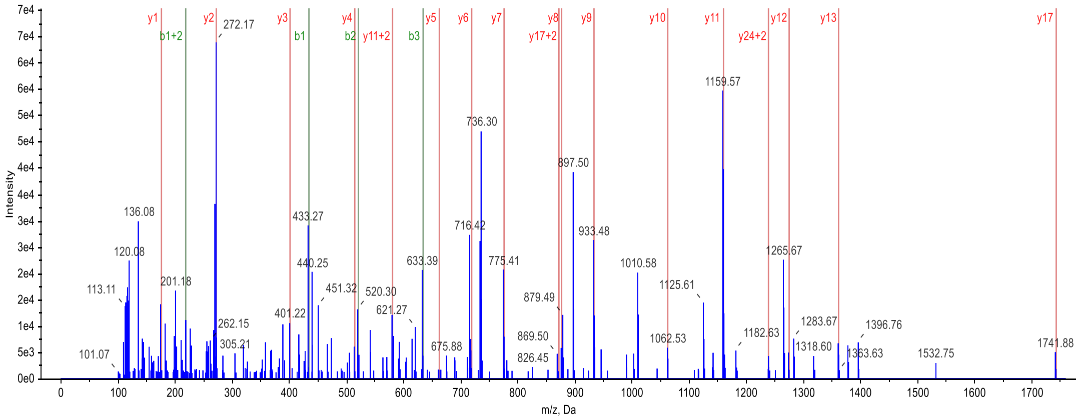

3 Accession No. CDI44335/ gi|550555057

3.1 VAGGPLGEVVDPLYPGGS[Pho]LDPLGLADDPEAFAELK

Residue	b	b+2	y	y+2
V	404.2810	202.6442	4471.3452	2236.1762
A	475.3182	238.1627	4068.0714	2034.5394
G	532.3396	266.6735	3997.0343	1999.0208
G	589.3611	295.1842	3940.0129	1970.5101
P	686.4139	343.7106	3882.9914	1941.9993
L	799.4979	400.2526	3785.9386	1893.4730
G	856.5194	428.7633	3672.8546	1836.9309
E	985.5620	493.2846	3615.8331	1808.4202
V	1084.6304	542.8188	3486.7905	1743.8989
V	1183.6988	592.3530	3387.7221	1694.3647
D	1298.7257	649.8665	3288.6537	1644.8305
P	1395.7785	698.3929	3173.6267	1587.3170
L	1508.8626	754.9349	3076.5740	1538.7906
Y[IT8]	1976.1313	988.5693	2963.4899	1482.2486
P	2073.1840	1037.0957	2496.2212	1248.6143
G	2130.2055	1065.6064	2399.1685	1200.0879
G	2187.2270	1094.1171	2342.1470	1171.5771
S[Pho]	2354.2253	1177.6163	2285.1255	1143.0664
L	2467.3094	1234.1583	2118.1272	1059.5672
D	2582.3363	1291.6718	2005.0431	1003.0252

Residue	b	b+2	y	y+2
P	2679.3891	1340.1982	1890.0162	945.5117
L	2792.4731	1396.7402	1792.9634	896.9853
G	2849.4946	1425.2509	1679.8793	840.4433
L	2962.5787	1481.7930	1622.8579	811.9326
A	3033.6158	1517.3115	1509.7738	755.3905
D	3148.6427	1574.8250	1438.7367	719.8720
D	3263.6697	1632.3385	1323.7098	662.3585
P	3360.7224	1680.8649	1208.6828	604.8450
E	3489.7650	1745.3862	1111.6301	556.3187
A	3560.8021	1780.9047	982.5875	491.7974
F	3707.8706	1854.4389	911.5503	456.2788
A	3778.9077	1889.9575	764.4819	382.7446
E	3907.9503	1954.4788	693.4448	347.2260
L	4021.0343	2011.0208	564.4022	282.7048
K[IT8]	4453.3347	2227.1710	451.3182	226.1627

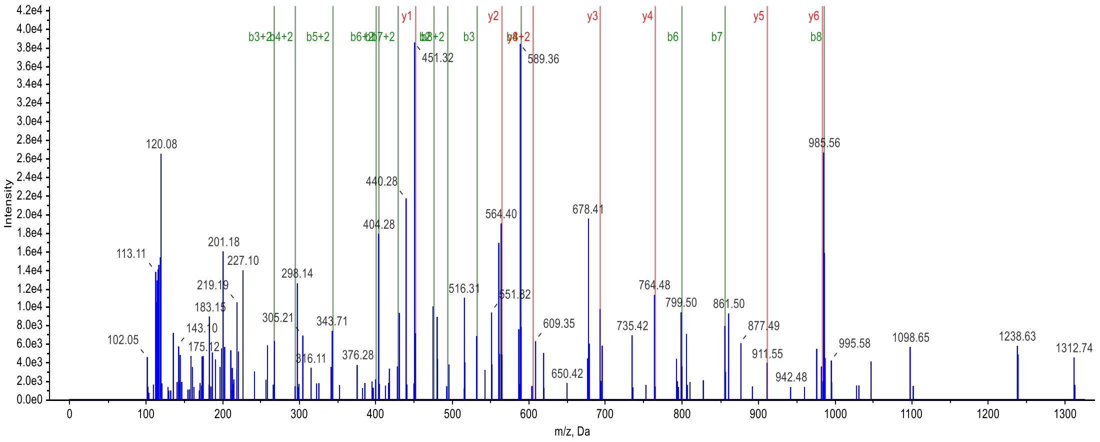

4 Accession No. EMS50795/gi|473952979

4.1 VLYLGPLSGEPPS[Pho]YLTGEFPGDYGWDTAGLSADPETFAK

Residue	b	b+2	y	y+2
V	404.2810	202.6442	4828.3183	2414.6628
L	517.3651	259.1862	4425.0446	2213.0259
Y	680.4284	340.7179	4311.9605	2156.4839
L	793.5125	397.2599	4148.8972	2074.9522
G	850.5340	425.7706	4035.8131	2018.4102
P	947.5867	474.2970	3978.7916	1989.8995
L	1060.6708	530.8390	3881.7389	1941.3731
S	1147.7028	574.3551	3768.6548	1884.8310
G	1204.7243	602.8658	3681.6228	1841.3150
E	1333.7669	667.3871	3624.6013	1812.8043
P	1430.8196	715.9135	3495.5587	1748.2830
P	1527.8724	764.4398	3398.5060	1699.7566
S[Pho]	1694.8708	847.9390	3301.4532	1651.2302
Y	1857.9341	929.4707	3134.4548	1567.7311
L	1971.0182	986.0127	2971.3915	1486.1994
T	2072.0658	1036.5366	2858.3074	1429.6574
G	2129.0873	1065.0473	2757.2598	1379.1335
E[NaX]	2280.1118	1140.5596	2700.2383	1350.6228
F	2427.1803	1214.0938	2549.2138	1275.1105
P	2524.2330	1262.6201	2402.1454	1201.5763
G	2581.2545	1291.1309	2305.0926	1153.0499

Residue	b	b+2	y	y+2
D	2696.2814	1348.6444	2248.0711	1124.5392
Y	2859.3448	1430.1760	2133.0442	1067.0257
G	2916.3662	1458.6867	1969.9809	985.4941
W	3102.4455	1551.7264	1912.9594	956.9833
D	3217.4725	1609.2399	1726.8801	863.9437
T	3318.5202	1659.7637	1611.8531	806.4302
A	3389.5573	1695.2823	1510.8055	755.9064
G	3446.5787	1723.7930	1439.7683	720.3878
L	3559.6628	1780.3350	1382.7469	691.8771
S	3646.6948	1823.8510	1269.6628	635.3350
A	3717.7319	1859.3696	1182.6308	591.8190
D	3832.7589	1916.8831	1111.5937	556.3005
P	3929.8116	1965.4095	996.5667	498.7870
E	4058.8542	2029.9308	899.5140	450.2606
T	4159.9019	2080.4546	770.4714	385.7393
F	4306.9703	2153.9888	669.4237	335.2155
A	4378.0074	2189.5074	522.3553	261.6813
K[IT8]	4810.3078	2405.6575	451.3182	226.1627

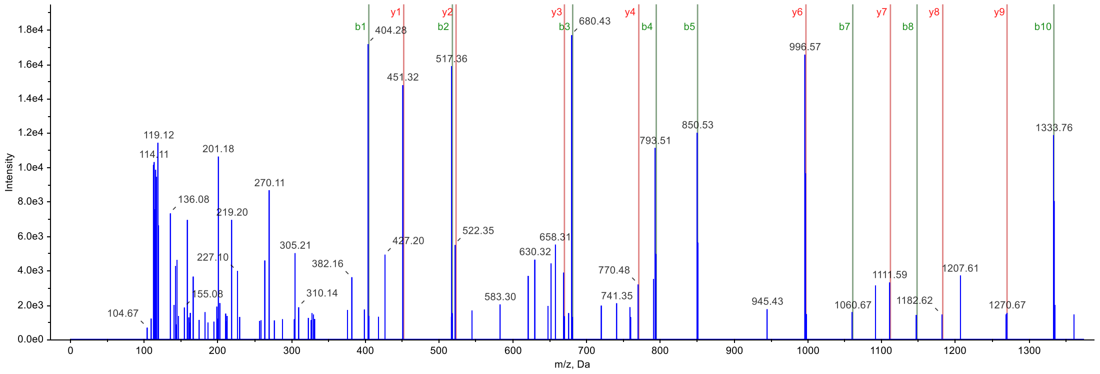

4.2 VLYLGPLSGEPPS[Pho]YLT[Pho]GEFPGDYGW[HKy]DT[Pho]AGLSADPETFAK[IT8]

Residue	b	b+2	y	y+2
V	404.2810	202.6442	4986.2589	2493.6331
L	517.3651	259.1862	4582.9851	2291.9962
Y	680.4284	340.7179	4469.9010	2235.4542
L	793.5125	397.2599	4306.8377	2153.9225
G	850.5340	425.7706	4193.7536	2097.3805
P	947.5867	474.2970	4136.7322	2068.8697
L	1060.6708	530.8390	4039.6794	2020.3433
S	1147.7028	574.3551	3926.5954	1963.8013
G	1204.7243	602.8658	3839.5633	1920.2853
E	1333.7669	667.3871	3782.5419	1891.7746
P	1430.8196	715.9135	3653.4993	1827.2533
P	1527.8724	764.4398	3556.4465	1778.7269
S[Pho]	1694.8708	847.9390	3459.3937	1730.2005
Y	1857.9341	929.4707	3292.3954	1646.7013
L	1971.0182	986.0127	3129.3321	1565.1697
T[Pho]	2152.0322	1076.5197	3016.2480	1508.6276
G	2209.0536	1105.0305	2835.2340	1418.1206
E	2338.0962	1169.5518	2778.2125	1389.6099
F	2485.1646	1243.0860	2649.1699	1325.0886
P	2582.2174	1291.6123	2502.1015	1251.5544

Residue	b	b+2	y	y+2
G	2639.2389	1320.1231	2405.0487	1203.0280
D	2754.2658	1377.6365	2348.0273	1174.5173
Y	2917.3291	1459.1682	2233.0003	1117.0038
G	2974.3506	1487.6789	2069.9370	1035.4721
W[HKy]	3180.4197	1590.7135	2012.9155	1006.9614
D	3295.4467	1648.2270	1806.8464	903.9268
T[Pho]	3476.4607	1738.7340	1691.8195	846.4134
A	3547.4978	1774.2525	1510.8055	755.9064
G	3604.5193	1802.7633	1439.7683	720.3878
L	3717.6033	1859.3053	1382.7469	691.8771
S	3804.6354	1902.8213	1269.6628	635.3350
A	3875.6725	1938.3399	1182.6308	591.8190
D	3990.6994	1995.8534	1111.5937	556.3005
P	4087.7522	2044.3797	996.5667	498.7870
E	4216.7948	2108.9010	899.5140	450.2606
T	4317.8425	2159.4249	770.4714	385.7393
F	4464.9109	2232.9591	669.4237	335.2155
A	4535.9480	2268.4776	522.3553	261.6813
K[IT8]	4968.2483	2484.6278	451.3182	226.1627

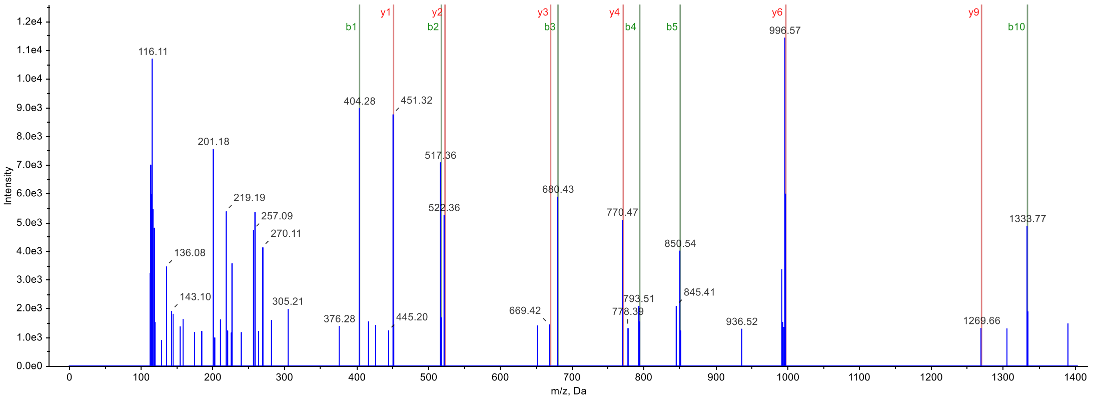

5 Accession No. EMT11232/ gi|475549279

5.1 VLYLGPLS[Pho]GDPPS[Pho]YLTGEFPGDYGWDT[Pho]AGLSADPETFAK

Residue	b	b+2	y	y+2
V	404.2810	202.6442	4984.2432	2492.6253
L	517.3651	259.1862	4580.9695	2290.9884
Y	680.4284	340.7179	4467.8854	2234.4463
L	793.5125	397.2599	4304.8221	2152.9147
G	850.5340	425.7706	4191.7380	2096.3726
P	947.5867	474.2970	4134.7165	2067.8619
L	1060.6708	530.8390	4037.6638	2019.3355
S[Pho]	1227.6692	614.3382	3924.5797	1962.7935
G	1284.6906	642.8489	3757.5813	1879.2943
D	1399.7176	700.3624	3700.5599	1850.7836
P	1496.7703	748.8888	3585.5329	1793.2701
P	1593.8231	797.4152	3488.4802	1744.7437
S[Pho]	1760.8215	880.9144	3391.4274	1696.2173
Y	1923.8848	962.4460	3224.4291	1612.7182
L	2036.9688	1018.9881	3061.3657	1531.1865
T	2138.0165	1069.5119	2948.2817	1474.6445
G	2195.0380	1098.0226	2847.2340	1424.1206
E	2324.0806	1162.5439	2790.2125	1395.6099
F	2471.1490	1236.0781	2661.1699	1331.0886
P	2568.2018	1284.6045	2514.1015	1257.5544

Residue	b	b+2	y	y+2
G	2625.2232	1313.1152	2417.0487	1209.0280
D	2740.2502	1370.6287	2360.0273	1180.5173
Y	2903.3135	1452.1604	2245.0003	1123.0038
G	2960.3350	1480.6711	2081.9370	1041.4721
W[2Ox]	3178.4041	1589.7057	2024.9155	1012.9614
D	3293.4310	1647.2192	1806.8464	903.9268
T[Pho]	3474.4450	1737.7262	1691.8195	846.4134
A	3545.4822	1773.2447	1510.8055	755.9064
G	3602.5036	1801.7555	1439.7683	720.3878
L	3715.5877	1858.2975	1382.7469	691.8771
S	3802.6197	1901.8135	1269.6628	635.3350
A	3873.6568	1937.3321	1182.6308	591.8190
D	3988.6838	1994.8455	1111.5937	556.3005
P	4085.7365	2043.3719	996.5667	498.7870
E	4214.7791	2107.8932	899.5140	450.2606
T	4315.8268	2158.4170	770.4714	385.7393
F	4462.8952	2231.9513	669.4237	335.2155
A	4533.9323	2267.4698	522.3553	261.6813
K[IT8]	4966.2327	2483.6200	451.3182	226.1627

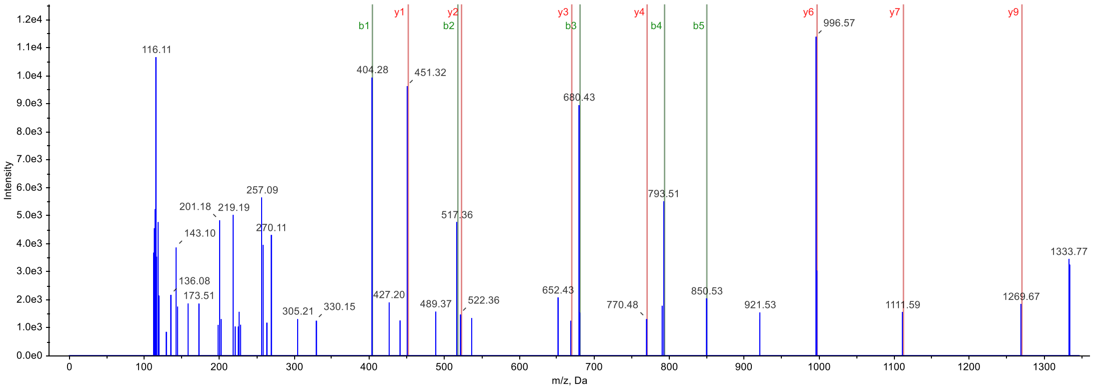

6 Accession No. EMT29003/ gi|475614191

6.1 VLYLGPLSGEPPS[Pho]YLNGEFPGDYGWDT[Pho]AGLS[Pho]ADPETFAK

Residue	b	b+2	y	y+2
V	404.2810	202.6442	4983.2592	2492.1332
L	517.3651	259.1862	4579.9854	2290.4964
Y	680.4284	340.7179	4466.9014	2233.9543
L	793.5125	397.2599	4303.8380	2152.4227
G	850.5340	425.7706	4190.7540	2095.8806
P	947.5867	474.2970	4133.7325	2067.3699
L	1060.6708	530.8390	4036.6798	2018.8435
S	1147.7028	574.3551	3923.5957	1962.3015
G	1204.7243	602.8658	3836.5637	1918.7855
E	1333.7669	667.3871	3779.5422	1890.2747
P	1430.8196	715.9135	3650.4996	1825.7534
P	1527.8724	764.4398	3553.4468	1777.2271
S[Pho]	1694.8708	847.9390	3456.3941	1728.7007
Y	1857.9341	929.4707	3289.3957	1645.2015
L	1971.0182	986.0127	3126.3324	1563.6698
N	2085.0611	1043.0342	3013.2483	1507.1278
G	2142.0826	1071.5449	2899.2054	1450.1063
E	2271.1251	1136.0662	2842.1839	1421.5956
F	2418.1936	1209.6004	2713.1413	1357.0743
P	2515.2463	1258.1268	2566.0729	1283.5401
G	2572.2678	1286.6375	2469.0202	1235.0137

Residue	b	b+2	y	y+2
D	2687.2947	1344.1510	2411.9987	1206.5030
Y	2850.3581	1425.6827	2296.9718	1148.9895
G	2907.3795	1454.1934	2133.9084	1067.4579
W[Kyn]	3097.4538	1549.2305	2076.8870	1038.9471
D	3212.4807	1606.7440	1886.8127	943.9100
T[Pho]	3393.4947	1697.2510	1771.7858	886.3965
A	3464.5318	1732.7695	1590.7718	795.8895
G	3521.5533	1761.2803	1519.7347	760.3710
L	3634.6373	1817.8223	1462.7132	731.8602
S[Pho]	3801.6357	1901.3215	1349.6291	675.3182
A	3872.6728	1936.8400	1182.6308	591.8190
D	3987.6998	1994.3535	1111.5937	556.3005
P	4084.7525	2042.8799	996.5667	498.7870
E	4213.7951	2107.4012	899.5140	450.2606
T	4314.8428	2157.9250	770.4714	385.7393
F	4461.9112	2231.4592	669.4237	335.2155
A	4532.9483	2266.9778	522.3553	261.6813
K[IT8]	4965.2486	2483.1280	451.3182	226.1627

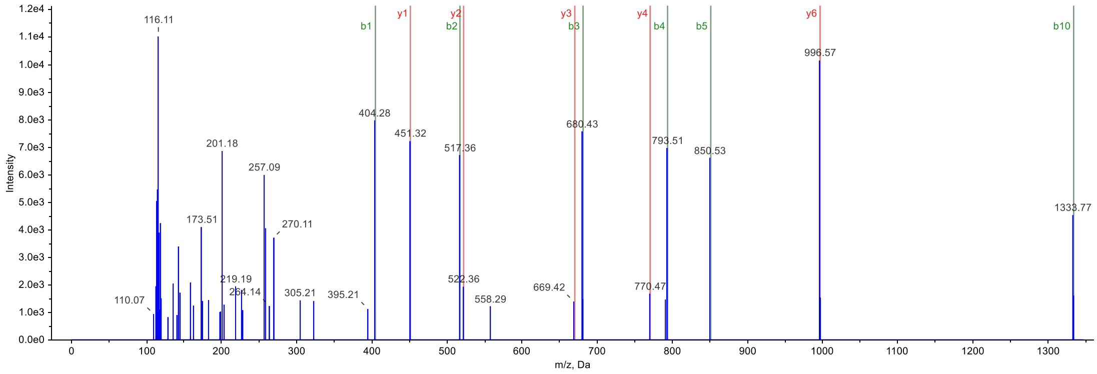

6.2 VLYLGPLSGEPPSYLNGEFPGDYGWDTAGLS[Pho]ADPETFAK

Residue	b	b+2	y	y+2
V	404.2810	202.6442	4985.3794	2493.1933
L	517.3651	259.1862	4582.1056	2291.5564
Y	680.4284	340.7179	4469.0215	2235.0144
L	793.5125	397.2599	4305.9582	2153.4827
G	850.5340	425.7706	4192.8741	2096.9407
P	947.5867	474.2970	4135.8527	2068.4300
L	1060.6708	530.8390	4038.7999	2019.9036
S	1147.7028	574.3551	3925.7159	1963.3616
G	1204.7243	602.8658	3838.6838	1919.8455
E	1333.7669	667.3871	3781.6624	1891.3348
P	1430.8196	715.9135	3652.6198	1826.8135
P	1527.8724	764.4398	3555.5670	1778.2871
S	1614.9044	807.9559	3458.5142	1729.7608
Y	1777.9678	889.4875	3371.4822	1686.2447
L	1891.0518	946.0296	3208.4189	1604.7131
N[Hex]	2167.1476	1084.0774	3095.3348	1548.1710
G	2224.1690	1112.5882	2819.2391	1410.1232
E	2353.2116	1177.1095	2762.2176	1381.6124
F	2500.2801	1250.6437	2633.1750	1317.0911
P	2597.3328	1299.1700	2486.1066	1243.5569
G	2654.3543	1327.6808	2389.0538	1195.0306
D	2769.3812	1385.1943	2332.0324	1166.5198

Residue	b	b+2	y	y+2
Y	2932.4446	1466.7259	2217.0054	1109.0064
G	2989.4660	1495.2366	2053.9421	1027.4747
W[Kyn]	3179.5402	1590.2738	1996.9206	998.9640
D	3294.5672	1647.7872	1806.8464	903.9268
T	3395.6149	1698.3111	1691.8195	846.4134
A	3466.6520	1733.8296	1590.7718	795.8895
G	3523.6734	1762.3404	1519.7347	760.3710
L	3636.7575	1818.8824	1462.7132	731.8602
S[Pho]	3803.7559	1902.3816	1349.6291	675.3182
A	3874.7930	1937.9001	1182.6308	591.8190
D	3989.8199	1995.4136	1111.5937	556.3005
P	4086.8727	2043.9400	996.5667	498.7870
E	4215.9153	2108.4613	899.5140	450.2606
T	4316.9630	2158.9851	770.4714	385.7393
F	4464.0314	2232.5193	669.4237	335.2155
A	4535.0685	2268.0379	522.3553	261.6813
K[IT8]	4967.3688	2484.1880	451.3182	226.1627

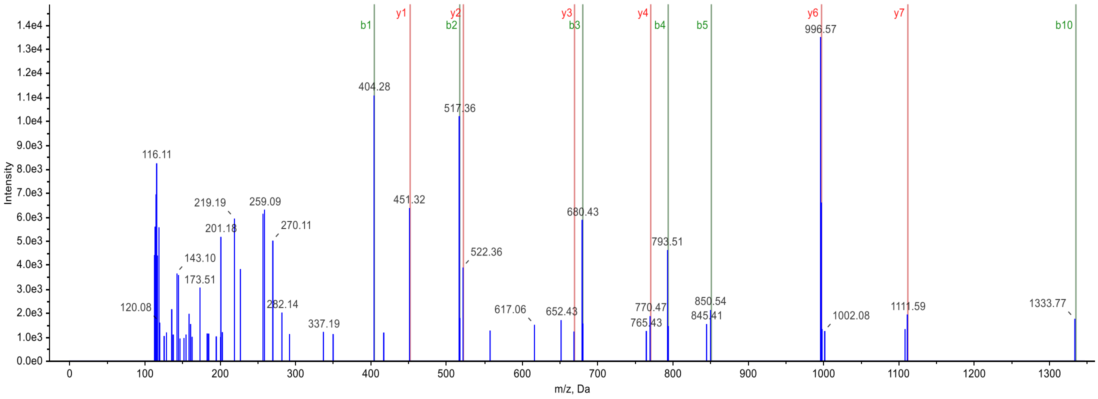

7 Accession No. CAA32109/gi|20182

7.1 VLYLGPLSGREPPS[Pho]YLTGEFPGDYGWDTAGLSADPETFAK

Residue	b	b+2	y	y+2
V	**404.2810**	202.6442	4984.4194	2492.7134
L	**517.3651**	259.1862	4581.1457	2291.0765
Y	**680.4284**	340.7179	4468.0616	2234.5344
L	**793.5125**	397.2599	4304.9983	2153.0028
G	**850.5340**	425.7706	4191.9142	2096.4607
P	947.5867	474.2970	4134.8927	2067.9500
L	1060.6708	530.8390	4037.8400	2019.4236
S	1147.7028	574.3551	3924.7559	1962.8816
G	1204.7243	602.8658	3837.7239	1919.3656
R	1360.8254	680.9163	3780.7024	1890.8549
E	1489.8680	745.4376	3624.6013	1812.8043
P	1586.9208	793.9640	3495.5587	1748.2830
P	1683.9735	842.4904	3398.5060	1699.7566
S[Pho]	1850.9719	925.9896	3301.4532	1651.2302
Y	2014.0352	1007.5212	3134.4548	1567.7311
L	2127.1193	1064.0633	2971.3915	1486.1994
T	2228.1670	1114.5871	2858.3074	1429.6574
G	2285.1884	1143.0978	2757.2598	1379.1335
E[NaX]	2436.2130	1218.6101	2700.2383	1350.6228
F	2583.2814	1292.1443	2549.2138	1275.1105
P	2680.3341	1340.6707	2402.1454	1201.5763

Residue	b	b+2	y	y+2
G	2737.3556	1369.1814	2305.0926	1153.0499
D	2852.3825	1426.6949	2248.0711	1124.5392
Y	3015.4459	1508.2266	2133.0442	1067.0257
G	3072.4673	1536.7373	1969.9809	985.4941
W	3258.5466	1629.7770	1912.9594	956.9833
D	3373.5736	1687.2904	1726.8801	863.9437
T	3474.6213	1737.8143	1611.8531	806.4302
A	3545.6584	1773.3328	1510.8055	755.9064
G	3602.6798	1801.8436	1439.7683	720.3878
L	3715.7639	1858.3856	1382.7469	691.8771
S	3802.7959	1901.9016	**1269.6628**	635.3350
A	3873.8330	1937.4202	1182.6308	591.8190
D	3988.8600	1994.9336	**1111.5937**	556.3005
P	4085.9128	2043.4600	**996.5667**	498.7870
E	4214.9553	2107.9813	899.5140	450.2606
T	4316.0030	2158.5052	**770.4714**	385.7393
F	4463.0714	2232.0394	**669.4237**	335.2155
A	4534.1086	2267.5579	**522.3553**	261.6813
K[IT8]	4966.4089	2483.7081	**451.3182**	226.1627

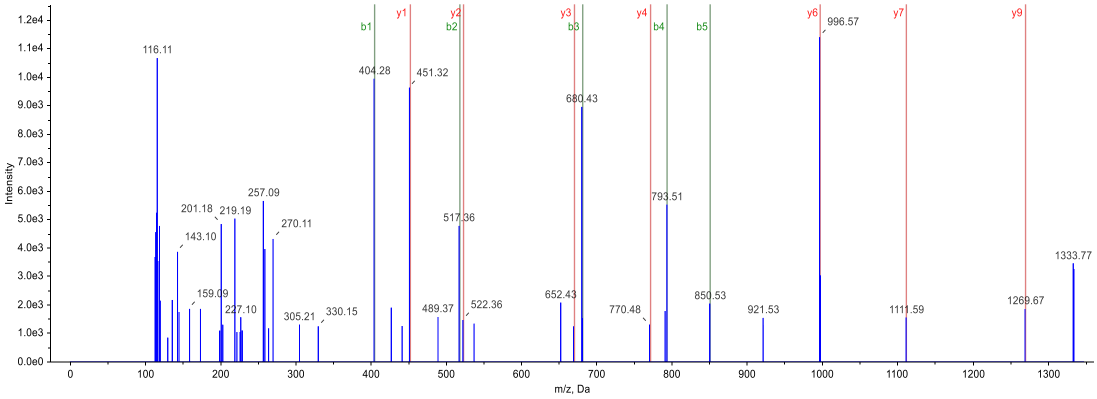

8 Accession No. 1908421A/ gi|445116

8.1 LAQNLAGEIIGT[Pho]RFEDADVK

Residue	b	b+2	y	y+2
L	**418.2967**	209.6520	2862.5222	1431.7647
A	**489.3338**	245.1705	2445.2328	1223.1200
Q	**617.3924**	309.1998	2374.1957	1187.6015
N	**731.4353**	**366.2213**	2246.1371	1123.5722
L	**844.5194**	**422.7633**	2132.0942	1066.5507
A	**915.5565**	458.2819	2019.0101	1010.0087
G	972.5780	486.7926	1947.9730	974.4901
E	**1101.6206**	551.3139	1890.9515	945.9794
I	**1214.7046**	607.8559	1761.9089	881.4581
I	1327.7887	664.3980	1648.8249	824.9161
G	1384.8101	692.9087	1535.7408	768.3740
T[Pho]	1565.8242	783.4157	1478.7194	739.8633
R	1721.9253	861.4663	1297.7053	649.3563
F	1868.9937	935.0005	1141.6042	571.3058
E	1998.0363	999.5218	994.5358	497.7715
D	2113.0632	1057.0352	865.4932	433.2503
A	2184.1003	1092.5538	**750.4663**	375.7368
D[1Me]	2313.1429	1157.0751	**679.4292**	340.2182
V	2412.2113	1206.6093	550.3866	275.6969
K[IT8]	2844.5117	1422.7595	**451.3182**	226.1627

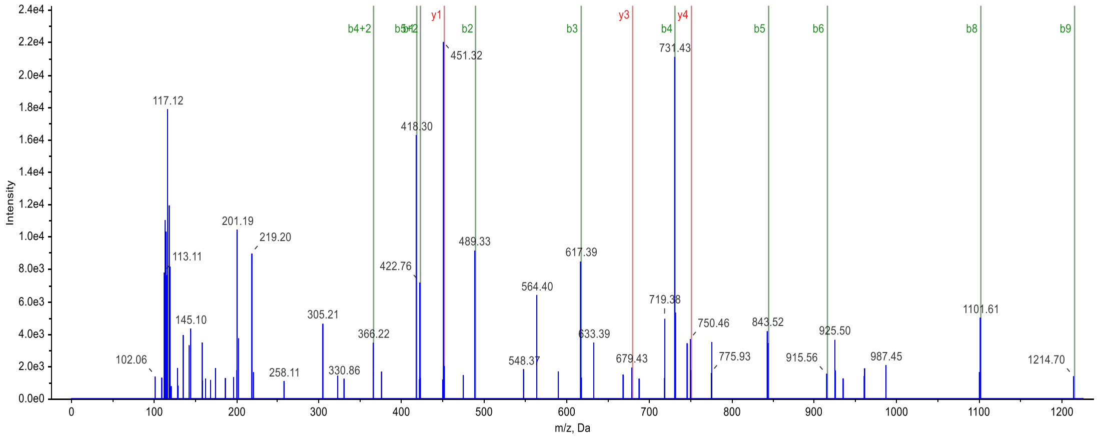

9 Accession No. CDI44415/gi|550554240

9.1 PAEYLQYDVDSLDQNLAQNLAGEIIGT[Pho]R

Residue	b	b+2	y	y+2
P	402.2654	201.6363	3490.7044	1745.8559
A	473.3025	237.1549	3089.4463	1545.2268
E	602.3451	301.6762	3018.4092	1509.7082
Y	765.4084	383.2079	2889.3666	1445.1869
L	878.4925	439.7499	2726.3033	1363.6553
Q	1006.5511	503.7792	2613.2192	1307.1132
Y	1169.6144	585.3108	2485.1606	1243.0840
D	1284.6413	642.8243	2322.0973	1161.5523
V	1383.7098	692.3585	2207.0704	1104.0388
D	1498.7367	749.8720	2108.0020	1054.5046
S	1585.7687	793.3880	1992.9750	996.9911
L	1698.8528	849.9300	1905.9430	953.4751
D	1813.8797	907.4435	1792.8589	896.9331
Q	1941.9383	971.4728	1677.8320	839.4196
N	2055.9812	1028.4943	1549.7734	775.3903

Residue	b	b+2	y	y+2
L	2169.0653	1085.0363	1435.7305	718.3689
A	2240.1024	1120.5548	1322.6464	661.8268
Q	2368.1610	1184.5841	1251.6093	626.3083
N	2482.2039	1241.6056	1123.5507	562.2790
L	2595.2880	1298.1476	1009.5078	505.2575
A	2666.3251	1333.6662	896.4237	448.7155
G	2723.3466	1362.1769	825.3866	413.1969
E	2852.3892	1426.6982	768.3651	384.6862
I	2965.4732	1483.2403	639.3226	320.1649
I	3078.5573	1539.7823	526.2385	263.6229
G	3135.5788	1568.2930	413.1544	207.0809
T[Pho]	3316.5928	1658.8000	356.1330	178.5701
R	3472.6939	1736.8506	175.1190	88.0631

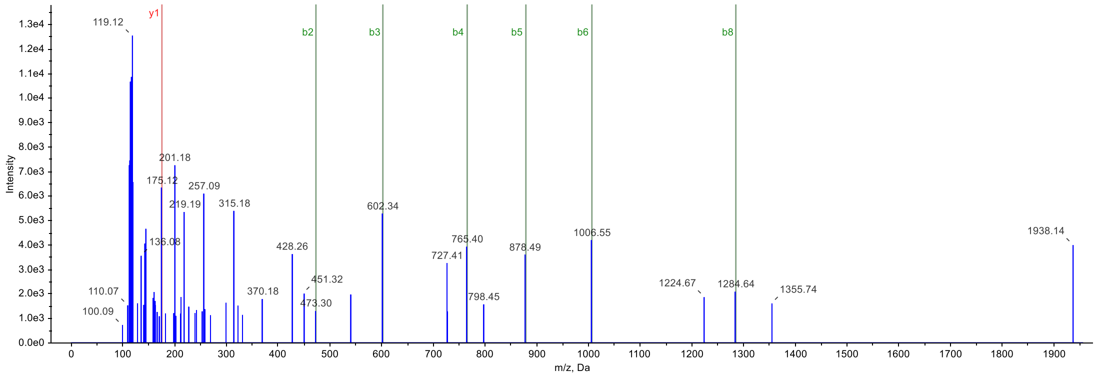

10 Accession No. XP_003562892/gi|357122377

10.1 PAEYLQYDPDS[Pho]LDQNLAQNLAGEVIGTRFEDADIK

Residue	b	b+2	y	y+2
P	402.2654	201.6363	4613.2544	2307.1309
A	473.3025	237.1549	4211.9963	2106.5018
E	602.3451	301.6762	4140.9592	2070.9832
Y	765.4084	383.2079	4011.9166	2006.4619
L	878.4925	439.7499	3848.8533	1924.9303
Q	1006.5511	503.7792	3735.7692	1868.3883
Y	1169.6144	585.3108	3607.7106	1804.3590
D	1284.6413	642.8243	3444.6473	1722.8273
P[Oxi]	1397.6890	699.3482	3329.6204	1665.3138
D	1512.7160	756.8616	3216.5727	1608.7900
S[Pho]	1679.7143	840.3608	3101.5458	1551.2765
L	1792.7984	896.9028	2934.5474	1467.7773
D	1907.8253	954.4163	2821.4633	1411.2353
Q	2035.8839	1018.4456	2706.4364	1353.7218
N	2149.9268	1075.4671	2578.3778	1289.6925
L	2263.0109	1132.0091	2464.3349	1232.6711
A	2334.0480	1167.5276	2351.2508	1176.1290
Q	2462.1066	1231.5569	2280.2137	1140.6105
N	2576.1495	1288.5784	2152.1551	1076.5812

Residue	b	b+2	y	y+2
L	2689.2336	1345.1204	2038.1122	1019.5597
A	2760.2707	1380.6390	1925.0281	963.0177
G	2817.2922	1409.1497	1853.9910	927.4991
E	2946.3348	1473.6710	1796.9696	898.9884
V	3045.4032	1523.2052	1667.9270	834.4671
I	3158.4872	1579.7473	1568.8586	784.9329
G	3215.5087	1608.2580	1455.7745	728.3909
T	3316.5564	1658.7818	1398.7530	699.8802
R	3472.6575	1736.8324	1297.7053	649.3563
F	3619.7259	1810.3666	1141.6042	571.3058
E	3748.7685	1874.8879	994.5358	497.7715
D	3863.7954	1932.4014	865.4932	433.2503
A	3934.8326	1967.9199	750.4663	375.7368
D	4049.8595	2025.4334	679.4292	340.2182
I	4162.9436	2081.9754	564.4022	282.7048
K[IT8]	4595.2439	2298.1256	451.3182	226.1627

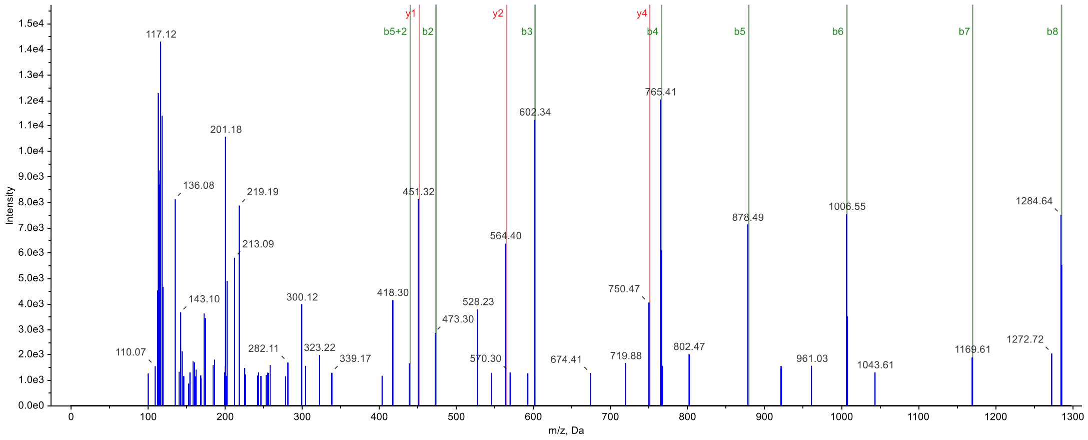

11 Accession No. EMT03206/gi|475485989

11.1 TGALLLDGNT[Pho]LNYFGNS[Pho]IPINLILAVVAEVVLVGGAEYYR

Residue	b	b+2	y	y+2
T	173.0921	87.0497	4453.2496	2227.1284
G	230.1135	115.5604	4281.1648	2141.0860
A	301.1506	151.0790	4224.1433	2112.5753
L	414.2347	207.6210	4153.1062	2077.0568
L	527.3188	264.1630	4040.0222	2020.5147
L	640.4028	320.7051	3926.9381	1963.9727
D	755.4298	378.2185	3813.8540	1907.4307
G	812.4512	406.7293	3698.8271	1849.9172
N	926.4942	463.7507	3641.8056	1821.4065
T[Pho]	1107.5082	554.2577	3527.7627	1764.3850
L	1220.5922	610.7998	3346.7487	1673.8780
N	1334.6352	667.8212	3233.6646	1617.3360
Y	1497.6985	749.3529	3119.6217	1560.3145
F	1644.7669	822.8871	2956.5584	1478.7828
G	1701.7884	851.3978	2809.4900	1405.2486
N	1815.8313	908.4193	2752.4685	1376.7379
S[Pho]	1982.8297	991.9185	2638.4256	1319.7164
I	2095.9137	1048.4605	2471.4272	1236.2172
P	2192.9665	1096.9869	2358.3431	1179.6752
I	2306.0506	1153.5289	2261.2904	1131.1488
N	2420.0935	1210.5504	2148.2063	1074.6068
L	2533.1775	1267.0924	2034.1634	1017.5853
I	2646.2616	1323.6344	1921.0793	961.0433

Residue	b	b+2	y	y+2
L	2759.3457	1380.1765	1807.9953	904.5013
A	2830.3828	1415.6950	1694.9112	847.9592
V	2929.4512	1465.2292	1623.8741	812.4407
V	3028.5196	1514.7634	1524.8057	762.9065
A	3099.5567	1550.2820	1425.7373	713.3723
E	3228.5993	1614.8033	1354.7001	677.8537
V	3327.6677	1664.3375	1225.6575	613.3324
V	3426.7362	1713.8717	1126.5891	563.7982
L	3539.8202	1770.4137	1027.5207	514.2640
V	3638.8886	1819.9480	914.4367	457.7220
G	3695.9101	1848.4587	815.3682	408.1878
G	3752.9316	1876.9694	758.3468	379.6770
A	3823.9687	1912.4880	701.3253	351.1663
E	3953.0113	1977.0093	630.2882	315.6477
Y	4116.0746	2058.5409	501.2456	251.1264
Y	4279.1379	2140.0726	338.1823	169.5948
R	4435.2390	2218.1232	175.1190	88.0631

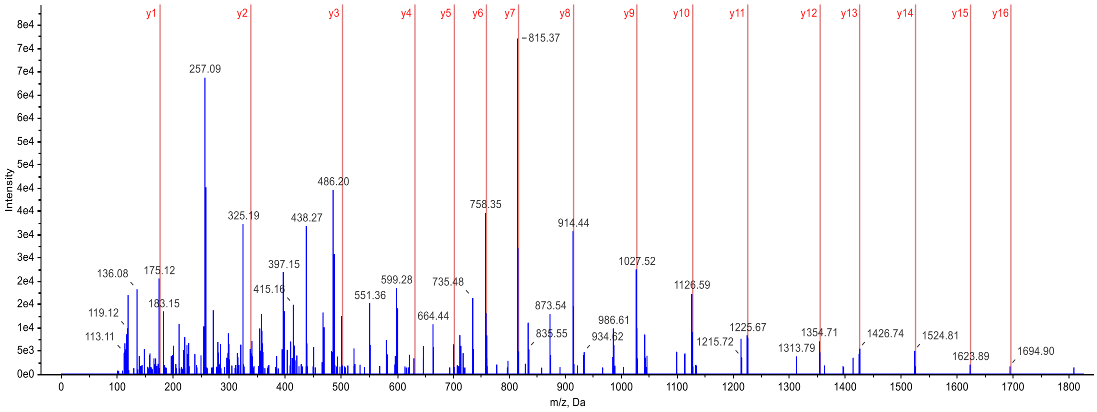

11.2 TGALLLDGNTLNYFGNS[Pho]IPINLILAVVAEVVLVGGAEYYR

Residue	b	b+2	y	y+2
T	406.2603	203.6338	4804.4808	2402.7441
G	463.2818	232.1445	4399.2278	2200.1175
A	534.3189	267.6631	4342.2063	2171.6068
L	647.4030	324.2051	4271.1692	2136.0882
L	760.4870	380.7471	4158.0852	2079.5462
L	873.5711	437.2892	4045.0011	2023.0042
D	988.5980	494.8027	3931.9170	1966.4622
G	1045.6195	523.3134	3816.8901	1908.9487
N	1159.6624	580.3348	3759.8686	1880.4379
T	1260.7101	630.8587	3645.8257	1823.4165
L	1373.7942	687.4007	3544.7780	1772.8926
N	1487.8371	744.4222	3431.6940	1716.3506
Y	1650.9004	825.9538	3317.6510	1659.3292
F	1797.9688	899.4881	3154.5877	1577.7975
G	1854.9903	927.9988	3007.5193	1504.2633
N	1969.0332	985.0202	2950.4978	1475.7525
S[Pho]	2136.0316	1068.5194	2836.4549	1418.7311
I	2249.1156	1125.0615	2669.4565	1335.2319
P	2346.1684	1173.5878	2556.3725	1278.6899
I	2459.2525	1230.1299	2459.3197	1230.1635

Residue	b	b+2	y	y+2
N[Dea]	2574.2794	1287.6433	2346.2356	1173.6215
L	2687.3635	1344.1854	2231.2087	1116.1080
I	2800.4475	1400.7274	2118.1246	1059.5660
L	2913.5316	1457.2694	2005.0406	1003.0239
A	2984.5687	1492.7880	1891.9565	946.4819
V	3083.6371	1542.3222	1820.9194	910.9633
V	3182.7055	1591.8564	1721.8510	861.4291
A	3253.7427	1627.3750	1622.7826	811.8949
E[GPE]	3579.8306	1790.4189	1551.7455	776.3764
V	3678.8990	1839.9531	1225.6575	613.3324
V	3777.9674	1889.4873	1126.5891	563.7982
L	3891.0515	1946.0294	1027.5207	514.2640
V	3990.1199	1995.5636	914.4367	457.7220
G	4047.1413	2024.0743	815.3682	408.1878
G	4104.1628	2052.5850	758.3468	379.6770
A	4175.1999	2088.1036	701.3253	351.1663
E	4304.2425	2152.6249	630.2882	315.6477
Y	4467.3058	2234.1566	501.2456	251.1264
Y	4630.3692	2315.6882	338.1823	169.5948
R	4786.4703	2393.7388	175.1190	88.0631

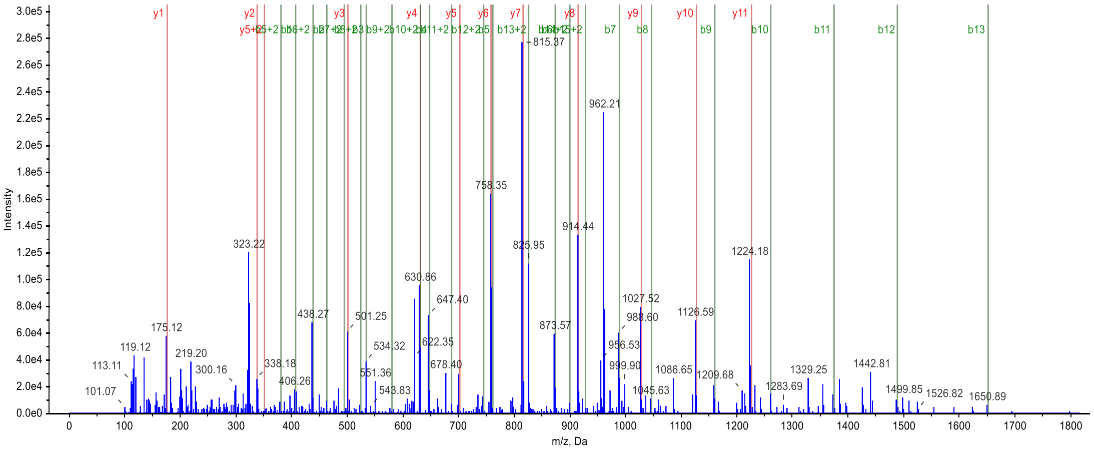

12 Accession No. XP_003564708/gi|357126063

12.1 GILS[Pho]QLNLETGIPIYEAEPLLLFFILFTLLGAIGALGDR

Residue	b	b+2	y	y+2
G	100.0393	50.5233	4339.3281	2170.1677
I	213.1234	107.0653	4240.2961	2120.6517
L	326.2074	163.6074	4127.2120	2064.1096
S[Pho]	493.2058	247.1065	4014.1279	2007.5676
Q	621.2644	311.1358	3847.1296	1924.0684
L	734.3484	367.6779	3719.0710	1860.0391
N	848.3914	424.6993	3605.9869	1803.4971
L	961.4754	481.2414	3491.9440	1746.4756
E	1090.5180	545.7626	3378.8599	1689.9336
T	1191.5657	596.2865	3249.8174	1625.4123
G	1248.5872	624.7972	3148.7697	1574.8885
I	1361.6712	681.3393	3091.7482	1546.3777
P	1458.7240	729.8656	2978.6641	1489.8357
I	1571.8081	786.4077	2881.6114	1441.3093
Y	1734.8714	867.9393	2768.5273	1384.7673
E	1863.9140	932.4606	2605.4640	1303.2356
A	1934.9511	967.9792	2476.4214	1238.7143
E	2063.9937	1032.5005	2405.3843	1203.1958
P[Oxi]	2177.0414	1089.0243	2276.3417	1138.6745

Residue	b	b+2	y	y+2
L	2290.1254	1145.5663	2163.2940	1082.1506
L	2403.2095	1202.1084	2050.2099	1025.6086
L	2516.2936	1258.6504	1937.1259	969.0666
F	2663.3620	1332.1846	1824.0418	912.5245
F	2810.4304	1405.7188	1676.9734	838.9903
I	2923.5144	1462.2609	1529.9050	765.4561
L	3036.5985	1518.8029	**1416.8209**	708.9141
F	3183.6669	1592.3371	**1303.7369**	652.3721
T	3284.7146	1642.8609	**1156.6684**	578.8379
L	3397.7987	1699.4030	1055.6208	528.3140
L	3510.8827	1755.9450	**942.5367**	471.7720
G	3567.9042	1784.4557	**829.4526**	415.2300
A	3638.9413	1819.9743	**772.4312**	386.7192
I	3752.0254	1876.5163	**701.3941**	351.2007
G	3809.0468	1905.0271	**588.3100**	294.6586
A	3880.0839	1940.5456	**531.2885**	266.1479
L	3993.1680	1997.0876	**460.2514**	230.6293
G	4050.1895	2025.5984	**347.1674**	174.0873
D	4165.2164	2083.1118	290.1459	145.5766
R	4321.3175	2161.1624	**175.1190**	88.0631

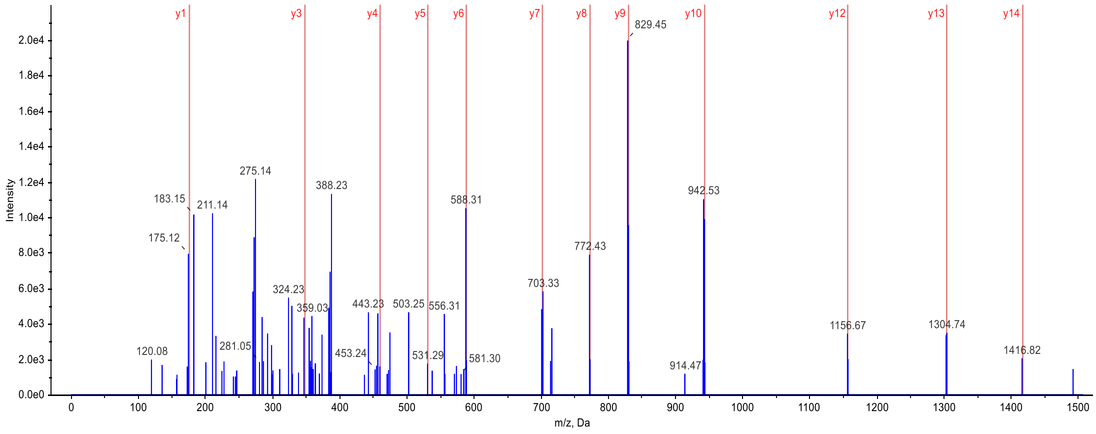

13 Accession No. ABC02751/gi|83317154

13.1 TLFNGT[Pho]FVLAGR

Residue	b	b+2	y	y+2
T	144.0655	72.5364	1417.6875	709.3474
L	257.1496	129.0784	1274.6293	637.8183
F	404.2180	202.6126	1161.5452	581.2763
N	518.2609	259.6341	1014.4768	507.7420
G	575.2824	**288.1448**	900.4339	450.7206
T[Pho]	756.2964	378.6518	843.4124	422.2099
F	903.3648	452.1860	**662.3984**	331.7028
V	1002.4332	501.7203	**515.3300**	258.1686
L	1115.5173	558.2623	**416.2616**	208.6344
A	1186.5544	593.7808	**303.1775**	152.0924
G	1243.5759	622.2916	**232.1404**	116.5738
R	1399.6770	700.3421	**175.1190**	88.0631

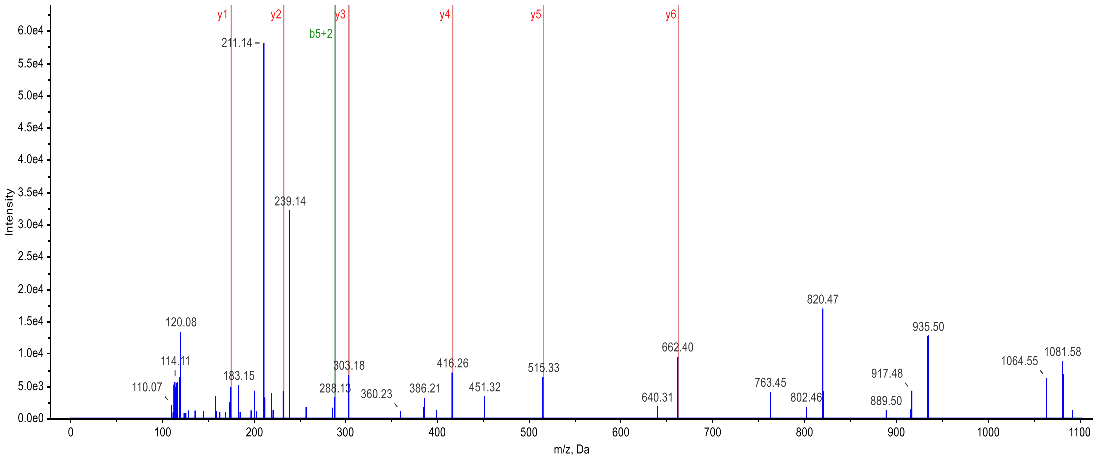

14 Accession No. P69555/gi|61230121

14.1 AT[Pho]QTVEDSSKPR

Residue	b	b+2	y	y+2
A	376.2497	188.6285	2007.0368	1004.0220
T[Pho]	557.2638	279.1355	1631.7943	816.4008
Q	685.3223	343.1648	1450.7803	725.8938
T	786.3700	393.6886	1322.7217	661.8645
V	885.4384	443.2229	1221.6740	611.3407
E	1014.4810	507.7441	1122.6056	561.8065
D	1129.5080	565.2576	993.5630	497.2852
S	1216.5400	608.7736	878.5361	439.7717
S	1303.5720	652.2896	791.5041	396.2557
K[IT8]	1735.8723	868.4398	704.4720	352.7397
P	1832.9251	916.9662	272.1717	136.5895
R	1989.0262	995.0167	175.1190	88.0631

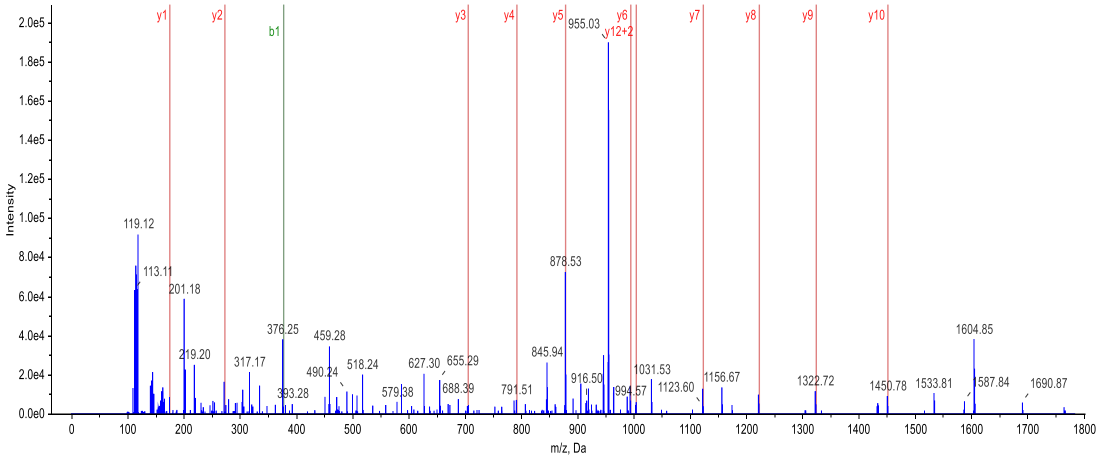

14.2 AT[Pho]QT[Pho]VEDSSKPRPK

Residue	b	b+2	y	y+2
A	376.2497	188.6285	2616.3562	1308.6817
T[Pho]	557.2638	279.1355	2241.1137	1121.0605
Q	685.3223	343.1648	2060.0997	1030.5535
T[Pho]	866.3363	433.6718	1932.0411	966.5242
V	965.4048	483.2060	1751.0271	876.0172
E	1094.4474	547.7273	1651.9587	826.4830
D	1209.4743	605.2408	1522.9161	761.9617
S	1296.5063	648.7568	1407.8892	704.4482
S	1383.5384	692.2728	1320.8572	660.9322
K[IT8]	1815.8387	908.4230	1233.8251	617.4162
P	1912.8914	956.9494	801.5248	401.2660
R	2068.9925	1034.9999	704.4720	352.7397
P	2166.0453	1083.5263	548.3709	274.6891
K[IT8]	2598.3456	1299.6765	451.3182	226.1627

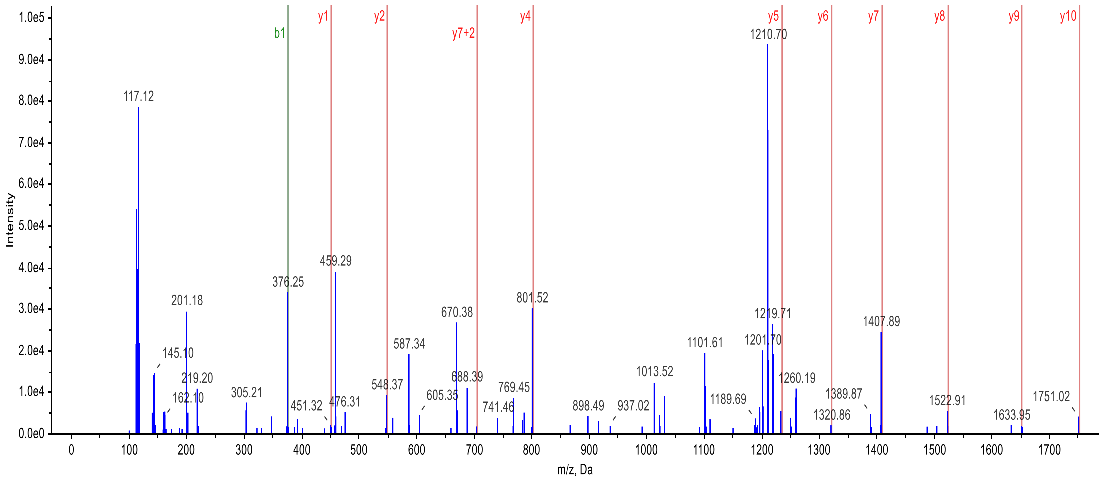

15 Accession No. CCO16140/gi|412991295

15.1 GGSTGYDNAVALPARS[Pho]DADDLQKENNK

Residue	b	b+2	y	y+2
G	362.2341	181.6207	3816.8692	1908.9382
G	419.2556	210.1314	3455.6424	1728.3248
S	506.2876	253.6474	3398.6209	1699.8141
T	607.3353	304.1713	3311.5889	1656.2981
G	664.3567	332.6820	3210.5412	1605.7742
Y	827.4201	414.2137	3153.5197	1577.2635
D	942.4470	471.7271	2990.4564	1495.7318
N[Dea]	1057.4739	529.2406	2875.4294	1438.2184
A	1128.5111	564.7592	2760.4025	1380.7049
V	1227.5795	614.2934	2689.3654	1345.1863
A	1298.6166	649.8119	2590.2970	1295.6521
L	1411.7007	706.3540	2519.2599	1260.1336
P[Oxi]	1524.7483	762.8778	2406.1758	1203.5915
A	1595.7854	798.3964	2293.1281	1147.0677

Residue	b	b+2	y	y+2
R	1751.8866	876.4469	2222.0910	1111.5491
S[Pho]	1918.8849	959.9461	2065.9899	1033.4986
D	2033.9119	1017.4596	1898.9915	949.9994
A	2104.9490	1052.9781	1783.9646	892.4859
D	2219.9759	1110.4916	1712.9275	856.9674
D	2335.0029	1168.0051	1597.9005	799.4539
L	2448.0869	1224.5471	1482.8736	741.9404
Q	2576.1455	1288.5764	1369.7895	685.3984
K[IT8]	3008.4458	1504.7265	1241.7309	621.3691
E	3137.4884	1569.2478	809.4306	405.2190
N	3251.5313	1626.2693	680.3880	340.6977
N[Dea]	3366.5583	1683.7828	566.3451	283.6762
K[IT8]	3798.8586	1899.9329	451.3182	226.1627

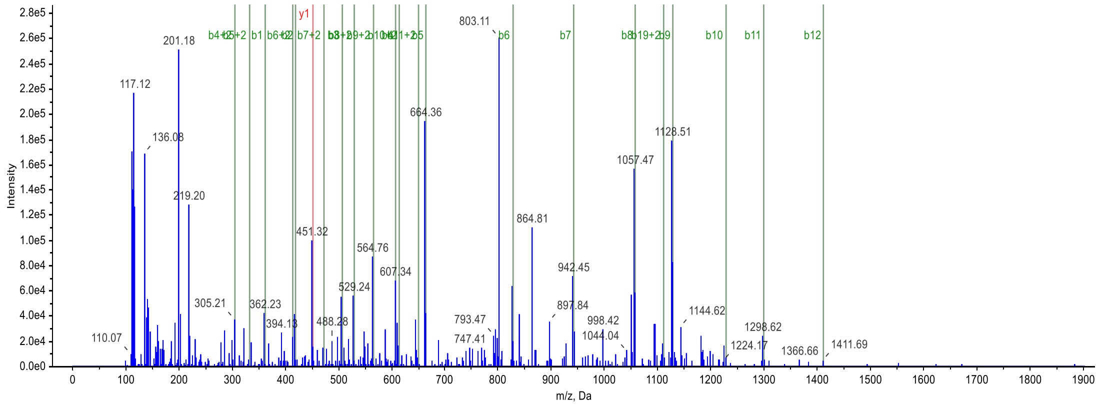

16 Accession No. BAJ97488/gi|326528933

16.1 VDGPAPSAGGT[Pho]ASR

Residue	b	b+2	y	y+2
V	404.2810	202.6442	1668.8259	834.9166
D	519.3080	260.1576	1265.5522	633.2797
G	576.3295	288.6684	1150.5252	575.7663
P	673.3822	337.1947	1093.5038	547.2555
A	744.4193	372.7133	996.4510	498.7291
P	841.4721	421.2397	925.4139	463.2106
S	928.5041	464.7557	828.3611	414.6842
A[3Me]	1041.5882	521.2977	741.3291	371.1682
G	1098.6097	549.8085	628.2450	314.6262
G	1155.6311	578.3192	571.2236	286.1154
T[Pho]	1336.6451	668.8262	514.2021	257.6047
A	1407.6822	704.3448	333.1881	167.0977
S	1494.7143	747.8608	262.1510	131.5791
R	1650.8154	825.9113	175.1190	88.0631

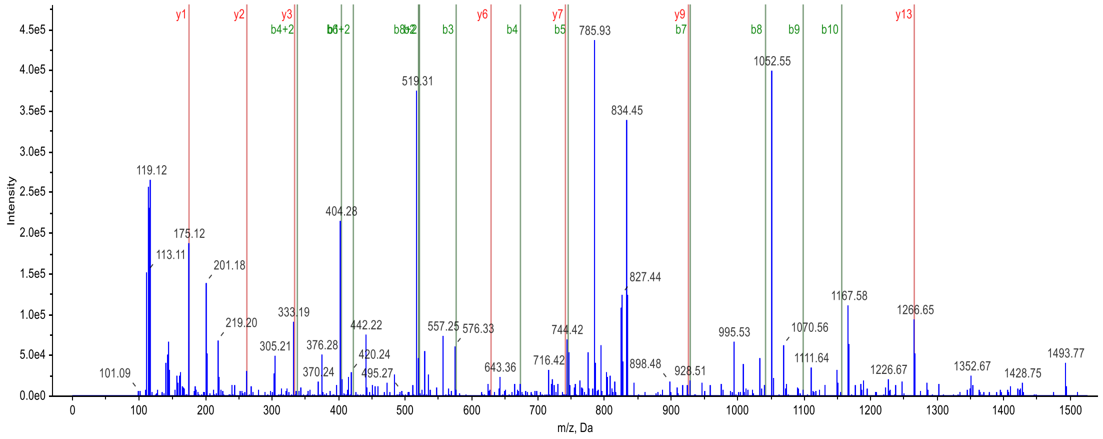

17 Accession No. ABA95524/gi|77552727

17.1 GLAYDIS[Pho]DDQQDITR

Residue	b	b+2	y	y+2
G	362.2341	181.6207	2398.1747	1199.5910
L	475.3182	238.1627	2036.9479	1018.9776
A	546.3553	273.6813	1923.8638	962.4356
Y[IT8]	1013.6240	507.3156	1852.8267	926.9170
D	1128.6509	564.8291	1385.5580	693.2827
I	1241.7350	621.3711	1270.5311	635.7692
S[Pho]	1408.7333	704.8703	1157.4470	579.2272
D	1523.7603	762.3838	990.4487	495.7280
D	1638.7872	819.8972	875.4217	438.2145
Q	1766.8458	883.9265	760.3948	380.7010
Q	1894.9044	947.9558	632.3362	316.6717
D	2009.9313	1005.4693	504.2776	252.6425
I	2123.0154	1062.0113	389.2507	195.1290
T	2224.0631	1112.5352	276.1666	138.5870
R	2380.1642	1190.5857	175.1190	88.0631

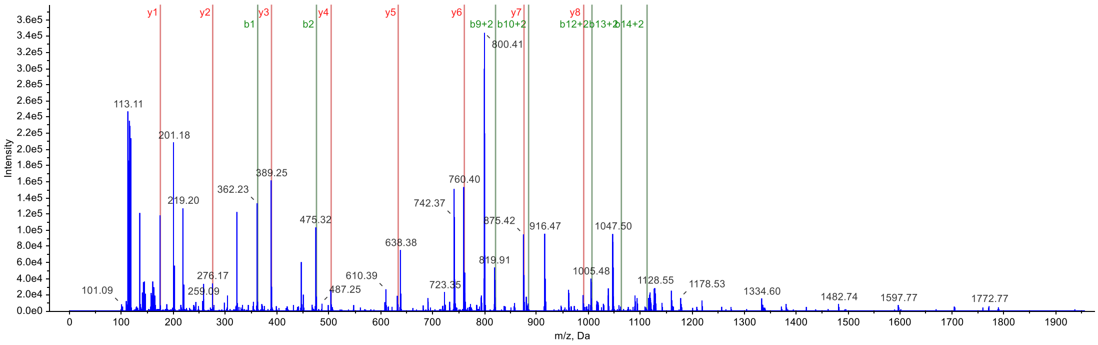

18 Accession No. ABB03412/gi|77798470

18.1 GLDFT[Pho]KD[Pho]DENVNSQPFMR

Residue	b	b+2	y	y+2
G	58.0287	29.5180	2577.1035	1289.0554
L	171.1128	86.0600	2520.0821	1260.5447
D	286.1397	143.5735	2406.9980	1204.0026
F	433.2082	217.1077	2291.9710	1146.4892
T[Pho]	614.2222	307.6147	2144.9026	1072.9550
K[IT8]	1046.5225	523.7649	1963.8886	982.4479
D[Pho]	1241.5158	621.2615	1531.5883	766.2978
D	1356.5427	678.7750	1336.5950	668.8012
E	1485.5853	743.2963	1221.5681	611.2877
N	1599.6282	800.3178	1092.5255	546.7664
V	1698.6966	849.8520	978.4826	489.7449
N	1812.7396	906.8734	879.4141	440.2107
S	1899.7716	950.3894	765.3712	383.1892
Q	2027.8302	1014.4187	678.3392	339.6732
P	2124.8829	1062.9451	550.2806	275.6439
F	2271.9514	1136.4793	453.2279	227.1176
M	2402.9918	1201.9996	306.1594	153.5834
R	2559.0930	1280.0501	175.1190	88.0631

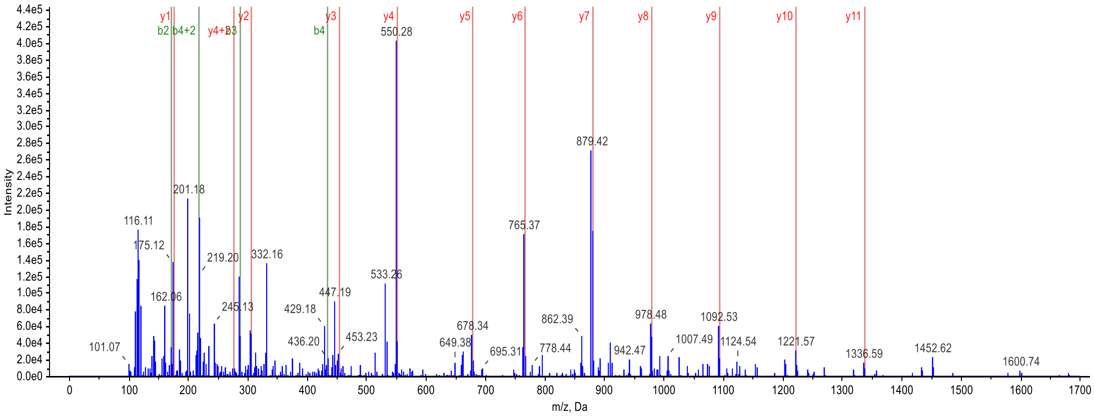

18.2 VT[Pho]PQPGVPPEEAGAAESSTGTWTTVWTDGLTSLDR

Residue	b	b+2	y	y+2
V	404.2810	202.6442	3997.9010	1999.4541
T[Pho]	585.2951	293.1512	3594.6272	1797.8172
P	682.3478	341.6775	3413.6132	1707.3102
Q	810.4064	405.7068	3316.5604	1658.7838
P	907.4592	454.2332	3188.5018	1594.7546
G	964.4806	482.7440	3091.4491	1546.2282
V	1063.5490	532.2782	3034.4276	1517.7174
P	1160.6018	580.8045	2935.3592	1468.1832
P	1257.6546	629.3309	2838.3064	1419.6569
E	1386.6972	693.8522	2741.2537	1371.1305
E	1515.7398	758.3735	2612.2111	1306.6092
A	1586.7769	793.8921	2483.1685	1242.0879
G	1643.7983	822.4028	2412.1314	1206.5693
A	1714.8354	857.9214	2355.1099	1178.0586
A	1785.8726	893.4399	2284.0728	1142.5400
E	1914.9152	957.9612	2213.0357	1107.0215
S	2001.9472	1001.4772	2083.9931	1042.5002

Residue	b	b+2	y	y+2
S	2088.9792	1044.9932	1996.9611	998.9842
T	2190.0269	1095.5171	1909.9290	955.4682
G	2247.0484	1124.0278	1808.8814	904.9443
T	2348.0960	1174.5517	1751.8599	876.4336
W	2534.1753	1267.5913	1650.8122	825.9097
T	2635.2230	1318.1151	1464.7329	732.8701
T	2736.2707	1368.6390	1363.6852	682.3462
V	2835.3391	1418.1732	1262.6375	631.8224
W	3021.4184	1511.2129	1163.5691	582.2882
T	3122.4661	1561.7367	977.4898	489.2485
D	3237.4930	1619.2502	876.4421	438.7247
G	3294.5145	1647.7609	761.4152	381.2112
L	3407.5986	1704.3029	704.3937	352.7005
T	3508.6463	1754.8268	591.3097	296.1585
S	3595.6783	1798.3428	490.2620	245.6346
L	3708.7623	1854.8848	403.2300	202.1186
D	3823.7893	1912.3983	290.1459	145.5766
R	3979.8904	1990.4488	175.1190	88.0631

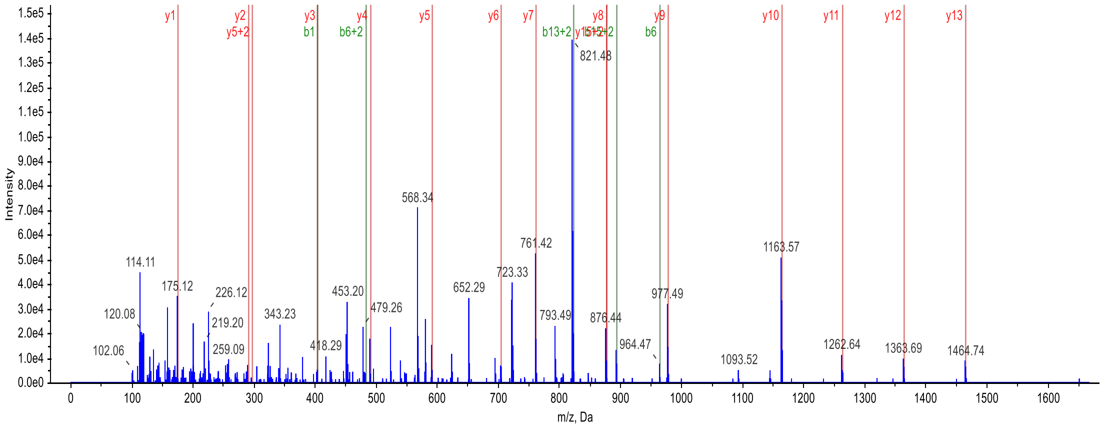

19 Accession No. CAB85674/gi|7414457

19.1 AAFRVTPRPGVPPEEAGAAVAAESS[Pho]TGTWTTVWTDGLTSLDR

Residue	b	b+2	y	y+2
A	114.0550	57.5311	4450.1351	2225.5712
A	185.0921	93.0497	4337.0874	2169.0473
F	332.1605	166.5839	4266.0503	2133.5288
R	488.2616	244.6344	4118.9819	2059.9946
V	587.3300	294.1686	3962.8808	1981.9440
T	688.3777	344.6925	3863.8124	1932.4098
P	785.4304	393.2189	3762.7647	1881.8860
R	941.5316	471.2694	3665.7119	1833.3596
P	1038.5843	519.7958	3509.6108	1755.3090
G	1095.6058	548.3065	3412.5581	1706.7827
V	1194.6742	597.8407	3355.5366	1678.2719
P	1291.7270	646.3671	3256.4682	1628.7377
P	1388.7797	694.8935	3159.4154	1580.2113
E	1517.8223	759.4148	3062.3626	1531.6850
E	1646.8649	823.9361	2933.3201	1467.1637
A	1717.9020	859.4547	2804.2775	1402.6424
G	1774.9235	887.9654	2733.2403	1367.1238
A	1845.9606	923.4839	2676.2189	1338.6131
A	1916.9977	959.0025	2605.1818	1303.0945
V	2016.0661	1008.5367	2534.1447	1267.5760
A	2087.1032	1044.0553	2435.0762	1218.0418

Residue	b	b+2	y	y+2
A	2158.1404	1079.5738	2364.0391	1182.5232
E	2287.1830	1144.0951	2293.0020	1147.0046
S	2374.2150	1187.6111	2163.9594	1082.4833
S[Pho]	2541.2133	1271.1103	2076.9274	1038.9673
T	2642.2610	1321.6341	1909.9290	955.4682
G	2699.2825	1350.1449	1808.8814	904.9443
T	2800.3302	1400.6687	1751.8599	876.4336
W	2986.4095	1493.7084	1650.8122	825.9097
T	3087.4572	1544.2322	1464.7329	732.8701
T	3188.5048	1594.7561	1363.6852	682.3462
V	3287.5732	1644.2903	1262.6375	631.8224
W	3473.6526	1737.3299	1163.5691	582.2882
T	3574.7002	1787.8538	977.4898	489.2485
D	3689.7272	1845.3672	876.4421	438.7247
G	3746.7486	1873.8780	761.4152	381.2112
L	3859.8327	1930.4200	704.3937	352.7005
T	3960.8804	1980.9438	591.3097	296.1585
S	4047.9124	2024.4598	490.2620	245.6346
L	4160.9965	2081.0019	403.2300	202.1186
D	4276.0234	2138.5154	290.1459	145.5766
R	4432.1245	2216.5659	175.1190	88.0631

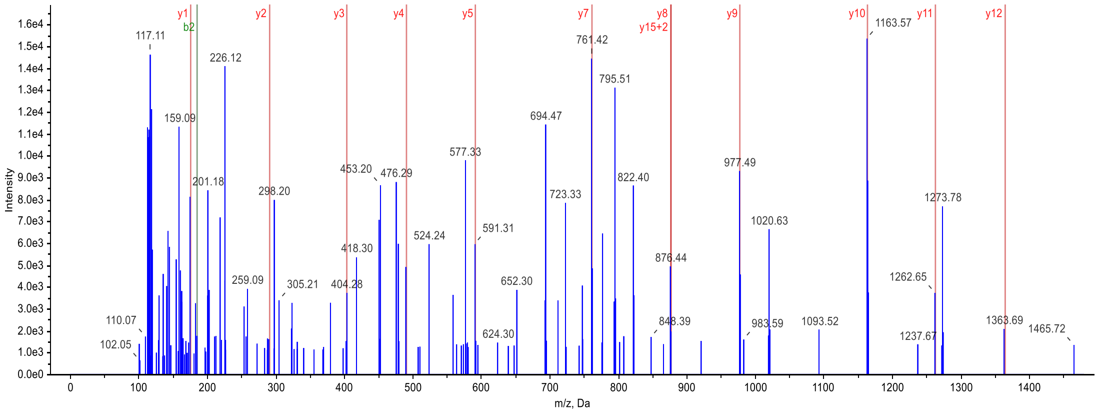

20 Accession No. CAA94018/gi|1771824

20.1 PGVPPEEAGAEVAAESS[Pho]TGTWTTVWTDGLTSLDR

Residue	b	b+2	y	y+2
P	402.2654	201.6363	3871.8217	1936.4145
G	459.2869	230.1471	3470.5635	1735.7854
V	558.3553	279.6813	3413.5421	1707.2747
P	655.4080	328.2077	3314.4737	1657.7405
P	752.4608	376.7340	3217.4209	1609.2141
E	881.5034	441.2553	3120.3681	1560.6877
E	1010.5460	505.7766	2991.3255	1496.1664
A	1081.5831	541.2952	2862.2829	1431.6451
G	1138.6046	569.8059	2791.2458	1396.1266
A	1209.6417	605.3245	2734.2244	1367.6158
E	1338.6843	669.8458	2663.1872	1332.0973
V	1437.7527	719.3800	2534.1447	1267.5760
A	1508.7898	754.8985	2435.0762	1218.0418
A	1579.8269	790.4171	2364.0391	1182.5232
E	1708.8695	854.9384	2293.0020	1147.0046
S	1795.9015	898.4544	2163.9594	1082.4833
S[Pho]	1962.8999	981.9536	2076.9274	1038.9673

Residue	b	b+2	y	y+2
T	2063.9476	1032.4774	1909.9290	955.4682
G	2120.9690	1060.9882	1808.8814	904.9443
T	2222.0167	1111.5120	1751.8599	876.4336
W	2408.0960	1204.5517	1650.8122	825.9097
T	2509.1437	1255.0755	1464.7329	732.8701
T	2610.1914	1305.5993	1363.6852	682.3462
V	2709.2598	1355.1335	1262.6375	631.8224
W	2895.3391	1448.1732	1163.5691	582.2882
T	2996.3868	1498.6970	977.4898	489.2485
D	3111.4137	1556.2105	876.4421	438.7247
G	3168.4352	1584.7212	761.4152	381.2112
L	3281.5193	1641.2633	704.3937	352.7005
T	3382.5669	1691.7871	591.3097	296.1585
S	3469.5990	1735.3031	490.2620	245.6346
L	3582.6830	1791.8452	403.2300	202.1186
D	3697.7100	1849.3586	290.1459	145.5766
R	3853.8111	1927.4092	175.1190	88.0631

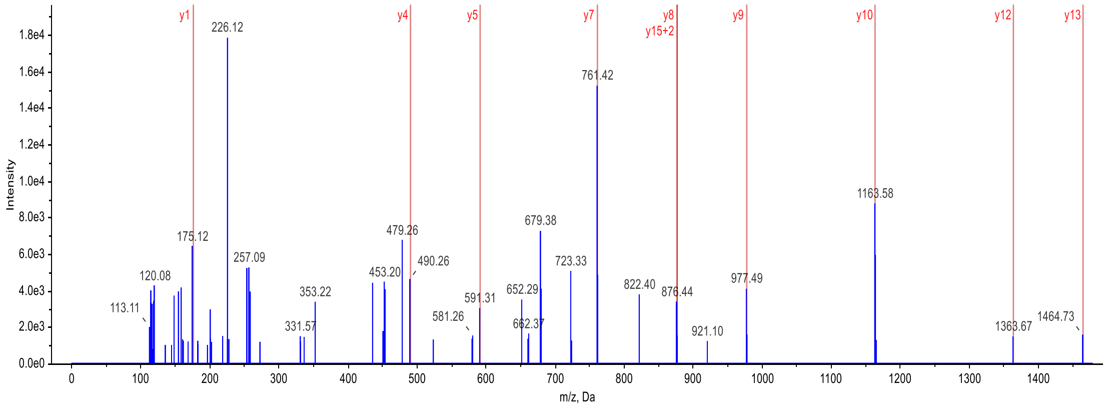

21 Accession No. CAC04358/gi|9909841

21.1 VTPQPGVPAEEAGAAVDAES[Pho]STGTWTTVWTDGLTSLDR

Residue	b	b+2	y	y+2
V	404.2810	202.6442	4577.2181	2289.1127
T	505.3287	253.1680	4173.9443	2087.4758
P	602.3815	301.6944	4072.8966	2036.9519
Q	730.4401	365.7237	3975.8438	1988.4256
P	827.4928	414.2501	3847.7853	1924.3963
G	884.5143	442.7608	3750.7325	1875.8699
V	983.5827	492.2950	3693.7110	1847.3592
P[Oxi]	1096.6304	548.8188	3594.6426	1797.8250
A	1167.6675	584.3374	3481.5949	1741.3011
E	1296.7101	648.8587	3410.5578	1705.7826
E	1425.7527	713.3800	3281.5152	1641.2613
A	1496.7898	748.8985	3152.4726	1576.7400
G	1553.8113	777.4093	3081.4355	1541.2214
A	1624.8484	812.9278	3024.4141	1512.7107
A	1695.8855	848.4464	2953.3770	1477.1921
V	1794.9539	897.9806	2882.3398	1441.6736
D	1909.9809	955.4941	2783.2714	1392.1394
A	1981.0180	991.0126	2668.2445	1334.6259
E	2110.0606	1055.5339	2597.2074	1299.1073

Residue	b	b+2	y	y+2
S[Pho]	2277.0589	1139.0331	2468.1648	1234.5860
S[IT8]	2668.2963	1334.6518	2301.1664	1151.0868
T	2769.3440	1385.1756	1909.9290	955.4682
G	2826.3654	1413.6864	1808.8814	904.9443
T	2927.4131	1464.2102	1751.8599	876.4336
W	3113.4924	1557.2499	1650.8122	825.9097
T	3214.5401	1607.7737	1464.7329	732.8701
T	3315.5878	1658.2975	1363.6852	682.3462
V	3414.6562	1707.8317	1262.6375	631.8224
W	3600.7355	1800.8714	1163.5691	582.2882
T	3701.7832	1851.3952	977.4898	489.2485
D	3816.8101	1908.9087	876.4421	438.7247
G	3873.8316	1937.4194	761.4152	381.2112
L	3986.9157	1993.9615	704.3937	352.7005
T	4087.9634	2044.4853	591.3097	296.1585
S	4174.9954	2088.0013	490.2620	245.6346
L	4288.0794	2144.5434	403.2300	202.1186
D	4403.1064	2202.0568	290.1459	145.5766
R	4559.2075	2280.1074	175.1190	88.0631

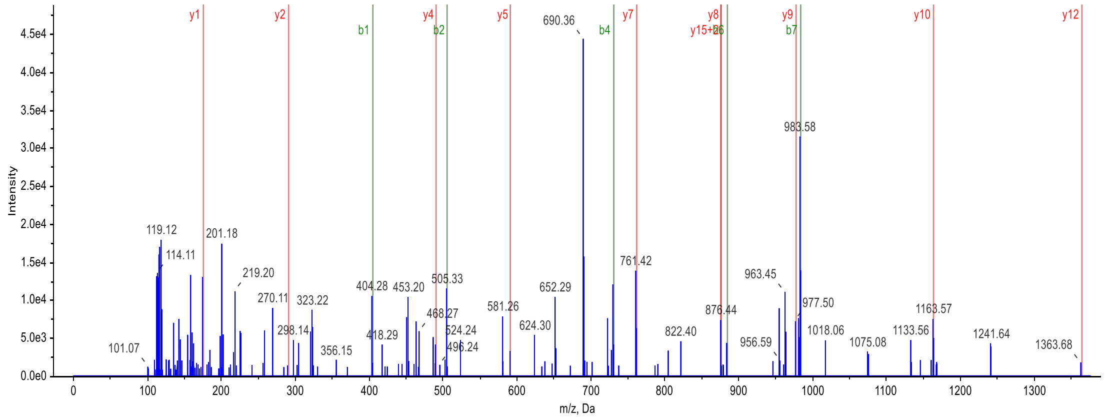

22 Accession No. AAG43944/gi|12004215

22.1 VT[Pho]PQPGVPPGGAGAAVAAESS[Pho]TGTWTTVWTDGLTSLDR

Residue	b	b+2	y	y+2
V	100.0757	50.5415	3870.7623	1935.8848
T[Pho]	281.0897	141.0485	3771.6939	1886.3506
P	378.1425	189.5749	3590.6799	1795.8436
Q	506.2010	253.6042	3493.6271	1747.3172
P	603.2538	302.1305	3365.5686	1683.2879
G	660.2753	330.6413	3268.5158	1634.7615
V	759.3437	380.1755	3211.4943	1606.2508
P	856.3964	428.7019	3112.4259	1556.7166
P	953.4492	477.2282	3015.3732	1508.1902
G	1010.4707	505.7390	2918.3204	1459.6638
G	1067.4921	534.2497	2861.2989	1431.1531
A	1138.5293	569.7683	2804.2775	1402.6424
G	1195.5507	598.2790	2733.2403	1367.1238
A	1266.5878	633.7976	2676.2189	1338.6131
A	1337.6249	669.3161	2605.1818	1303.0945
V	1436.6934	718.8503	2534.1447	1267.5760
A	1507.7305	754.3689	2435.0762	1218.0418
A	1578.7676	789.8874	2364.0391	1182.5232
E	1707.8102	854.4087	2293.0020	1147.0046

Residue	b	b+2	y	y+2
S	1794.8422	897.9247	2163.9594	1082.4833
S[Pho]	1961.8406	981.4239	2076.9274	1038.9673
T	2062.8882	1031.9478	1909.9290	955.4682
G	2119.9097	1060.4585	1808.8814	904.9443
T	2220.9574	1110.9823	1751.8599	**876.4336**
W	2407.0367	1204.0220	1650.8122	825.9097
T	2508.0844	1254.5458	**1464.7329**	732.8701
T	2609.1321	1305.0697	1363.6852	682.3462
V	2708.2005	1354.6039	1262.6375	631.8224
W	2894.2798	1447.6435	**1163.5691**	582.2882
T	2995.3275	1498.1674	**977.4898**	489.2485
D	3110.3544	1555.6808	**876.4421**	438.7247
G	3167.3759	1584.1916	**761.4152**	381.2112
L	3280.4599	1640.7336	704.3937	352.7005
T	3381.5076	1691.2574	**591.3097**	296.1585
S	3468.5396	1734.7735	**490.2620**	245.6346
L	3581.6237	1791.3155	403.2300	202.1186
D	3696.6506	1848.8290	**290.1459**	145.5766
R	3852.7518	1926.8795	**175.1190**	88.0631

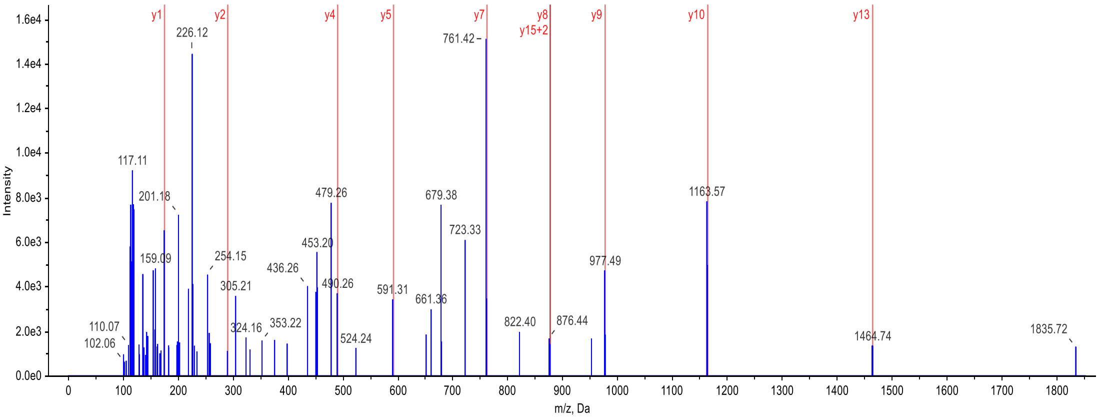

22.2 VTPQPGVPPGGAGAAVAAES[Pho]S[Pho]TGTWTTVWTDGLTSLDR

Residue	b	b+2	y	y+2
V	**404.2810**	202.6442	4174.9677	2087.9875
T	**505.3287**	253.1680	3771.6939	1886.3506
P	**602.3815**	301.6944	3670.6462	1835.8268
Q	**730.4401**	**365.7237**	3573.5935	1787.3004
P	827.4928	**414.2501**	3445.5349	1723.2711
G	**884.5143**	**442.7608**	3348.4821	1674.7447
V	**983.5827**	**492.2950**	3291.4607	1646.2340
P	**1080.6355**	540.8214	3192.3922	1596.6998
P	1177.6882	**589.3478**	3095.3395	1548.1734
G	1234.7097	617.8585	2998.2867	1499.6470
G	1291.7312	646.3692	2941.2653	1471.1363
A	1362.7683	681.8878	2884.2438	1442.6255
G	1419.7897	710.3985	2813.2067	1407.1070
A	1490.8269	745.9171	2756.1852	1378.5962
A	1561.8640	781.4356	2685.1481	1343.0777
V	1660.9324	830.9698	2614.1110	1307.5591
A	1731.9695	866.4884	2515.0426	1258.0249
A	1803.0066	902.0069	2444.0055	1222.5064
E	1932.0492	966.5282	2372.9683	1186.9878

Residue	b	b+2	y	y+2
S[Pho]	2099.0476	1050.0274	2243.9258	1122.4665
S[Pho]	2266.0459	1133.5266	2076.9274	1038.9673
T	2367.0936	1184.0504	1909.9290	955.4682
G	2424.1151	1212.5612	1808.8814	904.9443
T	2525.1627	1263.0850	1751.8599	**876.4336**
W	2711.2421	1356.1247	**1650.8122**	825.9097
T	2812.2897	1406.6485	**1464.7329**	732.8701
T	2913.3374	1457.1723	**1363.6852**	682.3462
V	3012.4058	1506.7066	**1262.6375**	631.8224
W	3198.4851	1599.7462	**1163.5691**	582.2882
T	3299.5328	1650.2700	**977.4898**	489.2485
D	3414.5598	1707.7835	**876.4421**	438.7247
G	3471.5812	1736.2943	**761.4152**	381.2112
L	3584.6653	1792.8363	**704.3937**	352.7005
T	3685.7130	1843.3601	**591.3097**	**296.1585**
S	3772.7450	1886.8761	**490.2620**	245.6346
L	3885.8291	1943.4182	**403.2300**	**202.1186**
D	4000.8560	2000.9316	**290.1459**	145.5766
R	4156.9571	2078.9822	**175.1190**	88.0631

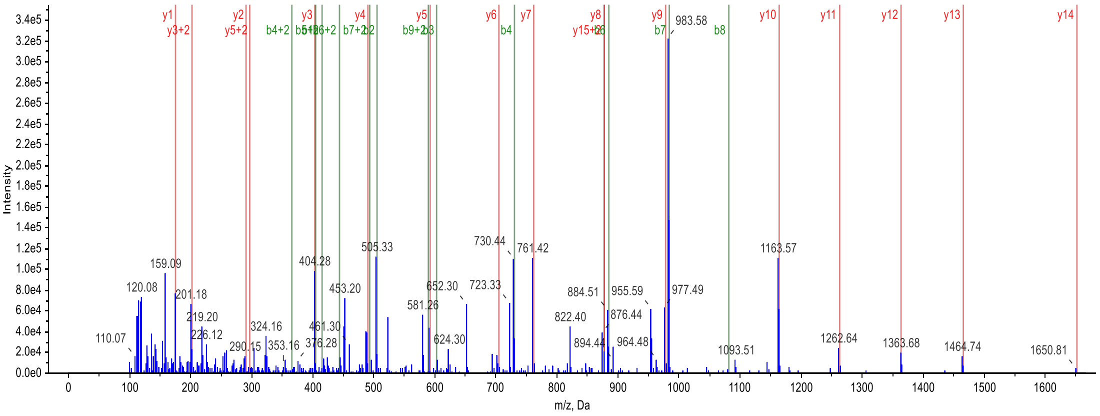

23 Accession No. CAA93205/gi|1518392

23.1 VTPQPGVPAEEAGAAVAAES[Pho]STGTWT[Pho]TVWTDGLTSLDR

Residue	b	b+2	y	y+2
V	**404.2810**	202.6442	4597.1996	2299.1035
T	**505.3287**	253.1680	4193.9259	2097.4666
P	602.3815	301.6944	4092.8782	2046.9427
Q	**730.4401**	365.7237	3995.8254	1998.4164
P	827.4928	414.2501	3867.7669	1934.3871
G	**884.5143**	442.7608	3770.7141	1885.8607
V	**983.5827**	492.2950	3713.6926	1857.3500
P	1080.6355	540.8214	3614.6242	1807.8157
A	1151.6726	576.3399	3517.5714	1759.2894
E	1280.7152	640.8612	3446.5343	1723.7708
E	1409.7578	705.3825	3317.4917	1659.2495
A	1480.7949	740.9011	3188.4491	1594.7282
G	1537.8164	769.4118	3117.4120	1559.2097
A	1608.8535	804.9304	3060.3906	1530.6989
A	1679.8906	840.4489	2989.3535	1495.1804
V	1778.9590	889.9831	2918.3163	1459.6618
A	1849.9961	925.5017	2819.2479	1410.1276
A	1921.0332	961.0202	2748.2108	1374.6090
E	2050.0758	1025.5415	2677.1737	1339.0905

Residue	b	b+2	y	y+2
S[Pho]	2217.0742	1109.0407	2548.1311	1274.5692
S[IT8]	2608.3116	1304.6594	2381.1328	1191.0700
T	2709.3592	1355.1833	1989.8954	995.4513
G	2766.3807	1383.6940	1888.8477	944.9275
T	2867.4284	1434.2178	1831.8262	916.4167
W	3053.5077	1527.2575	1730.7785	865.8929
T[Pho]	3234.5217	1617.7645	1544.6992	772.8533
T	3335.5694	1668.2883	**1363.6852**	682.3462
V	3434.6378	1717.8225	**1262.6375**	631.8224
W	3620.7171	1810.8622	**1163.5691**	582.2882
T	3721.7648	1861.3860	**977.4898**	489.2485
D	3836.7917	1918.8995	**876.4421**	438.7247
G	3893.8132	1947.4102	**761.4152**	381.2112
L	4006.8973	2003.9523	704.3937	352.7005
T	4107.9449	2054.4761	**591.3097**	296.1585
S	4194.9770	2097.9921	**490.2620**	245.6346
L	4308.0610	2154.5342	403.2300	202.1186
D	4423.0880	2212.0476	**290.1459**	145.5766
R	4579.1891	2290.0982	**175.1190**	88.0631

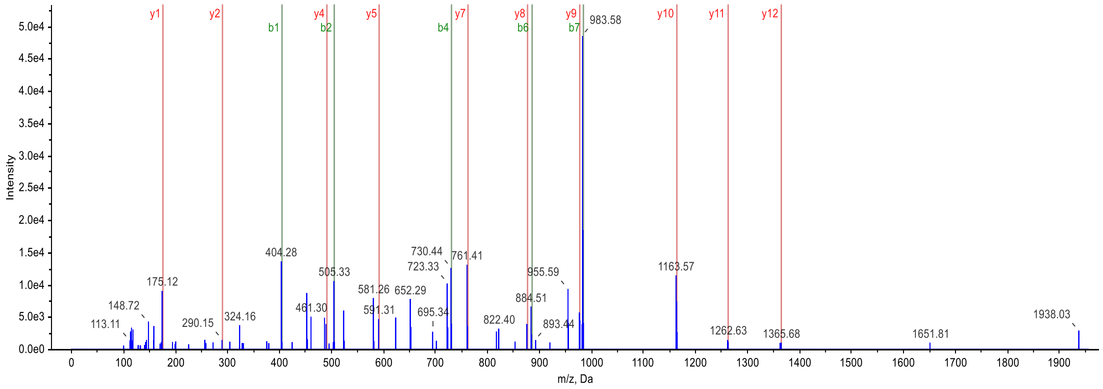

23.2 VTPQPGVPAEEAGAAVAAESS[Pho]TGTWTTVWTDGLTSLDR

Residue	b	b+2	y	y+2
V	**404.2810**	202.6442	4195.0174	2098.0123
T	**505.3287**	253.1680	3791.7436	1896.3754
P	602.3815	301.6944	3690.6959	1845.8516
Q	**730.4401**	**365.7237**	3593.6432	1797.3252
P	**827.4928**	414.2501	3465.5846	1733.2959
G	**884.5143**	**442.7608**	3368.5318	1684.7696
V	**983.5827**	492.2950	3311.5104	1656.2588
P	1080.6355	540.8214	3212.4420	1606.7246
A	1151.6726	576.3399	3115.3892	1558.1982
E	1280.7152	640.8612	3044.3521	1522.6797
E	1409.7578	705.3825	2915.3095	1458.1584
A	1480.7949	740.9011	2786.2669	1393.6371
G	1537.8164	769.4118	2715.2298	1358.1185
A	1608.8535	804.9304	2658.2083	1329.6078
A	1679.8906	840.4489	2587.1712	1294.0892
V	1778.9590	889.9831	2516.1341	1258.5707
A	1849.9961	925.5017	2417.0657	1209.0365
A	1921.0332	961.0202	2346.0286	1173.5179
E	2050.0758	1025.5415	2274.9914	1137.9994

Residue	b	b+2	y	y+2
S	2137.1078	1069.0576	2145.9489	1073.4781
S[Pho]	2304.1062	1152.5567	2058.9168	1029.9621
T	2405.1539	1203.0806	1891.9185	946.4629
G	2462.1753	1231.5913	1790.8708	895.9390
T[Dhy]	2545.2125	1273.1099	1733.8493	867.4283
W	2731.2918	1366.1495	1650.8122	825.9097
T	2832.3394	1416.6734	**1464.7329**	732.8701
T	2933.3871	1467.1972	**1363.6852**	682.3462
V	3032.4555	1516.7314	**1262.6375**	631.8224
W	3218.5349	1609.7711	**1163.5691**	582.2882
T	3319.5825	1660.2949	**977.4898**	489.2485
D	3434.6095	1717.8084	**876.4421**	438.7247
G	3491.6309	1746.3191	**761.4152**	381.2112
L	3604.7150	1802.8611	**704.3937**	352.7005
T	3705.7627	1853.3850	**591.3097**	**296.1585**
S	3792.7947	1896.9010	**490.2620**	245.6346
L	3905.8788	1953.4430	**403.2300**	202.1186
D	4020.9057	2010.9565	**290.1459**	145.5766
R	4177.0068	2089.0071	**175.1190**	88.0631

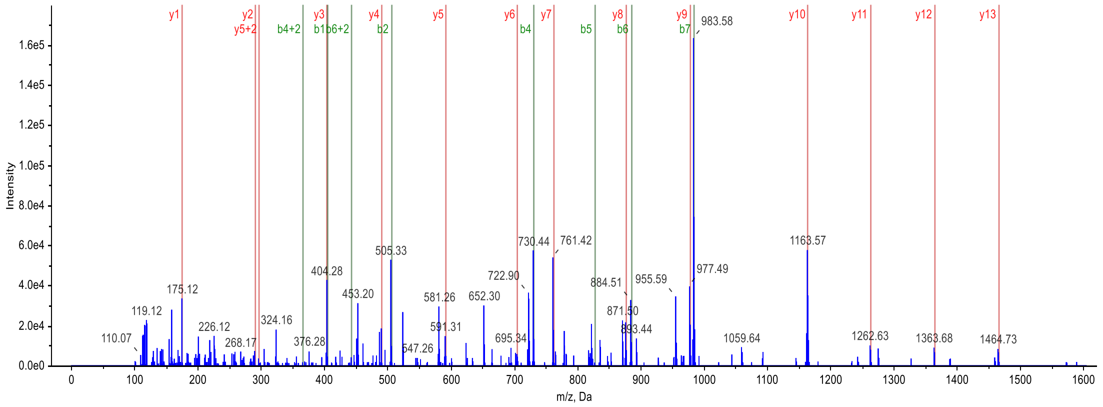

24 Accession No. AFA27686/gi|374975191

24.1 VTPQPGVPPEEAGAAVAGEIGT[Pho]WT[Pho]TVWTDGLTSLDR

Residue	b	b+2	y	y+2
V	**404.2810**	202.6442	4158.9615	2079.9844
T	**505.3287**	253.1680	3755.6878	1878.3475
P	602.3815	301.6944	3654.6401	1827.8237
Q	**730.4401**	365.7237	3557.5873	1779.2973
P	827.4928	414.2501	3429.5287	1715.2680
G	**884.5143**	**442.7608**	3332.4760	1666.7416
V	**983.5827**	**492.2950**	3275.4545	1638.2309
P	1080.6355	540.8214	3176.3861	1588.6967
P[Oxi]	1193.6832	597.3452	3079.3333	1540.1703
E	1322.7257	661.8665	2966.2857	1483.6465
E	1451.7683	726.3878	2837.2431	1419.1252
A	1522.8055	761.9064	2708.2005	1354.6039
G	1579.8269	790.4171	2637.1634	1319.0853
A	1650.8640	825.9357	2580.1419	1290.5746
A	1721.9011	861.4542	2509.1048	1255.0560
V	1820.9696	910.9884	2438.0677	1219.5375
A	1892.0067	946.5070	2338.9992	1170.0033
G	1949.0281	975.0177	2267.9621	1134.4847

Residue	b	b+2	y	y+2
E	2078.0707	1039.5390	2210.9407	1105.9740
I	2191.1548	1096.0810	2081.8981	1041.4527
G	2248.1763	1124.5918	1968.8140	984.9106
T[Pho]	2429.1903	1215.0988	1911.7926	956.3999
W	2615.2696	1308.1384	1730.7785	865.8929
T[Pho]	2796.2836	1398.6454	1544.6992	772.8533
T	2897.3313	1449.1693	**1363.6852**	682.3462
V	2996.3997	1498.7035	**1262.6375**	631.8224
W	3182.4790	1591.7431	**1163.5691**	582.2882
T	3283.5267	1642.2670	**977.4898**	489.2485
D	3398.5536	1699.7804	**876.4421**	438.7247
G	3455.5751	1728.2912	**761.4152**	381.2112
L	3568.6591	1784.8332	**704.3937**	352.7005
T	3669.7068	1835.3570	**591.3097**	**296.1585**
S	3756.7388	1878.8731	**490.2620**	245.6346
L	3869.8229	1935.4151	**403.2300**	202.1186
D	3984.8499	1992.9286	**290.1459**	145.5766
R	4140.9510	2070.9791	**175.1190**	88.0631

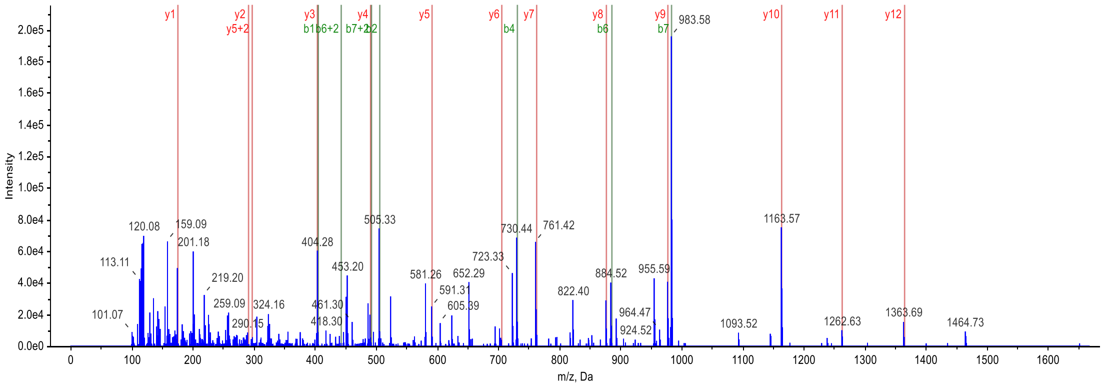

24.2 VTPQPGVPPEEAGAAVAGEIGTWT[Pho]T[Pho]VWTDGLTSLDR

Residue	b	b+2	y	y+2
V	**404.2810**	202.6442	4158.9615	2079.9844
T	**505.3287**	253.1680	3755.6878	1878.3475
P	602.3815	301.6944	3654.6401	1827.8237
Q	**730.4401**	365.7237	3557.5873	1779.2973
P	**827.4928**	414.2501	3429.5287	1715.2680
G	**884.5143**	**442.7608**	3332.4760	1666.7416
V	**983.5827**	492.2950	3275.4545	1638.2309
P	**1080.6355**	540.8214	3176.3861	1588.6967
P[Oxi]	1193.6832	597.3452	3079.3333	1540.1703
E	1322.7257	661.8665	2966.2857	1483.6465
E	1451.7683	726.3878	2837.2431	1419.1252
A	1522.8055	761.9064	2708.2005	1354.6039
G	1579.8269	790.4171	2637.1634	1319.0853
A	1650.8640	825.9357	2580.1419	1290.5746
A	1721.9011	861.4542	2509.1048	1255.0560
V	1820.9696	910.9884	2438.0677	1219.5375
A	1892.0067	946.5070	2338.9992	1170.0033
G	1949.0281	975.0177	2267.9621	1134.4847

Residue	b	b+2	y	y+2
E	2078.0707	1039.5390	2210.9407	1105.9740
I	2191.1548	1096.0810	2081.8981	1041.4527
G	2248.1763	1124.5918	1968.8140	984.9106
T	2349.2239	1175.1156	1911.7926	956.3999
W	2535.3032	1268.1553	1810.7449	905.8761
T[Pho]	2716.3173	1358.6623	1624.6656	812.8364
T[Pho]	2897.3313	1449.1693	1443.6516	722.3294
V	2996.3997	1498.7035	**1262.6375**	631.8224
W	3182.4790	1591.7431	**1163.5691**	582.2882
T	3283.5267	1642.2670	**977.4898**	489.2485
D	3398.5536	1699.7804	**876.4421**	438.7247
G	3455.5751	1728.2912	**761.4152**	381.2112
L	3568.6591	1784.8332	**704.3937**	352.7005
T	3669.7068	1835.3570	**591.3097**	**296.1585**
S	3756.7388	1878.8731	**490.2620**	245.6346
L	3869.8229	1935.4151	**403.2300**	202.1186
D	3984.8499	1992.9286	**290.1459**	145.5766
R	4140.9510	2070.9791	**175.1190**	88.0631

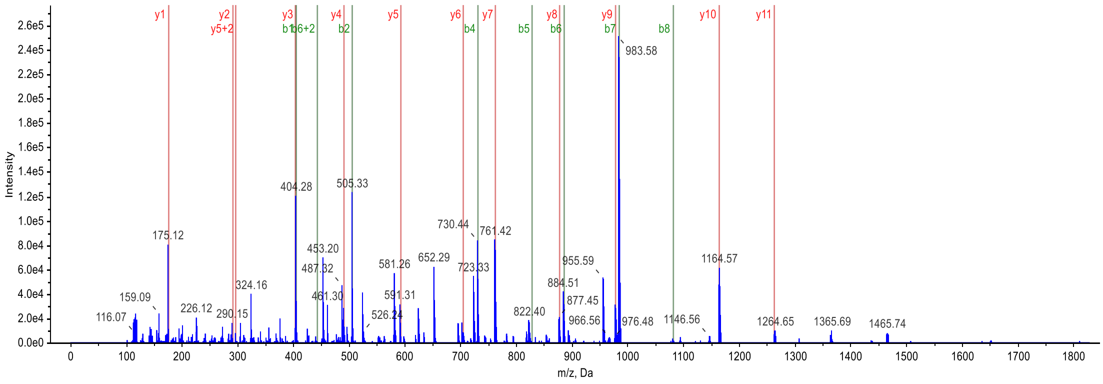

25 Accession No. EMT31124/gi|475620495

25.1 GDSS[Pho]PLEVIAINDTGGVK

Residue	b	b+2	y	y+2
G	362.2341	181.6207	2460.2843	1230.6458
D	477.2610	239.1342	2099.0575	1050.0324
S	564.2931	282.6502	1984.0305	992.5189
S[Pho]	731.2914	366.1494	1896.9985	949.0029
P	828.3442	414.6757	1730.0001	865.5037
L	941.4283	471.2178	1632.9474	816.9773
E	1070.4709	535.7391	1519.8633	760.4353
V	1169.5393	585.2733	1390.8207	695.9140
I	1282.6233	641.8153	1291.7523	646.3798
A	1353.6604	677.3339	1178.6682	589.8378
I	1466.7445	733.8759	1107.6311	554.3192
N	1580.7874	790.8974	994.5471	497.7772
D	1695.8144	848.4108	880.5041	440.7557
T	1796.8621	898.9347	765.4772	383.2422
G	1853.8835	927.4454	664.4295	332.7184
G	1910.9050	955.9561	607.4080	304.2077
V	2009.9734	1005.4903	550.3866	275.6969
K[IT8]	2442.2737	1221.6405	451.3182	226.1627

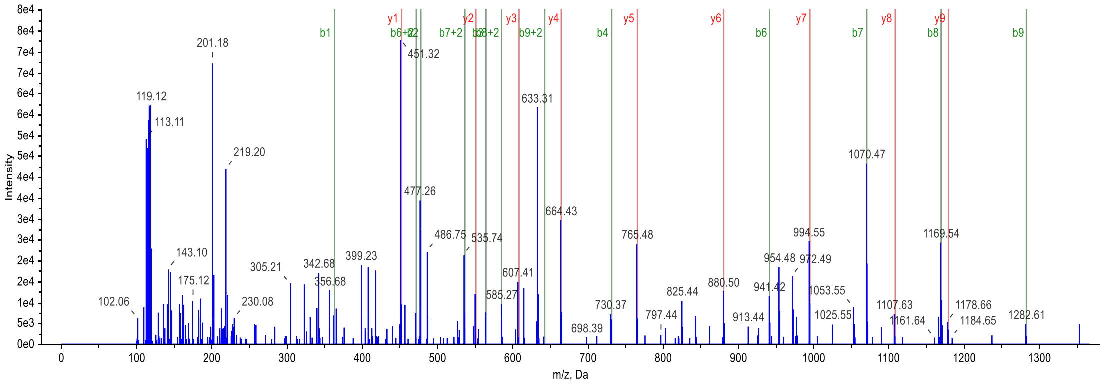

26 Accession No. XP_003568189/gi|357133147

26.1 PGVVALDEAVTVGSVT[Pho]

Residue	b	b+2	y	y+2
P	98.0600	49.5337	1593.7771	797.3922
G	155.0815	78.0444	1496.7244	748.8658
V	254.1499	127.5786	1439.7029	720.3551
V	353.2183	177.1128	1340.6345	670.8209
A	424.2554	212.6314	1241.5661	621.2867
L	537.3395	269.1734	1170.5290	585.7681
D	652.3665	326.6869	1057.4449	529.2261
E	781.4090	391.2082	942.4180	471.7126
A	852.4462	426.7267	813.3754	407.1913
V	951.5146	476.2609	742.3383	371.6728
T	1052.5623	526.7848	643.2698	322.1386
V	1151.6307	576.3190	542.2222	271.6147
G	1208.6521	604.8297	443.1538	222.0805
S	1295.6842	648.3457	386.1323	193.5698
V	1394.7526	697.8799	299.1003	150.0538
T[Pho]	1575.7666	788.3869	200.0319	100.5196

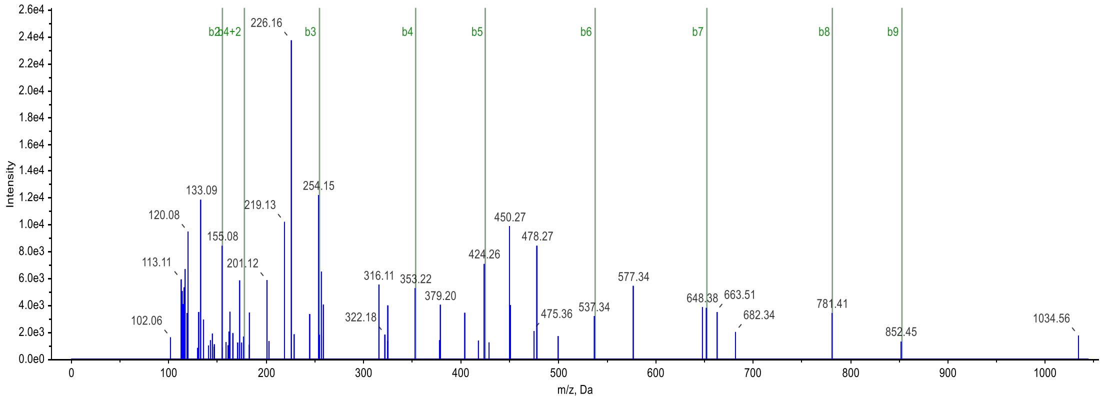

27 Accession No. EMS64844/gi|474384779

27.1 NPLIAAAS[Pho]VIAAGLAVGLAS[Pho]IGPGVGQGTAAGQAVEGIAR

Residue	b	b+2	y	y+2
N	115.0502	58.0287	3729.8976	1865.4525
P	**212.1030**	106.5551	3615.8547	1808.4310
L	**325.1870**	163.0972	3518.8019	1759.9046
I	**438.2711**	219.6392	3405.7179	1703.3626
A	509.3082	255.1577	3292.6338	1646.8205
A	**580.3453**	290.6763	3221.5967	1611.3020
A	**651.3824**	326.1949	3150.5596	1575.7834
S[Pho]	818.3808	409.6940	3079.5225	1540.2649
V	917.4492	459.2282	2912.5241	1456.7657
I	1030.5333	515.7703	2813.4557	1407.2315
A	1101.5704	551.2888	2700.3716	1350.6895
A	1172.6075	586.8074	2629.3345	1315.1709
G	1229.6290	615.3181	2558.2974	1279.6523
L	1342.7130	671.8602	2501.2759	1251.1416
A	1413.7501	707.3787	2388.1919	1194.5996
V	1512.8186	756.9129	2317.1548	1159.0810
G	1569.8400	785.4236	2218.0864	1109.5468
L	1682.9241	841.9657	2161.0649	1081.0361
A	1753.9612	877.4842	2047.9808	1024.4941
S[Pho]	1920.9596	960.9834	1976.9437	988.9755

Residue	b	b+2	y	y+2
I	2034.0436	1017.5254	1809.9454	905.4763
G	2091.0651	1046.0362	**1696.8613**	**848.9343**
P	2188.1178	1094.5626	**1639.8398**	**820.4236**
G	2245.1393	1123.0733	1542.7871	771.8972
V	2344.2077	1172.6075	1485.7656	743.3864
G	2401.2292	1201.1182	1386.6972	693.8522
Q[Dea]	2530.2718	1265.6395	1329.6757	665.3415
G	2587.2932	1294.1503	**1200.6331**	600.8202
T	2688.3409	1344.6741	**1143.6117**	572.3095
A	2759.3780	1380.1927	**1042.5640**	521.7856
A	2830.4152	1415.7112	**971.5269**	486.2671
G	2887.4366	1444.2219	**900.4898**	450.7485
Q	3015.4952	1508.2512	843.4683	422.2378
A	3086.5323	1543.7698	**715.4097**	358.2085
V	3185.6007	1593.3040	**644.3726**	322.6899
E	3314.6433	1657.8253	**545.3042**	273.1557
G	3371.6648	1686.3360	**416.2616**	208.6344
I	3484.7488	1742.8781	359.2401	180.1237
A	3555.7860	1778.3966	**246.1561**	123.5817
R	3711.8871	1856.4472	**175.1190**	88.0631

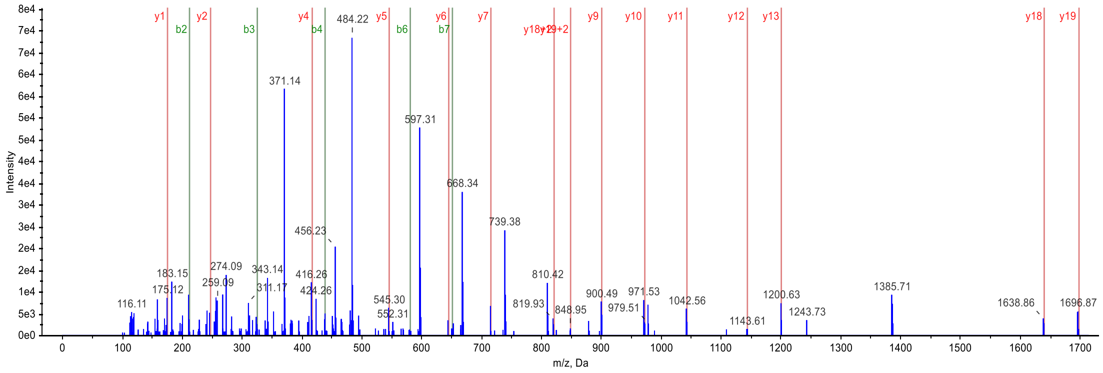

28 Accession No. AEJ10071/gi|338825698

28.1 T[Pho]GLGQVMSGELVEFAEGTR

Residue	b	b+2	y	y+2
T[Pho]	486.2266	243.6170	2379.1569	1190.0821
G	543.2481	272.1277	1893.9375	947.4724
L	656.3322	328.6697	1836.9160	918.9617
G	713.3536	**357.1805**	1723.8320	862.4196
Q[1Me]	855.4279	428.2176	1666.8105	833.9089
V	954.4963	477.7518	1524.7363	762.8718
M	1085.5368	543.2720	**1425.6679**	713.3376
S	1172.5688	586.7880	**1294.6274**	647.8173
G	1229.5903	615.2988	**1207.5953**	604.3013
E	1358.6328	679.8201	**1150.5739**	575.7906
L	1471.7169	736.3621	**1021.5313**	511.2693
V	1570.7853	785.8963	**908.4472**	454.7272
E	1699.8279	850.4176	**809.3788**	405.1930
F	1846.8963	923.9518	**680.3362**	340.6717
A	1917.9334	959.4704	**533.2678**	267.1375
E	2046.9760	1023.9917	**462.2307**	231.6190
G	2103.9975	1052.5024	**333.1881**	167.0977
T	2205.0452	1103.0262	276.1666	138.5870
R	2361.1463	1181.0768	**175.1190**	88.0631

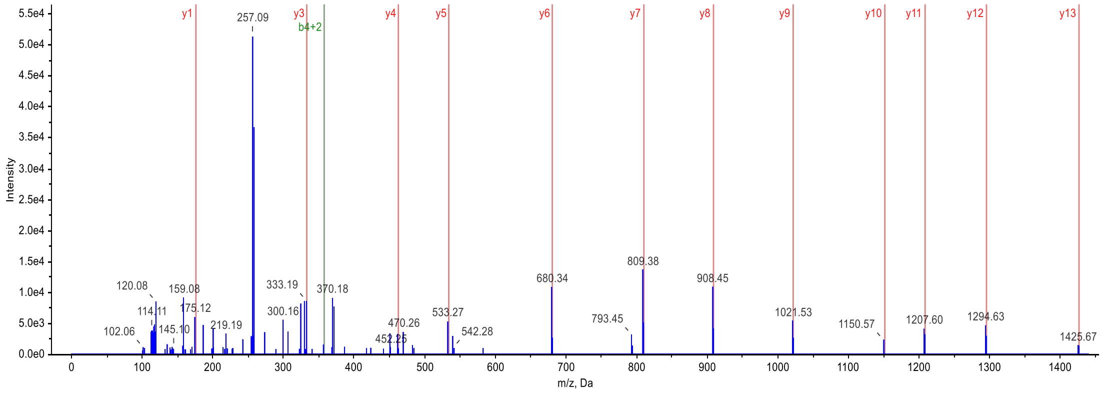

29 Accession No. ABR67212/gi|150035733

29.1 GFQLILS[Pho]GELDALPEQAFYLVGNIDEASTK

Residue	b	b+2	y	y+2
G	362.2341	181.6207	3832.9870	1916.9971
F	509.3025	255.1549	3471.7602	1736.3837
Q	637.3611	319.1842	3324.6917	1662.8495
L	750.4452	375.7262	3196.6332	1598.8202
I	863.5292	432.2682	3083.5491	1542.2782
L	976.6133	488.8103	2970.4650	1485.7362
S[Pho]	1143.6116	572.3095	2857.3810	1429.1941
G	1200.6331	600.8202	2690.3826	1345.6949
E	1329.6757	665.3415	2633.3612	1317.1842
L	1442.7598	721.8835	2504.3186	1252.6629
D	1557.7867	779.3970	2391.2345	1196.1209
A	1628.8238	814.9155	2276.2076	1138.6074
L	1741.9079	871.4576	2205.1704	1103.0889
P	1838.9606	919.9840	2092.0864	1046.5468
E	1968.0032	984.5053	1995.0336	998.0204

Residue	b	b+2	y	y+2
Q	2096.0618	1048.5345	1865.9910	933.4991
A	2167.0989	1084.0531	1737.9324	869.4699
F	2314.1673	1157.5873	1666.8953	833.9513
Y[YDA]	2383.1888	1192.0980	1519.8269	760.4171
L	2496.2729	1248.6401	1450.8055	725.9064
V	2595.3413	1298.1743	1337.7214	669.3643
G	2652.3628	1326.6850	1238.6530	619.8301
N	2766.4057	1383.7065	1181.6315	591.3194
I	2879.4897	1440.2485	1067.5886	534.2979
D	2994.5167	1497.7620	954.5045	477.7559
E	3123.5593	1562.2833	839.4776	420.2424
A	3194.5964	1597.8018	710.4350	355.7211
S	3281.6284	1641.3178	639.3979	320.2026
T	3382.6761	1691.8417	552.3658	276.6866
K[IT8]	3814.9764	1907.9918	451.3182	226.1627

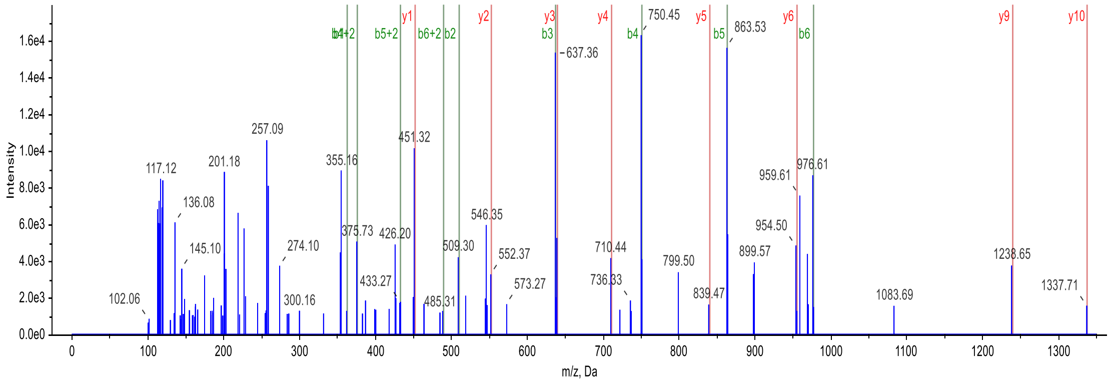

30 Accession No. XP_004960054/gi|514742084

30.1 QVAHAPQELNS[Pho]PR

Residue	b	b+2	y	y+2
Q	433.2712	217.1392	1830.9165	915.9619
V	532.3396	266.6735	1398.6526	699.8299
A	603.3767	302.1920	1299.5841	650.2957
H	740.4357	370.7215	1228.5470	614.7772
A	811.4728	406.2400	1091.4881	546.2477
P	908.5255	454.7664	1020.4510	510.7291
Q	1036.5841	518.7957	923.3982	462.2028
E	1165.6267	583.3170	795.3397	398.1735
L	1278.7108	639.8590	666.2971	333.6522
N	1392.7537	696.8805	553.2130	277.1101
S[Pho]	1559.7521	780.3797	439.1701	220.0887
P	1656.8048	828.9060	272.1717	136.5895
R	1812.9059	906.9566	175.1190	88.0631

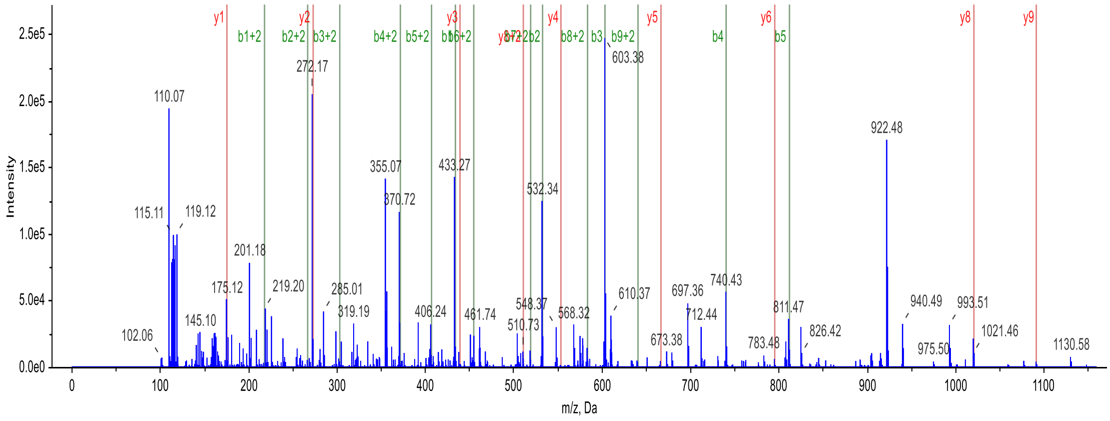

31 Accession No. EMS47290/gi|473841507

31.1 VAEQLS[Pho]DDEGEDQSK

Residue	b	b+2	y	y+2
V	**404.2810**	202.6442	2338.0907	1169.5490
A	**475.3182**	238.1627	1934.8170	967.9121
E	**604.3608**	**302.6840**	1863.7798	932.3936
Q	**732.4193**	366.7133	1734.7372	867.8723
L	**845.5034**	423.2553	1606.6787	803.8430
S[Pho]	1012.5018	506.7545	1493.5946	747.3009
D	1127.5287	564.2680	1326.5962	663.8018
D	1242.5556	621.7815	1211.5693	606.2883
E	1371.5982	686.3028	**1096.5424**	548.7748
G	1428.6197	714.8135	**967.4998**	484.2535
E	1557.6623	779.3348	**910.4783**	455.7428
D	1672.6892	836.8483	**781.4357**	391.2215
Q	1800.7478	900.8775	**666.4088**	333.7080
S	1887.7798	944.3936	**538.3502**	269.6787
K[IT8]	2320.0802	1160.5437	**451.3182**	226.1627

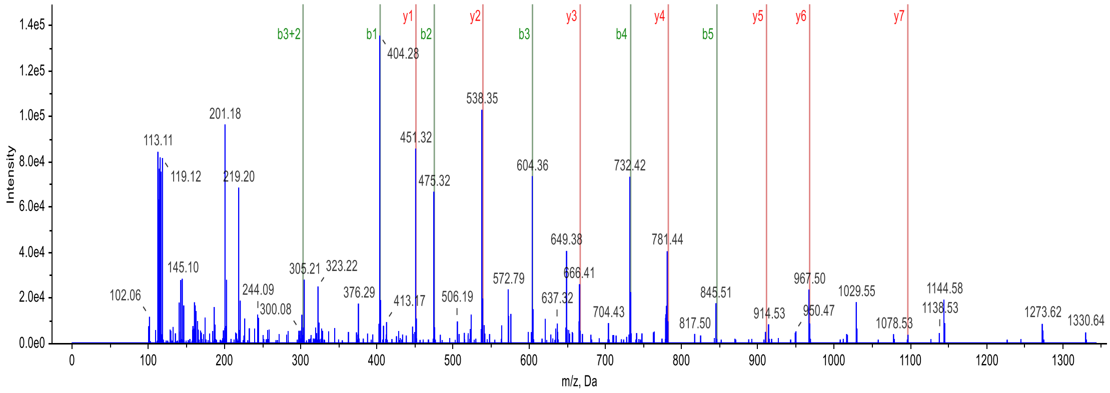

32 Accession No. BAJ91166/gi|326505854

32.1 AAAVAALSSVLTAEQSGS[Pho]SDNLR

Residue	b	b+2	y	y+2
A	**376.2497**	188.6285	2602.3027	1301.6550
A	**447.2869**	224.1471	2227.0602	1114.0337
A	**518.3240**	**259.6656**	2156.0231	1078.5152
V	**617.3924**	**309.1998**	2084.9860	1042.9966
A	**688.4295**	**344.7184**	1985.9176	993.4624
A	**759.4666**	**380.2369**	1914.8804	957.9439
L	**872.5507**	**436.7790**	1843.8433	922.4253
S	**959.5827**	**480.2950**	1730.7593	865.8833
S	**1046.6147**	523.8110	1643.7272	822.3673
V	1145.6832	**573.3452**	1556.6952	778.8512
L	**1258.7672**	629.8872	**1457.6268**	729.3170
T	**1359.8149**	**680.4111**	**1344.5427**	672.7750
A	1430.8520	715.9296	**1243.4951**	622.2512
E	1559.8946	780.4509	**1172.4579**	586.7326
Q	1687.9532	844.4802	1043.4153	522.2113
S	1774.9852	887.9962	**915.3568**	458.1820
G	1832.0067	916.5070	**828.3247**	414.6660
S[Pho]	1999.0050	1000.0062	**771.3033**	386.1553
S	2086.0371	1043.5222	**604.3049**	302.6561
D	2201.0640	1101.0356	**517.2729**	259.1401
N	2315.1069	1158.0571	**402.2459**	201.6266
L	2428.1910	1214.5991	**288.2030**	144.6051
R	2584.2921	1292.6497	**175.1190**	88.0631

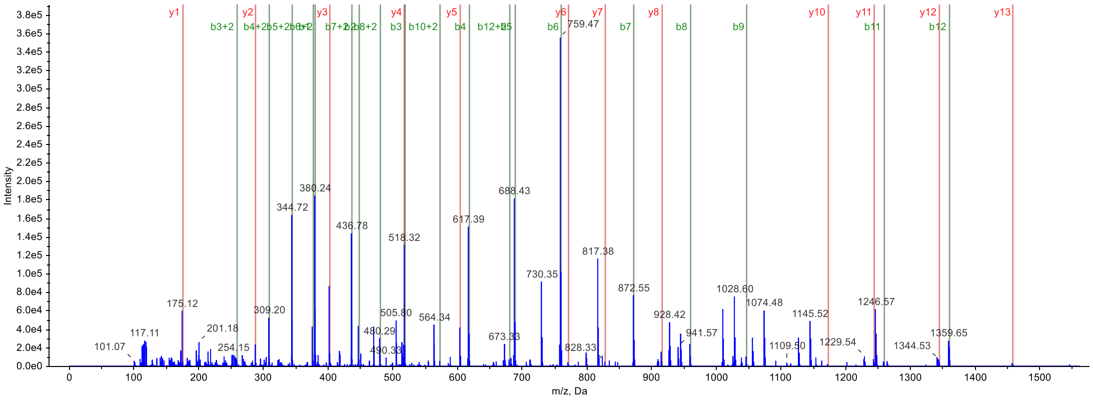

33 Accession No. EMS67681/gi|474425093

33.1 SDS[Pho]QVFLFANSK

Residue	b	b+2	y	y+2
S	392.2447	196.6260	2031.0408	1016.0240
D	507.2716	254.1394	1639.8034	820.4053
S[Pho]	674.2700	337.6386	1524.7765	762.8919
Q	802.3285	401.6679	1357.7781	679.3927
V	901.3970	451.2021	1229.7195	615.3634
F	1048.4654	524.7363	1130.6511	565.8292
L	1161.5494	581.2784	983.5827	492.2950
F	1308.6178	654.8126	870.4986	435.7530
A	1379.6550	690.3311	723.4302	362.2188
N	1493.6979	747.3526	652.3931	326.7002
S	1580.7299	790.8686	538.3502	269.6787
K[IT8]	2013.0302	1007.0188	451.3182	226.1627

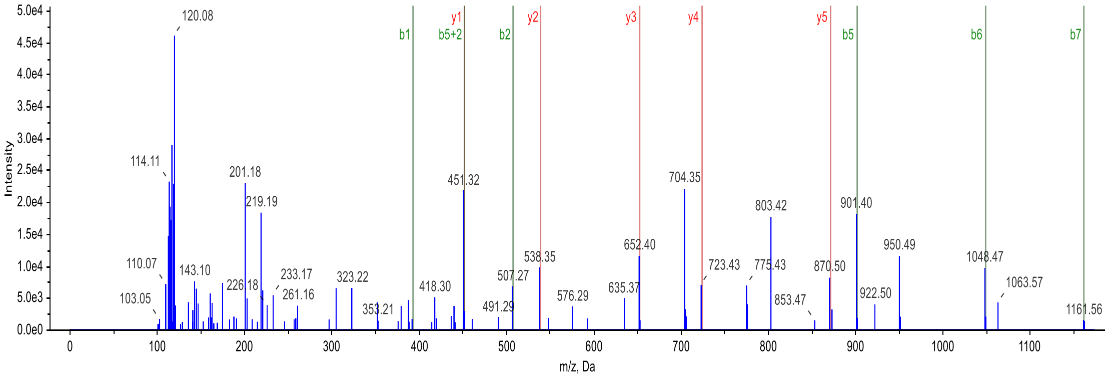

34 Accession No. XP_003559644/gi|357115740

34.1 EWQTAAAAAFSES[Pho]DVDEEEDEDELVEVIGAEDEETVLTK

Residue	b	b+2	y	y+2
E	434.2552	217.6313	4969.2659	2485.1366
W	620.3345	310.6709	4536.0180	2268.5126
Q	748.3931	374.7002	4349.9387	2175.4730
T[Dhy]	831.4302	416.2188	4221.8801	2111.4437
A	902.4673	451.7373	4138.8430	2069.9251
A	973.5045	487.2559	4067.8058	2034.4066
A	1044.5416	522.7744	3996.7687	1998.8880
A	1115.5787	558.2930	3925.7316	1963.3694
A	1186.6158	593.8115	3854.6945	1927.8509
F	1333.6842	667.3457	3783.6574	1892.3323
S	1420.7162	710.8618	3636.5890	1818.7981
E	1549.7588	775.3831	3549.5570	1775.2821
S[Pho]	1716.7572	858.8822	3420.5144	1710.7608
D	1831.7841	916.3957	3253.5160	1627.2616
V	1930.8526	965.9299	3138.4891	1569.7482
D	2045.8795	1023.4434	3039.4206	1520.2140
E	2174.9221	1087.9647	2924.3937	1462.7005
E	2303.9647	1152.4860	2795.3511	1398.1792
E	2433.0073	1217.0073	2666.3085	1333.6579
D	2548.0342	1274.5207	2537.2659	1269.1366

Residue	b	b+2	y	y+2
E	2677.0768	1339.0420	2422.2390	1211.6231
D	2792.1038	1396.5555	2293.1964	1147.1018
E	2921.1463	1461.0768	2178.1694	1089.5884
L	3034.2304	1517.6188	2049.1268	1025.0671
V	3133.2988	1567.1531	1936.0428	968.5250
E	3262.3414	1631.6743	1836.9744	918.9908
V	3361.4098	1681.2086	1707.9318	854.4695
I	3474.4939	1737.7506	1608.8634	804.9353
G	3531.5154	1766.2613	1495.7793	748.3933
A	3602.5525	1801.7799	1438.7578	719.8826
E	3731.5951	1866.3012	1367.7207	684.3640
D	3846.6220	1923.8146	1238.6781	619.8427
E	3975.6646	1988.3359	1123.6512	562.3292
E	4104.7072	2052.8572	994.6086	497.8079
T	4205.7549	2103.3811	865.5660	433.2866
V	4304.8233	2152.9153	764.5183	382.7628
L	4417.9074	2209.4573	665.4499	333.2286
T	4518.9550	2259.9812	552.3658	276.6866
K[IT8]	4951.2554	2476.1313	451.3182	226.1627

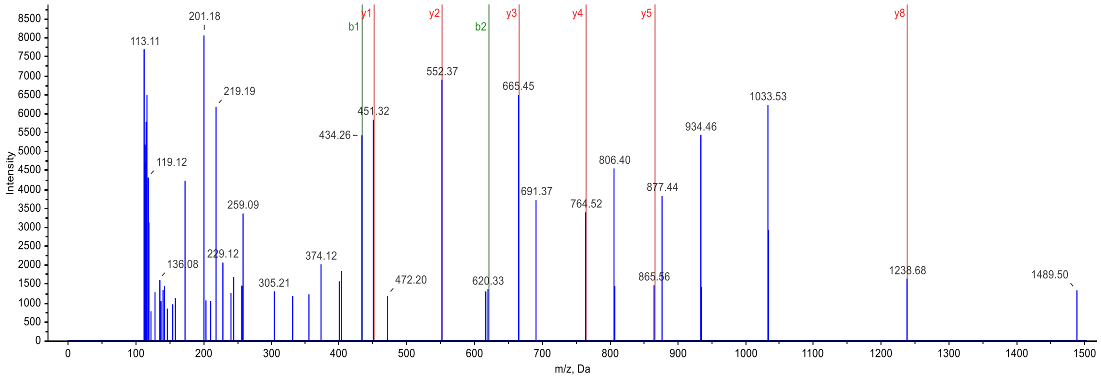

35 Accession No. XP_006300324/ gi|565485369

35.1 LSELLGVEVVMANDS[Pho]IGEEVQKLVAALPEGGVLLLENVR

Residue	b	b+2	y	y+2
L	140.1070	70.5571	4513.4182	2257.2127
S	227.1390	114.0731	4374.3184	2187.6629
E	356.1816	178.5944	4287.2864	2144.1468
L	469.2657	235.1365	4158.2438	2079.6256
L	582.3497	291.6785	4045.1598	2023.0835
G	639.3712	320.1892	3932.0757	1966.5415
V	738.4396	369.7234	3875.0542	1938.0308
E	867.4822	434.2447	3775.9858	1888.4965
V	966.5506	483.7790	3646.9432	1823.9753
V	1065.6190	533.3132	3547.8748	1774.4410
M	1196.6595	598.8334	3448.8064	1724.9068
A	1267.6966	634.3520	3317.7659	1659.3866
N	1381.7396	691.3734	3246.7288	1623.8680
D	1496.7665	748.8869	3132.6859	1566.8466
S[Pho]	1663.7649	832.3861	3017.6589	1509.3331
I	1776.8489	888.9281	2850.6606	1425.8339
G	1833.8704	917.4388	2737.5765	1369.2919
E	1962.9130	981.9601	2680.5550	1340.7812
E	2091.9556	1046.4814	2551.5125	1276.2599
V	2191.0240	1096.0156	2422.4699	1211.7386

Residue	b	b+2	y	y+2
Q	2319.0826	1160.0449	2323.4014	1162.2044
K[IT8]	2751.3829	1376.1951	2195.3429	1098.1751
L	2864.4670	1432.7371	1763.0425	882.0249
V	2963.5354	1482.2713	1649.9585	825.4829
A	3034.5725	1517.7899	1550.8901	775.9487
A	3105.6096	1553.3084	1479.8530	740.4301
L	3218.6937	1609.8505	1408.8158	704.9116
P	3315.7464	1658.3769	1295.7318	648.3695
E	3444.7890	1722.8981	1198.6790	599.8431
G	3501.8105	1751.4089	1069.6364	535.3218
G	3558.8319	1779.9196	1012.6150	506.8111
V	3657.9004	1829.4538	955.5935	478.3004
L	3770.9844	1885.9959	856.5251	428.7662
L	3884.0685	1942.5379	743.4410	372.2241
L	3997.1526	1999.0799	630.3570	315.6821
E	4126.1951	2063.6012	517.2729	259.1401
N	4240.2381	2120.6227	388.2303	194.6188
V	4339.3065	2170.1569	274.1874	137.5973
R	4495.4076	2248.2074	175.1190	88.0631

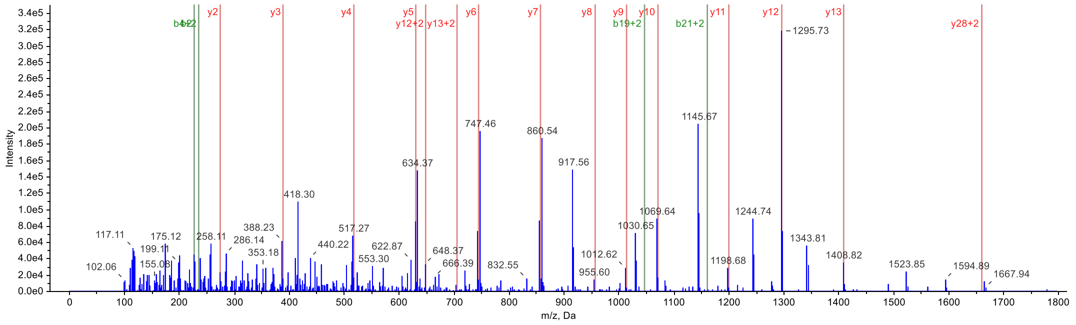

36 Accession No. XP_003564482/ gi|357125604

36.1 AHGTAVGLPSDDDMGNS[Pho]EVGHNALGAGR

Residue	b	b+2	y	y+2
A	376.2497	188.6285	3089.4049	1545.2061
H	513.3087	257.1580	2714.1624	1357.5849
G	570.3301	285.6687	2577.1035	1289.0554
T	671.3778	336.1925	2520.0821	1260.5447
A	742.4149	371.7111	2419.0344	1210.0208
V	841.4833	421.2453	2347.9973	1174.5023
G	898.5048	449.7560	2248.9289	1124.9681
L	1011.5889	506.2981	2191.9074	1096.4573
P	1108.6416	554.8244	2078.8233	1039.9153
S	1195.6737	598.3405	1981.7706	991.3889
D	1310.7006	655.8539	1894.7385	947.8729
D	1425.7275	713.3674	1779.7116	890.3594
D	1540.7545	770.8809	1664.6847	832.8460
M	1671.7950	836.4011	1549.6577	775.3325

Residue	b	b+2	y	y+2
G	1728.8164	864.9119	1418.6172	709.8123
N	1842.8594	921.9333	1361.5958	681.3015
S[Pho]	2009.8577	1005.4325	1247.5528	624.2801
E	2138.9003	1069.9538	1080.5545	540.7809
V	2237.9687	1119.4880	951.5119	476.2596
G	2294.9902	1147.9987	852.4435	426.7254
H	2432.0491	1216.5282	795.4220	398.2146
N	2546.0920	1273.5497	658.3631	329.6852
A	2617.1291	1309.0682	544.3202	272.6637
L	2730.2132	1365.6102	473.2831	237.1452
G	2787.2347	1394.1210	360.1990	180.6031
A	2858.2718	1429.6395	303.1775	152.0924
G	2915.2932	1458.1503	232.1404	116.5738
R	3071.3944	1536.2008	175.1190	88.0631

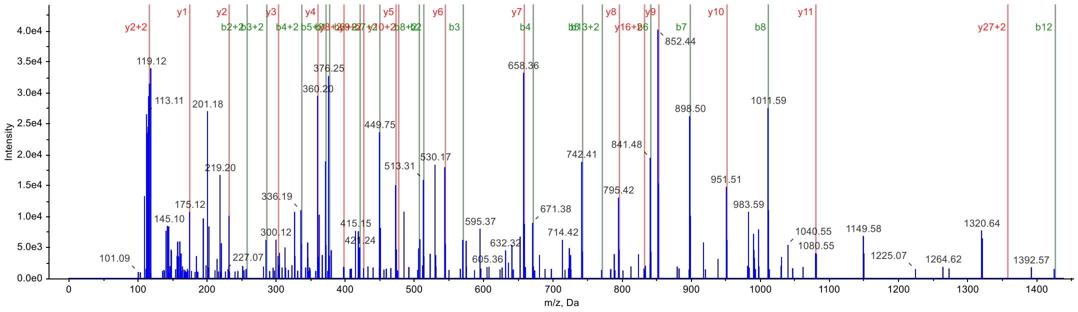

37 Accession No. BAD36412/ gi|51091643

37.1 LSS[Pho]IDAQLR

Residue	b	b+2	y	y+2
L	418.2967	209.6620	1386.7296	693.8684
S	505.3287	253.1680	969.4401	485.2237
S[Pho]	672.3271	336.6672	882.4081	441.7077
I	785.4112	393.2092	715.4097	358.2085
D	900.4381	450.7227	602.3257	301.6665
A	971.4752	486.2412	487.2987	244.1530
Q	1099.5338	550.2705	416.2616	208.6344
L	1212.6178	606.8126	288.2030	144.6051
R	1368.7190	684.8631	175.1190	88.0631

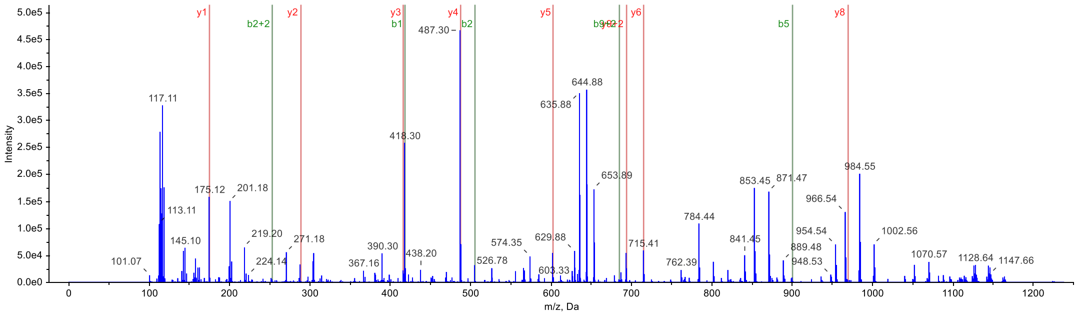

38 Accession No. EMT08717/ gi|475535263

38.1 ATGAFILTAS[Pho]HNPGGPTEDFGIK

Residue	b	b+2	y	y+2
A	376.2497	188.6285	3005.5230	1503.2651
T	477.2974	239.1524	2630.2805	1315.6439
G	534.3189	267.6631	2529.2328	1265.1200
A	605.3560	303.1816	2472.2113	1236.6093
F[Oxi]	768.4193	384.7133	2401.1742	1201.0907
I	881.5034	441.2553	2238.1109	1119.5591
L	994.5875	497.7974	2125.0268	1063.0171
T	1095.6351	548.3212	2011.9428	1006.4750
A	1166.6723	583.8398	1910.8951	955.9512
S[Pho]	1333.6706	667.3389	1839.8580	920.4326
H	1470.7295	735.8684	1672.8596	836.9334
N	1584.7725	792.8899	1535.8007	768.4040
P	1681.8252	841.4162	1421.7578	711.3825
G	1738.8467	869.9270	1324.7050	662.8561
G	1795.8681	898.4377	1267.6835	634.3454
P	1892.9209	946.9641	1210.6621	605.8347
T	1993.9686	997.4879	1113.6093	557.3083
E	2123.0112	1062.0092	1012.5616	506.7845
D	2238.0381	1119.5227	883.5190	442.2632
F	2385.1065	1193.0569	768.4921	384.7497
G	2442.1280	1221.5676	621.4237	311.2155
I	2555.2121	1278.1097	564.4022	282.7048
K[IT8]	2987.5124	1494.2598	451.3182	226.1627

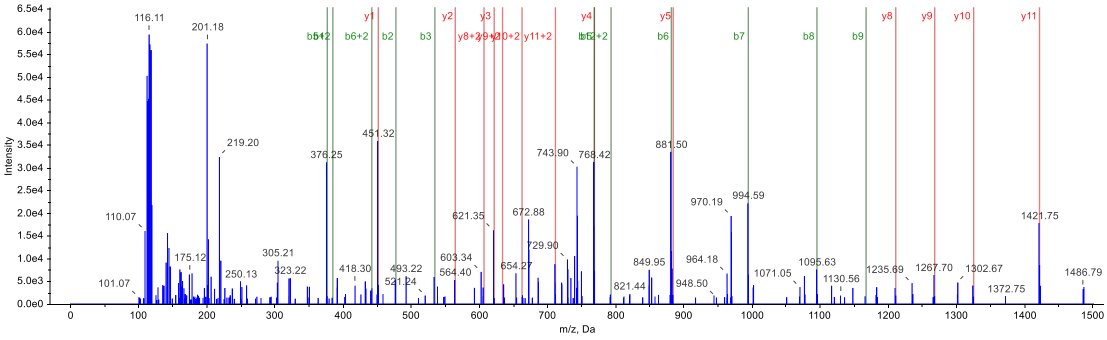

39 Accession No. EMT06309/ gi|475519222

39.1 FDWDHPVHLQPMS[Pho]PTTTK

Residue	b	b+2	y	y+2
F	452.2810	226.6442	2841.3891	1421.1982
D	567.3080	284.1576	2390.1153	1195.5613
W	753.3873	377.1973	2275.0884	1138.0478
D	868.4142	434.7108	2089.0091	1045.0082
H	1005.4732	503.2402	1973.9821	987.4947
P	1102.5259	551.7666	1836.9232	918.9652
V	1201.5943	601.3008	1739.8706	870.4389
H	1338.6533	669.8303	1640.8020	820.9047
L	1451.7373	726.3723	1503.7431	752.3752
Q	1579.7959	790.4016	1390.6591	695.8332
P	1676.8487	838.9280	1262.6005	631.8039
M[Oxi]	1823.8841	912.4457	1165.5477	583.2775
S[Pho]	1990.8824	995.9448	1018.5123	509.7598
P	2087.9352	1044.4712	851.5140	426.2606
T	2188.9829	1094.9951	754.4612	377.7342
T	2290.0306	1145.5189	653.4136	327.2104
T	2391.0782	1196.0427	552.3658	276.6866
K[IT8]	2823.3785	1412.1929	451.3182	226.1627

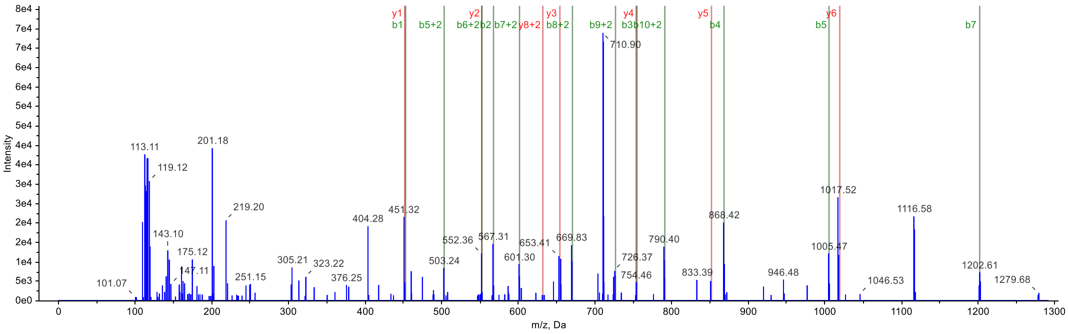

40 Accession No. EMT21987/ gi|475592099

40.1 FDWDHPMHLQPTS[Pho]PTAVK

Residue	b	b+2	y	y+2
F	452.2810	226.6442	2795.3836	1398.1954
D	567.3080	284.1576	2344.1098	1172.5586
W	753.3873	377.1973	2229.0829	1115.0451
D	868.4142	434.7108	2043.0036	1022.0054
H	1005.4732	503.2402	1927.9766	964.4920
P	1102.5259	551.7666	1790.9177	895.9625
M	1233.5664	617.2868	1693.8650	847.4361
H	1370.6253	685.8163	1562.8245	781.9159
L	1483.7094	742.3583	1425.7656	713.3864
Q	1611.7680	806.3876	1312.6815	656.8444
P	1708.8207	854.9140	1184.6229	592.8151
T	1809.8684	905.4378	1087.5702	544.2887
S[Pho]	1976.8668	988.9370	986.5225	493.7649
P	2073.9195	1037.4634	819.5241	410.2657
T	2174.9672	1087.9872	722.4714	361.7393
A	2246.0043	1123.5058	621.4237	311.2155
V	2345.0727	1173.0400	550.3866	275.6969
K[IT8]	2777.3731	1389.1902	451.3182	226.1627

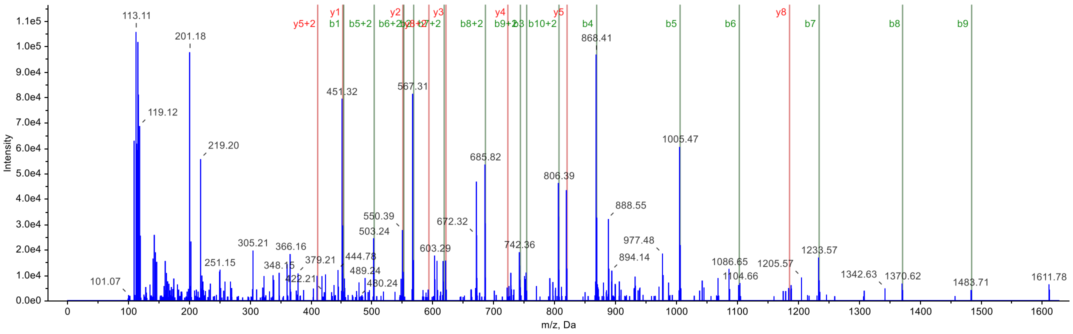

41 Accession No. EMS63629/ gi|474368000

41.1 SDDATEVSETDS[Pho]PGDSLR

Residue	b	b+2	y	y+2
S	392.2447	196.6260	2264.9709	1132.9891
D	507.2716	254.1394	1873.7336	937.3704
D	622.2986	311.6529	1758.7066	879.8569
A	693.3357	347.1715	1643.6796	822.3434
T	794.3833	397.6953	1572.6425	786.8249
E	923.4259	462.2166	1471.5948	736.3011
V	1022.4943	511.7508	1342.5522	671.7798
S	1109.5264	555.2668	1243.4838	622.2455
E	1238.5690	619.7881	1156.4518	578.7295
T	1339.6166	670.3120	1027.4092	514.2082
D	1454.6436	727.8254	926.3615	463.6844
S[Pho]	1621.6420	811.3246	811.3346	406.1709
P	1718.6947	859.8510	644.3362	322.6717
G	1775.7162	888.3617	547.2835	274.1454
D	1890.7431	945.8752	490.2620	245.6346
S	1977.7752	989.3912	375.2350	188.1212
L	2090.8592	1045.9332	288.2030	144.6051
R	2246.9603	1123.9838	175.1190	88.0631

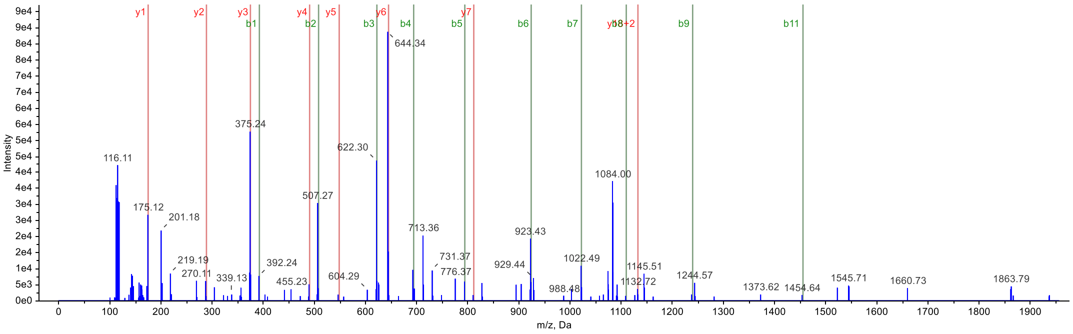

42 Accession No. BAN63108/ gi|524456011

42.1 QYS[Pho]SGGTEK

Residue	b	b+2	y	y+2
G	362.2341	181.6207	1378.7551	689.8812
A	433.2712	217.1392	1017.5283	509.2678
A	504.3083	252.6578	946.4912	473.7492
S[Pho]	671.3067	336.1570	875.4541	438.2307
L	784.3908	392.6990	708.4557	354.7315
S	871.4228	436.2150	595.3717	298.1895
G	928.4442	464.7258	508.3396	254.6735
K[IT8]	1360.7446	680.8759	451.3182	226.1627

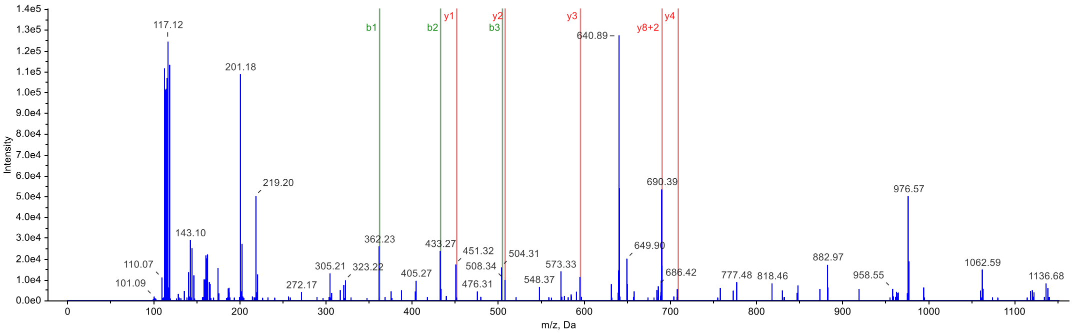

42.2 QYSS[Pho]GGTEK

Residue	b	b+2	y	y+2
Q[Dea]	434.2552	217.6313	1645.7930	823.4002
Y	597.3186	299.1629	1212.5451	606.7762
S[Pho]	764.3169	382.6621	1049.4817	525.2445
S	851.3489	426.1781	882.4834	441.7453
G	908.3704	454.6888	795.4514	398.2293
G	965.3919	483.1996	738.4299	369.7186
T	1066.4396	533.7234	681.4084	341.2079
E	1195.4821	598.2447	580.3608	290.6840
K[IT8]	1627.7825	814.3949	451.3182	226.1627

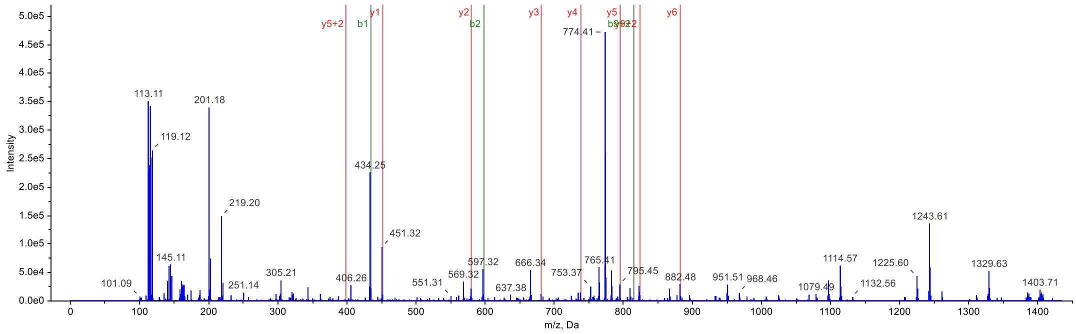

42.3 GAAS[Pho]LSGK

Residue	b	b+2	y	y+2
Q	433.2712	217.1392	1644.8090	822.9081
Y	596.3345	298.6709	1212.5451	606.7762
S	683.3666	342.1869	1049.4817	525.2445
S[Pho]	850.3649	425.6861	962.4497	481.7285
G	907.3864	454.1968	795.4514	398.2293
G	964.4079	482.7076	738.4299	369.7186
T	1065.4555	533.2314	681.4084	341.2079
E	1194.4981	597.7527	580.3608	290.6840
K[IT8]	1626.7985	813.9029	451.3182	226.1627

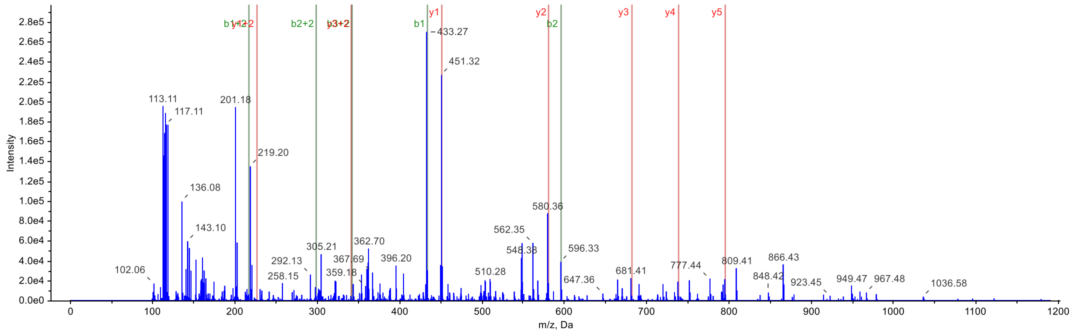

43 Accession No. EMS54049/ gi|474063387

43.1 AMNKPAEYDS[Pho]DDEIIGTAR

Residue	b	b+2	y	y+2
A	376.2497	188.6285	2784.3371	1392.6722
M	507.2902	254.1488	2409.0947	1205.0510
N	621.3332	311.1702	2278.0542	1139.5307
K[IT8]	1053.6335	527.3204	2164.0112	1082.5093
P	1150.6862	575.8468	1731.7109	866.3591
A	1221.7234	611.3653	1634.6582	817.8327
E	1350.7660	675.8866	1563.6210	782.3142
Y	1513.8293	757.4183	1434.5784	717.7929
D	1628.8562	814.9318	1271.5151	636.2612
S[Pho]	1795.8546	898.4309	1156.4882	578.7477
D	1910.8815	955.9444	989.4898	495.2485
D	2025.9085	1013.4579	874.4629	437.7351
E	2154.9511	1077.9792	759.4359	380.2216
I	2268.0351	1134.5212	630.3933	315.7003
I	2381.1192	1191.0632	517.3093	259.1583
G	2438.1407	1219.5740	404.2252	202.6162
T	2539.1883	1270.0978	347.2037	174.1055
A	2610.2254	1305.6164	246.1561	123.5817
R	2766.3266	1383.6669	175.1190	88.0631

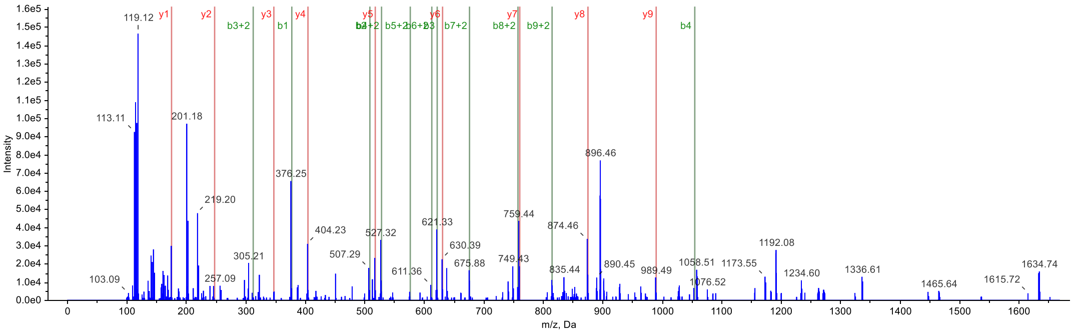

44 Accession No. AAC05084/ gi|2754825

44.1 DEDPLLDS[Pho]DDERPESFDDELR

Residue	b	b+2	y	y+2
D	420.2396	210.6234	2891.2409	1446.1241
E	549.2822	275.1447	2472.0086	1236.5079
D	664.3091	332.6582	2342.9660	1171.9866
P	761.3619	381.1846	2227.9391	1114.4732
L	874.4459	437.7266	2130.8863	1065.9468
L	987.5300	494.2686	2017.8023	1009.4048
D	1102.5569	551.7821	1904.7182	952.8627
S[Pho]	1269.5553	635.2813	1789.6912	895.3493
D	1384.5823	692.7948	1622.6929	811.8501
D	1499.6092	750.3082	1507.6659	754.3366
E	1628.6518	814.8295	1392.6390	696.8231
R	1784.7529	892.8801	1263.5964	632.3018
P	1881.8057	941.4065	1107.4953	554.2513
E	2010.8483	1005.9278	1010.4425	505.7249
S	2097.8803	1049.4438	881.3999	441.2036
F	2244.9487	1122.9780	794.3679	397.6876
D	2359.9756	1180.4915	647.2995	324.1534
D	2475.0026	1238.0049	532.2726	266.6399
E	2604.0452	1302.5262	417.2456	209.1264
L	2717.1292	1359.0683	288.2030	144.6051
R	2873.2304	1437.1188	175.1190	88.0631

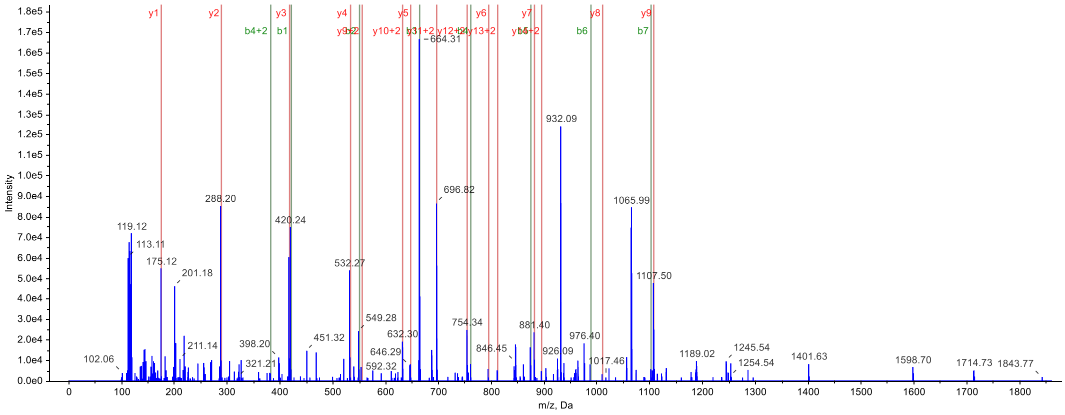

45 Accession No. AGW21711/ gi|544370429

45.1 S[Pho]LGQNPTEAELQAMINEVDADGNGTIDFPEFLNLMAR

Residue	b	b+2	y	y+2
S[Pho]	472.2110	236.6091	4487.9968	2244.5020
L	585.2951	293.1512	4016.7931	2008.9002
G	642.3165	321.6619	3903.7090	1952.3581
Q	770.3751	385.6912	3846.6875	1923.8474
N	884.4180	442.7127	3718.6290	1859.8181
P	981.4708	491.2390	3604.5860	1802.7967
T	1082.5185	541.7629	3507.5333	1754.2703
E	1211.5611	606.2842	3406.4856	1703.7464
A	1282.5982	641.8027	3277.4430	1639.2261
E	1411.6408	706.3240	3206.4059	1603.7066
L	1524.7248	762.8661	3077.3633	1539.1853
Q	1652.7834	826.8953	2964.2792	1482.6433
D	1767.8104	884.4088	2836.2207	1418.6140
M	1898.8508	949.9291	2721.1937	1361.1005
I	2011.9349	1006.4711	2590.1532	1295.5803
N	2125.9778	1063.4926	2477.0692	1239.0382
E[KXX]	2292.9763	1146.9918	2363.0262	1182.0168
V	2392.0447	1196.5260	2196.0278	1098.5175

Residue	b	b+2	y	y+2
D	2507.0717	1254.0395	2096.9593	1048.9833
A	2578.1088	1289.5580	1981.9324	991.4698
D	2693.1357	1347.0715	1910.8953	955.9513
G	2750.1572	1375.5822	1795.8683	898.4378
N[Dea]	2865.1841	1433.0957	1738.8469	869.9271
G	2922.2056	1461.6064	1623.8199	812.4136
T	3023.2533	1512.1303	1566.7985	783.9029
I	3136.3373	1568.6723	1465.7508	733.3790
D	3251.3643	1626.1858	1352.6667	676.8370
F	3398.4327	1699.7200	1237.6398	619.3235
P	3495.4855	1748.2464	1090.5714	545.7893
E	3624.5280	1812.7677	993.5186	497.2629
F	3771.5965	1886.3019	864.4760	432.7416
L	3884.6805	1942.8439	717.4076	359.2074
N	3998.7236	1999.8654	604.3235	302.6654
L	4111.8075	2056.4074	490.2806	245.6439
M	4242.8480	2121.9276	377.1966	189.1019
A	4313.8851	2157.4462	246.1561	123.5817
R	4469.9862	2235.4968	175.1190	88.0631

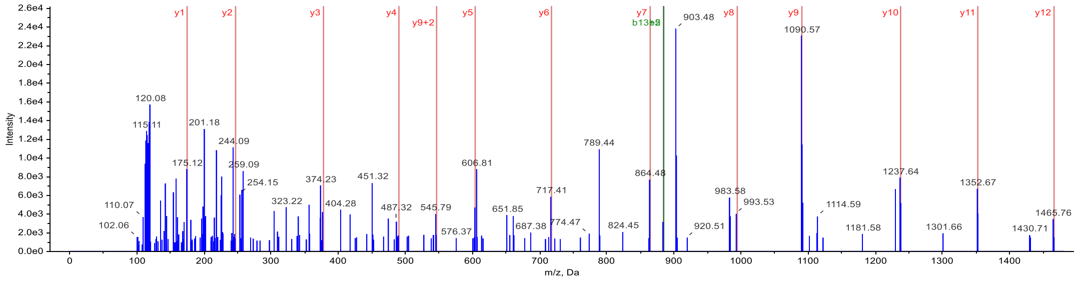

45.2 SLGQNPT[Pho]EAELQAMINEVDADGNGTIDFPEFLNLMAR

Residue	b	b+2	y	y+2
S	392.2447	196.6260	4691.2619	2346.1346
L	505.3287	253.1680	4300.0245	2150.5159
G	562.3502	281.6787	4186.9404	2093.9738
Q	690.4088	345.7080	4129.9189	2065.4631
N	804.4517	402.7295	4001.8604	2001.4338
P	901.5045	451.2559	3887.8174	1944.4124
T[Pho]	1082.5185	541.7629	3790.7647	1895.8860
E	1211.5611	606.2842	3609.7507	1805.3790
A	1282.5982	641.8027	3480.7081	1740.8577
E	1411.6408	706.3240	3409.6710	1705.3391
L	1524.7248	762.8661	3280.6284	1640.8178
Q	1652.7834	826.8953	3167.5443	1584.2758
A	1723.8205	862.4139	3039.4857	1520.2465
M	1854.8610	927.9341	2968.4486	1484.7279
I	1967.9451	984.4762	2837.4081	1419.2077
N	2081.9880	1041.4976	2724.3241	1362.6657
E	2211.0306	1106.0189	2610.2811	1305.6442
V	2310.0990	1155.5531	2481.2385	1241.1229

Residue	b	b+2	y	y+2
D	2425.1260	1213.0666	2382.1701	1191.5887
A	2496.1631	1248.5852	2267.1432	1134.0752
D[Dhy]	2593.1794	1297.0934	2196.1061	1098.5567
G	2650.2009	1325.6041	2099.0897	1050.0485
N	2764.2438	1382.6256	2042.0682	1021.5378
G	2821.2653	1411.1363	1928.0253	964.5163
T[IT8]	3226.5183	1613.7628	1871.0038	936.0056
I	3339.6024	1670.3048	1465.7508	733.3790
D	3454.6293	1727.8183	1352.6667	676.8370
F	3601.6978	1801.3525	1237.6398	619.3235
P	3698.7505	1849.8789	1090.5714	545.7893
E	3827.7931	1914.4002	993.5186	497.2629
F	3974.8615	1987.9344	864.4760	432.7416
L	4087.9456	2044.4764	717.4076	359.2074
N	4201.9885	2101.4979	604.3235	302.6654
L	4315.0726	2158.0399	490.2806	245.6439
M	4446.1131	2223.5602	377.1966	189.1019
A	4517.1502	2259.0787	246.1561	123.5817
R	4673.2513	2337.1293	175.1190	88.0631

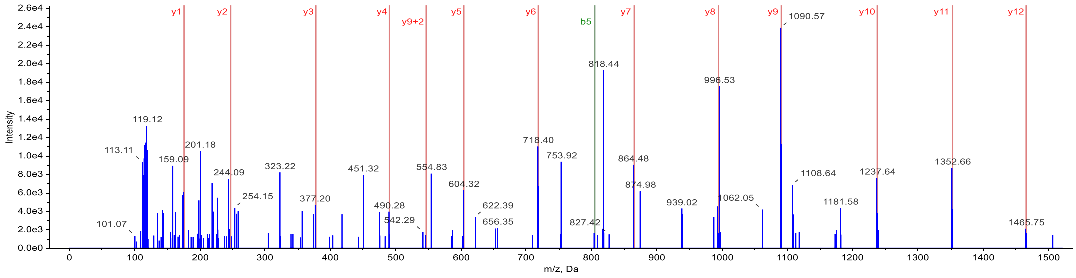

45.3 SLGQNPTEAELQAMINEVDADGNGT[Pho]IDFPEFLNLMAR

Residue	b	b+2	y	y+2
S	392.2447	196.6260	4487.0338	2244.0205
L	505.3287	253.1680	4095.7964	2048.4018
G	562.3502	281.6787	3982.7123	1991.8598
Q	690.4088	345.7080	3926.6908	1963.3491
N	804.4517	402.7295	3797.6323	1899.3198
P	901.5045	451.2559	3683.5893	1842.2983
T	1002.5521	501.7797	3586.5366	1793.7719
E	1131.5947	566.3010	3485.4889	1743.2481
A	1202.6318	601.8196	3356.4463	1678.7268
E[NaX]	1353.6564	677.3318	3285.4092	1643.2082
L	1466.7404	733.8739	3134.3846	1567.6960
Q	1594.7990	797.9032	3021.3006	1511.1539
D	1709.8260	855.4166	2893.2420	1447.1246
M[Oxi]	1856.8614	928.9343	2778.2151	1389.6112
I	1969.9454	985.4764	2631.1797	1316.0935
N	2083.9884	1042.4978	2518.0956	1259.5514
E	2213.0310	1107.0191	2404.0527	1202.5300
V	2312.0994	1156.5533	2275.0101	1138.0087

Residue	b	b+2	y	y+2
D	2427.1263	1214.0668	2175.9417	1088.4745
A	2498.1634	1249.5853	2060.9147	1030.9610
D	2613.1904	1307.0988	1989.8776	995.4424
G	2670.2118	1335.6096	1874.8507	937.9290
N	2784.2548	1392.6310	1817.8292	909.4182
G	2841.2762	1421.1417	1703.7863	852.3968
T[Pho]	3022.2902	1511.6488	1646.7648	823.8860
I	3135.3743	1568.1908	1465.7508	733.3790
D	3250.4012	1625.7043	1352.6667	676.8370
F	3397.4697	1699.2385	1237.6398	619.3235
P	3494.5224	1747.7648	1090.5714	545.7893
E	3623.5650	1812.2861	993.5186	497.2629
F	3770.6334	1885.8203	864.4760	432.7416
L	3883.7175	1942.3624	717.4076	359.2074
N	3997.7604	1999.3838	604.3235	302.6654
L	4110.8445	2055.9259	490.2806	245.6439
M	4241.8850	2121.4461	377.1966	189.1019
A	4312.9221	2156.9647	246.1561	123.5817
R	4469.0232	2235.0162	175.1190	88.0631

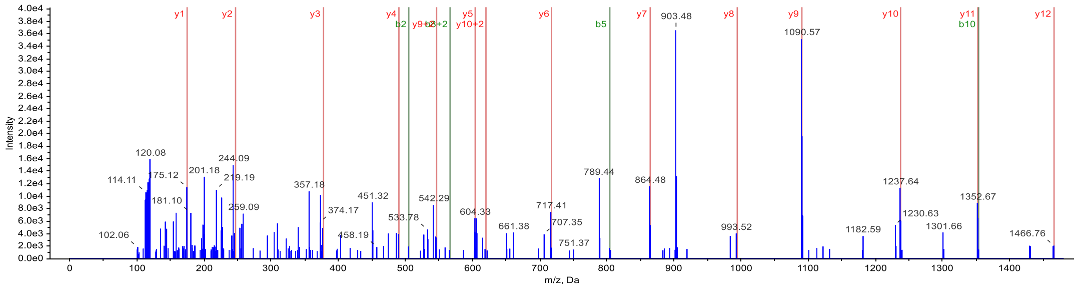

46 Accession No. ABR18064/ gi|148909959

46.1 SLGQNPTEAELQDMIS[Pho]EVDADGNGTIDFPEFLNLMAR

Residue	b	b+2	y	y+2
S	392.2447	196.6260	4436.0253	2218.5163
L	505.3287	253.1680	4044.7879	2022.8976
G	562.3502	281.6787	3931.7038	1966.3555
Q	690.4088	345.7080	3874.6823	1937.8448
N	804.4517	402.7295	3746.6238	1873.8155
P	901.5045	451.2559	3632.5808	1816.7941
T	1002.5521	501.7797	3535.5281	1768.2677
E	1131.5947	566.3010	3434.4804	1717.7438
A	1202.6318	601.8196	3305.4378	1653.2225
E	1331.6744	666.3409	3234.4007	1617.7040
L	1444.7585	722.8829	3105.3581	1553.1827
Q	1572.8171	786.9122	2992.2740	1496.6407
D	1687.8440	844.4257	2864.2155	1432.6114
M[Oxi]	1834.8794	917.9433	2749.1885	1375.0979
I	1947.9635	974.4854	2602.1531	1301.5802
S[Pho]	2114.9618	1057.9846	2489.0690	1245.0382
E	2244.0044	1122.5059	2322.0707	1161.5390
V	2343.0729	1172.0401	2193.0281	1097.0177

Residue	b	b+2	y	y+2
D	2458.0998	1229.5535	2093.9597	1047.4835
A	2529.1369	1265.0721	1978.9327	989.9700
D	2644.1639	1322.5856	1907.8956	954.4515
G	2701.1853	1351.0963	1792.8687	896.9380
N	2815.2282	1408.1178	1735.8472	868.4272
G	2872.2497	1436.6285	1621.8043	811.4058
T[Dhy]	2955.2868	1478.1470	1564.7828	782.8951
I	3068.3709	1534.6891	1481.7457	741.3765
D	3183.3978	1592.2026	1368.6616	684.8345
F	3330.4662	1665.7368	1253.6347	627.3210
P	3427.5190	1714.2631	1106.5663	553.7868
E	3556.5616	1778.7844	1009.5135	505.2604
F	3703.6300	1852.3186	880.4709	440.7391
L	3816.7141	1908.8607	733.4025	367.2049
N	3930.7570	1965.8821	620.3185	310.6629
L	4043.8411	2022.4242	506.2755	253.6414
M[Oxi]	4190.8765	2095.9419	393.1915	197.0994
A	4261.9136	2131.4604	246.1561	123.5817
R	4418.0147	2209.5110	175.1190	88.0631

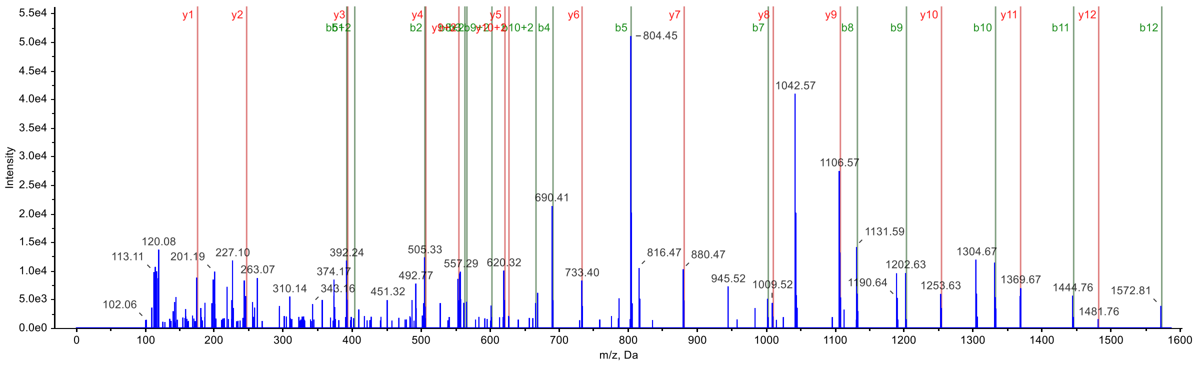

47 Accession No. AAR99410/ gi|41072339

47.1 SLGQNPTEAELQDMINEVDADGNGTIDFPEFLNLT[Pho]AR

Residue	b	b+2	y	y+2
S	392.2447	196.6260	4388.0396	2194.5236
L	505.3287	253.1680	3996.8023	1998.9048
G	562.3502	281.6787	3883.7182	1942.3627
Q	690.4088	345.7080	3826.6967	1913.8520
N	804.4617	402.7295	3698.6382	1849.8227
P	901.5045	451.2559	3584.5952	1792.8013
T	1002.5521	501.7797	3487.6425	1744.2749
E	1131.5947	566.3010	3386.4948	1693.7510
A	1202.6318	601.8196	3257.4522	1629.2297
E	1331.6744	666.3409	3186.4151	1593.7112
L	1444.7686	722.8829	3057.3726	1529.1899
Q	1572.8171	786.9122	2944.2884	1472.6478
D	1687.8440	844.4257	2816.2298	1408.6186
M[DTM]	1770.8811	885.9442	2701.2029	1351.1061
I	1883.9652	942.4862	2618.1658	1309.5865
N[Dea]	1998.9921	999.9997	2505.0817	1253.0445
E	2128.0347	1064.5210	2390.0648	1195.5310
V	2227.1032	1114.0552	2261.0122	1131.0097

Residue	b	b+2	y	y+2
D	2342.1301	1171.5687	2161.9438	1081.4755
A	2413.1672	1207.0872	2046.9168	1023.9621
D	2528.1942	1264.6007	1975.8797	988.4436
G	2585.2156	1293.1114	1860.8528	930.9300
N[Oxi]	2715.2535	1358.1304	1803.8313	902.4193
G	2772.2749	1386.6411	1673.7936	837.4004
T	2873.3226	1437.1649	1616.7720	808.8896
I	2986.4067	1493.7070	1515.7243	758.3658
D	3101.4336	1551.2204	1402.6403	701.8238
F	3248.5020	1624.7546	1287.6133	644.3103
P	3345.5548	1673.2810	1140.5449	570.7761
E	3474.5974	1737.8023	1043.4921	522.2497
F	3621.6658	1811.3365	914.4496	457.7284
L	3734.7499	1867.8786	767.3811	384.1942
N	3848.7928	1924.9000	654.2971	327.6522
L	3961.8768	1981.4421	540.2541	270.6307
T[Pho]	4142.8909	2071.9491	427.1701	214.0887
A	4213.9280	2107.4676	246.1561	123.5817
R	4370.0291	2185.5182	175.1190	88.0631

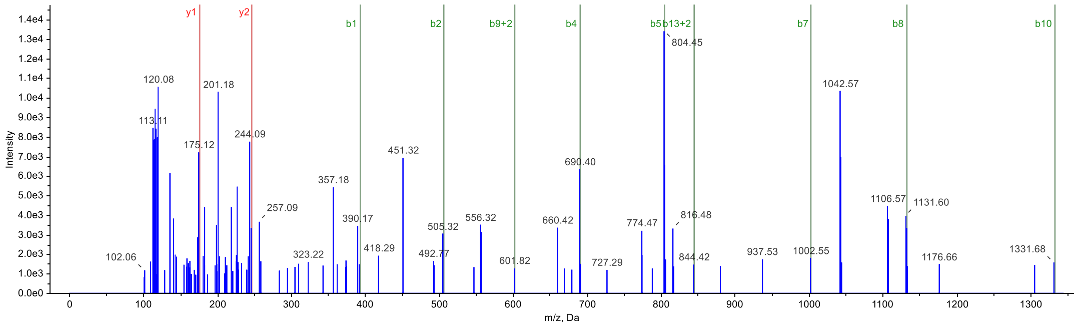

48 Accession No. XP_004970381/ gi|514783586

48.1 EEWT[Pho]AEVFDVDLLR

Residue	b	b+2	y	y+2
E	434.2552	217.6313	2106.0098	1053.5085
E	563.2978	282.1525	1672.7618	836.8846
W	749.3771	375.1922	1543.7192	772.3633
T[Pho]	930.3911	465.6992	1357.6399	679.3236
A	1001.4283	501.2178	1176.6259	588.8166
E	1130.4709	565.7391	1105.5888	553.2980
V	1229.5393	615.2733	976.5462	488.7767
F	1376.6077	688.8075	877.4778	439.2425
D	1491.6346	746.3209	730.4094	365.7083
V	1590.7030	795.8552	615.3824	308.1949
D	1705.7300	853.3686	516.3140	258.6606
L	1818.8140	909.9107	401.2871	201.1472
L	1931.8981	966.4527	288.2030	144.6051
R	2087.9992	1044.5032	175.1190	88.0631

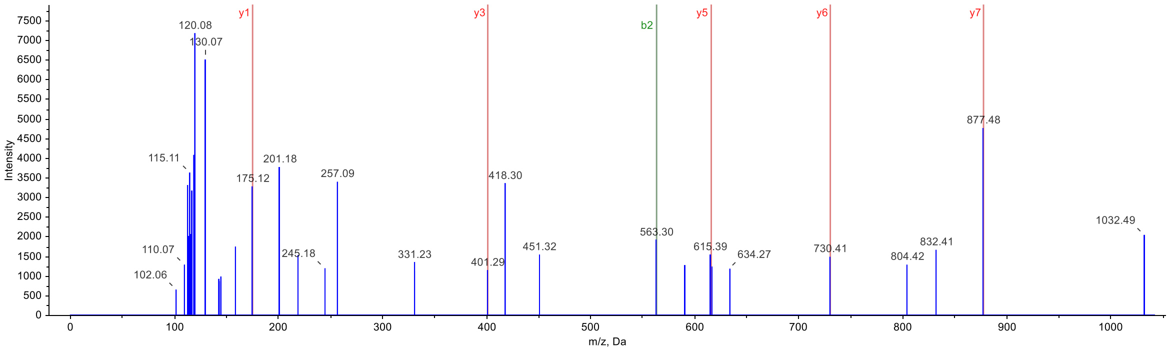

49 Accession No. AAT01376/ gi|46576015

49.1 AS[Pho]AEVLGK

Residue	b	b+2	y	y+2
A	376.2497	188.6285	1462.8126	731.9100
S[Pho]	543.2481	272.1277	1087.5702	544.2887
A	614.2852	307.6462	920.5718	460.7895
E	743.3278	372.1675	849.5347	425.2710
V	842.3962	421.7018	720.4921	360.7497
L	955.4803	478.2438	621.4237	311.2155
G	1012.5018	506.7545	508.3396	254.6735
K[IT8]	1444.8021	722.9047	451.3182	226.1627

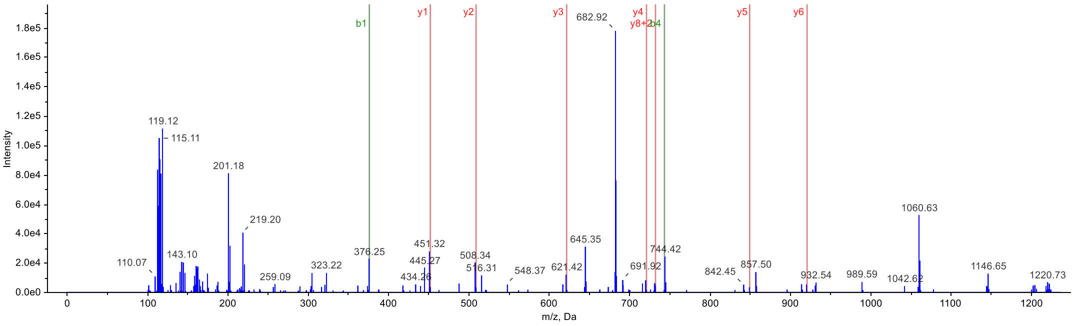

50 Accession No. EMT11740/ gi|475552158

50.1 SSS[Pho]FGQQTSGFQQSDSFKQR

Residue	b	b+2	y	y+2
S	392.2447	196.6260	3006.3492	1503.6782
S	479.2767	240.1420	2615.1118	1308.0595
S[Pho]	646.2751	323.6412	2528.0797	1264.5435
F	793.3435	397.1754	2361.0814	1181.0443
G	850.3649	425.6861	2214.0130	1107.5101
Q	978.4235	489.7154	2156.9915	1078.9994
Q	1106.4821	553.7447	2028.9329	1014.9701
T	1207.5298	604.2685	1900.8744	950.9408
S	1294.5618	647.7845	1799.8267	900.4170
G	1351.5833	676.2953	1712.7946	856.9010
F	1498.6517	749.8295	1655.7732	828.3902
Q[Dea]	1627.6943	814.3508	1508.7048	754.8560
Q	1755.7528	878.3801	1379.6622	690.3347
S	1842.7849	921.8961	1251.6036	626.3054
D[Pho]	2037.7781	1019.3927	1164.5716	582.7894
S	2124.8102	1062.9087	969.5783	485.2928
F	2271.8786	1136.4429	882.5463	441.7768
K[IT8]	2704.1789	1352.5931	735.4779	368.2426
Q	2832.2375	1416.6224	303.1775	152.0924
R	2988.3386	1494.6729	175.1190	88.0631

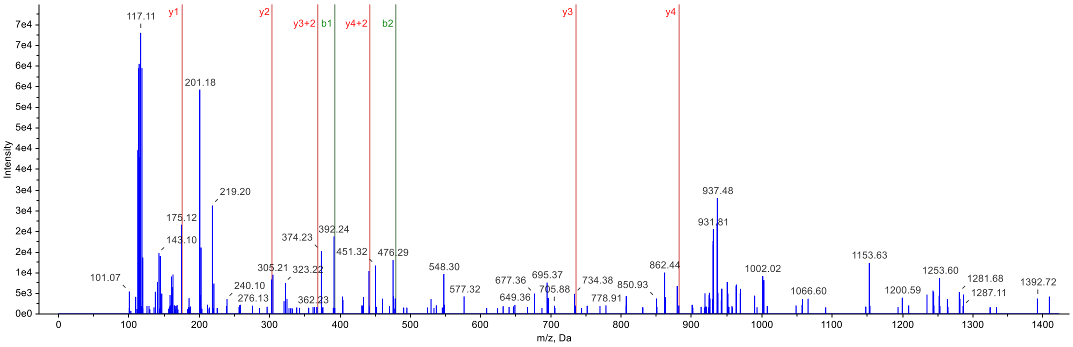

50.2 SSS[Pho]FGQQTSGFQQSDS[Pho]FKQR

Residue	b	b+2	y	y+2
S	392.2447	196.6260	3005.3651	1503.1862
S	479.2767	240.1420	2614.1278	1307.5675
S[Pho]	646.2751	323.6412	2527.0957	1264.0515
F	793.3435	397.1754	2360.0974	1180.5523
G	850.3649	425.6861	2213.0290	1107.0181
Q	978.4235	489.7154	2156.0075	1078.5074
Q	1106.4821	553.7447	2027.9489	1014.4781
T	1207.5298	604.2685	1899.8903	950.4488
S	1294.5618	647.7845	1798.8427	899.9250
G	1351.5833	676.2953	1711.8106	856.4090
F	1498.6517	749.8295	1654.7892	827.8982
Q	1626.7102	813.8588	1507.7208	754.3640
Q	1754.7688	877.8881	1379.6622	690.3347
S	1841.8009	921.4041	1251.6036	626.3054
D	1956.8278	978.9175	1164.5716	582.7894
S[Pho]	2123.8262	1062.4167	1049.5446	525.2760
F	2270.8946	1135.9509	882.5463	441.7768
K[IT8]	2703.1949	1352.1011	735.4779	368.2426
Q	2831.2535	1416.1304	303.1775	152.0924
R	2987.3546	1494.1809	175.1190	88.0631

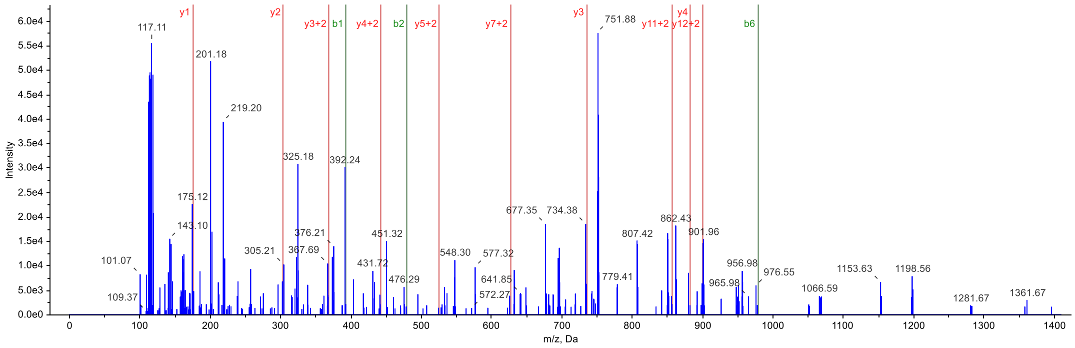

51 Accession No. ABA95233/ gi|77552436

51.1 SLGANLFDRPQPNS[Pho]PTVYDWLYSDETR

Residue	b	b+2	y	y+2
S	392.2447	196.6260	3526.6829	1763.3351
L	505.3287	253.1680	3134.4265	1567.7164
G	562.3502	281.6787	3021.3415	1511.1744
A	633.3873	317.1973	2964.3200	1482.6636
N	747.4302	374.2188	2893.2829	1447.1451
L	860.5143	430.7608	2779.2400	1390.1236
F	1007.5827	504.2950	2666.1559	1333.5816
D	1122.6097	561.8085	2519.0875	1260.0474
R	1278.7108	639.8590	2404.0605	1202.5339
P	1375.7635	688.3854	2247.9594	1124.4833
Q	1503.8221	752.4147	2150.9067	1075.9570
P	1600.8749	800.9411	2022.8481	1011.9277
N	1714.9178	857.9625	1925.7963	963.4013

Residue	b	b+2	y	y+2
S[Pho]	1881.9162	941.4617	1811.7524	906.3798
P	1978.9689	989.9881	1644.7540	822.8807
T	2080.0166	1040.5119	1547.7013	774.3543
V	2179.0850	1090.0461	1446.6536	723.8304
Y	2342.1483	1171.5778	1347.5852	674.2962
D	2457.1753	1229.0913	1184.5218	592.7646
W	2643.2546	1322.1309	1069.4949	535.2511
L	2756.3387	1378.6730	883.4156	442.2114
Y	2919.4020	1460.2046	770.3315	385.6694
S	3006.4340	1503.7206	607.2682	304.1377
D	3121.4610	1561.2341	520.2362	260.6217
E	3250.5036	1625.7554	405.2092	203.1083
T	3351.5512	1676.2793	276.1666	138.5870
R	3507.6523	1754.3298	175.1190	88.0631

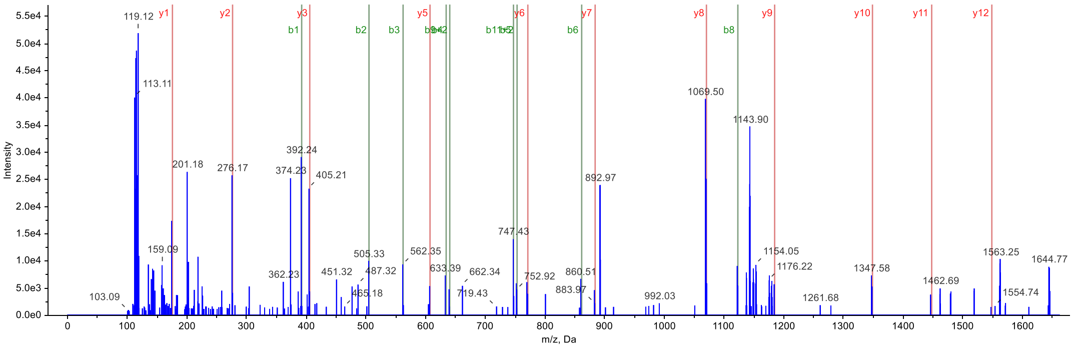

52 Accession No. XP_002465460/ gi|242036131

52.1 GT[Pho]GAPVYLAAVLEYLAAEVLELAGNAAR

Residue	b	b+2	y	y+2
G	58.0287	29.5180	2898.4649	1449.7361
T[Pho]	239.0427	120.0250	2841.4434	1421.2253
G	296.0642	148.5357	2660.4294	1330.7183
A	367.1013	184.0543	2603.4079	1302.2076
P[Oxi]	480.1490	240.5781	2532.3708	1266.6890
V	579.2174	290.1123	2419.3231	1210.1652
Y	742.2807	371.6440	2320.2547	1160.6310
L	855.3648	428.1860	2157.1914	1079.0993
A	926.4019	463.7046	2044.1073	1022.5573
A	997.4390	499.2232	1973.0702	987.0387
V	1096.5075	548.7574	1902.0331	951.5202
L	1209.5915	605.2994	1802.9647	901.9860
E	1338.6341	669.8207	1689.8806	845.4440
Y	1501.6974	751.3524	1560.8380	780.9227
L	1614.7815	807.8944	1397.7747	699.3910
A	1685.8186	843.4129	1284.6906	642.8490
A	1756.8557	878.9315	1213.6535	607.3304
E	1885.8983	943.4528	1142.6164	571.8118
V	1984.9667	992.9870	1013.5738	507.2905
L	2098.0508	1049.5290	914.5054	457.7563
E	2227.0934	1114.0503	801.4213	401.2143
L	2340.1775	1170.5924	672.3787	336.6930
A	2411.2146	1206.1109	559.2947	280.1510
G	2468.2360	1234.6217	488.2576	244.6324
N	2582.2790	1291.6431	431.2361	216.1217
A	2653.3161	1327.1617	317.1932	159.1002
A	2724.3532	1362.6802	246.1561	123.5817
R	2880.4543	1440.7308	175.1190	88.0631

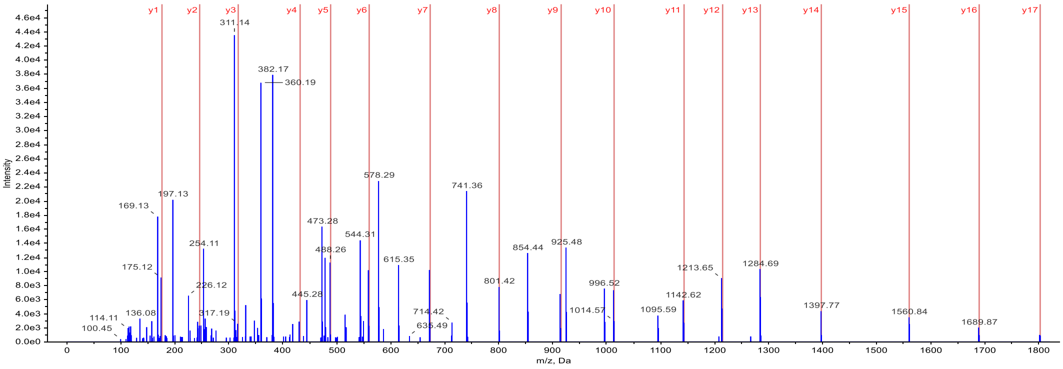

53 Accession No. XP_003569004/gi|357134801

53.1 VGS[Pho]GAPVY[Pho]LAAVLEYLAAEVLELAGNAAR

Residue	b	b+2	y	y+2
V	128.1070	64.5571	3076.5203	1538.2638
G	185.1285	93.0679	2948.4206	1474.7140
S[Pho]	352.1268	176.5670	2891.3992	1446.2032
G	409.1483	205.0778	2724.4008	1362.7040
A	480.1854	240.5963	2667.3793	1334.1933
P	577.2382	289.1227	2596.3422	1298.6748
V	676.3066	338.6569	2499.2895	1250.1484
Y[Pho]	919.3362	460.1718	2400.2211	1200.6142
L	1032.4203	516.7138	2157.1914	1079.0993
A	1103.4574	552.2323	2044.1073	1022.5573
A	1174.4945	587.7509	1973.0702	987.0387
V	1273.5629	637.2851	1902.0331	951.5202
L	1386.6470	693.8271	1802.9647	901.9860
E	1515.6896	758.3484	1689.8806	845.4440
Y	1678.7529	839.8801	1560.8380	780.9227
L	1791.8370	896.4221	1397.7747	699.3910
A	1862.8741	931.9407	1284.6906	642.8490
A	1933.9112	967.4592	1213.6535	607.3304
E	2062.9538	1031.9805	1142.6164	571.8118
V	2162.0222	1081.5147	1013.5738	507.2905
L	2275.1063	1138.0568	914.5054	457.7563
E	2404.1489	1202.5781	801.4213	401.2143
L	2517.2329	1259.1201	672.3787	336.6930
A	2588.2701	1294.6387	559.2947	280.1510
G	2645.2915	1323.1494	488.2576	244.6324
N	2759.3344	1380.1709	431.2361	216.1217
A	2830.3716	1415.6894	317.1932	159.1002
A	2901.4087	1451.2080	246.1561	123.5817
R	3057.5098	1529.2585	175.1190	88.0631

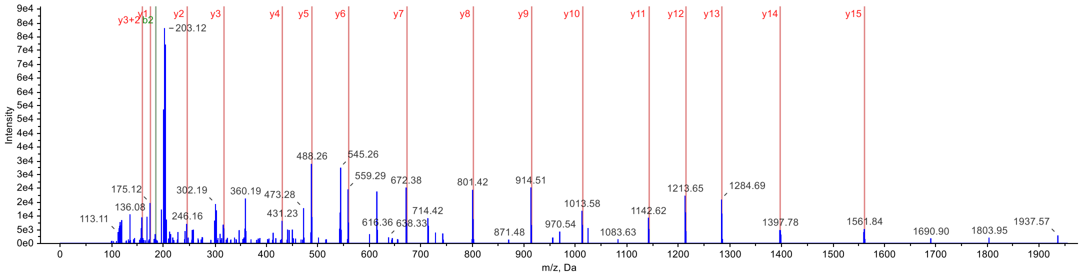

53.2 PVY[Pho]LAAVLEYLAAEVLELAGNAAR

Residue	b	b+2	y	y+2
P	402.2654	201.6363	2900.5476	1450.7774
V	501.3338	251.1705	2499.2895	1250.1484
Y[Pho]	744.3635	372.6854	2400.2211	1200.6142
L	857.4475	429.2274	2157.1914	1079.0993
A	928.4847	464.7460	2044.1073	1022.5573
A	999.5218	500.2645	1973.0702	987.0387
V	1098.5902	549.7987	1902.0331	951.5202
L	1211.6742	606.3408	1802.9647	901.9860
E	1340.7168	670.8621	1689.8806	845.4440
Y	1503.7802	752.3937	1560.8380	780.9227
L	1616.8642	808.9358	1397.7747	699.3910
A	1687.9013	844.4543	1284.6906	642.8490
A	1758.9385	879.9729	1213.6535	607.3304
E	1887.9810	944.4942	1142.6164	571.8118
V	1987.0495	994.0284	1013.5738	507.2905
L	2100.1335	1050.5704	914.5054	457.7563
E	2229.1761	1115.0917	801.4213	401.2143
L	2342.2602	1171.6337	672.3787	336.6930
A	2413.2973	1207.1523	559.2947	280.1510
G	2470.3188	1235.6630	488.2576	244.6324
N	2584.3617	1292.6845	431.2361	216.1217
A	2655.3988	1328.2030	317.1932	159.1002
A	2726.4359	1363.7216	246.1561	123.5817
R	2882.5370	1441.7722	175.1190	88.0631

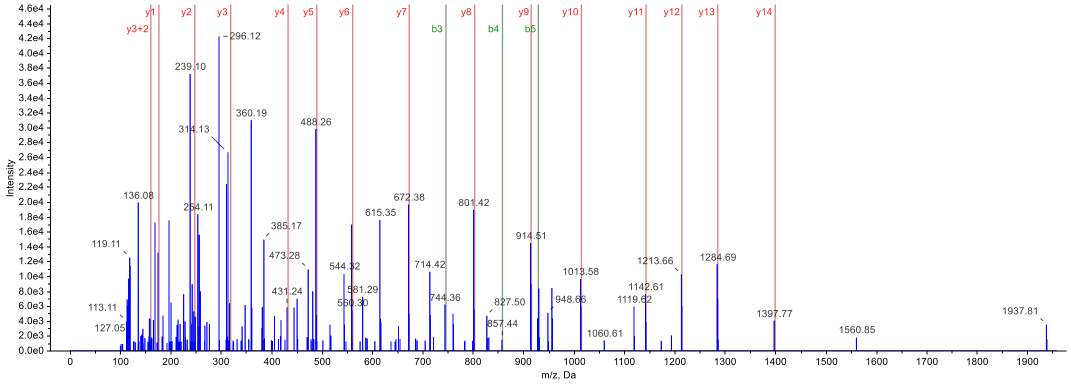

53.3 PVYLAAVLEY[Pho]LAAEVLELAGNAAR

Residue	b	b+2	y	y+2
P	402.2654	201.6363	2900.5476	1450.7774
V	501.3338	251.1705	2499.2895	1250.1484
Y	664.3971	332.7022	2400.2211	1200.6142
L	777.4812	389.2442	2237.1577	1119.0825
A	848.5183	424.7628	2124.0737	1062.5405
A	919.5554	460.2814	2053.0365	1027.0219
V	1018.6238	509.8156	1981.9994	991.5034
L	1131.7079	566.3576	1882.9310	941.9691
E	1260.7505	630.8789	1769.8470	885.4271
Y[Pho]	1503.7802	752.3937	1640.8044	820.9068
L	1616.8642	808.9358	**1397.7747**	699.3910
A	1687.9013	844.4543	**1284.6906**	642.8490
A	1758.9385	879.9729	**1213.6535**	607.3304
E	1887.9810	944.4942	**1142.6164**	571.8118
V	1987.0495	994.0284	**1013.5738**	507.2905
L	2100.1335	1050.5704	**914.5054**	457.7563
E	2229.1761	1115.0917	**801.4213**	401.2143
L	2342.2602	1171.6337	**672.3787**	336.6930
A	2413.2973	1207.1523	**559.2947**	280.1510
G	2470.3188	1235.6630	**488.2576**	244.6324
N	2584.3617	1292.6845	**431.2361**	216.1217
A	2655.3988	1328.2030	**317.1932**	**159.1002**
A	2726.4359	1363.7216	**246.1561**	123.5817
R	2882.5370	1441.7722	**175.1190**	88.0631

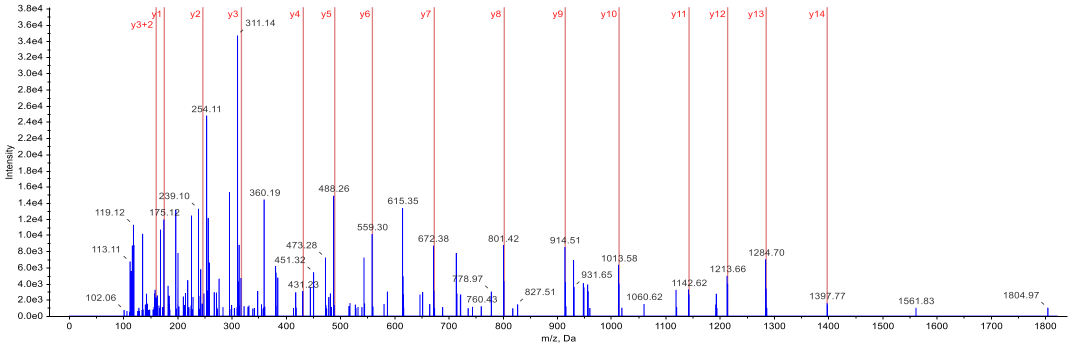

54 Accession No. XP_006479831/gi|568852331

54.1 VGAGAPVYLS[Pho]AVLEYLAAEVLELAGNAAR

Residue	b	b+2	y	y+2
V	**404.2810**	202.6442	3177.6862	1589.3467
G	461.3025	231.1549	2774.4124	1387.7099
A	532.3396	266.6735	2717.3910	1359.1991
G	589.3611	295.1842	2646.3539	1323.6806
A	**660.3982**	330.7027	2589.3324	1295.1698
P	757.4510	**379.2291**	2518.2953	1259.6513
V	**856.5194**	**428.7633**	2421.2425	1211.1249
Y	**1019.5827**	**510.2950**	2322.1741	1161.5907
L	1132.6668	**566.8370**	2159.1108	1080.0590
S[Pho]	1299.6651	650.3362	2046.0267	1023.5170
A	1370.7022	685.8548	1879.0284	940.0178
V	1469.7707	735.3890	1807.9912	904.4993
L	1582.8547	791.9310	1708.9228	854.9651
E	1711.8973	856.4523	1595.8388	798.4230
Y[YDA]	1780.9188	890.9630	1466.7962	733.9017
L	1894.0028	947.5051	**1397.7747**	699.3910
A	1965.0400	983.0236	**1284.6906**	642.8490
A	2036.0771	1018.5422	**1213.6535**	607.3304
E	2165.1197	1083.0635	**1142.6164**	571.8118
V	2264.1881	1132.5977	**1013.5738**	507.2905
L	2377.2721	1189.1397	**914.5054**	457.7563
E	2506.3147	1253.6610	**801.4213**	401.2143
L	2619.3988	1310.2030	**672.3787**	336.6930
A	2690.4359	1345.7216	**559.2947**	280.1510
G	2747.4574	1374.2323	**488.2576**	244.6324
N	2861.5003	1431.2538	**431.2361**	216.1217
A	2932.5374	1466.7723	**317.1932**	**159.1002**

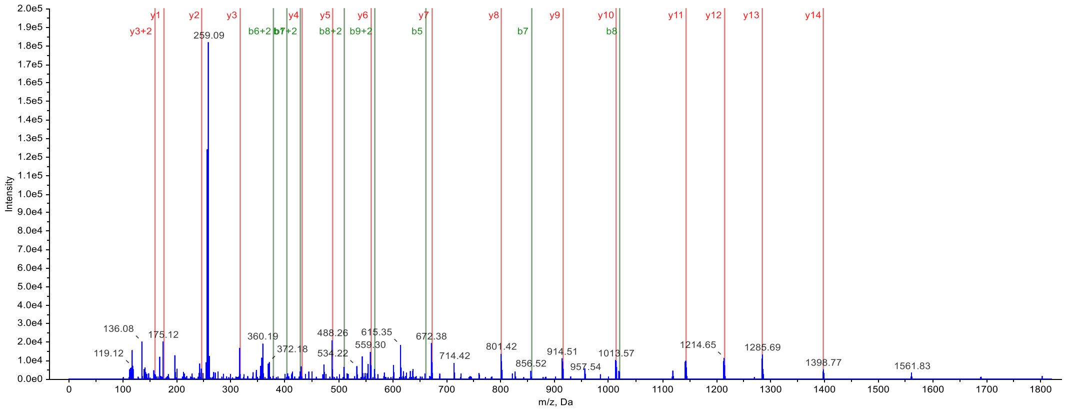

Supplement: Supplementary Figure S9 — Workflow of plant treatment and sampling Seeds of Puccinellia tenuiflora were sowed on vermiculite and grown in Hoagland solution in pots. Seedlings about 50-day-old were treated with 0 mM, 150 mM Na2CO3 for 12 h (12 HAT150), 12 HAT200, 24 HAT150, and 24 HAT200, respectively. After treatment, leaves from both control and treatments were harvested for experiments. [file mmc2.pdf]
