# Supplementary material for: Na2CO3-responsive Photosynthetic and ROS Scavenging Mechanisms in Chloroplasts of Alkaligrass Revealed by Phosphoproteomics
Source: Genomics Proteomics Bioinformatics. 2020 Jul 16;18(3):271–88. doi: 10.1016/j.gpb.2018.10.011 (PMC7801222; doi:10.1016/j.gpb.2018.10.011)
Supplement: Supplementary Table S3 [file mmc5.docx]

**Table S3 Na_2_CO_3_-responsive proteins in alkaligrass leaves**

| **Spot**  **No.** | **Protein name** | **Accession No.** | **Loc** | **Biological function** | **Thr.**  **MM(Da)**  **/p*I*** | **Exp.**  **MM(Da)**  **/p*I*** | **Sco** | **QM** | **V% ± S.D.** |
| --- | --- | --- | --- | --- | --- | --- | --- | --- | --- |
| **Photosynthesis (37)** | | | | | | | | | |
| Chlorophyll *a*/*b* binding protein (6)  **  **  **  ** | | | | | | | | | |
| 2778 | Chlorophyll *a*/*b* binding protein (Lhca1) | ACO06087 | Chl | PSI Light harvesting | 31,427  /6.12 | 23,380  /5.30 | 108 | 2 |  |
| 3117 | Light harvesting chlorophyll *a*/*b* binding protein (Lhca5) | AFS34654 | Chl | PSI Light harvesting | 20,709 /6.34 | 16,059  /4.91 | 121 | 5 | **  **  **  * |
| 2569 | Light harvesting chlorophyll *a*/*b* binding protein (Lhcb1) | ADL41158 | Chl | Light harvesting, state transitions | 28,367/  5.14 | 27,171  /4.86 | 161 | 2 | **  **  **  ** |
| 4491 | Light harvesting chlorophyll *a*/*b* binding protein (Lhcb1) | ADL41158 | Chl | Light harvesting, state transitions | 28,367/  5.14 | 27,323  /4.72 | 81 | 2 | **  **  **  * |
| 4019 | Light harvesting chlorophyll *a*/*b* binding protein of LHCII type II (Lhcb2) | AAC15992 | Chl | Light harvesting, state transitions | 28,564  /5.62 | 27,852  /6.81 | 86 | 2 | **  **  **  ** |
| 4501 | Light harvesting chlorophyll *a*/*b* binding protein CP29, photosystem II (CP29) | CAA44777 | Chl | PSII disassembly, energy dissipation | 30,839  /5.33 | 28,338  /4.83 | 201 | 4 | **  **  ** |
| Photosystem II related protein (5) | | | | | | | | | |
| 2263 | Oxygen-evolving enhancer protein 1 (PsbO) | ABQ52657 | Chl | Photosynthetic oxygen evolution, PSII D1 repair | 34,719  /6.08 | 31,545  /4.92 | 209 | 3 | **  **  **  ** |
| 2264 | Oxygen-evolving enhancer protein 1 (PsbO) | ABQ52657 | Chl | Photosynthetic oxygen evolution, PSII D1 repair | 34,719  /6.08 | 31,049  /5.00 | 350 | 4 | **  **  **  ** |
| 2282 | Oxygen-evolving enhancer protein 1 (PsbO) | ABQ52657 | Chl | Photosynthetic oxygen evolution, PSII D1 repair | 34,719  /6.08 | 31,284  /5.09 | 170 | 3 | **  **  **  ** |
| 3303 | Oxygen-evolving enhancer protein 1 (PsbO) | ABQ52657 | Chl | Photosynthetic oxygen evolution, PSII D1 repair | 34,719  /6.08 | 13,459  /5.68 | 132 | 5 | **  **  ** |
| 4512 | Oxygen-evolving enhancer protein 1 (PsbO) | ABQ52657 | Chl | Photosynthetic oxygen evolution, PSII D1 repair | 34,719  /6.08 | 31,545  /4.89 | 195 | 3 | **  **  *  * |
| Photosynthetic electron transfer chain (2) | | | | | | | | | |
| 4030 | Ferredoxin-NADP(+) reductase (FNR) | CAD30024 | Chl | Electron transport | 39,181  /8.29 | 34,767  /6.95 | 147 | 3 | **  **  **  ** |
| 4066 | Ferredoxin-NADP(+) reductase (FNR) | AAA34029 | Chl | Electron transport | 41,399  /8.67 | 36,321  /6.35 | 140 | 2 | **  **  **  ** |
| Calvin cycle (22) | | | | | | | | | |
| 4006 | Predicted protein, containing cd00884, carbonic anhydrase domain (CA)* | BAK00501 | Chl | Catalyze the hydration of CO_2_ to form bicarbonate | 25,214  /8.04 | 24,844  /6.68 | 117 | 2 | **  **  **  ** |
| 4008 | Carbonic anhydrase isoform X1 (CA) | XP_010232070 | Chl | Catalyze the hydration of CO_2_ to form bicarbonate | 28,104  /8.34 | 24,904  /6.72 | 284 | 5 | **  **  **  ** |
| 4196 | Carbonic anhydrase isoform X1 (CA) | XP_010232070 | Chl | Catalyze the hydration of CO_2_ to form bicarbonate | 28,104  /8.34 | 24,450  /6.22 | 221 | 3 | **  **  * |
| 4233 | Carbonic anhydrase isoform X1 (CA) | XP_010232070 | Chl | Catalyze the hydration of CO_2_ to form bicarbonate | 28,104  /8.34 | 25,427  /5.81 | 221 | 4 | **  * |
| 1698 | Ribulose-1,5-bisphosphate carboxylase activase A isoform X1 (RCA) | XP_010237358 | Chl | Activate RuBisCO | 52,371  /5.68 | 42,408  /5.41 | 380 | 4 | **  **  ** |
| 1438 | Ribulose-1,5-bisphosphate carboxylase activase (RCA) | AAP72270 | Chl | Activate RuBisCO | 22,493  /4.98 | 50,539  /5.05 | 163 | 2 | **  **  * |
| 1702 | Ribulose-1,5-bisphosphate carboxylase activase (RCA) | XP_010037804 | Chl | Activate RuBisCO | 49,294  /6.4 | 42,610  /5.55 | 310 | 4 | **  **  *  * |
| 1065 | RuBisCO large subunit-binding protein alpha subunit (RBP) | XP_003558045 | Chl | Assemble RuBisCO | 61,688  /5.31 | 67,272  /5.09 | 89 | 2 | **  **  **  ** |
| 974 | RuBisCO large subunit-binding protein beta subunit (RBP) | EMS68298 | Chl | Assemble RuBisCO | 83,354  /7.79 | 72,116  /5.82 | 412 | 8 | **  **  **  ** |
| 3120 | Ribulose-1,5-bisphosphate carboxylase small chain PW9 (RBS) | XP_003573910 | Chl | Combines CO_2_ to produce 3-phosphoglycerate | 19,728  /8.97 | 15,995  /5.65 | 139 | 3 | **  **  **  ** |
| 3137 | Ribulose-1,5-bisphosphate carboxylase small chain PW9 (RBS) | XP_003573910 | Chl | Combines CO_2_ to produce 3-phosphoglycerate | 19,728  /8.97 | 15,197  /6.30 | 159 | 3 | **  **  **  ** |
| 3147 | Ribulose-1,5-bisphosphate carboxylase small chain PW9 (RBS) | XP_003573910 | Chl | Combines CO_2_ to produce 3-phosphoglycerate | 19,728  /8.97 | 15,442  /5.71 | 87 | 2 | **  **  **  ** |
| 3231 | Ribulose-1,5-bisphosphate carboxylase small chain PW9 (RBS) | XP_003573910 | Chl | Combines CO_2_ to produce 3-phosphoglycerate | 19,728  /8.97 | 14,347  /5.25 | 108 | 3 | **  **  * |
| 3232 | Ribulose-1,5-bisphosphate carboxylase small chain PW9 (RBS) | XP_003573910 | Chl | Combines CO_2_ to produce 3-phosphoglycerate | 19,728  /8.97 | 14,474  /6.30 | 91 | 2 | **  **  ** |

Table S3 *(continued from previous page.)*

| **Spot**  **no.** | **Protein name** | **Accession no.** | **Loc** | **Biological function** | **Thr.**  **MM(Da)**  **/p*I*** | **Exp.**  **MM(Da)**  **/p*I*** | **Sco** | **QM** | **V% ± S.D.** |
| --- | --- | --- | --- | --- | --- | --- | --- | --- | --- |
| 1866 | Glyceraldehyde 3-phosphate dehydrogenase subunit A (GAPDH) | ABD37955 | Chl | Reversibly converts 1,3-BPG to GAP | 41,524  /9.15 | 38,929  /6.56 | 127 | 3 | **  **  **  ** |
| 1917 | Glyceraldehyde 3-phosphate dehydrogenase A (GAPDH) | XP_003579898 | Chl | Reversibly converts 1,3-BPG to GAP | 43,1110  /7.01 | 38,001  /6.72 | 322 | 5 | **  **  **  ** |
| 1634 | Predicted protein, glyceraldehyde 3-phosphate dehydrogenase B (GAPDH)* | BAJ86633 | Chl | Reversibly converts 1,3-BPG to GAP | 47,357  /5.9 | 44,397  /5.81 | 255 | 4 | **  **  ** |
| 1649 | Predicted protein, glyceraldehyde 3-phosphate dehydrogenase B (GAPDH)* | BAJ86633 | Chl | Reversibly converts 1,3-BPG to GAP | 47,357  /5.9 | 43,978  /6.01 | 142 | 3 | **  **  **  ** |
| 4109 | Predicted protein, glyceraldehyde 3-phosphate dehydrogenase B (GAPDH)* | BAJ86633 | Chl | Reversibly converts 1,3-BPG to GAP | 47,357  /5.9 | 28,628  /6.62 | 432 | 7 | **  **  **  * |
| 4403 | Triosephosphate isomerase (TPI) | P46225 | Chl | Reversible converts DHAP to GAP | 31,955  /6 | 27,258  /5.38 | 139 | 2 | **  ** |
| 2626 | Ribulose-phosphate 3-epimerase (RPE) | XP_003558725 | Chl | Reversible converts R5P to Ru5P | 29,438  /7.7 | 26,148  /6 | 221 | 2 | **  **  ** |
| 1748 | Phosphoribulokinase (PRK) | CAA41020 | Chl | Catalyze the formation of RuBP | 45,406  /5.84 | 41,089  /5.03 | 317 | 6 | **  **  **  ** |
| Photorespiration (2) | | | | | | | | | |
| 3879 | Phosphoglycolate phosphatase 1B (PGP) | XP_006652446 | Chl | Photorespiration, catalyze the generation of glycolate | 39,724  /6.02 | 33,355  /5.02 | 178 | 5 | **  **  **  ** |
| 3235 | Glycine decarboxylase (GDC) | AAM92707 | Mit | Photorespiration, convert glycine to THF | 21,578  /4.99 | 14,359  /4.60 | 143 | 2 | **  **  ** |
| **Carbohydrate and energy metabolism (22)** | | | | | | | | | |
| Energy metabolism (11) | | | | | | | | | |
| 1027 | ATP synthase CF1 α subunit | AAU06224 | ^#^Chl | ATP synthesis | 55,549  /6.03 | 68,995  /5.86 | 198 | 5 | **  **  ** |
| 4274 | ATP synthase CF1 α subunit | YP_007026550 | ^#^Chl | ATP synthesis | 55,761  /6.11 | 31,308  /5.43 | 699 | 10 | **  **  * |
| 1103 | ATP synthase CF1 α subunit | YP_007026550 | ^#^Chl | ATP synthesis | 55,761  /6.11 | 65,180  /5.97 | 455 | 8 | **  **  **  ** |
| 1109 | ATP synthase CF1 α subunit | YP_007026550 | ^#^Chl | ATP synthesis | 55,761  /6.11 | 64,974  /6.13 | 464 | 8 | **  **  *  * |
| 1171 | ATP synthase CF1 β subunit | CAA25114 | ^#^Chl | ATP synthesis | 53,842  /5.11 | 62,853  /5.13 | 236 | 6 | **  **  **  ** |
| 1195 | ATP synthase CF1 β subunit | NP_114266 | ^#^Chl | ATP synthesis | 53,881  /5.06 | 61,770  /5.44 | 755 | 12 | **  **  **  * |
| 1140 | ATP synthase CF1 β subunit | NP_039390 | ^#^Chl | ATP synthesis | 54,037  /5.47 | 63,955  /5.38 | 176 | 7 | **  **  **  ** |
| 1179 | ATP synthase CF1 β subunit | ABH02572 | ^#^Chl | ATP synthesis | 52,989  /5.16 | 62,556  /5.28 | 238 | 6 | **  **  **  **  **  **  **  ** |
| 1193 | ATP synthase CF1 β subunit | ABH02572 | ^#^Chl | ATP synthesis | 52,989  /5.16 | 61,284  /5.24 | 239 | 6 |  |
| 1220 | ATP synthase β subunit | CAA52636 | Chl, Mit | ATP synthesis | 59,326  /5.56 | 60,419  /5.50 | 523 | 9 | **  **  **  ** |
| 1817 | ATP synthase γ subunit | EMS56225 | ^#^Chl | ATP synthesis | 52,111  /7.52 | 39,700  /6.06 | 56 | 2 | **  **  **  ** |
| Mitochondrion respiratory chain (2) | | | | | | | | | |
| 988 | Carbonic anhydrase-like 2 gamma subunit (γCA) | XP_010235460 | Mit | Mitochondria respiration | 27,782  /8.29 | 70,094  /5.61 | 257 | 5 | **  **  **  * |
| 4212 | Carbonic anhydrase-like 2 gamma subunit (γCA) | XP_010235460 | Mit | Mitochondria respiration | 27,782  /8.29 | 29,236  /6.49 | 115 | 4 | **  **  *  * |
| TCA cycle (4) | | | | | | | | | |
| 4547 | Dihydrolipoyl dehydrogenase, mitochondrial (DLD) | XP_003568861 | Mit | Convert dihydrolipoic acid and NAD+ into lipoic acid and NADH | 52,727  /6.93 | 66,009  /6.97 | 116 | 3 | **  **  **  ** |
| 1572 | Os01g0654500, isocitrate dehydrogenase (IDH)* | NP_001043749 | Cyt, Mit | Convert isocitrate to 2-oxo-glutarate | 46,356  /6.34 | 46,479  /6.35 | 72 | 4 | **  **  **  ** |

Table S3 *(continued from previous page.)*

| **Spot**  **no.** | **Protein name** | **Accession no.** | **Loc** | **Biological function** | **Thr.**  **MM(Da)**  **/p*I*** | **Exp.**  **MM(Da)**  **/p*I*** | **Sco** | **QM** | **V% ± S.D.** |
| --- | --- | --- | --- | --- | --- | --- | --- | --- | --- |
| 910 | Succinate dehydrogenase flavoprotein subunit (SDH) | XP_002310225 | Chl, Mit | Catalyzes the oxidation of succinate to fumarate | 70,557  /6.4 | 75,737  /6.26 | 220 | 4 | **  **  **  ** |
| 1982 | Malate dehydrogenase (MDH) | XP_003567846 | Mit | Catalyzes the oxidation of malate to oxaloacetate | 35,600  /8.54 | 37,067  /6.79 | 99 | 3 | **  **  **  ** |
| Glycolysis (3) | | | | | | | | | |
| 1778 | Predicted protein, fructose-bisphosphate aldolase (FBA)* | BAJ85287 | Cyt | Reversibly converts FBP to DHAP and GAP | 38,102  /6.06 | 40,063  /6.84 | 191 | 3 | **  **  **  ** |
| 1862 | Glyceraldehyde 3-phosphate dehydrogenase (GAPDH) | AFS33113 | Cyt | Reversibly converts 1,3-BPG to GAP | 36,801  /6.4 | 38,870  /6.85 | 256 | 4 | **  **  ** |
| 4287 | Glyceraldehyde 3-phosphate dehydrogenase 2 (GAPDH) | P08477 | Cyt | Reversibly converts 1,3-BPG to GAP | 33,443  /6.2 | 31,760  /5.14 | 440 | 6 | **  **  ** |
| Pentose phosphate pathway (1) | | | | | | | | | |
| 3185 | Predicted protein, containing pfam03446, 6-phosphogluconate dehydrogenase NAD binding domain (6PGD)* | BAJ91168 | ^#^Chl, Cyt | Catalyzes the decarboxylating reduction of 6PG into Ru5P | 30,560  /5.97 | 15,185  /4.97 | 103 | 2 | **  **  **  ** |
| Mannitol biosynthesis (1) | | | | | | | | | |
| 4068 | NADPH-dependent mannose 6-phosphate reductase (M6PR) | BAD07953 | Cyt | Reversibly converts mannose 6-phosphate to mannitol 1-phosphate | 35,388  /5.88 | 35,401  /6.37 | 77 | 2 | **  **  ** |
| **Other metabolisms (17)** | | | | | | | | | |
| Chlorophyll metabolism (5) | | | | | | | | | |
| 1695 | Glutamate-1-semialdehyde 2,1-aminomutase (GSA-AT) | P18492 | Chl | Converts GSA to ALA | 49,690  /6.39 | 42,880  /5.87 | 391 | 7 | **  **  ** |
| 1818 | Uroporphyrinogen decarboxylase (UROD) | Q42855 | Chl | Converts URO III to Coprogen III | 36,758  /5.84 | 39,670  /6.36 | 125 | 2 | ** |
| 4448 | Coproporphyrinogen-III oxidase (CPOX) | EMS67249 | Chl | Catalyze the formation of protoporphyrinogen IX | 34,864  /6.02 | 37,012  /5.73 | 396 | 6 | **  **  **  ** |
| 1699 | Magnesium-protoporyphyrin IX chelatase ChlI subunit (MgCh) | EAY90669 | Chl | Insertion of Mg^2+^ into protoporphyrin IX | 44,823  /5.51 | 42,812  /5.03 | 122 | 4 | **  **  **  ** |
| 4369 | Magnesium-protoporyphyrin IX chelatase ChlI subunit (MgCh) | XP_003557120 | Chl | Insertion of Mg^2+^ into protoporphyrin IX | 45,420  /5.9 | 29,455  /5.80 | 434 | 9 | **  **  **  ** |
| Nitrogen metabolism (2) | | | | | | | | | |
| 4517 | Ferredoxin-nitrite reductase (NiR) | BAD53072 | Chl | Catalyzes the assimilation of nitrite to NH_4_^+^ | 70,256  /6.88 | 73,033  /6.27 | 84 | 3 | **  **  * |
| 4534 | Ferredoxin-nitrite reductase (NiR) | BAD53072 | Chl | Catalyzes the assimilation of nitrite to NH_4_^+^ | 70,256  /6.88 | 73,265  /6.41 | 84 | 3 | **  **  **  ** |
| Nucleotide metabolism (2) | | | | | | | | | |
| 3013 | Unknown, containing cd04413, nucleoside diphosphate kinase group I-like domain (NDPK)* | ACN31682 | Chl, Mit | Catalyzes the production of (d)NTPs; Oxidative stress response | 26,014  /9.04 | 17,845  /6.63 | 149 | 3 | **  **  **  ** |
| 3080 | Os12g0548300, nucleoside diphosphate kinase 2 (NDPK)* | NP_001066971 | Chl | Catalyzes the production of (d)NTPs; Oxidative stress response | 23,626  /9.51 | 16,700  /5.53 | 94 | 2 | **  **  **  ** |
| Amino acid metabolism (6) | | | | | | | | | |
| 1599 | Aspartate aminotransferase (AAT) | EMS51671 | Chl | Catalyzes the formation of aspartate and α-ketoglutarate | 52,199  /6.77 | 45,750  /5.85 | 178 | 4 | **  **  **  ** |
| 1781 | Glutamine synthetase (GS) | S18603 | Cyt | Catalyzes glutamate and NH_4_^+^ to form glutamine | 40,932  /5.4 | 40,254  /5.5 | 136 | 3 | **  **  **  ** |
| 1007 | Predicted protein, containing cd07940, 2-isopropylmalate synthase, N-terminal catalytic TIM barrel domain (IPMS)* | BAJ93533 | Chl | Reversibly convert acetyl-CoA and 3-methyl-2-oxobutanoate to (2S)-2-isopropylmalate | 67,880  /6.28 | 70,316  /5.72 | 173 | 7 | **  **  **  ** |
| 1503 | S-adenosylmethionine synthetase (SAMS) | CAJ01702 | Cyt | Participate in lignin biosynthesis | 43,243  /5.49 | 48,505  /5.93 | 283 | 4 | **  **  **  ** |
| 1178 | S-adenosyl-L-homocysteine hydrolase (SAHH) | CAJ01706 | Cyt | Reversible hydration of SAH into homocysteine | 49,960  /5.81 | 62,359  /5.91 | 388 | 5 | **  **  **  ** |
| 3873 | Cysteine synthase (CSase) | EMS61355 | Chl | Catalyzes the formation of cysteine | 98,315  /5.04 | 35,804  /5.09 | 445 | 7 | **  **  ** |
| Formate metabolism (1) | | | | | | | | | |
| 1664 | Predicted protein, formate dehydrogenase (FDH)* | BAJ95739 | Mit | Catalyzes the oxidation of formate into CO_2_ | 41,693  /6.51 | 43,770  /6.95 | 262 | 5 | **  **  **  ** |

Table S3 *(continued from previous page)*

| **Spot**  **no.** | **Protein name** | **Accession no.** | **Loc** | **Biological function** | **Thr.**  **MM(Da)**  **/p*I*** | **Exp.**  **MM(Da)**  **/p*I*** | **Sco** | **QM** | **V% ± S.D.** |
| --- | --- | --- | --- | --- | --- | --- | --- | --- | --- |
| Fatty acid metabolism (1) | | | | | | | | | |
| 1446 | ATP citrate lyase A-1 (ACL) | NP_172537 | Cyt | Involved in fatty acid biosynthesis | 46,991  /5.39 | 50,300  /5.45 | 61 | 2 | **  **  **  ** |
| **Stress and defense (4)** | | | | | | | | | |
| 4149 | Manganese superoxide dismutase (MnSOD) | AAA33512 | Mit | ROS scavenging | 25,563  /7.11 | 24,944  /6.35 | 124 | 2 | **  ** |
| 2618 | Predicted protein, containing cd03187, glutathione S-transferase C-terminal alpha helical domain (GST)* | BAJ85232 | Cyt | ROS scavenging | 25,155  /5.51 | 26,400  /6.30 | 73 | 2 | **  **  **  ** |
| 4288 | Glyoxalase I (GLO I) | AAS98483 | Cyt | Catalyzes methylglyoxal detoxification | 29,720  /4.99 | 32,315  /5.16 | 82 | 3 | **  **  **  ** |
| 4359 | Glyoxalase I (GLO I) | AAS98483 | Cyt | Catalyzes methylglyoxal detoxification | 29,720  /4.99 | 26,933  /5.56 | 171 | 6 | **  **  **  ** |
| **Membrane and transporting (5)** | | | | | | | | | |
| 870 | Vacuolar proton-ATPase subunit A (VHA-A) | ABD85016 | Cyt | Sequestrate Na^+^ into the vacuole | 68,754  /5.23 | 78,292  /5.43 | 272 | 10 | **  **  **  ** |
| 876 | Vacuolar proton-ATPase subunit A (VHA-A) | ABD85016 | Cyt | Sequestrate Na^+^ into the vacuole | 68,754  /5.23 | 78,168  /5.46 | 243 | 9 | **  **  *  * |
| 1191 | Vacuolar proton ATPase subunit B (VHA-B) | NP_195563 | Cyt | Sequestrate Na^+^ into the vacuole | 54,271  /5.03 | 62,162  /5.19 | 96 | 5 | **  **  **  ** |
| 4035 | Vacuolar proton ATPase subunit E (VHA-E) | AAD10335 | Cyt | Sequestrate Na^+^ into the vacuole | 26,359  /6.57 | 31,403  /6.99 | 207 | 5 | **  **  * |
| 4286 | Vacuolar proton ATPase subunit E (VHA-E) | AAD10335 | Cyt | Sequestrate Na^+^ into the vacuole | 26,359  /6.57 | 30,511  /5.13 | 337 | 5 | **  **  **  * |
| **Signaling (2)** | | | | | | | | | |
| 3930 | 14-3-3-like protein (14-3-3) | AAP48904 | Cyt | Signal transduction | 28,864  /4.79 | 30,449  /4.78 | 108 | 2 | **  **  * |
| 3932 | 14-3-3-like protein (14-3-3)* | BAB11739 | Cyt | Signal transduction | 29,491  /4.75 | 31,808  /4.78 | 162 | 3 | **  **  **  * |
| **Protein synthesis and turnover (10)** | | | | | | | | | |
| 1602 | 30S ribosomal protein S1 (30S RP) | ABF95619 | Chl | Protein synthesis | 43,282  /4.7 | 45,462  /4.57 | 129 | 3 | **  **  **  ** |
| 2921 | Eukaryotic translation initiation factor 5A1 (eIF5A) | AAZ95171 | Cyt | Protein synthesis | 17,580  /5.7 | 19,768  /5.67 | 70 | 3 | **  **  **  ** |
| 2927 | Eukaryotic translation initiation factor 5A1 (eIF5A) | AAZ95171 | Cyt | Protein synthesis | 17,983  /5.45 | 19,705  /5.38 | 90 | 3 | **  **  **  ** |
| 1498 | Elongation factor Tu (EF-Tu) | Q43467 | Chl | Protein synthesis | 52,177  /6.21 | 48,352  /5.32 | 68 | 2 | **  **  ** |
| 3815 | Predicted protein, nascent polypeptide associated complex subunit alpha-like protein 1 (NACA)* | XP_001785878 | Nuc | Protein folding | 21,592  /4.36 | 29,635  /4.25 | 155 | 5 | **  **  **  * |
| 782 | Heat shock cognate 70 kDa protein 2-like (Hsc70) | XP_006664117 | Cyt | Protein folding | 71,546  /5.09 | 83,532  /5.23 | 262 | 6 | **  **  **  ** |
| 905 | ATP-dependent zinc metalloprotease FtsH 2 (FtsH) | EMS55427 | Chl | Degradation of photodamaged D1 protein | 71,987  /5.7 | 75,856  /5.25 | 696 | 10 | **  **  **  ** |
| 1588 | Proteasome subunit alpha type-3 (Proteasome α3) | XP_004970311 | Cyt, Nuc | Protein degradation | 27,465  /5.75 | 45,967  /5.28 | 105 | 3 | **  **  **  ** |
| 1013 | Predicted protein, proteasome subunit alpha type-6 (Proteasome α6)* | BAJ85935 | Nuc | Protein degradation | 27,547  /6.33 | 69,983  /5.28 | 166 | 3 | **  **  *  * |
| 4020 | Proteasome subunit alpha type-6 (Proteasome α6) | Q9LSU3 | Nuc | Protein degradation | 27,670  /6.19 | 27,919  /6.86 | 35 | 2 | **  **  ** |
| **Cell wall metabolism (2)** | | | | | | | | | |
| 3283 | Predicted protein, caffeoyl-CoA O-methyltransferase (CCoAOMT)* | BAK01687 | ^#^Chl, Cyt | Lignin biosynthesis | 29,520  /5.09 | 13,996  /4.60 | 111 | 3 | **  **  **  ** |
| 4281 | Predicted protein, caffeoyl-CoA O-methyltransferase (CCoAOMT)* | BAK01687 | ^#^Chl, Cyt | Lignin biosynthesis | 29,520  /5.09 | 28,052  /5.27 | 215 | 3 | **  ** |
| **Cell cycle (1)** | | | | | | | | | |
| 4228 | Predicted protein, containing pfam00415, regulator of chromosome condensation repeat (RCC1)* | BAJ88198 | ^#^Nuc | Cell cycle | 47,167  /5.35 | 22,143  /5.90 | 394 | 8 | **  **  ** |

Table S3 *(continued from previous page.)*

| **Spot**  **no.** | **Protein name** | **Accession no.** | **Loc** | **Biological function** | **Thr.**  **MM(Da)**  **/p*I*** | **Exp.**  **MM(Da)**  **/p*I*** | **Sco** | **QM** | **V% ± S.D.** |
| --- | --- | --- | --- | --- | --- | --- | --- | --- | --- |
| **Miscellaneous and function unknown (4)** | | | | | | | | | |
| 4062 | Unknown, containing cd00200, WD40 domain (WD40)* | ACL54313 | Cyt | Miscellaneous | 36,700  /6.13 | 36,540  /6.46 | 142 | 3 | **  **  **  ** |
| 4581 | Os07g0212200, containing cd05265, atypical short-chain dehydrogenase/reductase domain (SDR)* | NP_001059177 | Chl | Function unknown | 40,983  /7.68 | 38,841  /5.84 | 77 | 4 | **  **  **  ** |
| 987 | Os07g0212200, containing cd05265, atypical short-chain dehydrogenase/reductase domain (SDR)* | NP_001059177 | Chl | Function unknown | 41,268  /7.68 | 71,210  /5.25 | 112 | 4 | **  **  **  ** |
| 1072 | Uncharacterized protein | XP_003573626 | Chl | Function unknown | 33,171  /8.64 | 66,849  /5.48 | 193 | 4 | **  **  **  ** |

*Note:* Proteins were identified by MALDI TOF-TOF mass spectrometry and searched by NCBInr green plant database, protein names marked with an asterisk (*) have been edited by us depending on searching against NCBI non-redundant protein database for functional domain. Protein subcellular localization (Loc) predicted by softwares (YLoc, LocTree3, Plant-mPLoc, ngLOC, and ChloroP). Only the consistent predictions from at least two tools were accepted as a confident result. Pounds (#) indicate the subcellular localizations were predicted based on literatures listed in Supplemental Table S6. Chl, chloroplast; Cyt, cytoplasm; Mit, mitochondria; Nuc, nucleus. Experimental (Exp.) molecular mass (Da) were calculated using ImageMaster 2D platinum software. Theoretical (Thr.) molecular mass (Da) were retrieved from the protein database. The mean values of protein spot volumes relative to total volume of all the spots (V%). Error bar indicates ± standard deviation (S.D.). The asterisks indicate significant differences (Student’s *t* test, *, P < 0.05; **, P < 0.01). Abbreviations: 1,3-BPG, 1,3-bisphosphoglyceric acid; 6PG, 6-phosphogluconate; ALA, 5-aminolaevulinic acid; Coprogen III, corproporphyrinogen III; DHAP, dihydroxyacetone phosphate; FBP, fructose 1,6-bisphosphate; GAP, glyceraldehyde 3-phosphate; GSA, glutamate 1-semialdehyde aminotransferase; MM, molecular mass; p*I*, isoelectricpoint; R5P, ribose 5-phosphate; ROS, reactive oxygen species; Ru5P, ribulose-5-phosphate; RuBP, ribulose-1,5-bisphosphate; THF, N^5^,N^10^-methylene tetrahydrofolate; URO III, uroporphyrinogen III.
