# Supplementary material for: Na2CO3-responsive Photosynthetic and ROS Scavenging Mechanisms in Chloroplasts of Alkaligrass Revealed by Phosphoproteomics
Source: Genomics Proteomics Bioinformatics. 2020 Jul 16;18(3):271–88. doi: 10.1016/j.gpb.2018.10.011 (PMC7801222; doi:10.1016/j.gpb.2018.10.011)
Supplement: Supplementary Table S8 [file mmc10.docx]

| **Protein name** | **Accession No.** | **Loc** | **Biological function** | **Peptide sequence (m/z, charge, score)** | **Ratio of phosphopeptide abundance (mean ± S.D.)** | |
| --- | --- | --- | --- | --- | --- | --- |
|  |  |  |  |  | **150 mM/0 mM** | **200 mM/0 mM** |
| **Photosynthesis (25)** | | | | | | |
| **Chlorophyll *a*/*b* binding protein (11)** | | | | | | |
| Light harvesting complex I chlorophyll *a*/*b* binding protein (Lhca2) | CAA59049 | Chl | Light harvesting | APERPIWFPG**S^57^**TPPPWLDG**S^66^**LPGDFGFDPWGLGSDPESLR (1166.01, 4, 12) | 0.66±0.08 | 0.60±0.04 |
| Light harvesting complex I chlorophyll *a*/*b* binding protein (Lhca5) | XP_003618083 | Chl | Light harvesting | QSL**S^66^**YLDGSLPGDFGFDPLGLSDPEGTGGFIEPR (1195.87, 3, 16) | 1.83±0.01 | 1.22±0.28 |
| Light harvesting chlorophyll *a*/*b* binding protein of LHCII type 1-like (Lhcb1) | CDI44335 | Chl | Light harvesting, state transition | VAGGPLGEVVDPLYPGG**S^193^**LDPLGLADDPEAFAELK (746.06, 6, 13) | 1.34±0.15 | 1.65±0.20 |
| Light harvesting chlorophyll *a*/*b* binding protein of LHCII type 1-like (Lhcb1) | EMS50795 | Chl | Light harvesting, state transition | VLYLGPLSGEPP**S^68^**YLTGEFPGDYGWDTAGLSADPETFAK (1246.35, 4, 12) | 1.93±0.34 | 1.78±0.32 |
|  |  |  |  | VLYLGPLSGEPP**S^68^**YL**T^71^**GEFPGDYGWD**T^82^**AGLSADPETFAK (1246.32, 4, 14) | 0.44±0.05 | 0.42±0.04 |
| Light harvesting chlorophyll *a*/*b* binding protein of LHCII type 1-like (Lhcb1) | EMT11232 | Chl | Light harvesting, state transition | VLYLGPL**S^63^**GDPP**S^68^**YLTGEFPGDYGWD**T^82^**AGLSADPETFAK (1246.82, 4, 12) | 0.36±0.16 | 0.47±0.13 |
| Light harvesting chlorophyll *a*/*b* binding protein of LHCII type 1-like (Lhcb1) | EMT29003 | Chl | Light harvesting, state transition | VLYLGPLSGEPP**S^68^**YLNGEFPGDYGWD**T^82^**AGL**S^86^**ADPETFAK (1247.32, 4, 13) | 0.33±0.08 | 0.40±0.10 |
|  |  |  |  | VLYLGPLSGEPPSYLNGEFPGDYGWDTAGL**S^86^**ADPETFAK (1247.10, 4, 12) | 0.47±0.04 | 0.55±0.06 |
| Light harvesting chlorophyll *a*/*b* binding protein of LHCII type 1-like (Lhcb1) | CAA32109 | Chl | Light harvesting, state transition | VLYLGPLSGREPP**S^65^**YLTGEFPGDYGWDTAGLSADPETFAK (1247.61, 4, 13) | 0.45±0.01 | 0.55±0.07 |
| Light-harvesting chlorophyll *a*/*b* binding protein CP29, chloroplastic (CP29) | 1908421A | Chl | PSII disassembly, energy dissipation | LAQNLAGEIIG**T^108^**RFEDADVK (716.39, 4, 11) | 1.94±0.49 | 1.46±0.14 |
| Light-harvesting chlorophyll *a*/*b* binding protein CP29.2, chloroplastic (CP29) | CDI44415 | Chl | PSII disassembly, energy dissipation | PAEYLQYDVDSLDQNLAQNLAGEIIG**T^108^**R (1164.24, 3, 13) | 1.32±0.26 | 1.66±0.15 |
| Light-harvesting chlorophyll *a*/*b* binding protein CP29.2, chloroplastic (CP29) | XP_003562892 | Chl | PSII disassembly, energy dissipation | PAEYLQYDPD**S^95^**LDQNLAQNLAGEVIGTRFEDADIK (1537.76, 3, 10) | 17.46±10.55 | 16.77±7.96 |
| Light-harvesting chlorophyll *a*/*b* binding protein CP26, chloroplastic (CP26) | EMT03206 | Chl | Energy dissipation | TGALLLDGN**T^165^**LNYFGN**S^172^**IPINLILAVVAEVVLVGGAEYYR (1114.07, 4, 13) | 1.60±0.07 | - |
|  |  |  |  | TGALLLDGNTLNYFGN**S^172^**IPINLILAVVAEVVLVGGAEYYR (1201.63, 4, 19) | 1.15±0.36 | 1.74±0.14 |
| **Photosystem II related protein (5)** | | | | | | |
| Photosystem II subunit S (PsbS) | XP_003564708 | Chl | Energy dissipation | GIL**S^122^**QLNLETGIPIYEAEPLLLFFILFTLLGAIGALGDR (1207.64, 4, 14) | 1.30±0.19 | 1.80±0.13 |
| Photosystem II 43 kDa protein (CP43) | ABC02751 | Chl | PSII core protein | TLFNG**T^20^**FVLAGR (709.84, 2, 8) | 1.68±0.10 | 1.03±0.12 |
| Photosystem II reaction center protein H (PsbH) | P69555 | Chl | D1 maturation and incorporation | A**T^3^**QTVEDSSKPR (1044.01, 2, 14) | 2.60±1.04 | 3.28±0.50 |
|  |  |  |  | A**T^3^**Q**T^5^**VEDSSKPRPK (1308.68, 2, 14) | 3.69±1.05 | - |
| Predicted protein, oxygen-evolving enhancer protein 1, chloroplastic (PsbO)* | CCO16140 | Chl | Photosynthetic oxygen evolution, PSII D1 repair | GGSTGYDNAVALPAR**S^264^**DADDLQKENNK (954.97, 4, 13) | 0.97±0.02 | 0.62±0.01 |
| Predicted protein, containing pfam11493, thylakoid soluble phosphoprotein of 9 kDa domain (TSP9)* | BAJ97488 | Chl | State transition | VDGPAPSAGG**T^87^**ASR (834.92, 2, 14) | 1.42±0.08 | 1.74±0.05 |
| **Calvin cycle (9)** | | | | | | |
| Expressed protein, ribulose bisphosphate carboxylase/oxygenase activase (RCA)* | ABA95524 | Chl | Activate RuBisCO | GLAYDI**S^71^**DDQQDITR (1199.59, 2, 9) | 0.87±0.17 | 0.60±0.02 |
| Ribulose-1,5-bisphosphate carboxylase/oxygenase large subunit (RBL) | ABB03412 | Chl | Combines CO_2_ to produce 3-phosphoglycerate | GLDF**T^181^**KDDENVNSQPFMR (859.71, 3, 13) | 1.33±0.08 | 1.56±0.06 |
|  |  |  |  | V**T^27^**PQPGVPPEEAGAAESSTGTWTTVWTDGLTSLDR (1000.47, 4, 12) | 4.97±0.23 | 1.23±0.25 |
| Ribulose-1,5-bisphosphate carboxylase/oxygenase large subunit (RBL) | CAB85674 | Chl | Combines CO_2_ to produce 3-phosphoglycerate | AAFRVTPRPGVPPEEAGAAVAAES**S^62^**TGTWTTVWTDGLTSLDR (1203.10, 4, 12) | 1.59±0.01 | 1.10±0.38 |
| Ribulose-1,5-bisphosphate carboxylase/oxygenase large subunit (RBL) | CAA94018 | Chl | Combines CO_2_ to produce 3-phosphoglycerate | PGVPPEEAGAEVAAES**S^53^**TGTWTTVWTDGLTSLDR (1291.28, 3, 9) | 1.78±0.19 | 1.48±0.48 |
| Ribulose-1,5-bisphosphate carboxylase/oxygenase large subunit (RBL) | CAC04358 | Chl | Combines CO_2_ to produce 3-phosphoglycerate | VTPQPGVPAEEAGAAVDAE**S^51^**STGTWTTVWTDGLTSLDR (1420.37, 3, 13) | 2.00±0.66 | 1.34±0.02 |
| Ribulose-1,5-bisphosphate carboxylase/oxygenase large subunit (RBL) | AAG43944 | Chl | Combines CO_2_ to produce 3-phosphoglycerate | V**T^36^**PQPGVPPGGAGAAVAAES**S^55^**TGTWTTVWTDGLTSLDR (1392.66, 3, 15) | 1.77±0.22 | 1.26±0.21 |
|  |  |  |  | VTPQPGVPPGGAGAAVAAE**S^54^S^55^**TGTWTTVWTDGLTSLDR (1044.50, 4, 13) | 1.91±0.29 | 1.17±0.01 |
| Ribulose-1,5-bisphosphate carboxylase/oxygenase large subunit (RBL) | CAA93205 | Chl | Combines CO_2_ to produce 3-phosphoglycerate | VTPQPGVPAEEAGAAVAAE**S^54^**STGTW**T^60^**TVWTDGLTSLDR (795.56, 6, 14) | 0.51±0.14 | 0.80±0.07 |
|  |  |  |  | VTPQPGVPAEEAGAAVAAES**S^55^**TGTWTTVWTDGLTSLDR (1473.04, 3, 12) | 2.56±0.54 | 2.53±0.90 |
| Ribulose-1,5-bisphosphate carboxylase/oxygenase large subunit (RBL) | AFA27686 | Chl | Combines CO_2_ to produce 3-phosphoglycerate | VTPQPGVPPEEAGAAVAGEIG**T^69^**W**T^71^**TVWTDGLTSLDR (1040.50, 4, 16) | 2.17±0.51 | 2.74±0.18 |
|  |  |  |  | VTPQPGVPPEEAGAAVAGEIGTW**T^71^T^72^**VWTDGLTSLDR (1386.99, 3, 15) | 2.16±0.03 | 2.44±0.17 |
| Glyceraldehyde-3-phosphate dehydrogenase A, chloroplastic (GAPDH) | EMT31124 | Chl | Reversibly converts 1,3-BPG to GAP | GDS**S^92^**PLEVIAINDTGGVK (820.77, 3, 17) | 1.72±0.08 | 0.99±0.53 |
| **Carbohydrate and energy metabolism (12)** | | | | | | |
| **Energy metabolism (3)** | | | | | | |
| ATP synthase subunit alpha, chloroplastic | EMS64844 | Chl | ATP synthesis | NPLIAAA**S^9^**VIAAGLAVGLA**S^21^**IGPGVGQGTAAGQAVEGIAR (1243.97, 3, 13) | 1.18±0.37 | 1.62±0.13 |

**Table S8 Na_2_CO_3_-responsive phosphoproteins in alkaligrass leaves**

Table S8 *(continued from previous page.)*

| **Protein name** | **Accession no.** | **Loc** | **Biological function** | **Peptide sequence (m/z, charge, score)** | **Ratio of phosphopeptide abundance (mean ± S.D.)** | |
| --- | --- | --- | --- | --- | --- | --- |
|  |  |  |  |  | **150 mM/0 mM** | **200 mM/0 mM** |
| ATP synthase CF1 alpha subunit | AEJ10071 | Chl | ATP synthesis | **T^43^**GLGQVMSGELVEFAEGTR (793.72, 3, 12) | 2.01±0.06 | 1.80±0.05 |
| ATP synthase beta subunit | ABR67212 | Chl | ATP synthesis | GFQLIL**S^445^**GELDALPEQAFYLVGNIDEASTK (959.00, 4, 11) | 1.06±0.03 | 1.97±0.38 |
| **Glycolysis (3)** | | | | | | |
| Glyceraldehyde-3-phosphate dehydrogenase (GAPDH) | AFS33113 | Cyt | Reversibly converts 1,3-BPG to GAP | FGIVEGLMTTVHAM**T^184^**ATQK (681.35, 4, 8) | - | 1.90±0.14 |
| Hypothetical protein, phosphoglycerate kinase, cytosolic (PGK)* | XP_006300324 | ^#^Cyt | Reversibly catalyzes 3-PG to produce 1,3-BPG | LSELLGVEVVMAND**S^98^**IGEEVQKLVAALPEGGVLLLENVR (1129.11, 4, 12) | 1.57±0.01 | 1.16±0.15 |
| 2,3-bisphosphoglycerate-independent phosphoglycerate mutase (PGAM) | XP_003564482 | Cyt | Catalyzes the interconversion of 3PGA and 2PGA | AHGTAVGLPSDDDMGN**S^81^**EVGHNALGAGR (1036.13, 3, 21) | 1.63±0.11 | 1.17±0.12 |
| **Pyruvate metabolism (1)** | | | | | | |
| Phosphoenolpyruvate carboxylase (PEPC) | BAD36412 | Cyt | Catalyzes HCO_3_^-^ and PEP to form oxaloacetate | LS**S^18^**IDAQLR (693.87, 2, 13) | 2.25±0.23 | 1.28±0.11 |
| **Starch and sucrose metabolism (4)** | | | | | | |
| Phosphoglucomutase, cytoplasmic (PGM) | EMT08717 | ^#^Cyt, Chl | Reversibly converts G1P to G6P | ATGAFILTA**S^99^**HNPGGPTEDFGIK (1103.91, 3, 13) | 1.58±0.03 | 0.85±0.06 |
| UDP-glucose 6-dehydrogenase (UGDH) | EMT06309 | Cyt | Reversibly converts UDP-glucose to UDP-glucuronate | FDWDHPVHLQPM**S^337^**PTTTK (942.47, 3, 12) | 0.56±0.02 | 0.60±0.01 |
| UDP-glucose 6-dehydrogenase (UGDH) | EMT21987 | ^#^Cyt | Reversibly converts UDP-glucose to UDP-glucuronate | FDWDHPMHLQPT**S^393^**PTAVK (699.60, 4, 12) | 0.70±0.02 | 0.60±0.02 |
| Sucrose-phosphate synthase (SPS) | EMS63629 | ^#^Cyt, Nuc | Catalyzes UDP-glucose and F6P to form UDP and S6P | SDDATEVSETD**S^638^**PGDSLR (1132.99, 2, 24) | 0.61±0.01 | 0.65±0.01 |
| **Other glycometabolism (1)** | | | | | | |
| Phosphoglycerate kinase, chloroplastic (PGK) | XP_003568189 | Chl | Reversibly catalyzes 3-PG to produce 1,3-BPG | PGVVALDEAVTVGSV**T^481^** (797.39, 2, 10) | 1.12±0.01 | 0.51±0.11 |
| **Stress and defense (2)** | | | | | | |
| Predicted protein, containing pfam12481, aluminium induced protein domain (AIP)* | XP_004960054 | Chl | Stress response | QVAHAPQELN**S^18^**PR (915.96, 2, 18) | 2.05±0.15 | 2.87±0.03 |
| Wheat cold induced 16 | BAN63108 | Cyt, Nuc | Stress response | QY**S^76^**SGGTEK (823.40, 2, 14) | 0.64±0.02 | 0.71±0.04 |
|  |  |  |  | QYS**S^77^**GGTEK (548.94, 3, 11) | 0.61±0.05 | 0.80±0.03 |
|  |  |  |  | GAA**S^130^**LSGK (689.88, 2, 11) | 1.21±0.47 | 0.39±0.03 |
| **Membrane and transporting (4)** | | | | | | |
| Hypothetical protein, fructokinase-like 2, chloroplastic (FLN)* | EMS47290 | ^#^Chl, Nuc | Chloroplast thylakoids development | VAEQL**S^113^**DDEGEDQSK (1169.55, 2, 14) | 0.58±0.01 | 0.76±0.06 |
| Zinc finger protein VAR3, chloroplastic (VAR3) | EMS67681 | Chl | Chloroplast development | SD**S^1098^**QVFLFANSK (677.69, 3, 12) | 1.69±0.18 | 1.29±0.06 |
| Predicted protein,villin-2-like* | BAJ91166 | ^#^Chl, Nuc | Actin reverse polymerization | AAAVAALSSVLTAEQSG**S^414^**SDNLR (868.11, 3, 22) | 1.43±0.32 | 1.61±0.12 |
| Hypothetical protein, containing pfam00335, tetraspanin family protein domain* | EMS54049 | PM | Intracellular trafficking | AMNKPAEYD**S^200^**DDEIIGTAR (934.12, 3, 13) | 0.86±0.07 | 1.52±0.02 |
| **Signaling (8)** | | | | | | |
| Predicted protein, containing cd05574, catalytic domain of phototropin-like* | AAC05084 | ^#^PM | Photoreceptor | DEDPLLD**S^384^**DDERPESFDDELR (964.42, 3, 13) | 0.53±0.07 | 1.06±0.18 |
| Calmodulin 3 (CaM) | AGW21711 | Cyt | Ca^2+^ sensor | TVMR**S^8^**LGQNPTEAELQAMINEVDADGNGTIDFPEFLNLMAR (1169.81, 4, 13) | 1.29±0.55 | 1.62±0.08 |
|  |  |  |  | SLGQNP**T^14^**EAELQAMINEVDADGNGTIDFPEFLNLMAR (1463.35, 3, 9) | 1.90±0.22 | 2.46±0.87 |
|  |  |  |  | SLGQNPTEAELQAMINEVDADGNG**T^32^**IDFPEFLNLMAR (1469.03, 3, 20) | 2.59±0.45 | 2.16±0.49 |
| Unknown, calmodulin (CaM)* | ABR18064 | Cyt | Ca^2+^ sensor | SLGQNPTEAELQDMI**S^54^**EVDADGNGTIDFPEFLNLMAR (1468.68, 3, 19) | 3.07±0.30 | 2.36±0.07 |
| Calmodulin (CaM) | AAR99410 | Cyt | Ca^2+^ sensor | SLGQNPTEAELQDMINEVDADGNGTIDFPEFLNL**T^73^**AR (1468.69, 3, 23) | 2.53±0.47 | 2.00±0.14 |
| Inactive receptor kinase-like | XP_004970381 | PM | Signal transduction | EEW**T^564^**AEVFDVDLLR (702.67, 3, 8) | 1.05±0.61 | 2.12±0.25 |
| Phytosulfokine receptor kinase (PSKR) | AAT01376 | PM | PSK signaling | A**S^345^**AEVLGK (731.91, 2, 14) | 0.61±0.01 | 1.28±0.32 |
| Hypothetical protein, containing cd01459, VWA copine domain* | EMT11740 | Nuc | Cellular signaling | SS**S^390^**FGQQTSGFQQSDSFKQR (1002.79, 3, 13) | 1.71±0.02 | 1.56±0.03 |
|  |  |  |  | SS**S^390^**FGQQTSGFQQSD**S^403^**FKQR (1002.79, 3, 13) | 2.03±0.28 | 1.70±0.07 |
| Auxin-repressed 12.5 kDa protein (ARP1) | ABA95233 | ^#^Cyt, Nuc | Hormone signaling | SLGANLFDRPQPN**S^108^**PTVYDWLYSDETR (1175.89, 3, 16) | 0.50±0.08 | 1.08±0.18 |
| **Gene expression, protein synthesis and turnover (11)** | | | | | | |
| Hypothetical protein, histone H2A* | XP_002465460 | Nuc | DNA modification | G**T^60^**GAPVYLAAVLEYLAAEVLELAGNAAR (1449.74, 2, 18) | - | 2.36±0.37 |

Table S8 *(continued from previous page.)*

| **Protein name** | **Accession no.** | **Loc** | **Biological function** | **Peptide sequence (m/z, charge, score)** | **Ratio of phosphopeptide abundance (mean ± S.D.)** | |
| --- | --- | --- | --- | --- | --- | --- |
|  |  |  |  |  | **150 mM/0 mM** | **200 mM/0 mM** |
| Histone H2A.6-like | XP_003569004 | Nuc | DNA modification | VG**S^55^**GAPV**Y^60^**LAAVLEYLAAEVLELAGNAAR (782.62, 4, 13) | 0.85±0.00 | 0.64±0.01 |
|  |  |  |  | PV**Y^60^**LAAVLEYLAAEVLELAGNAAR (967.52, 3, 16) | 1.37±0.11 | 1.97±0.10 |
|  |  |  |  | PVYLAAVLE**Y^67^**LAAEVLELAGNAAR (991.20, 3, 13) | 1.69±0.05 | 1.27±0.06 |
| Histone H2AX-like | XP_006479831 | Nuc | DNA modification | VGAGAPVYL**S^57^**AVLEYLAAEVLELAGNAAR (829.93, 4, 13) | 1.06±0.11 | 1.52±0.02 |
| Hypothetical protein, histone H2AX-like* | ESW34361 | Nuc | DNA modification | GSGSPVYL**S^61^**AVLEYLAAEVLELAGNAAR (967.49, 3, 14) | 1.37±0.11 | 1.97±0.09 |
| 30S ribosomal protein 1, chloroplastic | XP_003559644 | Chl | Protein synthesis | EWQTAAAAAFSE**S^189^**DVDEEEDEDELVEVIGAEDEETVLTK (1243.07, 4, 9) | 0.91±0.27 | 0.63±0.02 |
| 60S ribosomal protein L13-1 | EMT10064 | Cyt | Protein synthesis | AGDS**T^156^**PEELANATQVQGDYMPIAR (973.46, 3, 19) | 1.54±0.01 | 1.41±0.06 |
| Uncharacterized protein, pfam06273 plant specific eukaryotic initiation factor 4B* | XP_003562957 | Nuc | Protein synthesis | GVDALASDLEKT**S^372^**PVGR (801.76, 3, 15) | 0.83±0.20 | 0.59±0.10 |
| Translation initiation factor 5A | ABB29987 | Cyt | Protein synthesis | SD**T^4^**DEHHFESK (1031.47, 2, 13) | 0.62±0.04 | 0.75±0.12 |
| Predicted protein, heat shock cognate 70 kDa protein* | BAJ86014 | Cyt | Protein folding | VQDLLLLDVTPL**S^406^**QGLE (1119.10, 2, 7) | - | 0.48±0.06 |
| Cytosolic heat shock protein 90 | AAP87284 | Cyt | Protein folding | EI**S^220^**DDEDEEEK (1013.45, 2, 17) | 0.90±0.03 | 1.66±0.11 |
| 26S protease regulatory subunit 6A homolog | XP_003570339 | Cyt | Protein degradation | SS**S^4^**PTPAPAAAPAAPMAVDETEDDQL**S^28^**SMSTDDIVR (1264.20, 3, 8) | 0.79±0.02 | 0.42±0.05 |
| **Unknown (1)** | | | | | | |
| Hypothetical protein | EMT31442 | Nuc | Function unknown | GPSGFPGAGGSGSD**S^44^**DEPQEYYTGGEK (1107.83, 3, 16) | 0.64±0.03 | 0.92±0.26 |

*Note*: Proteins were identified by LC-MS/MS, protein names marked with an asterisk (*) have been edited by us depending on searching against NCBI non-redundant protein database for functional domain. Protein subcellular localization (Loc) predicted by softwares (YLoc, Cello, Plant-mPLoc, ngLOC, and ChloroP). Pounds (#) indicate the subcellular localizations were predicted based on literatures. Chl, chloroplast; Cyt, cytoplasm; Mit, mitochondria; Nuc, nucleus; PM, plasma membrane. The average ratios of phosphopeptides between control (0 mM) and treatment (150 mM and 200 mM Na_2_CO_3_ for 24 h) were quantified according to the isobaric tags for relative and absolute quantification (iTRAQ) from three biological replicates, error bar indicates ± standard deviation (S.D.). ‘-’ indicate that the phosphorylation site without quantitative information in three replicates. 1,3-BPG, 1,3-bisphosphoglycerate; 3-PG, 3-phosphoglycerate; 2PGA, 2-phosphoglycerate; 3PGA, 3-phosphoglycerate; F6P, fructose 6-phosphate; G1P, glucose 1-phosphate; G6P, glucose 6-phosphate; GAP, glyceraldehyde 3-phosphate; PEP, phosphoenolpyruvate; PSK, phytosulfokine; S6P, sucrose-6-phosphate; UDP-glucose, uridine diphosphate glucose.
