# Supplementary material for: Na2CO3-responsive Photosynthetic and ROS Scavenging Mechanisms in Chloroplasts of Alkaligrass Revealed by Phosphoproteomics
Source: Genomics Proteomics Bioinformatics. 2020 Jul 16;18(3):271–88. doi: 10.1016/j.gpb.2018.10.011 (PMC7801222; doi:10.1016/j.gpb.2018.10.011)
Supplement: Supplementary Table S10 [file mmc12.docx]

**Table S10 Na_2_CO_3_-responsive phosphoproteins in chloroplasts from alkaligrass leaves**

| **Protein name** | **GenBank Accession No.** | **Biological function of the identified protein** | **Sequence of the identified phosphopeptide** | **Ratio of phosphopeptide** **abundance**  **(mean ± S.D.)** | |
| --- | --- | --- | --- | --- | --- |
|  |  |  |  | **150 mM/0 mM** | **200 mM/0 mM** |
| **Photosynthesis (48)** | | | | | |
| Chlorophyll *a*/*b* binding protein (18) | | | | | |
| Light harvesting complex I chlorophyll *a*/*b* binding protein (Lhca2) | ^#^CAA59049 | Light harvesting | APERPIWFPG**S^57^**TPPPWLDG**S^66^**LPGDFGFDPWGLGSDPESLR | 0.66±0.08 | 0.60±0.04 |
| Hypothetical protein, photosystem I light-harvesting complex type 4 protein (Lhca4)* | ERM94529 | Light harvesting, energy dissipation | NPG**S**^166^VNQDPIFK | 1.87 ± 0.06 | 3.05 ± 0.35 |
| Light harvesting complex I chlorophyll *a*/*b* binding protein (Lhca5) | ^#^XP_003618083 | Light harvesting | QSL**S^66^**YLDGSLPGDFGFDPLGLSDPEGTGGFIEPR | 1.83±0.01 | 1.22±0.28 |
| Predicted protein, chlorophyll *a*/*b* binding protein of LHCII type 1-like (Lhcb1)* | BAK03973 | Light harvesting, state transition | VAA**S**^46^SSPWYGSDR  VAASS**S**^48^PWYGSDR  VAASSSPWYG**S**^53^DR | 1.26 ± 0.01  1.23 ± 0.03  1.12 ± 0.02 | 1.83 ± 0.13  1.66 ± 0.15  1.52 ± 0.03 |
| Chlorophyll *a*/*b* binding protein of LHCII type 1-like (Lhcb1) | ADL41158 | Light harvesting, state transition | AKPV**S**^45^SGSPWYGSDR  AKPVSSG**S**^48^PWYGSDR  AKPVSSGSPWYG**S**^53^DR | 1.25 ± 0.10  1.39 ± 0.29  1.59 ± 0.62 | 1.88 ± 0.20  1.72 ± 0.12  1.56 ± 0.04 |
| Os03g0592500, chlorophyll *a*/*b* binding protein of LHCII type I (Lhcb1)* | BAF12500 | Light harvesting, state transition | TVK**S**^41^APQSIWYGPDRPK | 2.20 ± 0.33 | 7.46 ± 4.12 |
| Predicted protein, chlorophyll *a*/*b* binding protein of LHCII type 1-like (Lhcb1)* | BAJ85110 | Light harvesting, state transition | QVS**S**^46^GSPWYGADR | 1.59 ± 0.05 | 2.03 ± 0.07 |
| Light harvesting chlorophyll *a*/*b* binding protein of LHCII type 1-like (Lhcb1) | ^#^CDI44335 | Light harvesting, state transition | VAGGPLGEVVDPLYPGG**S^193^**LDPLGLADDPEAFAELK | 1.34±0.15 | 1.65±0.20 |
| Light harvesting chlorophyll *a*/*b* binding protein of LHCII type 1-like (Lhcb1) | ^#^EMS50795 | Light harvesting, state transition | VLYLGPLSGEPP**S^68^**YLTGEFPGDYGWDTAGLSADPETFAK | 1.93±0.34 | 1.78±0.32 |
|  |  |  | VLYLGPLSGEPP**S^68^**YL**T^71^**GEFPGDYGWD**T^82^**AGLSADPETFAK | 0.44±0.05 | 0.42±0.04 |
| Light harvesting chlorophyll *a*/*b* binding protein of LHCII type 1-like (Lhcb1) | ^#^EMT11232 | Light harvesting, state transition | VLYLGPL**S^63^**GDPP**S^68^**YLTGEFPGDYGWD**T^82^**AGLSADPETFAK | 0.36±0.16 | 0.47±0.13 |
| Light harvesting chlorophyll a-b binding protein of LHCII type 1-like (Lhcb1) | ^#^EMT29003 | Light harvesting, state transition | VLYLGPLSGEPP**S^68^**YLNGEFPGDYGWD**T^82^**AGL**S^86^**ADPETFAK | 0.33±0.08 | 0.40±0.10 |
|  |  |  | VLYLGPLSGEPPSYLNGEFPGDYGWDTAGL**S^86^**ADPETFAK | 0.47±0.04 | 0.55±0.06 |
| Light harvesting chlorophyll *a*/*b* binding protein of LHCII type 1-like (Lhcb1) | ^#^CAA32109 | Light harvesting, state transition | VLYLGPLSGREPP**S^65^**YLTGEFPGDYGWDTAGLSADPETFAK | 0.45±0.01 | 0.55±0.07 |
| Chlorophyll *a*/*b* binding protein of LHCII type III (Lhcb3) | P27523 | Light harvesting, state transition | I**T**^44^MGNDLWYGPDR | 1.13 ± 0.25 | 1.98 ± 0.16 |
| Light-harvesting chlorophyll *a*/*b* binding protein CP29, chloroplastic (CP29) | ^#^1908421A | PSII disassembly, energy dissipation | LAQNLAGEIIG**T^108^**RFEDADVK | 1.94±0.49 | 1.46±0.14 |
| Light-harvesting chlorophyll *a*/*b* binding protein CP29.2, chloroplastic (CP29) | ^#^CDI44415 | PSII disassembly, energy dissipation | PAEYLQYDVDSLDQNLAQNLAGEIIG**T^108^**R | 1.32±0.26 | 1.66±0.15 |
| Light-harvesting chlorophyll *a*/*b* binding protein CP29.2, chloroplastic (CP29) | ^#^XP_003562892 | PSII disassembly, energy dissipation | PAEYLQYDPD**S^95^**LDQNLAQNLAGEVIGTRFEDADIK | 17.46±10.55 | 16.77±7.96 |
| Light-harvesting chlorophyll *a*/*b* binding protein CP26, chloroplastic (CP26) | ^#^EMT03206 | Energy dissipation | TGALLLDGN**T^165^**LNYFGN**S^172^**IPINLILAVVAEVVLVGGAEYYR | 1.60±0.07 | - |
|  |  |  | TGALLLDGNTLNYFGN**S^172^**IPINLILAVVAEVVLVGGAEYYR | 1.15±0.36 | 1.74±0.14 |
| Chlorophyll *a*/*b* binding protein CP24 | XP_003579885 | Light harvesting, energy dissipation | TADNFAN**S**^186^TGDQGYPGGK | 2.09 ± 0.16 | 2.45 ± 0.10 |
| Photosystem II related protein (11) | | | | | |
| Photosystem II subunit S (PsbS) | ^#^XP_003564708 | Energy dissipation | GIL**S^122^**QLNLETGIPIYEAEPLLLFFILFTLLGAIGALGDR | 1.30±0.19 | 1.80±0.13 |
| Photosystem II 43 kDa protein (CP43) | ^#^ABC02751 | PSII core protein | TLFNG**T^20^**FVLAGR | 1.68±0.10 | 1.03±0.12 |
| Photosystem II reaction center protein H (PsbH) | A1EA37 | D1 maturation and incorporation | A**T**^3^QTVEDSSKPR  A**T**^3^Q**T**^5^VEDSSKPRPK | 0.61 ± 0.02  3.78 ± 1.18 | 0.72 ± 0.03  5.15 ± 2.20 |
| Photosystem II reaction center protein H (PsbH) | ACF08666 | D1 maturation and incorporation | A**T**^3^Q**T**^5^VEDSSKPKPR | 4.96 ± 0.17 | 6.80 ± 1.12 |
| Photosystem II reaction center protein H (PsbH) | ^#^P69555 | D1 maturation and incorporation | A**T^3^**QTVEDSSKPR | 2.60±1.04 | 3.28±0.50 |
|  |  |  | A**T^3^**Q**T^5^**VEDSSKPRPK | 3.69±1.05 | - |
| Oxygen-evolving enhancer protein 1 (PsbO) | EMT33794 | Photosynthetic oxygen evolution, PSII D1 repair | QLVATGKPE**S**^220^FSGPFLVPSYR | 0.32 ± 0.11 | 0.81 ± 0.14 |
| Predicted protein, oxygen-evolving enhancer protein 1, chloroplastic (PsbO)* | ^#^CCO16140 | Photosynthetic oxygen evolution, PSII D1 repair | GGSTGYDNAVALPAR**S^264^**DADDLQKENNK | 0.97±0.02 | 0.62±0.01 |
| PsbP domain-containing protein 1 (PsbP) | EMS62589 | Photosynthetic oxygen evolution, PSII activation | SYA**S**^184^NNELAVMPK | 0.47 ± 0.00 | - |
| Photosystem II subunit L (PsbL) | AAN32350 | CP43 assemble and PSII dimerization | **T**^2^ASNPNEQNVELNR | - | 48.38 ± 41.55 |
| Predicted protein, thylakoid lumenal 29 kDa protein (TL29)* | BAJ87776 | Stabilizing or assembling the lumenal side of PSII | TLYSAYGS**S**^220^GQWGFFDK | 0.63 ± 0.00 | 0.82 ± 0.13 |
| Predicted protein, containing pfam11493, thylakoid soluble phosphoprotein of 9 kDa domain (TSP9)* | ^#^BAJ97488 | State transition | VDGPAPSAGG**T^87^**ASR | 1.42±0.08 | 1.74±0.05 |
| Photosystem I related protein (5) | | | | | |
| Photosystem I iron-sulfur center (PsaC) | P0C359 | Carries the [4Fe–4S] F_A_ and F_B_ clusters | VYLGPE**T**^73^TR | 1.51 ± 0.01 | 1.39 ± 0.09 |
| Photosystem I reaction center subunit II (PsaD) | XP_003564108 | Provide the docking site for soluble ferredoxin | VFP**S**^154^GEVQYLHPK | 1.71 ± 0.16 | - |
| Photosystem I reaction center subunit IV (PsaE) | XP_003563195 | Provide the docking site for soluble ferredoxin | ESYWYNGTG**S**^106^VVTVDQDPNTR | 3.07 ± 1.10 | 4.51 ± 2.25 |

Table S10 *(continued from previous page.)*

| **Protein name** | **Accession no.** | **Biological function** | | | **Peptide sequence** | **Ratio of phosphopeptide abundance**  **(mean ± S.D.)** | | | | |  |
| --- | --- | --- | --- | --- | --- | --- | --- | --- | --- | --- | --- |
|  |  |  |  |  |  | **150 mM/0 mM** | | **200 mM/0 mM** | | |  |
| Photosystem I reaction center subunit IV (PsaE) | ACG30530 | | Provide the docking site for soluble ferredoxin | VNYAGV**S**^92^TNNYALDEVLEVK | | | 1.83 ± 0.31 | | | 1.74 ± 0.25 | |
| Photosystem I reaction center subunit VI (PsaH) | EMT19581 | Stabilizing PSI complex | | | GPQLPP**T**^136^PGPR | 5.82 ± 3.57 | | 4.35 ± 1.89 | | |  |
| Photosynthetic electron transfer chain related protein (3) | | | | | | | | | | |  |
| Cytochrome *f* (Cyt *f*) | A1EA21 | Electron transport | | | VQL**Y**^316^EMNF | 1.77 ± 0.15 | | 1.91 ± 0.17 | | |  |
| Predicted protein, containing pfam13806, rieske-like (2Fe-2S) domain (ISP)* | BAK01659 | Electron transport | | | AQPGSTA**S**^207^DVNIEEVR | 2.42 ± 0.74 | | 3.76 ± 0.98 | | |  |
| Ferredoxin-NADP reductase, chloroplastic (FNR) | KEH27588 | Electron transport | | | LVY**T**^168^NDAGEVVK | 1.23 ± 0.35 | | 2.18 ± 0.35 | | |  |
| Calvin cycle (11) | | | | | | | | | | |  |
| Ribulose bisphosphate carboxylase/oxygenase activase (RCA) | EEH57944 | Activate RuBisCO | | | LGIAPIIM**S**^166^AGELESGNAGEPAK | 0.27 ± 0.01 | | 0.41 ± 0.01 | | |  |
| Expressed protein, ribulose bisphosphate carboxylase/oxygenase activase (RCA)* | ^#^ABA95524 | Activate RuBisCO | | | GLAYDI**S^71^**DDQQDITR | 0.87±0.17 | | 0.60±0.02 | | |  |
| Ribulose-1,5-bisphosphate carboxylase/oxygenase large subunit (RBL) | ^#^ABB03412 | Combines CO2 to produce 3-phosphoglycerate | | | GLDF**T^181^**KDDENVNSQPFMR | 1.33±0.08 | | 1.56±0.06 | | |  |
|  |  |  |  |  | V**T^27^**PQPGVPPEEAGAAESSTGTWTTVWTDGLTSLDR | 4.97±0.23 | | 1.23±0.25 | | |  |
| Ribulose-1,5-bisphosphate carboxylase/oxygenase large subunit (RBL) | ^#^CAB85674 | Combines CO_2_ to produce 3-phosphoglycerate | | | AAFRVTPRPGVPPEEAGAAVAAES**S^62^**TGTWTTVWTDGLTSLDR | 1.59±0.01 | | 1.10±0.38 | | |  |
| Ribulose-1,5-bisphosphate carboxylase/oxygenase large subunit (RBL) | ^#^CAA94018 | Combines CO_2_ to produce 3-phosphoglycerate | | | PGVPPEEAGAEVAAES**S^53^**TGTWTTVWTDGLTSLDR | 1.78±0.19 | | 1.48±0.48 | | |  |
| Ribulose-1,5-bisphosphate carboxylase/oxygenase large subunit (RBL) | ^#^CAC04358 | Combines CO_2_ to produce 3-phosphoglycerate | | | VTPQPGVPAEEAGAAVDAE**S^51^**STGTWTTVWTDGLTSLDR | 2.00±0.66 | | 1.34±0.02 | | |  |
| Ribulose-1,5-bisphosphate carboxylase/oxygenase large subunit (RBL) | ^#^AAG43944 | Combines CO_2_ to produce 3-phosphoglycerate | | | V**T^36^**PQPGVPPGGAGAAVAAES**S^55^**TGTWTTVWTDGLTSLDR | 1.77±0.22 | | 1.26±0.21 | | |  |
|  |  |  |  |  | VTPQPGVPPGGAGAAVAAE**S^54^S^55^**TGTWTTVWTDGLTSLDR | 1.91±0.29 | | 1.17±0.01 | | |  |
| Ribulose-1,5-bisphosphate carboxylase/oxygenase large subunit (RBL) | ^#^CAA93205 | Combines CO_2_ to produce 3-phosphoglycerate | | | VTPQPGVPAEEAGAAVAAE**S^54^**STGTW**T^60^**TVWTDGLTSLDR | 0.51±0.14 | | 0.80±0.07 | | |  |
|  |  |  |  |  | VTPQPGVPAEEAGAAVAAES**S^55^**TGTWTTVWTDGLTSLDR | 2.56±0.54 | | 2.53±0.90 | | |  |
| Ribulose-1,5-bisphosphate carboxylase/oxygenase large subunit (RBL) | ^#^AFA27686 | Combines CO_2_ to produce 3-phosphoglycerate | | | VTPQPGVPPEEAGAAVAGEIG**T^69^**W**T^71^**TVWTDGLTSLDR | 2.17±0.51 | | 2.74±0.18 | | |  |
|  |  |  |  |  | VTPQPGVPPEEAGAAVAGEIGTW**T^71^T^72^**VWTDGLTSLDR | 2.16±0.03 | | 2.44±0.17 | | |  |
| Glyceraldehyde-3-phosphate dehydrogenase A, chloroplastic (GAPDH) | ^#^EMT31124 | Reversibly converts 1,3-BPG to GAP | | | GDS**S^92^**PLEVIAINDTGGVK | 1.72±0.08 | | 0.99±0.53 | | |  |
| Fructose-bisphosphate aldolase (FBA) | AAB70542 | Reversibly catalyze FBP to GAP and DHAP | | | GLVPSAG**S**^147^NNESWCQGLDGLASR | 31.27 ± 25.90 | | 33.01 ± 10.64 | | |  |
| **Carbohydrate and energy metabolism (12)** | | | | | | | | | | |  |
| ATP synthesis (9) | | | | | | | | | | |  |
| ATP synthase α subunit | KEH17711 | ATP synthesis | | | GEIIA**S**^125^ESR | 0.47 ± 0.02 | | 0.75 ± 0.02 | | |  |
| ATP synthase subunit alpha, chloroplastic | ^#^EMS64844 | ATP synthesis | | | NPLIAAA**S^9^**VIAAGLAVGLA**S^21^**IGPGVGQGTAAGQAVEGIAR | 1.18±0.37 | | 1.62±0.13 | | |  |
| ATP synthase CF1 alpha subunit | ^#^AEJ10071 | ATP synthesis | | | **T^43^**GLGQVMSGELVEFAEGTR | 2.01±0.06 | | 1.80±0.05 | | |  |
| ATP synthase β subunit | ABH02573 | ATP synthesis | | | AITLEEENK**S**^497^KK | 0.46 ± 0.01 | | 0.47 ± 0.05 | | |  |
| ATP synthase β subunit | CAB89989 | ATP synthesis | | | GRNTGGQPINA**T**^53^CEVQQLLGNNR | 3.72 ± 0.55 | | 3.75 ± 1.84 | | |  |
| ATP synthase β subunit | AAK72724 | ATP synthesis | | | D**T**^52^VRQQINVTCEVQQLLGNNR | 1.07 ± 0.05 | | 0.48 ± 0.06 | | |  |
| ATP synthase beta subunit | ^#^ABR67212 | ATP synthesis | | | GFQLIL**S^445^**GELDALPEQAFYLVGNIDEASTK | 1.06±0.03 | | 1.97±0.38 | | |  |
| ATP synthase ε subunit | ACF08646 | ATP synthesis | | | AEG**T**^110^KELVEAK | 0.56 ± 0.07 | | - | | |  |
| Hypothetical protein, ATP synthase subunit B* | EAY89475 | ATP synthesis | | | AELGGVKDA**S**^123^EEVR | 0.59 ± 0.07 | | - | | |  |
| Sucrose and fatty acid biosynthesis (2) | | | | | | | | | | |  |
| Sucrose-phosphate synthase (SPS) | AAQ14552 | Sucrose biosynthesis | | | LV**S**^174^DDEDEQSK | 0.76 ± 0.02 | | 0.62 ± 0.03 | | |  |
| Pyruvate dehydrogenase E1 component subunit alpha-2 (PDH) | Q8H1Y0 | Fatty acid biogenesis | | | YHGH**S**^296^MSDPGSTYR | 2.28 ± 0.26 | | 2.74 ± 0.20 | | |  |
| Other glycometabolism (1) |  |  | | |  |  | |  | | |  |
| Phosphoglycerate kinase, chloroplastic (PGK) | ^#^XP_003568189 | Reversibly catalyzes 3-PG to produce 1,3-BPG | | | PGVVALDEAVTVGSV**T^481^** | 1.12±0.01 | | 0.51±0.11 | | |  |
| **Stress and defense (2)** | | | | | | | | | | |  |
| Predicted protein, containing pfam12481, aluminium induced protein domain (AIP)* | BAK01943 | Stress response | | | QVAHAPQELN**S**^18^PR | 1.77 ± 0.16 | | 2.53 ± 0.18 | | |  |
| Predicted protein, containing pfam12481, aluminium induced protein domain (AIP)* | ^#^XP_004960054 | Stress response | | | QVAHAPQELN**S^18^**PR | 2.05±0.15 | | 2.87±0.03 | | |  |
| **Membrane and transporting (8)** | | | | | | | | | | |  |
| Na^+^/H^+^ antiporter | BAJ06107 | Na^+^ compartmentalization | | | GFVPFVPG**S**^524^PVER | - | | 1.91 ± 0.11 | | |  |
| Villin-2 | EMT05628 | Actin reverse polymerization | | | AAAVAALSSVLTAEQSG**S**^260^SDNLR | 1.43 ± 0.09 | | | 1.79 ± 0.24 | |  |
| Predicted protein,villin-2-like* | ^#^BAJ91166 | Actin reverse polymerization | | | AAAVAALSSVLTAEQSG**S^414^**SDNLR | 1.43±0.32 | | | 1.61±0.12 | |  |
| Predicted protein, protein curvature thylakoid 1A (CURT1A)* | BAK02001 | Induce thylakoid membrane curvature | | | ASSDDTSTSAA**S**^66^GDELVDDLK | 0.81 ± 0.08 | | | 2.13 ± 0.30 | |  |
| Dynamin-2A-like protein (DLP) | XP_004965129 | Thylakoid organization | | | QSH**S**^719^DGSLDTMAR | 1.53 ± 0.03 | | | 0.96 ± 0.02 | |  |
| Hypothetical protein, fructokinase-like 2 (FLN)* | EMS47290 | Chloroplast thylakoids development | | | VAEQL**S**^113^DDEGEDQSK | 0.56 ± 0.03 | | | 0.57 ± 0.05 | |  |

Table S10 *(continued from previous page.)*

| **Protein name** | **Accession No.** | **Biological function** | **Peptide sequence** | **Ratio of phosphopeptide abundance**  **(mean ± S.D.)** | | |
| --- | --- | --- | --- | --- | --- | --- |
|  |  |  |  | **150 mM/0 mM** | | **200 mM/0 mM** |
| Hypothetical protein, fructokinase-like 2, chloroplastic (FLN)* | ^#^EMS47290 | Chloroplast thylakoids development | VAEQL**S^113^**DDEGEDQSK | 0.58±0.01 | | 0.76±0.06 |
| Zinc finger protein VAR3, chloroplastic (VAR3) | ^#^EMS67681 | Chloroplast development | SD**S^1098^**QVFLFANSK | 1.69±0.18 | 1.29±0.06 | |
| **Signaling (2)** | | | | | | |
| Putative protein phosphatase 2C (PP2C) | EMT08603 | Signal transduction | SI**S**^313^ADGLNSLR | 0.98 ± 0.00 | | 2.67 ± 0.97 |
| Calcium sensing receptor, chloroplastic (CAS) | AAS00828 | Signal transduction | KLLPG**S**^384^VDG | 0.73 ± 0.01 | | 2.46 ± 0.19 |
| **Gene expression, protein synthesis, and turnover (8)** | | | | | | |
| RNA binding protein (RNA-BP) | EMT29522 | Transcription | GQDPQGM**S**^485^PGPGGR | 1.89 ± 0.21 | | 1.98 ± 0.07 |
| Hypothetical protein, containing cd12432, RNA recognition motif (RRM)* | EEC75374 | Transcription | SDSTASGD**S**^587^PKER | 3.02 ± 0.16 | | 1.75 ± 0.11 |
| Hypothetical protein, containing pfam07727, reverse transcriptase domain (RT)* | CAN61094 | Transcription | PID**T**^382^FIDVNIK | 0.90 ± 0.07 | | 7.99 ± 1.01 |
| Predicted protein, zinc finger protein (ZF)* | BAJ99149 | Transcription | LQ**S**^227^PGAQQYYGTSR | - | | 2.14 ± 0.48 |
| Serine/arginine-rich splicing factor 33-like (SRSF) | XP_004980077 | RNA processing | RG**Y**^29^GGGGGGGGGGGGGGGGGGGGGGR | 5.55 ± 0.78 | | 0.62 ± 0.04 |
| Predicted protein, probable alanine-tRNA ligase (AlaRS), chloroplastic* | ACO61321 | Protein synthesis | MA**S**^3^AASSSTETAAPK | 0.04 ± 0.02 | | - |
| 30S ribosomal protein 1, chloroplastic | ^#^XP_003559644 | Protein synthesis | EWQTAAAAAFSE**S^189^**DVDEEEDEDELVEVIGAEDEETVLTK | 0.91±0.27 | | 0.63±0.02 |
| ATP-dependent zinc metalloprotease FtsH 2 (FtsH), chloroplastic | EMT25919 | Protein degradation | QV**S**^378^VDVPDVR | 1.52 ± 0.06 | | 1.86 ± 0.04 |
| **Function unknown (4)** | | | | | | |
| NHL repeat-containing protein 2 | EMS60685 | Function unknown | TLDLTGVQPP**S**^1069^PKPK | 0.49 ± 0.07 | | - |
| Unnamed protein, no putative conserved domains have been detected* | CBF59389 | Function unknown | RESL**Y**^466^GSLS**S**^471^LEDDIVR | 0.01 ± 0.00 | | 0.80 ± 0.10 |
| Hypothetical protein, pfam04398, protein of unknown function* | DAA41082 | Function unknown | NLFFF**S**^215^RLAGR | 0.01 ± 0.00 | | 0.03 ± 0.01 |
| Hypothetical protein | XP_010104076 | Function unknown | EEEPEQYWQ**T**^77^AGER | 11 ± 3.25 | | 54 ± 23.02 |

*Note*: The name and functional categories of the proteins identified by LC-MS/MS searched against the NCBI non-redundant green plant database. * indicates protein whose name was edited based on search against NCBI non-redundant protein database for functional domain. # indicates the chloroplast-localized phosphoprotein that was identified in the leaf phosphoproteome. The phosphorylated amino acid residues in the identified phosphopeptides are put in bold. The average ratios of phosphopeptides were calculated from three biological replicates. Protein samples were prepared from leaves and chloroplasts of *Puccinellia tenuiflora* that were treated with 0 mM, 150 mM, or 200 mM Na_2_CO_3_ for 24 h. ‘-’ indicates the lack of quantitative information for phosphorylation sites in three replicates. 1,3-BPG, 1,3-bisphosphoglycerate; 3-PG, 3-phosphoglycerate; DHAP, dihydroxyacetone phosphate; FBP, fructose 1,6-bisphosphate; GAP, glyceraldehyde 3-phosphate.
