# Supplementary material for: Na2CO3-responsive Photosynthetic and ROS Scavenging Mechanisms in Chloroplasts of Alkaligrass Revealed by Phosphoproteomics
Source: Genomics Proteomics Bioinformatics. 2020 Jul 16;18(3):271–88. doi: 10.1016/j.gpb.2018.10.011 (PMC7801222; doi:10.1016/j.gpb.2018.10.011)
Supplement: Supplementary Table S15 [file mmc17.docx]

**Table S15 Primers used for overexpression of *PtFBA* in *Synechocystis* 6803**

| **Name** | **Primer sequence (5’–3’)** | **Purpose** |
| --- | --- | --- |
| Primers used for *PtFBA* gene clone | | |
| *PtFBA*-FP-1 | CAATGGCGTCTGCTACTCTCCTCA | Amplification of full length cDNA of *PtFBA* |
| *PtFBA*-RP-1 | CGTCAGTTCAGGTCGCTCCACT |  |
| Primers used to construct the P*psbAII-PtFBA* expression vector | | |
| *PtFBA*-FP-2 | GGAATTCCATATGGCGTCTGCTACTCT | Amplification of *PtFBA* encoding gene fragment |
| *PtFBA*-RP-2 | GGAATTCCATATGCTAGAGATTGGTCAGTTC |  |
| *slr0168*-FP | GAGTAGTTCCCTCAACACCAGT | Segregation analysis |
| *slr0168*-RP | TTCCAGGCCACATTGTTGTC |  |
| Primers used for RT-PCR | | |
| *PtFBA*-FP-3 | GGAATTCCATATGGCGTCTGCTACTCT | *PtFBA* transcript |
| *PtFBA*-RP-3 | GGAATTCCATATGCTAGAGATTGGTCAGTTC |  |
| *16S rRNA*-FP | CGACTGCTAATACCCAATGTGC | *16S rRNA* transcript |
| *16S rRNA*-RP | GTCCCTCAGTGTCAGTTTCAGC |  |
